# Design, synthesis, and biological evaluation of thiazole/thiadiazole carboxamide scaffold-based derivatives as potential c-Met kinase inhibitors for cancer treatment

**DOI:** 10.1080/14756366.2023.2247183

**Published:** 2023-08-29

**Authors:** Xiang Nan, Qiu-Xu Wang, Shao-Jun Xing, Zhi-Gang Liang

**Affiliations:** a Department of Stomatology, Shenzhen Second People’s Hospital, Shenzhen, China; b School of Biomedical Engineering, National-Regional Key Technology Engineering Laboratory for Medical Ultrasound, Guangdong Key Laboratory for Biomedical Measurements and Ultrasound Imaging, Shenzhen University Medical School, Shenzhen, China

**Keywords:** Thiazole, thiadiazoles, c-Met inhibitors, biological evaluation, docking study

## Abstract

As part of our continuous efforts to discover novel c-Met inhibitors as antitumor agents, four series of thiazole/thiadiazole carboxamide-derived analogues were designed, synthesised, and evaluated for the *in vitro* activity against c-Met and four human cancer cell lines. After five cycles of optimisation on structure–activity relationship, compound **51am** was found to be the most promising inhibitor in both biochemical and cellular assays. Moreover, **51am** exhibited potency against several c-Met mutants. Mechanistically, **51am** not only induced cell cycle arrest and apoptosis in MKN-45 cells but also inhibited c-Met phosphorylation in the cell and cell-free systems. It also exhibited a good pharmacokinetic profile in BALB/c mice. Furthermore, the binding mode of **51am** with both c-Met and VEGFR-2 provided novel insights for the discovery of selective c-Met inhibitors. Taken together, these results indicate that **51am** could be an antitumor candidate meriting further development.

## Graphical Abstract

**Figure:**
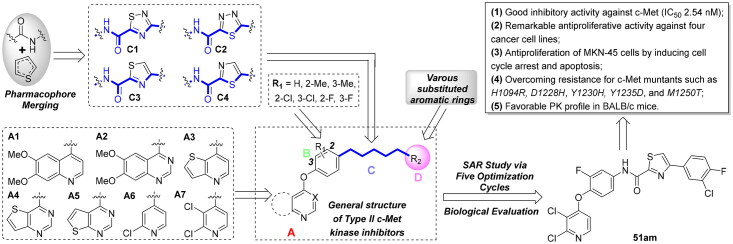

## Introduction

Cancer, a leading cause of death second only to cardiovascular diseases, is a serious public health issue to human worldwide according to WHO.^1^ The uncontrolled proliferation of abnormal cells is a predominant feature of cancer initiation and progression. Protein kinases, which transfer the terminal phosphate group of ATP to tyrosine, threonine, or serine residues of proteins, play critical roles in plenty of processes such as cell growth, differentiation, metastasis, homeostasis, and death.^2–5^ Protein kinases have been intensively explored for drug discovery, resulting ∼80+ small molecule kinase inhibitors (SMKIs) that have been commercialised for targeted therapy of cancer and other diseases.^6^ However, SMKIs are still emerged with two critical challenges: (i) lack of exquisite selectivity for target kinase versus other kinases and (ii) acquired resistance after different periods of clinical usage.

**Figure 1.**
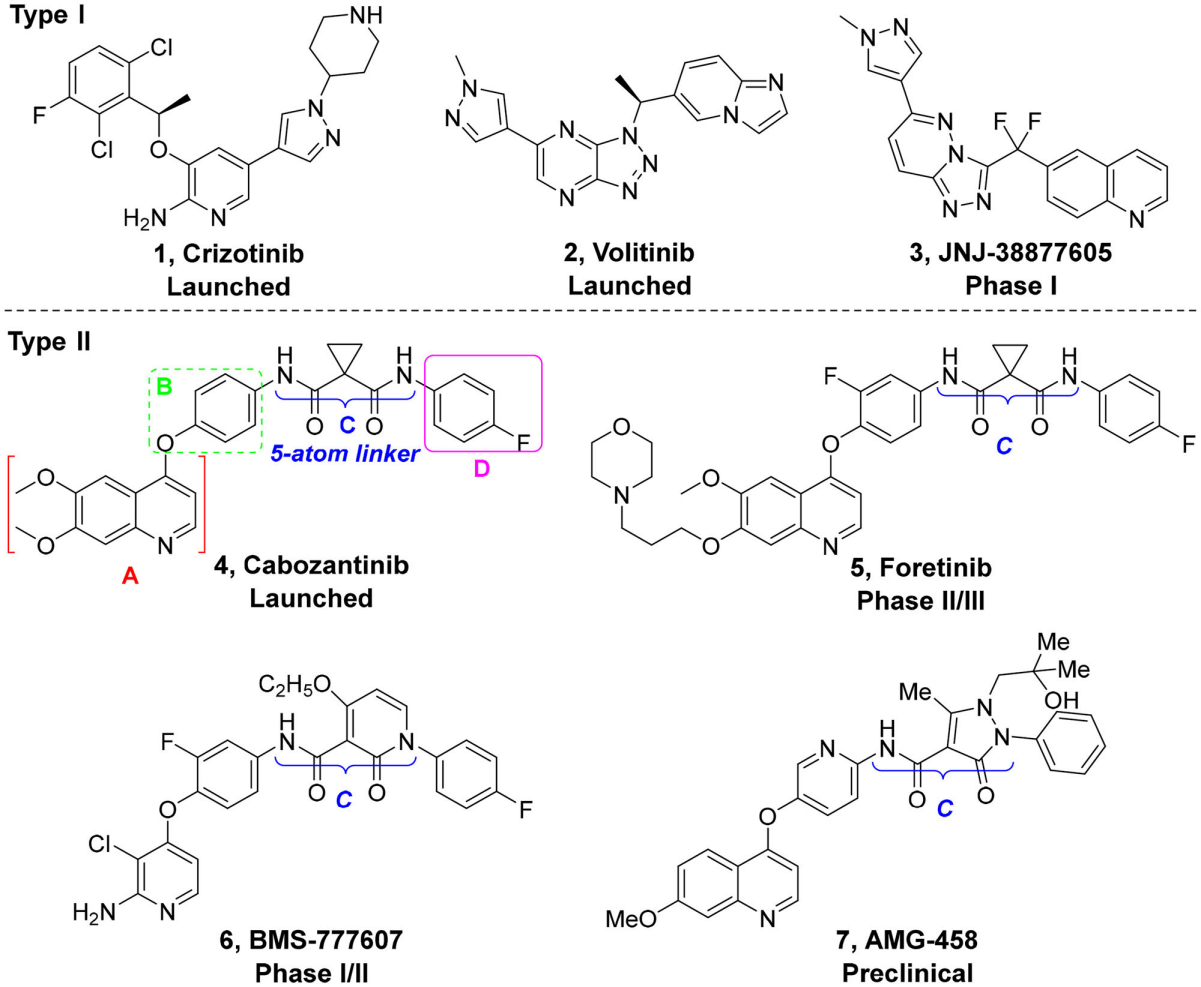
Structures of representative small molecule c-Met inhibitors.

Cellular-mesenchymal epithelial transition factor (c-Met) is a structurally unique transmembrane member of the receptor tyrosine kinases.^7^^,^^8^ Upon activation by hepatocyte growth factor (HGF) and/or cross-talk with other signalling pathways, the intracellular C-terminal docking domain recruits and then activates a broad range of downstream signalling effectors and adaptors.^9–11^ Deregulated HGF/c-Met signalling hijacks a series of cellular processes and promotes the tumours initiation and progression. Moreover, the overexpression of HGF and/or c-Met has been detected in various human solid tumours and is closely associated with poor prognosis and resistance to other kinase inhibitors.^12^ Small-molecule c-Met inhibitors have attracted considerable attention because it can inhibit c-Met irrespective of the activation mechanism.^13–16^ Based on the structural characteristics and bonding modes to c-Met, the reported c-Met inhibitors can be categorised into two types as shown in Figure 1. Type I c-Met inhibitors bind to the ATP binding pocket of c-Met with a U-shaped conformation. On the contrary, type II c-Met inhibitors not only bind to the ATP active site but also extend the inactive DFG out conformation engaging in hydrophobic interactions. Similar to other kinase inhibitors, the efficacy of c-Met inhibitors is also compromised due to the spot mutations at the ATP-binding site, such as Y1230C and D1228V/N/H for type I inhibitors,^17–19^ and due to low selectivity for type II inhibitors.^11^^,^^12^

Accumulating evidencesuggests that the mutations near the active site of c-Met may be overcome by type II inhibitors since their binding interactions with c-Met extend beyond the entrance of c-Met’s active site resulting in additional hydrophobic interactions.^20–24^ The structure of type II c-Met inhibitors can be disconnected into four moieties (A–D) based on their interactions with c-Met (Figure 1), among which moiety C serves as a linker to form H-bonds and plays a vital role against c-Met.^25–27^ Moreover, the low kinase selectivity associated with type II c-Met inhibitors could be improved significantly by modifying the moiety C.^28–30^ By analysing the conformation of type II inhibitors with c-Met, we found that the linear moiety C commonly assumed a pseudocyclic via intramolecular H-bonding; therefore, incorporation of various rings into the moiety C could possess the inherent advantage to generate novel c-Met inhibitors, exemplified by BMS-777607 (**6**) ^31^ and AMG-458 (**7**) .^28^^,^^32^ Pharmacophore merging, an efficient strategy to boost efficacy and overcome drug resistance, has been applied widely in drug design and discovery.^33^ The amide functional group is found in many biologically active molecules, including peptides, proteins and clinically approved drugs, and displays unique ability to form H-bonding interaction.^34^ Thiazole and thiadiazole are two classes of five-membered heterocycles and their derivatives exhibit important pharmaceutical activities such as antitumor, antibacterial, antifungal, antitubercular, and analgesic.^35–37^ Thiadiazole is considered as the bioisostere of pyrimidine and oxadiazole; its mesoionic nature allows analogues to cross cellular membranes and interact with biological targets and the sulphur atom imparts improved liposolubility.^38–41^ Recent studies have demonstrated the utilisation of thiadiazole as a promising scaffold for antitumor drug discovery by inhibiting diverse targets, such as histone deacetylase (HDAC), c-Src/Abl tyrosine kinase, focal adhesion kinase (FAK) and tubulin polymerisation for 1,3,4-thiadiazoles,^38^^,^^39^ as well as cathepsin K and glycogen synthase kinase-3β for 1,2,4-thiadiazoles.^42^ Thiazole is another privileged scaffold in lead identification given its ability to interact with crucial biological targets involved in diseases ^43^^,^^44^ and is also found in several approved drugs and many drug candidates.^45^^,^^46^ Moreover, introducing thiadiazole/thiazole moiety can be used to tune whole physicochemical and pharmacokinetic properties of candidates. In addition, thiazole and thiadiazole are assumed to preferentially form H-bonding with c-Met given its electron-rich characteristics.^47^

**Figure 2.**
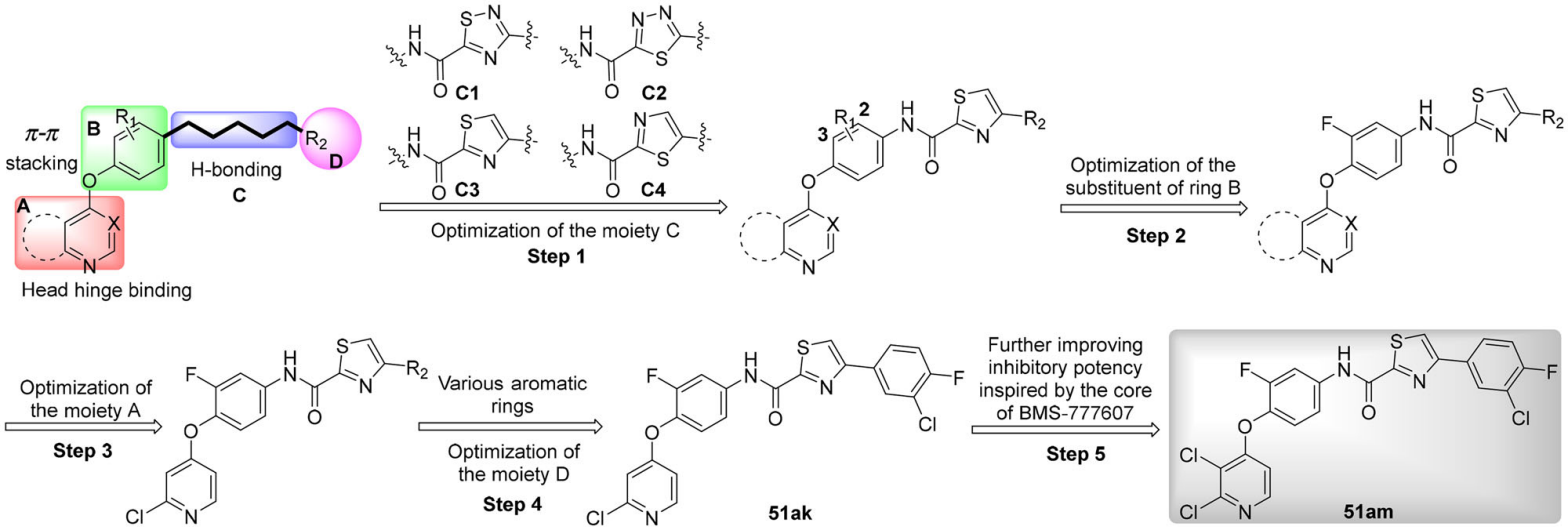
Design strategy of target compounds.

Given the above considerations and insights, in this work, we postulated that incorporation of the intrinsic antitumor functional fragments, thiazole/thiadiazole carboxamide, into the moiety C could facilitate the discovery of novel type II c-Met inhibitors with improved efficacy and selectivity, and reduced toxicity. Meanwhile, modifications of the moieties A, B, and D were performed simultaneously to investigate the effect on activity and improve the potential drug-likeness. Therefore, based on the general chemical structure of type II c-Met inhibitors and the rational drug design, a small-molecular library of 40 novel derivatives was prepared and evaluated (Figure 2).

## Results and discussion

### Chemistry

**Scheme 1.**
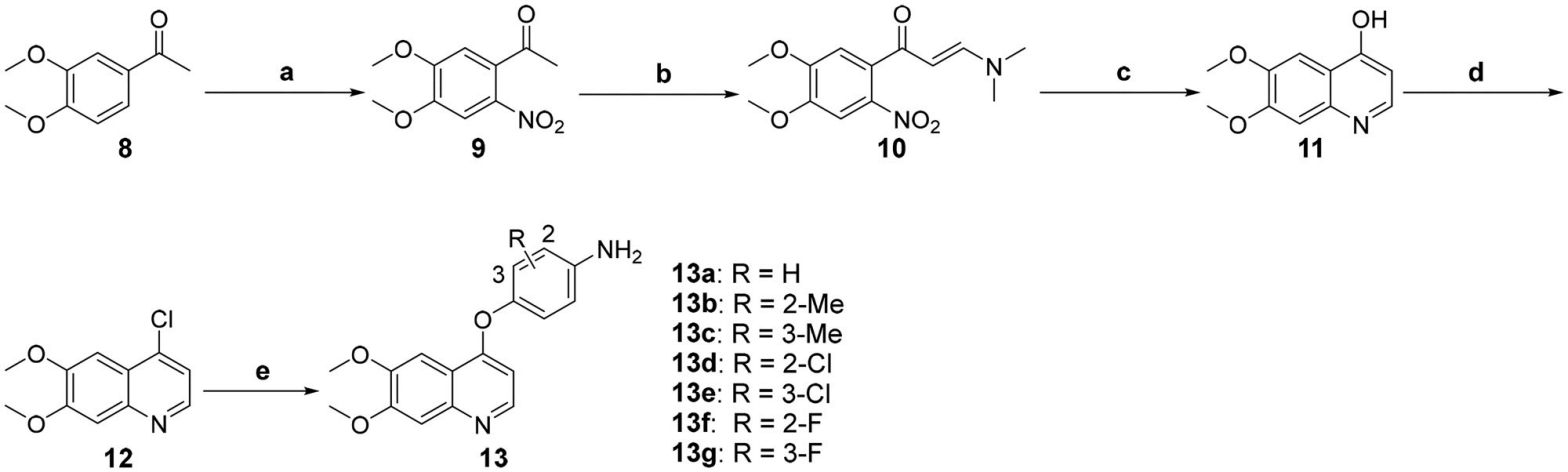
Synthesis of the intermediates **13a**–**13g**; reagents and conditions: (a) conc. HNO_3_ (40%), 0 °C, overnight; (b) DMF-DMA, toluene, reflux, 10 h; (c) Fe (powder), AcOH, 80 °C, 2 h; (d) POCl_3_, DMF (cat.), reflux, 1 h; (e) (1) 4-nitrophenols, PhCl, 140 °C, 20 h; (2) SnCl_2_, EtOH, 70 °C, 6 h.

The anilines **13a**–**13g** were synthesised starting from 3,4-dimethoxyacetophenone **8** as shown in Scheme 1.^22^^,^^29^^,^^30^^,^^48^ Compound **8** was subjected to regioselective nitration to afford *o*-nitroacetophenone **9**, which underwent aminomethylenation with DMF-DMA to give α,β-unsaturated ketone **10**. Cyclisation of **10** in the presence of Fe powder and AcOH followed by chlorination with POCl_3_ furnished chloride **12**. Intermediate **12** was coupled with various substituted *p*-nitrophenols and the resulting aryl ethers were reduced with SnCl_2_ to give the desired compounds.

**Scheme 2.**
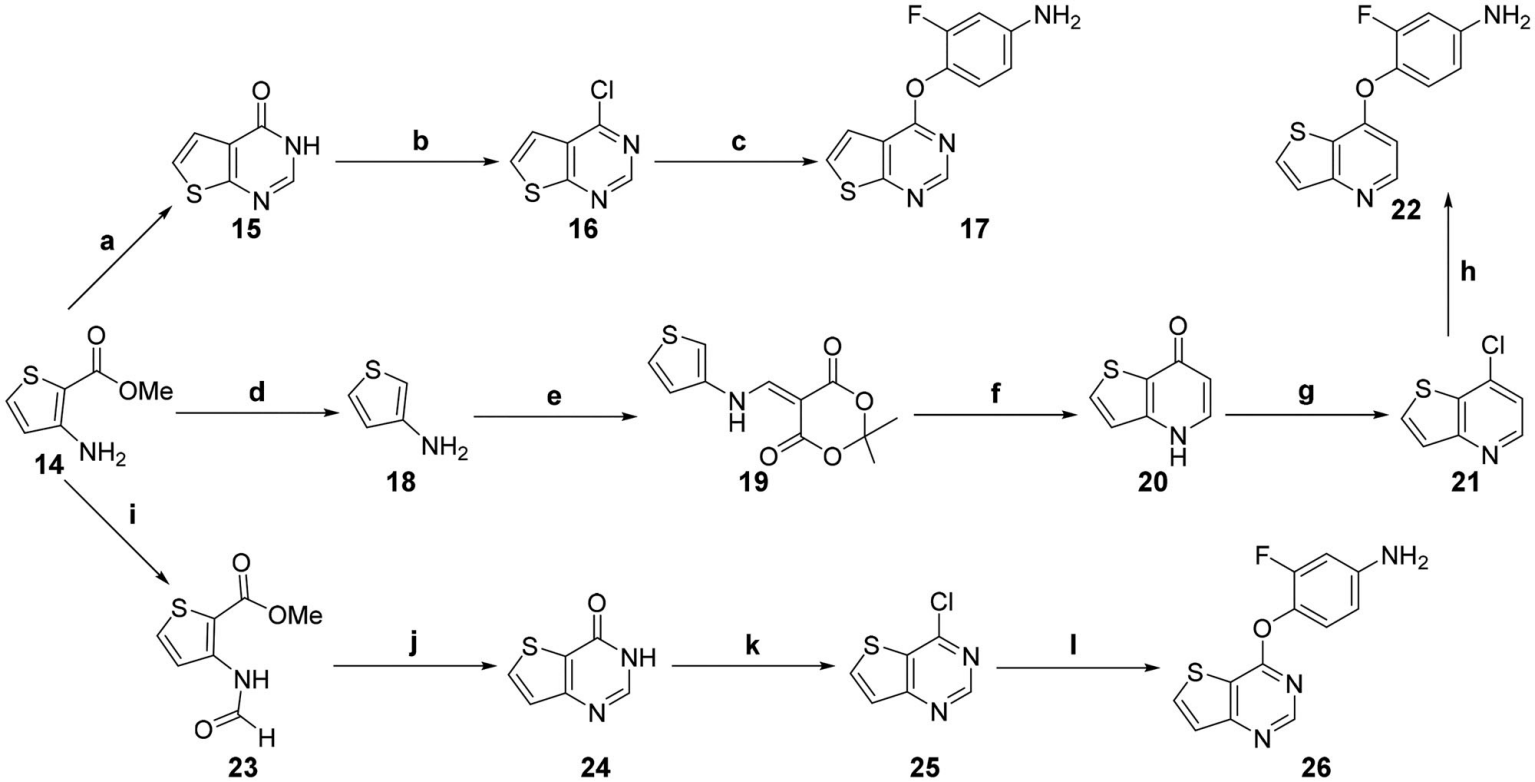
Synthesis of the intermediates **17**, **22**, and **26**; reagents and conditions: (a) formamide, 170 °C, 10 h; (b) POCl_3_, DMF (cat.), toluene, reflux, 6 h; (c) 4-amino-2-fluorophenol, NaH, DMF, 0 °C, 1.5 h; (d) (1) aq. NaOH, reflux, 30 min; (2) oxalic acid, 1-propanol, 38 °C, 45 min; (e) triethyl orthoformate, 2,2-dimethyl-1,3-dioxane-4,6-dione, 85 °C, overnight; (f) Ph_2_O, 240 °C, 30 min; (g) POCl_3_, DMF (cat.), 0 °C → reflux, 2 h; (h) (1) 2-fluoro-4-nitrophenol, K_2_CO_3_, Ph_2_O, 160 °C, 6 h; (2) SnCl_2_, MeOH, 70 °C, 6 h; (i) HCOOH, Ac_2_O, 0 °C → rt, 12 h; (j) HCONH_2_, 150 °C, 8 h; (k) oxalyl chloride, DMF (cat.), CH_2_Cl_2_, 0 °C → reflux, 3 h; (l) 4-amino-2-fluorophenol, NaH, DMF, 80 °C, 2 h.

The synthesis of intermediates **17**, **22** and **26** is depicted in Scheme 2 using 3-amino-2-thiophene carboxylic acid methyl ester **14** as starting material.^30^^,^^49^^,^^50^ Cyclisation of **14** with formamide yielded ketone **15**, which was converted into **17** by employing a two-step strategy through chlorination and nucleophilic substitution under basic conditions. Material **14** was converted into **18** via hydrolysation followed by decarboxylation. Condensation of **18** with 2,2-dimethyl-1,3-dioxane-4,6-dione in the presence of triethyl orthoformate produced imine **19**, which was used directly. Heating of **19** in Ph_2_O generated ketone **20**, which was chlorinated with POCl_3_ to afford chloride **21**. Treatment of **21** through nucleophilic substitution and reduction yielded compound **22**. On the other hand, material **14** underwent an efficient sequence of formylation, cyclisation, chlorination, and condensation to provide **26** in good overall yield.

**Scheme 3.**
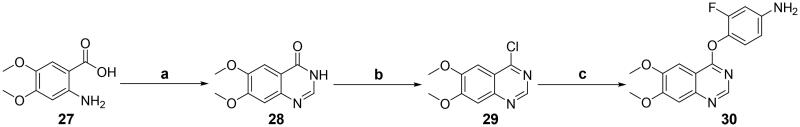
Synthesis of the intermediate **30**; reagents and conditions: (a) formamide, 150 °C, 8 h; (b) POCl_3_, 0 °C → reflux, 6 h; (c) 4-amino-2-fluorophenol, NaH, DMSO, 0 °C → 60 °C, overnight.

The intermediate **30** was prepared according to the synthetic route described in Scheme 3.^30^^,^^51^ The starting material 2-amino-4,5-dimethoxybenzoic acid **27** underwent a sequence of cyclisation and chlorination under basic conditions to afford 4-chloroquinazoline **29** in good overall yield. Treatment of **29** with 4-amino-2-fluorophenol in the presence of NaH gave the desired compound.

**Scheme 4.**
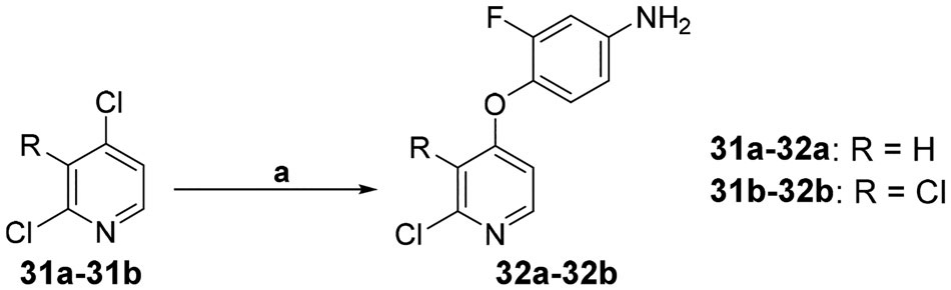
Synthesis of the intermediate **32**; reagents and conditions: (a) R = H, 4-amino-2-fluorophenol, NaH, DMF, 0 °C → 80 °C, overnight; R = Cl, 4-amino-2-fluorophenol, NaH, DMF, 0 °C → 100 °C, 3 h.

The synthesis of **32a**–**32b** was similar to those described for **17**, **26**, and **30**. For access to 4-phenoxy substituted pyridine **32**, 4-chloropyridines **31a**–**31b** were subjected to SnAr conditions in the presence of 4-amino-2-fluorophenol, which displaced the halogen at the 4-position of pyridine (Scheme 4).^31^^,^^52^

**Scheme 5.**
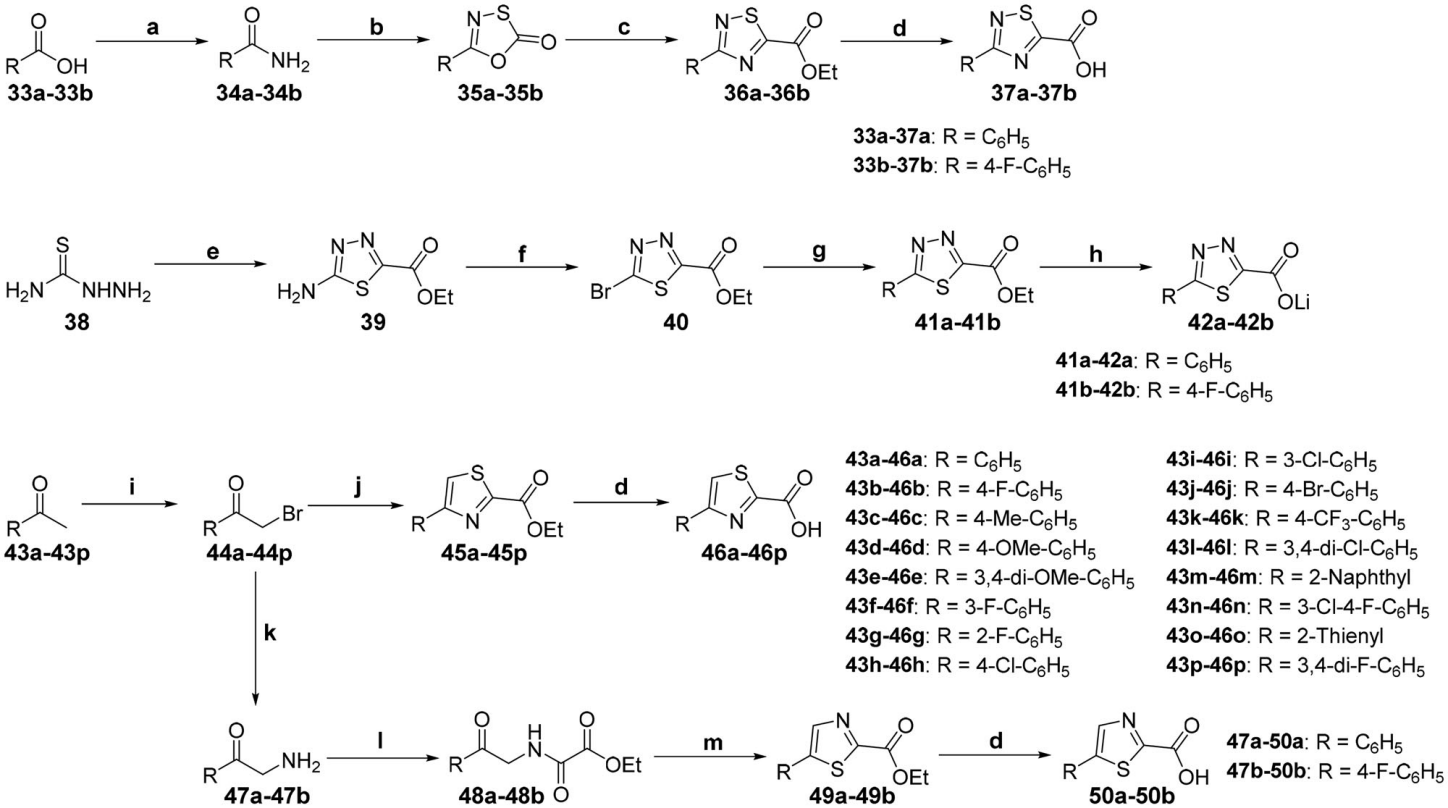
Synthesis of the intermediates **37**, **42**, **46**, and **50**; reagents and conditions: (a) (1) SOCl_2_, 80 °C, 4 h; (2) NH_4_OH, THF, 0 °C → rt, 30 min; (b) (chlorothio)formyl chloride, toluene, 100 °C, 3 h; (c) ethyl cyanoformate, *n*-dodecane, 160 °C, 16 h; (d) aq. LiOH, MeOH, rt, 4 h; (e) ethyl oxalyl monochloride, POCl_3_, 70 °C, 6 h; (f) *t*-BuONO, CuBr_2_, CH_3_CN, 60 °C, 30 min; (g) Pd(OAc)_2_, Xantphos, NMM, toluene, H_2_O, rt, 7 h; (h) aq. LiOH, MeOH, 0 °C, 1 h; (i) NBS, *p*-TsOH, CH_3_CN, 50 °C, 24 h; (j) ethyl thiooxamate, EtOH, reflux, 6 h; (k) (1) urotropin, CHCl_3_, 50 °C, 2 h; (2) conc. HCl, EtOH, reflux, 2 h; (l) ethyl oxalyl monochloride, TEA, CH_2_Cl_2_, 0 °C → rt, overnight; (m) P_2_S_5_, CHCl_3_, reflux, 5 h.

The intermediates **37**, **42**, **46**, and **50** were synthesised according to the synthetic sequence outlined in Scheme 5. Acid **33** underwent acylation and animation to afford amide **34**. Condensation of **34** with (chlorothio)formyl chloride in toluene at 100 °C yielded 1,3,4-oxathiazol-2-one **35,**^53^ which was treated with ethyl cyanoformate (ECF) in *n*-dodecane to generate 1,2,4-thiadiazole-5-carboxylic acid ethyl ester **36.**^54^ 1,2,4-Thiadiazole-5-carboxylic acid **37** was obtained smoothly via hydrolysation of **36** in the mixture of aq. LiOH and MeOH at room temperature.^55^ Cyclisation of thiosemicarbazide **38** with ethyl oxalyl monochloride on exposure of POCl_3_ produced 5-amino-1,3,4-thiadiazol-2-carboxylic acid ethyl ester **39**, which was converted into 5-bromo-1,3,4-thiadiazole-2-carboxylic acid ethyl ester **40** via Sandmeyer bromination in the presence of *t*-BuONO and CuBr_2_.^56^ Suzuki–Miyaura coupling of **40** with corresponding boronic acid was performed using a highly active catalyst system Pd(OAc)_2_/Xantphos in combination with NMM to give **41** in high yield and excellent quality,^56^ which was then hydrolysed in the mixture of aq. LiOH/MeOH at 0 °C for 1 h to afford lithium salt **42**. Treatment of ketone **43** with NBS afforded the α-bromoketone **44**, which was subjected to ethyl thiooxamate to generate thiazole-2-carboxylic acid ethyl ester **45.**^57^ α-Bromoketones **44a***–***44b** were converted to the corresponding α-aminoketones **47a***–***47b** via Delépine reaction in which the α-bromoketone was reacted with urotropin followed by cleavage of the resulting salt with conc. HCl,^58^ treatment of which with ethyl oxalyl monochloride produced **48**. Cyclisation of **48** with phosphorus pentasulfide in CHCl_3_ furnished thiazole-2-carboxylic acid ethyl ester **49.**^59^ The intermediates **46** and **50** were prepared from **45** and **49** respectively using a similar procedure for the preparation of **37**.

**Scheme 6.**
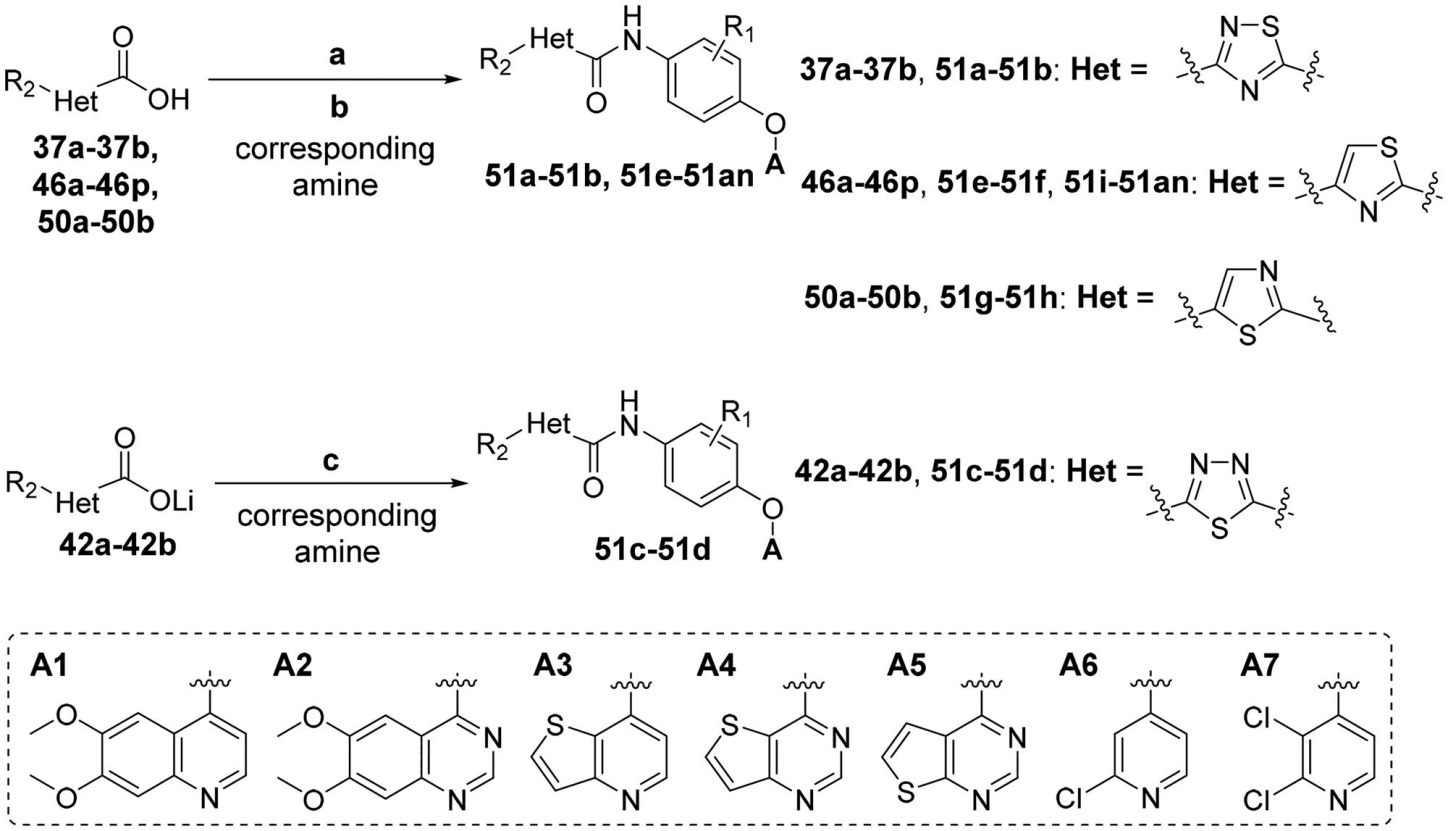
Synthesis of target compounds **51a**–**51an**; reagents and conditions: (a) oxalyl chloride, DMF (cat.), dry CH_2_Cl_2_, 0 °C → rt, 1.5 h; (b) corresponding amine, TEA, dry CH_2_Cl_2_, 0 °C → rt, 4–6 h; (c) corresponding amine, HATU, TEA, DMF, rt, overnight.

The synthesis of target compounds **51a**–**51an** is summarised in Scheme 6. Compounds **51a**–**51b** and **51e**–**51an** were prepared by acylation of various amines (**13**, **17**, **22**, **26**, **30**, and **32**) with corresponding carbonyl chloride, which was generated by the reaction of prepared acid (**37**, **46**, and **50**) with oxalyl chloride catalysed by DMF. On the contrary, given the fact that the 1,3,4-thiadiazole-2-carboxylic acid is unstable in solution because it undergoes a spontaneous decarboxylation process, therefore, 1,3,4-thiadiazole carboxamide scaffold-based derivatives **51c**–**51d** were synthesised by direct condensation of lithium salt **42** with corresponding aniline.^60^ All target compounds were purified by flash column chromatography on silica gel using hexane–ethyl acetate as eluent, and their structures were characterised by NMR, MS, IR and elemental analysis.

### Biology

#### In vitro c-Met enzymatic assay and SAR

Inhibition of target compounds on c-Met kinase activity was measured by using the homogeneous time-resolved fluorescence (HTRF) assay. Foretinib was used as the reference compound, and the screening results (IC_50_ ± SD) as mean values of experiments performed in triplicate are shown in Table 1.

**Table 1.** *In vitro* c-Met inhibitory activities of target compounds **51a**–**51an**.

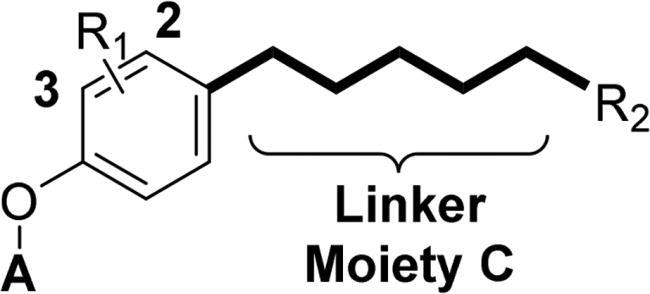					
Compd.	A	R_1_	Linker	R_2_	IC_50_ (nM)^a^
**51a**	**A1**	H	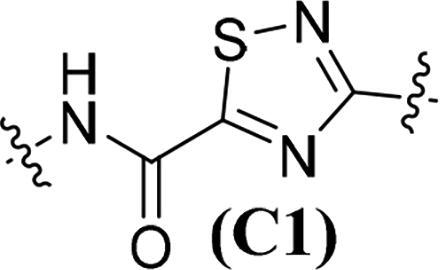	C_6_H_5_	56.64 ± 4.82
**51b**	**A1**	H	**C1**	4-F-C_6_H_5_	50.15 ± 5.14
**51c**	**A1**	H	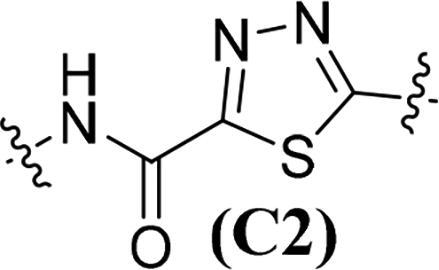	C_6_H_5_	45.67 ± 3.88
**51d**	**A1**	H	**C2**	4-F-C_6_H_5_	41.53 ± 2.76
**51e**	**A1**	H	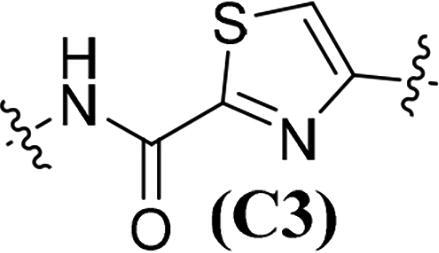	C_6_H_5_	34.48 ± 4.65
**51f**	**A1**	H	**C3**	4-F-C_6_H_5_	29.05 ± 3.53
**51g**	**A1**	H	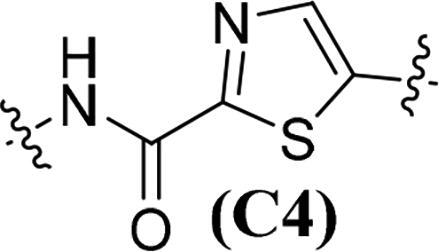	C_6_H_5_	39.36 ± 3.25
**51h**	**A1**	H	**C4**	4-F-C_6_H_5_	35.42 ± 5.03
**51i**	**A1**	2-F	**C3**	C_6_H_5_	31.25 ± 4.16
**51j**	**A1**	3-F	**C3**	C_6_H_5_	29.54 ± 3.42
**51k**	**A1**	2-Cl	**C3**	C_6_H_5_	33.05 ± 2.66
**51l**	**A1**	3-Cl	**C3**	C_6_H_5_	31.86 ± 4.51
**51m**	**A1**	2-Me	**C3**	C_6_H_5_	39.82 ± 5.47
**51n**	**A1**	3-Me	**C3**	C_6_H_5_	45.58 ± 6.03
**51o**	**A2**	3-F	**C3**	C_6_H_5_	29.64 ± 3.84
**51p**	**A2**	3-F	**C3**	4-F-C_6_H_5_	24.67 ± 1.95
**51q**	**A3**	3-F	**C3**	C_6_H_5_	38.48 ± 4.23
**51r**	**A3**	3-F	**C3**	4-F-C_6_H_5_	35.26 ± 5.19
**51s**	**A4**	3-F	**C3**	C_6_H_5_	43.26 ± 6.25
**51t**	**A4**	3-F	**C3**	4-F-C_6_H_5_	40.42 ± 3.66
**51u**	**A5**	3-F	**C3**	C_6_H_5_	40.36 ± 5.53
**51v**	**A5**	3-F	**C3**	4-F-C_6_H_5_	37.56 ± 2.88
**51w**	**A6**	3-F	**C3**	C_6_H_5_	24.27 ± 1.67
**51x**	**A6**	3-F	**C3**	4-Me-C_6_H_5_	31.54 ± 4.25
**51y**	**A6**	3-F	**C3**	4-OMe-C_6_H_5_	36.68 ± 2.97
**51z**	**A6**	3-F	**C3**	3,4-Di-OMe-C_6_H_5_	45.51 ± 6.28
**51aa**	**A6**	3-F	**C3**	4-F-C_6_H_5_	15.62 ± 2.43
**51ab**	**A6**	3-F	**C3**	3-F-C_6_H_5_	23.24 ± 2.16
**51ac**	**A6**	3-F	**C3**	2-F-C_6_H_5_	18.49 ± 3.05
**51ad**	**A6**	3-F	**C3**	4-Cl-C_6_H_5_	17.78 ± 1.82
**51ae**	**A6**	3-F	**C3**	3-Cl-C_6_H_5_	23.42 ± 3.61
**51af**	**A6**	3-F	**C3**	4-Br-C_6_H_5_	19.23 ± 1.44
**51ag**	**A6**	3-F	**C3**	4-CF_3_-C_6_H_5_	39.41 ± 5.25
**51ah**	**A6**	3-F	**C3**	3,4-Di-Cl-C_6_H_5_	9.26 ± 1.34
**51ai**	**A6**	3-F	**C3**	2-Naphthyl	61.36 ± 4.58
**51aj**	**A6**	3-F	**C3**	2-Thienyl	49.55 ± 5.36
**51ak**	**A6**	3-F	**C3**	3-Cl-4-F-C_6_H_5_	3.89 ± 0.52
**51al**	**A6**	3-F	**C3**	3,4-Di-F-C_6_H_5_	5.23 ± 0.74
**51am**	**A7**	3-F	**C3**	3-Cl-4-F-C_6_H_5_	2.54 ± 0.49
**51an**	**A7**	3-F	**C3**	3,4-Di-F-C_6_H_5_	3.73 ± 0.62
**Foretinib** ^b^					1.96 ± 0.23

^a^ IC_50_ (50% inhibitory concentration) values are expressed as mean ± standard deviation (SD). Each assay was performed in triplicate with duplicated samples.

^b^ Positive control.

The results in Table 1 suggested that most of the target compounds displayed varying degrees of potency against c-Met, with IC_50_ values ranging from 2.54 to 61.36 nM. It is worth to note that five of them (**51ah**, IC_50_ = 9.26 nM; **51ak**, IC_50_ = 3.89 nM; **51al**, IC_50_ = 5.23 nM; **51am**, IC_50_ = 2.54 nM; **51an**, IC_50_ = 3.73 nM) exhibited comparable potency against c-Met relative to foretinib, indicating that introduction of thiazole-2-carboxamide (**C3**) as the 5-atom linker could obtain potent c-Met inhibitory activity. Using the type II kinase inhibitor binding element hybrid design approach, we started to explore the structure–activity relationships (SARs) by varying the moieties **A**, **B**, **C** and **D**. Considering the critical role of 5-atom linker on activity, the SAR study of moiety C was initially performed. Meanwhile, 6,7-dimethoxyquinoline core from the launched c-Met inhibitor cabozantinib was introduced into the moiety **A** to synergistically generate better inhibitory activity. Compounds **51a**–**51b** (**51a**, IC_50_ = 56.64 nM, R_1_ = H, R_2_ = C_6_H_5_, **A1**, **C1**; **51b**, IC_50_ = 50.15 nM, R_1_ = H, R_2_ = 4-F-C_6_H_5_, **A1**, **C1**) and **51c**–**51d** (**51c**, IC_50_ = 45.67 nM, R_1_ = H, R_2_ = C_6_H_5_, **A1**, **C2**; **51d**, IC_50_ = 41.53 nM, R_1_ = H, R_2_ = 4-F-C_6_H_5_, **A1**, **C2**) only displayed moderate inhibitory activity against c-Met compared to foretinib. Based on these results, replacement of the thiadiazole carboxamides (**C1** and **C2**) with thiazole carboxamides (**C3** and **C4**) was performed to extend our investigation for the discovery of c-Met inhibitors. To our delight, compounds **51e**–**51h** (**51e**, IC_50_ = 34.48 nM, R_1_ = H, R_2_ = C_6_H_5_, **A1**, **C3**; **51f**, IC_50_ = 29.05 nM; R_1_ = H, R_2_ = 4-F-C_6_H_5_, **A1**, **C3**; **51g**, IC_50_ = 39.36 nM, R_1_ = H, R_2_ = C_6_H_5_, **A1**, **C4**; **51h**, IC_50_ = 35.42 nM; R_1_ = H, R_2_ = 4-F-C_6_H_5_, **A1**, **C4**) exhibited greater potency than compounds **51a**–**51d**, suggesting that the thiazole carboxamide moiety was more suitable for H-binding interactions with c-Met than that of thiadiazole carboxamide. Notably, **C3**-based derivatives exhibited higher potency than that of **C4** derived analogues (**51e**, IC_50_ = 34.48 nM vs. **51g**, IC_50_ = 39.36 nM; **51f**, IC_50_ = 29.05 nM vs. **51h**, IC_50_ = 35.42 nM) when other moieties of compounds were fixed, revealing that the relative position of heteroatom in thiazole ring had a non-negligible influence on c-Met. Therefore, **C3**-based derivatives were further studied. Investigation of the effect of the substituents and its positions (R_1_) on the middle benzene ring was performed. Addition of one halogen atom (such as F and Cl) on middle benzene ring (**51i**, IC_50_ = 31.25 nM, R_1_ = 2-F, R_2_ = C_6_H_5_, **A1**, **C3**; **51j**, IC_50_ = 29.54 nM, R_1_ = 3-F, R_2_ = C_6_H_5_, **A1**, **C3**; **51k**, IC_50_ = 33.05 nM, R_1_ = 2-Cl, R_2_ = C_6_H_5_, **A1**, **C3**; **51l**, IC_50_ = 31.86 nM, R_1_ = 3-Cl, R_2_ = C_6_H_5_, **A1**, **C3**) increased activity relative to **51e** (**51e**, IC_50_ = 34.48 nM), especially for compound **51j** in which one F atom was located at the 3-position of benzene ring. In contrast, changing the halogen atom to a methyl group (**51m**, IC_50_ = 39.82 nM, R_1_ = 2-Me, R_2_ = C_6_H_5_, **A1**, **C3**; **51n**, IC_50_ = 45.58 nM, R_1_ = 3-F, R_2_ = C_6_H_5_, **A1**, **C3**) led to a decrease of activity.

With the promising moiety C (**C3**) and suitable moiety B (3-F-Phenyl) in hand, the screening of moiety **A** was subsequently conducted. Replacing the 6,7-dimethoxyquinoline **A1** to 6,7-dimethoxyquinazoline **A2** displayed comparable inhibitory activity against c-Met (**51o**, IC_50_ = 29.64 nM, R_1_ = 3-F, R_2_ = C_6_H_5_, **A2**, **C3** vs. **51j**, IC_50_ = 29.54 nM, R_1_ = 3-F, R_2_ = C_6_H_5_, **A1**, **C3**). However, replacement of the 6,7-dimethoxyquinoline **A2** with thieno[3,2-*b*]pyridine **A3** (**51q**, IC_50_ = 38.48 nM, R_1_ = 3-F, R_2_ = C_6_H_5_, **C3**; **51r**, IC_50_ = 35.26 nM, R_1_ = 3-F, R_2_ = 4-F-C_6_H_5_, **C3**), thieno[3,2-*d*]pyrimidine **A4** (**51s**, IC_50_ = 43.26 nM, R_1_ = 3-F, R_2_ = C_6_H_5_, **C3**; **51t**, IC_50_ = 40.42 nM, R_1_ = 3-F, R_2_ = 4-F-C_6_H_5_, **C3**) and thieno[2,3-*d*]pyrimidine **A5** (**51u**, IC_50_ = 40.36 nM, R_1_ = 3-F, R_2_ = C_6_H_5_, **C3**; **51v**, IC_50_ = 37.56 nM, R_1_ = 3-F, R_2_ = 4-F-C_6_H_5_, **C3**) resulted in a slight loss of potency in comparison with **51o**–**51p**, suggesting that sulphur-containing fused rings were not suitable for moiety A when **C3** was adopted as the 5-atom linker. To our delight, switching the 6,7-dimethoxyquinoline **A2** to 2-chloropyridine **A6** (**51w**, IC_50_ = 24.27 nM, R_1_ = 3-F, R_2_ = C_6_H_5_, **C3**; **51aa**, IC_50_ = 15.62 nM, R_1_ = 3-F, R_2_ = 4-F-C_6_H_5_, **C3**) resulted in an increase of the activity against c-Met compared to **51o**–**51p**. Further studies on the inhibitory potency of moiety A are currently in progress in our laboratory.

After three optimisation cycles, the effect of moiety D (R_2_) on c-Met was further extensively studied. Using **51w** (IC_50_ = 24.27 nM, R_1_ = 3-F, R_2_ = C_6_H_5_, **C3**) as a reference compound, incorporation of mono-electron-donating groups (mono-EDGs) showed a decreased potency, such as **51x** (IC_50_ = 31.54 nM, R_1_ = 3-F, R_2_ = 4-Me-C_6_H_5_, **C3**, **A6**), **51y** (IC_50_ = 36.68 nM, R_1_ = 3-F, R_2_ = 4-OMe-C_6_H_5_, **C3**, **A6**) and **51z** (IC_50_ = 45.51 nM, R_1_ = 3-F, R_2_ = 3,4-di-OMe-C_6_H_5_, **C3**, **A6**). Therefore, investigation of EDGs was not conducted. In contrast, introducing mono-electron-withdrawing groups (such as F, Cl, and Br) led to improved activity. It should be noted that the activity was correlated with the position of substituents on benzene ring, such as the mono-EWG at the 4-position of benzene ring (**51aa**, IC_50_ = 15.62 nM, R_1_ = 3-F, R_2_ = 4-F-C_6_H_5_, **C3**, **A6**) displayed higher inhibitory activity than that of mono-EWG at the 3- (**51ab**, IC_50_ = 23.24 nM, R_1_ = 3-F, R_2_ = 3-F-C_6_H_5_, **C3**, **A6**) and 2-position of benzene ring (**51ac**, IC_50_ = 18.49 nM, R_1_ = 3-F, R_2_ = 2-F-C_6_H_5_, **C3**, **A6**), the similar trend was also observed in chlorine substituted compounds **51ad** (IC_50_ = 17.78 nM, R_1_ = 3-F, R_2_ = 4-Cl-C_6_H_5_, **C3**, **A6**) and **51ae** (IC_50_ = 23.42 nM, R_1_ = 3-F, R_2_ = 3-Cl-C_6_H_5_, **C3**, **A6**). Addition of a strong EWG (**51ag**, IC_50_ = 39.41 nM, R_1_ = 3-F, R_2_ = 4-CF_3_-C_6_H_5_, **C3**, **A6**) led to a significant loss in activity in comparison to **51w**. 2-Thiophene analogue **51aj** (IC_50_ = 49.55 nM, R_1_ = 3-F, R_2_ = 2-thienyl, **C3**, **A6**) exhibited 2.1-fold loss of activity compared to **51w**, revealing that the electron-rich five-membered ring was not favourable for c-Met inhibitory activity. The bulky substituent (**51ai**, IC_50_ = 46.87 nM, R_1_ = 3-F, R_2_ = 2-naphthyl, **C3**, **A6**, decreased 2.6-fold) also weakened the potency, indicating the hydrophobic pocket of c-Met was fairly sensitive to the size of terminal tail. Based on the above results, modification of moiety D was further explored by introducing double electron-withdrawing groups, which showed an additional increase on c-Met inhibitory efficacy than that of mono-electron-withdrawing-based derivatives (**51ak**, IC_50_ = 3.89 nM, R_1_ = 3-F, R_2_ = 3-Cl-4-F-C_6_H_5_, **C3**, **A6**; **51al**, IC_50_ = 5.23 nM, R_1_ = 3-F, R_2_ = 3,4-di-F-C_6_H_5_, **C3**, **A6**), which showed that the terminal benzene ring substituted with double-electron withdrawing groups was preferable for c-Met inhibitory potency. Inspired by the promising core of BMS-777607 (**6**), two other derivatives (**51am**, IC_50_ = 2.54 nM, R_1_ = 3-F, R_2_ = 3-Cl-4-F-C_6_H_5_, **C3**, **A7**; **51an**, IC_50_ = 3.73 nM, R_1_ = 3-F, R_2_ = 3,4-di-F-C_6_H_5_, **C3**, **A7**) designed by addition of another chlorine atom at the 3-position of pyridine exhibited positive influence on inhibitory potency. In summary, of these 40 newly synthesised compounds, **51am** was the most active compound with an IC_50_ value of 2.54 nM against c-Met, demonstrating that the favourable moiety A, a suitable linker (moiety C) as well as an appropriate terminal hydrophobic tail were all crucial for the discovery of novel type II c-Met inhibitors.

#### In vitro antiproliferative activity

*MET* amplification has been reported in gastric cancer, one of the solid tumours with the high incidence rate, and is associated with poor prognosis. Lung cancer is also closely related to the overexpression of HGF and/or MET and poor prognosis. Recent studies have showed that the c-Met activation in colon cancer occurs in a ligand-dependent paracrine manner. Moreover, c-Met inhibitor SGX523 effectively inhibited the phosphorylation of c-Met and its downstream protein Akt in MDA-MB-231 cells.^61^ Based on these findings, four cancer cell lines (MKN-45, A549, HT-29, and MDA-MB-231) with confirmed high expression of c-Met ^62^^,^^63^ were selected for *in vitro* cytotoxicity assays to determine the antiproliferative effect of the target compounds and further examine whether it was a specific consequence of the dysregulation of c-Met. Additionally, the selectivity of target compounds towards cancer cells versus human normal cell lines (HUVEC and FHC) was determined concurrently. As shown in Table 2, all target compounds were evaluated for *in vitro* cytotoxicity and selectivity relative to normal cell lines, using foretinib as the positive control. The results showed that all target compounds displayed moderate to significant cytotoxicity against four types of tumour cell lines and demonstrated a certain degree of selectivity for the normal cell lines, HUVEC and FHC. Among them, the most promising compound **51am** showed remarkable antiproliferative activity against A549, HT-29, and MDA-MB-231 cell lines with IC_50_ values of 0.83, 0.68, and 3.94 μM, respectively, and possessed a higher selectivity index (SI, IC_50_ of normal cells/IC_50_ of tumour cells) than foretinib. These positive results indicated that utilising thiazole/thiadiazole carboxamide as the moiety C was favourable for cytotoxicity. In addition, some of the target compounds (**51ak**, **51am**–**51an**) exhibited acceptable cytotoxicity against MDA-MB-231 cells compared with foretinib, indicating that these compounds have potential to overcome drug resistance in some specific tumour types. Moreover, most of the target compounds showed greater cytotoxicity against MKN-45 cells than other three cell lines, indicating that the MKN-45 cells were more sensitive to these compounds.

**Table 2.** *In vitro* antiproliferative activities of compounds **51a**–**51an** against six different human cell lines.

Compd.	IC_50_ (µM)^a^					
	MKN-45^b^	A549^c^	HT-29^d^	MDA-MB-231^e^	HUVEC^f^	FHC^g^
**51a**	9.25 ± 0.86	26.35 ± 1.24	24.45 ± 1.68	>50	>100	>100
**51b**	7.89 ± 1.35	22.48 ± 3.15	26.53 ± 1.42	38.65 ± 4.07	>100	>100
**51c**	6.94 ± 1.06	20.56 ± 1.96	29.26 ± 3.85	34.66 ± 2.58	53.45 ± 4.62	71.34 ± 5.25
**51d**	6.65 ± 0.94	18.98 ± 2.46	24.63 ± 1.87	33.56 ± 4.35	52.35 ± 3.85	68.63 ± 4.83
**51e**	5.86 ± 0.93	16.55 ± 2.16	21.75 ± 1.66	>50	56.54 ± 4.81	64.52 ± 6.12
**51f**	4.95 ± 0.82	14.68 ± 3.27	22.36 ± 1.94	31.84 ± 2.56	45.52 ± 3.83	ND^h^
**51g**	6.21 ± 0.85	17.43 ± 2.23	23.42 ± 1.58	32.76 ± 2.23	48.36 ± 2.54	54.47 ± 3.25
**51h**	5.97 ± 0.98	16.84 ± 1.58	22.73 ± 3.32	34.25 ± 2.82	42.14 ± 4.35	50.22 ± 2.89
**51i**	5.35 ± 0.84	15.63 ± 1.86	22.89 ± 2.43	35.53 ± 4.62	44.32 ± 5.64	60.24 ± 5.33
**51j**	4.58 ± 0.75	13.85 ± 1.76	19.56 ± 2.89	32.34 ± 2.78	45.57 ± 5.12	>100
**51k**	6.87 ± 0.86	14.52 ± 1.64	23.36 ± 4.32	33.28 ± 4.21	>100	>100
**51l**	6.23 ± 0.72	13.25 ± 2.41	20.86 ± 3.56	32.11 ± 2.54	40.86 ± 2.32	51.65 ± 3.96
**51m**	6.43 ± 0.76	14.05 ± 1.87	22.34 ± 1.75	35.25 ± 4.23	38.84 ± 3.55	49.62 ± 5.04
**51n**	7.21 ± 0.95	17.48 ± 2.25	24.41 ± 2.14	ND	>100	>100
**51o**	4.23 ± 0.96	12.25 ± 1.63	18.66 ± 3.13	29.58 ± 3.54	36.62 ± 4.23	50.27 ± 3.58
**51p**	3.17 ± 0.84	9.83 ± 1.22	15.24 ± 2.26	28.13 ± 1.96	33.56 ± 2.68	46.36 ± 6.24
**51q**	5.95 ± 0.63	12.86 ± 1.82	19.36 ± 2.48	32.53 ± 2.68	69.54 ± 2.68	80.63 ± 4.52
**51r**	5.02 ± 0.77	10.54 ± 1.76	18.27 ± 3.62	28.86 ± 3.13	40.58 ± 2.25	51.24 ± 4.61
**51s**	6.05 ± 0.94	18.45 ± 2.33	25.56 ± 1.76	32.78 ± 2.70	43.42 ± 3.65	65.49 ± 5.56
**51t**	5.45 ± 0.65	14.46 ± 2.87	23.12 ± 3.34	36.62 ± 2.83	41.36 ± 2.39	67.44 ± 4.38
**51u**	5.53 ± 1.26	16.82 ± 2.28	26.42 ± 3.22	35.89 ± 2.52	38.84 ± 3.53	48.25 ± 5.04
**51v**	4.23 ± 0.94	12.61 ± 1.83	19.24 ± 2.85	29.11 ± 2.52	54.35 ± 4.16	63.28 ± 3.35
**51w**	2.53 ± 0.62	7.26 ± 1.37	12.48 ± 2.39	26.29 ± 3.09	38.64 ± 3.53	48.25 ± 5.04
**51x**	3.46 ± 0.75	8.65 ± 1.72	14.29 ± 2.13	28.57 ± 1.85	30.76 ± 2.43	43.41 ± 3.68
**51y**	3.22 ± 0.86	9.28 ± 1.85	13.95 ± 2.26	28.49 ± 2.35	32.43 ± 1.98	ND
**51z**	4.05 ± 1.12	11.26 ± 1.96	15.62 ± 2.51	20.52 ± 3.14	26.52 ± 3.84	>100
**51aa**	0.95 ± 0.18	4.83 ± 0.72	4.36 ± 0.38	11.68 ± 1.39	15.32 ± 2.16	24.63 ± 4.23
**51ab**	1.49 ± 0.63	6.02 ± 0.87	6.95 ± 0.82	10.13 ± 1.88	12.74 ± 0.83	20.25 ± 3.62
**51ac**	1.94 ± 0.26	7.54 ± 0.62	9.53 ± 0.75	18.06 ± 2.14	19.45 ± 0.83	31.34 ± 2.63
**51ad**	1.74 ± 0.23	6.75 ± 0.55	8.04 ± 0.93	15.20 ± 1.46	18.52 ± 2.04	26.46 ± 1.28
**51ae**	1.95 ± 0.68	7.08 ± 0.95	9.62 ± 1.56	19.20 ± 2.35	13.05 ± 0.95	24.50 ± 1.84
**51af**	2.06 ± 0.45	8.23 ± 1.42	11.65 ± 1.63	20.54 ± 3.65	11.45 ± 0.83	21.55 ± 2.82
**51ag**	5.84 ± 1.21	12.36 ± 2.85	20.37 ± 3.02	30.58 ± 2.64	43.22 ± 3.53	ND
**51ah**	0.53 ± 0.42	3.17 ± 0.51	2.78 ± 0.49	7.29 ± 1.22	12.36 ± 0.85	20.44 ± 1.86
**51ai**	6.12 ± 1.38	15.33 ± 2.81	21.49 ± 1.58	30.26 ± 2.16	>100	>100
**51aj**	4.55 ± 0.91	11.46 ± 2.17	17.53 ± 2.44	25.87 ± 3.41	52.26 ± 3.74	>100
**51ak**	0.22 ± 0.04	1.81 ± 0.34	1.56 ± 0.32	4.24 ± 0.68	8.52 ± 1.35	13.20 ± 0.84
**51al**	0.35 ± 0.07	2.26 ± 0.68	1.94 ± 0.65	5.82 ± 0.73	6.14 ± 0.75	10.17 ± 1.65
**51am**	0.092 ± 0.006	0.83 ± 0.36	0.68 ± 0.09	3.94 ± 1.32	2.54 ± 0.46	8.63 ± 1.52
**51an**	0.15 ± 0.07	1.32 ± 0.35	0.94 ± 0.27	4.65 ± 0.93	4.83 ± 0.69	9.54 ± 1.26
**Foretinib** ^i^	0.058 ± 0.006	0.45 ± 0.05	0.37 ± 0.05	0.89 ± 0.14	0.68 ± 0.09	4.06 ± 0.75

^a^ Fifty percent inhibitory concentration after 48 h of drug treatment obtained from MTT assay. IC_50_ values are expressed as mean ± standard deviation (SD). Each assay was performed in triplicate with duplicated samples.

^b^ Gastric cancer.

^c^ Lung carcinoma.

^d^ Colon adenocarcinoma.

^e^ Triple-negative breast cancer.

^f^ Human umbilical vein endothelial cells.

^g^ Human normal colorectal mucosa epithelial cells.

^h^ Not determined.

^i^ Positive control.

Preliminary SAR correlations were formulated to facilitate further identification of more efficient c-Met inhibitors. It was observed that compounds **51e**–**51f** containing thiazole-2-carboxamide scaffold (**C3**) conferred a better effect on cytotoxicity than compounds **51a**–**51d** and **51g**–**51h**. Regarding the effect of substituents on the benzene ring (moiety B), the cytotoxicity of compounds **51i**–**51n** followed the rank order of F > Cl > Me in all tested cancer cell lines. Except for methyl derivatives **51m**–**51n**, the results suggested that incorporating a halogen atom at 3-position of benzene ring resulted in better cytotoxicity than that its 2-position (**51j** > **51i**, **51l** > **51k)**. Moreover, the pyridine core was favoured because of the promising cytotoxicity results for **51w**–**51an**. The SAR of moiety D was then examined, and the results demonstrated that weak electron withdrawing groups (**51aa** and **51ac–51ad**) on the benzene ring were favourable for cytotoxic activity. However, electron donating groups (**51x–51z**), strong electron withdrawing group (**51ag**), and bulky group (**51ai**) were detrimental to cytotoxic potency. The substitution positions on the benzene ring also resulted in significant changes in cytotoxicity, with the cytotoxic results of compounds **51aa**–**51ae** showing a clear preference (4-F > 3-F > 2-F, 4-Cl > 3-Cl). Furthermore, the dual substituted electron withdrawing groups on the phenyl ring (**51ah**, **51ak**–**51an**) generally afforded greater inhibitory activity than mono electron withdrawing groups (**51aa**–**51af**). When exploring the heterocycle (2-thienyl, **51aj**) and fused ring (2-naphthyl, **51ai**) as moiety D, we found that both of them were unfavourable for toxicity. On the basis of the *in vitro* results, compound **51am** was selected for further biological evaluation.

#### Induction of apoptosis and cycle arrest

**Figure 3.**
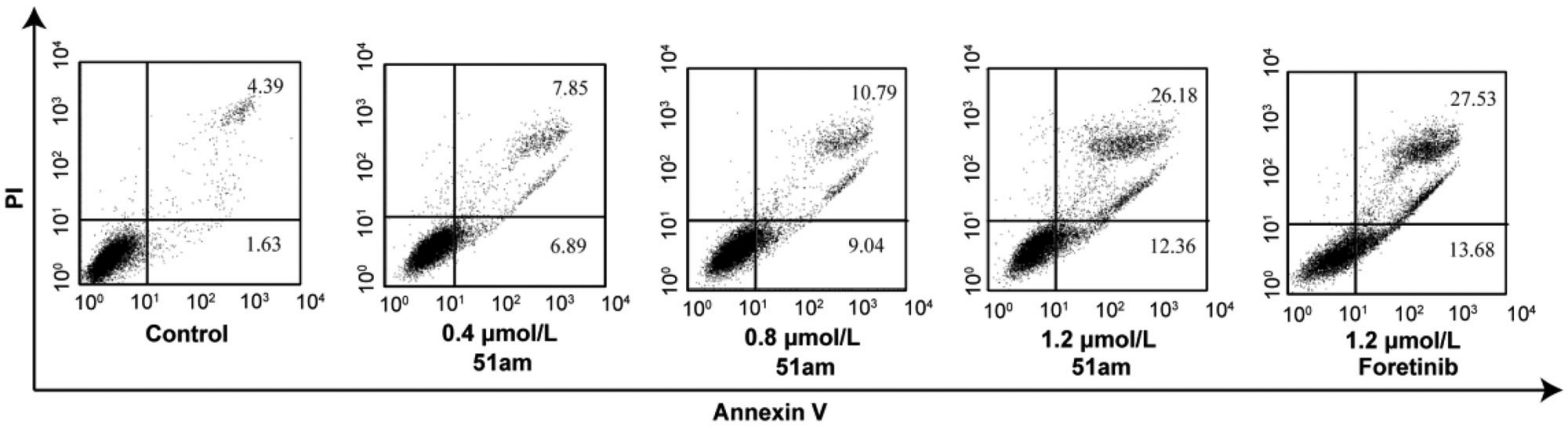
The effect of **51am** and foretinib on MKN-45 cells apoptosis by Annexin V/PI double staining.

**Figure 4.**
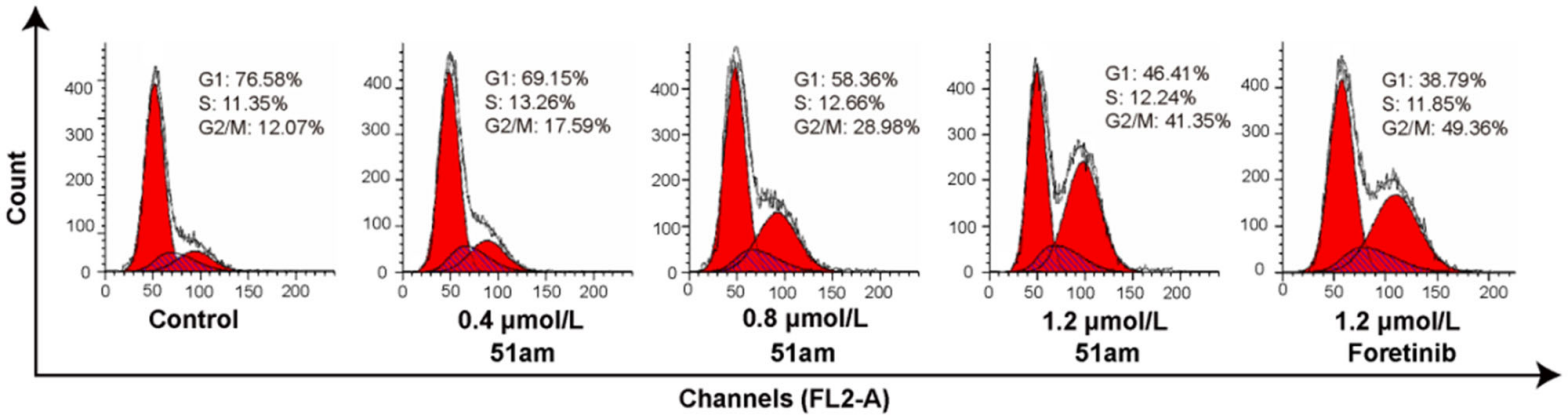
The effect of **51am** and foretinib on MKN-45 cells cycle arrest by PI staining with RNase.

As shown in Table 2, compound **51am** displayed higher sensitivity to MKN-45 cells than other three tested cancer cell lines in the preliminary cytotoxicity profile, therefore, MKN-45 cells were used in the subsequent mechanistic study. Apoptosis is one of the main modes of cell death after drug treatment, which is controlled by the expression of many regulatory factors. To determine the antiproliferative potency of **51am** on human cancer cells, its effect on MKN-45 cells apoptosis and cycle arrest was determined using Annexin V/PI double staining and analysed by flow cytometer. As shown in Figure 3, after MKN-45 cells were treated with different concentrations of compound **51am** (0.4, 0.8, and 1.2 μM) for 24 h, the percentages of apoptotic cells were determined to be 14.74%, 19.83%, and 38.54%, respectively, which were higher than that of the control group (6.02%), suggesting that **51am** induced MKN-45 cell apoptosis in a dose-dependent manner. More importantly, the percentage of apoptotic cells following treatment with **51am** was comparable to that of foretinib (**51am** vs. foretinib, 38.54% vs. 41.21%) at the same concentration (1.2 μM). We then investigated the effect of **51am** on cell cycle distribution of MKN-45 cells. As depicted in Figure 4, the percentage of cells in G2/M phase significantly increased in a dose-dependent manner compared with the control group after treatment with **51am** (0.4, 0.8, and 1.2 μM) for 24 h. Notably, cell cycle distribution of MKN-45 cells after treatment with 1.2 μM of **51am** exhibited a similar trend to that observed for foretinib. Taken together, these results suggested that **51am** could effectively induce cell cycle arrest and apoptosis in a dose-dependent manner, consequently inhibiting the proliferation of cancer cells.

#### Western blotting analysis of c-Met phosphorylation

**Figure 5.**
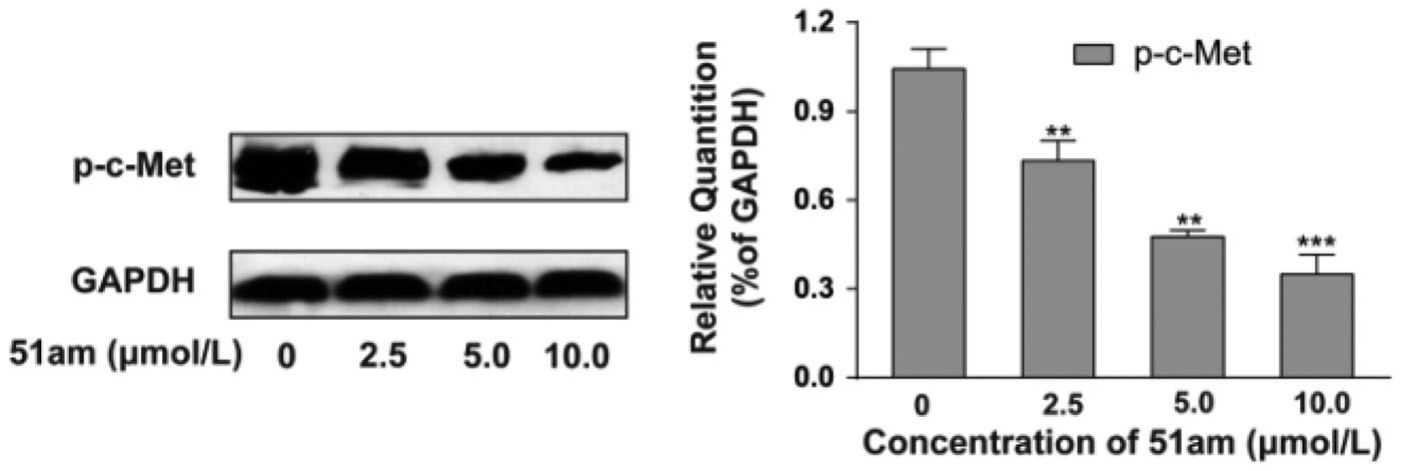
**51am** inhibited c-Met phosphorylation in MKN-45 cells.

To obtain further insight into the mechanism of **51am** induced apoptosis, western blot assay was carried out to investigate the effect of **51am** on the phosphorylation of c-Met in living cells. MKN-45 cells were treated with **51am** for 24 h in a series of concentrations (0, 2.5, 5.0, and 10.0 μM) using DMSO as the negative control, and the level of GAPDH served as the loading control. As depicted in Figure 5, compound **51am** inhibited c-Met phosphorylation in a dose-dependent manner, which was consistent with the observed *in vitro* activity. This suggested that the antiproliferative activity of **51am** may be partially due to the inhibition of c-Met kinase.

#### In vitro kinase profile of compound 51am

The emerging oncogenic mutations of c-Met that confer resistance to small molecule c-Met inhibitors have become critical issues to be solved urgently. Consequently, **51am** was tested for anti-drug resistance against a spectrum of mutations. As shown in Table 3, **51am** maintained potency against mutations H1094R, D1228H, Y1230H, Y1235D, and M1250T, with IC_50_ values of 93.6, 29.4, 45.8, 54.2, and 26.5 nM, respectively. On the other hand, comprehensive understanding the off-target kinase inhibition is critical for any kinase inhibitors, particularly to help explain the correlations between efficacy and potential side effects. Given the remarkable potency of **51am** against wild type c-Met and its mutants, we moved forward to investigate the kinase selectivity profile of **51am** against other protein kinases. In addition to high potency against c-Met, it displayed good inhibitory effects against c-Kit (IC_50_ = 4.94 nM), Flt-3 (IC_50_ = 6.12 nM), and Ron (IC_50_ = 3.83 nM). In contrast to its high potency against c-Met, **51am** afforded 108–207-fold less potent against PDGFRα, PDGFRβ, VEGFR-2, and Flt-4. It also showed negligible inhibitory activity against EGFR with IC_50_ > 10 μM. Notably, in terms of selectivity between c-Met and VEGFR-2 for candidate compounds, **51am** (c-Met, IC_50_: 2.54 nM; VEGFR-2, IC_50_: 527 nM) showed greater selectivity than foretinib (c-Met, IC_50_: 1.96 nM; VEGFR-2, IC_50_: 4.58 nM), suggesting that the **C3** scaffold-based type II c-Met inhibitors could confer selectivity against c-Met, overcome acquired drug resistances, and possibly reduce VEGFR-2 related side effects.

**Table 3.** c-Met mutant and kinase selectivity profile of **51am**.

Enzyme	IC_50_ (nM)	Enzyme	IC_50_ (nM)
c-Met *H1094R*	93.6	Ron	3.83
c-Met *D1228H*	29.4	PDGFRα	425
c-Met *Y1230H*	45.8	PDGFRβ	513
c-Met *Y1235D*	54.2	VEGFR-2	527
c-Met *M1250T*	26.5	EGFR	>10 000
Wild type c-Met	2.54	Flt-3	6.12
c-kit	4.94	Flt-4	276

#### Pharmacokinetic profile of compound 51am

Identification of drugs with an acceptable balance of potency, physical properties, and pharmacokinetics (PK) has always been a challenge that often hinders evaluation of the compounds in xenograft models and therapeutic use in clinical practice. Therefore, the PK profile of compound **51am** was evaluated in BALB/c mice after intravenous (i.v.) injection and oral administration (p.o.) because of its excellent *in vitro* activity. As shown in Table 4, after p.o. (10 mg/kg), **51am** showed a promising overall PK profile, including rapid absorption (*T*_max_ = 4.1 h), high maximum concentration (*C*_max_ = 1756 ng/mL), good plasma exposure (AUC_0–∞_ = 11.5 µg.h/mL), acceptable elimination half-life (*T*_1/2_ = 5.6 h), and favourable clearance (*CL* = 0.87 L/h.kg). When administered via the i.v. route (1.5 mg/kg), **51am** was characterised by slightly better maximum concentration (*C*_max_ = 552 ng/mL) and plasma exposure (AUC_0–∞_ = 2.5 µg.h/mL), and a good half-life (*T*_1/2_ = 3.2 h). Moreover, **51am** exhibited good oral bioavailability (*F* = 69%) in BALB/c mice. Based on the good overall PK profile, **51am** could be a potential candidate for cancer therapy.

**Table 4.** Pharmacokinetic profile of **51am** in BALB/c mice^a^.

Compd.	51am	
Route	i.v. (1.5 mg/kg)	p.o. (10 mg/kg)
*T*_1/2_ (h)	3.2	5.6
*C*_max_ (ng/mL)	552	1756
*T*_max_ (h)	–	4.1
AUC_0–∞_ (µg.h/mL)	2.5	11.5
*CL* (L/h.kg)^b^	0.6	–
*F*/%^c^	–	69

^a^ Data reported as the average of six animals.

^b^ *CL* = dose/AUC_i.v._.

^c^ *F* = (AUC_p.o._/AUC_i.v._) × (Dose_i.v._/Dose_p.o._) × 100%.

### Binding mode analysis

**Figure 6.**
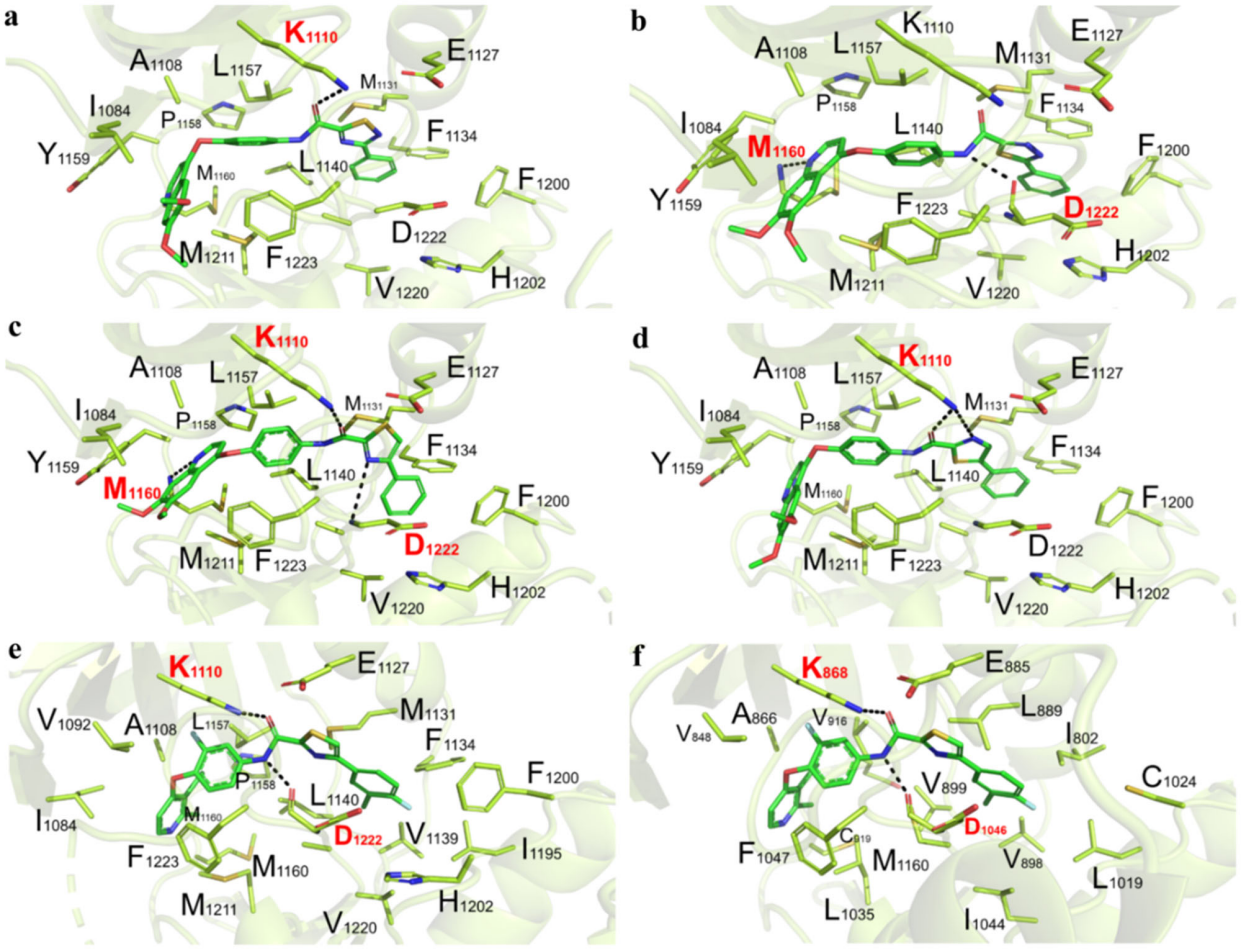
(a) The proposed binding mode of compound **51a** (C1 scaffold as moiety C) with the active site of c-Met. Compound was shown in coloured sticks, green: carbon atom, blue: nitrogen atom, pink: oxygen atom, yellow: sulphur atom; (b) the proposed binding mode of compound **51c** (C2 scaffold as moiety C) with the active site of c-Met; (c) the proposed binding mode of compound **51e** (C3 scaffold as moiety C) with the active site of c-Met; (d) the proposed binding mode of compound **51g** (C4 scaffold as moiety C) with the active site of c-Met; (e) the proposed binding mode of compound **51am** with the active site of c-Met; (f) the proposed binding mode of compound **51am** with the active site of VEGFR-2.

To further explore the binding modes of target compounds to the specific kinases, molecular docking simulation studies were performed concurrently. Amino acid sequences for c-Met and VEGFR-2 were retrieved from the Uniprot database with accessible numbers P08581 and P35968, respectively. 3D crystal structures for c-Met (PDB ID: 3zcl) and VEGFR-2 (PDB ID: 1ywn) with the lowest resolutions at 1.4 Å and 1.7 Å, respectively, were chosen for docking and molecular dynamics simulations. Given the fact that **C3**-based derivatives generally exhibited favourable potency in both biochemical and cellular assays *in vitro* than other three types of moiety C, molecular docking study was further performed to explore the binding details of block C. Compounds **51a**, **51c**, **51e**, and **51g** were selected as template molecules, in which the moieties A, B, and D were fixed only the moiety C was varied. As shown in Figure 6(a), compound **51a** bearing the **C1** moiety only formed one hydrogen bond between the oxygen atom of carbonyl (moiety C) and residue Lys1110 of c-Met (estimated binding energy: –9.46 kcal/mol), which could be attributed to the intramolecular repulsion by the conformational change induced by 1,2,4-thiadiazole moiety. For compound **51c**, the delicate structural difference between **51a** and **51c** gave rise to the movement of **51c** towards the left in the active site of c-Met (Figure 6(b)), resulting in formation of another hydrogen bond between the nitrogen atom of quinoline core and residue Met1160 in addition to the hydrogen bond formed by the H atom of NH (moiety C) and Asp1222 (estimated binding energy: –10.25 kcal/mol). As shown in Figure 6(c), we clearly observed that there were three hydrogen bonds between **51e** and c-Met kinase, the nitrogen atom of quinoline core and residue Met1160, the oxygen atom of carbonyl (moiety C) and residue Lys1110, and the nitrogen atom of thiazole and residue Asp1222, most likely due to the elimination of intramolecular repulsion when changing the thiadiazole ring to a thiazole ring and the newly formed favourable hydrophobic interactions (estimated binding energy: –11.85 kcal/mol). Conversely, when exchanging the positions of the S and N atoms, the nitrogen atom of thiazole of compound **51g** formed one hydrogen bond with Lys1110 instead of Asp1222 (Figure 6(d)) (estimated binding energy: –13.06 kcal/mol). Subsequently, the binding modes of **51am** with c-Met or VEGFR-2 were analysed as shown in Figure 6(e,f), since VEGFR-2 associated side-effects have been a vital concern in designing and discovering novel antitumor candidates. As shown in Figure 6(e), **51am** generated three hydrogen bonds with c-Met and also formed excellent hydrophobic interactions with hydrophobic amino acid residues; whereas, only two hydrogen bonds were produced between **51am** and VEGFR-2. The hydrophobic interactions of **51am**/VEGFR-2 complex decreased significantly because the hydrophobic amino acids in the hydrophobic cavity of c-Met were replaced with amino acids with shorter side chains for VEGFR-2 (Figure 6(f)). In summary, these docking results could provide a basis for the rational design of novel c-Met inhibitors with high selectivity.

## Conclusions

By combining the type II c-Met inhibitors binding element hybrid design approach with the pharmacophore merging strategy, thiazole/thiadiazole carboxamide scaffold-based derivatives were designed, synthesised, and evaluated for their antitumor activity. After five cycles of optimisation, **51am** was found to be as the most potent c-Met inhibitor among the synthesised target compounds in both enzyme- and cell-based assays. Further biological evaluation demonstrated that **51am** not only induced cell cycle arrest and cell apoptosis but also inhibited c-Met activity in cell and cell-free systems. **51am** simultaneously possessed acceptable inhibitory efficacy against a spectrum of c-Met mutations and moderate selectivity for wild type c-Met compared with other test kinases. More importantly, **51am** achieved a better balance between cell-based activity and the favourable pharmacokinetic profile. In addition, the binding modes of **51am** to both c-Met and VEGFR-2 offered insights for designing novel c-Met inhibitors with high selectivity. Taken together, these positive results suggested that **51am** could be a potential c-Met inhibitor deserving further development.

## Experimental

### Chemistry

Common chemicals (reagents and solvents) used in this paper were purchased from Meryer (Shanghai) Chemical Technology Co., Ltd. (Shanghai, China). All reactions were monitored by thin layer chromatography (TLC) on silica gel 60 GF254 plates (Shanxi Nuotai Biotechnology Co., Ltd, Yuncheng, China) and the spots were visualised using UV light (254 nm). Flash column chromatography was performed using silica gel (200–300 mesh, Qingdao, China) with a linear solvent gradient. Mass spectra were recorded on a Bruker Daltonics APEXII49e spectrometer (Billerica, MA). ^1^H and ^13^C NMR spectra were measured on a Bruker Avance III spectrometer (Billerica, MA) and referenced to TMS. Chemical shifts are recorded as *δ* in units of parts per million (ppm) and coupling constants are given in Hz. Peak multiplicities are defined as s (singlet), d (doublet), t (triplet), q (quartet), m (multiplet), and br (broad). Melting points were measured by using a Gongyi X-5 microscopy digital melting point apparatus and are uncorrected. IR spectra were obtained on an NIC-5DX spectrophotometer. The purity of all tested compounds was determined by HPLC (Agilent Technologies 1100 series, Santa Clara, CA) equipped with a C-18 bound-phase column (Eclipse Plus C18, 5 μM particle size, 4.6 mm × 250 mm). A gradient elution was performed with MeOH and water as a mobile phase and was monitored at 254 nm. All tested target compounds were >95% pure.

#### General procedure for the preparation of 4-((6,7-dimethoxyquinolin-4-yl)oxy)anilines (13a–13g)

The key intermediates **13a**–**13g** were prepared according to the methods of our previous works (Supporting Information),^22^^,^^29^^,^^30^ therefore, only characterisation data for the title compounds are presented here.

##### 4-((6,7-Dimethoxyquinolin-4-yl)oxy)aniline (13a).^22^^,^^29^

Light yellow solid; m.p. 212–214 °C. IR (KBr) *ν*_max_/cm^–^^1^ 3375, 3152, 1582, 1509, 1478, 1431, 1344, 1248, 1216, 994, 898, 849, 818. ^1^H NMR (400 MHz, DMSO-*d*_6_) *δ* 8.42 (d, *J* = 4.0 Hz, 1H), 7.50 (s, 1H), 7.36 (s, 1H), 6.92 (d, *J* = 8.0 Hz, 2H), 6.66 (d, *J* = 8.0 Hz, 2H), 6.36 (d, *J* = 4.0 Hz, 1H), 5.17 (br s, 2H), 3.93 (s, 6H).

##### 4-((6,7-Dimethoxyquinolin-4-yl)oxy)-2-methylaniline (13b)

Greyish yellow solid; m.p. 206–208 °C. IR (KBr) *ν*_max_/cm^–^^1^ 3402, 3211, 1650, 1579, 1505, 1479, 1461, 1431, 1344, 1299, 1247, 1228, 1208, 1167, 1001, 948, 892, 852, 817. ^1^H NMR (400 MHz, DMSO-*d*_6_) *δ* 8.41 (d, *J* = 5.2 Hz, 1H), 7.49 (s, 1H), 7.35 (s, 1H), 6.85 (s, 1H), 6.81 (dd, *J* = 2.4, 8.4 Hz, 1H), 6.70 (d, *J* = 8.4 Hz, 1H), 6.36 (d, *J* = 5.2 Hz, 1H), 4.90 (br s, 2H), 3.92 (s, 6H), 2.08 (s, 3H).

##### 4-((6,7-Dimethoxyquinolin-4-yl)oxy)-3-methylaniline (13c)

Yellow solid; m.p. 209–211 °C. IR (KBr) *ν*_max_/cm^–^^1^ 3331, 3189, 1623, 1578, 1507, 1481, 1432, 1349, 1300, 1272, 1254, 1211, 1167, 993, 892, 850, 824. ^1^H NMR (400 MHz, DMSO-*d*_6_) *δ* 8.40 (d, *J* = 5.2 Hz, 1H), 7.54 (s, 1H), 7.36 (s, 1H), 6.84 (d, *J* = 8.4 Hz, 1H), 6.55 (s, 1H), 6.50 (dd, *J* = 2.4, 8.4 Hz, 1H), 6.25 (d, *J* = 5.2 Hz, 1H), 5.07 (br s, 2H), 3.93 (s, 6H), 1.94 (s, 3H).

##### 2-Chloro-4-((6,7-dimethoxyquinolin-4-yl)oxy)aniline (13d)

Orange solid; m.p. 234–236 °C. IR (KBr) *ν*_max_/cm^–^^1^ 3405, 3191, 1579, 1505, 1478, 1459, 1432, 1344, 1300, 1247, 1215, 1164, 994, 913, 849, 816. ^1^H NMR (400 MHz, DMSO-*d*_6_) *δ* 8.44 (d, *J* = 5.2 Hz, 1H), 7.48 (s, 1H), 7.36 (s, 1H), 7.19 (d, *J* = 2.4 Hz, 1H), 6.99 (dd, *J* = 2.8, 8.8 Hz, 1H), 6.92–6.90 (m, 1H), 6.41 (d, *J* = 5.2 Hz, 1H), 5.42 (br s, 2H), 3.93 (s, 3H), 3.92 (s, 3H).

##### 3-Chloro-4-((6,7-dimethoxyquinolin-4-yl)oxy)aniline (13e)

Pale yellow solid; m.p. 213–215 °C. IR (KBr) *ν*_max_/cm^–^^1^ 3419, 3343, 1625, 1592, 1580, 1508, 1477, 1427, 1347, 1302, 1255, 1219, 1158, 989, 843, 826. ^1^H NMR (400 MHz, DMSO-*d*_6_) *δ* 8.44 (d, *J* = 5.2 Hz, 1H), 7.51 (s, 1H), 7.38 (s, 1H), 7.09 (d, *J* = 8.8 Hz, 1H), 6.78 (d, *J* = 2.4 Hz, 1H), 6.63 (dd, *J* = 2.4, 8.4 Hz, 1H), 6.28 (d, *J* = 5.2 Hz, 1H), 5.48 (s, 2H), 3.93 (s, 6H).

##### 4-((6,7-Dimethoxyquinolin-4-yl)oxy)-2-fluoroaniline (13f)

Brown solid; m.p. 204–206 °C. IR (KBr) *ν*_max_/cm^–^^1^ 3405, 3168, 1596, 1582, 1505, 1479, 1460, 1431, 1344, 1302, 1247, 1209, 1185, 1168, 996, 955, 849, 817. ^1^H NMR (400 MHz, DMSO-*d*_6_) *δ* 8.44 (d, *J* = 5.2 Hz, 1H), 7.48 (s, 1H), 7.36 (s, 1H), 7.05 (dd, *J* = 2.8, 12.0 Hz, 1H), 6.90–6.82 (m, 2H), 6.42 (d, *J* = 5.2 Hz, 1H), 5.21 (s, 2H), 3.93 (s, 3H), 3.92 (s, 3H).

##### 4-((6,7-Dimethoxyquinolin-4-yl)oxy)-3-fluoroaniline (13g).^22^^,^^30^

Pale yellow solid; m.p. 193–195 °C. IR (KBr) *ν*_max_/cm^–^^1^ 3336, 3149, 1625, 1579, 1510, 1486, 1462, 1431, 1351, 1304, 1257, 1213, 1167, 990, 895, 838, 824. ^1^H NMR (400 MHz, DMSO-*d*_6_) *δ* 8.45 (d, *J* = 5.6 Hz, 1H), 7.51 (s, 1H), 7.38 (s, 1H), 7.09–7.04 (m, 1H), 6.56 (dd, *J* = 2.4, 13.2 Hz, 1H), 6.47 (dd, *J* = 2.0, 8.8 Hz, 1H), 6.39 (d, *J* = 5.2 Hz, 1H), 5.50 (s, 2H), 3.93 (s, 6H).

#### Synthesis of thieno[2,3-d]pyrimidin-4(3H)-one (15)

A mixture of **14** (3.14 g, 20 mmol) and formamide (20 mL) was heated at 170 °C for 10 h. After cooling to room temperature, cooled water (30 mL) was added to the reaction mixture. The solid was removed by filtration, washed with water, and dried under vacuum for 12 h. The crude residue was suspended in ethyl ether, stirred for 30 min and filtered. The resultant grey solid (1.88 g, 62% yield) was used for the next step without further purification. M.p. 261–263 °C. IR (KBr) *ν*_max_/cm^–^^1^ 3071, 1666, 1592, 1578, 1466, 1368, 1287, 1167, 981, 800, 703, 636, 565. ^1^H NMR (400 MHz, DMSO-*d*_6_) *δ* 12.64 (br s, 1H), 8.14 (s, 1H), 7.56 (d, *J* = 5.2 Hz, 1H), 7.35 (d, *J* = 5.2 Hz, 1H).

#### Synthesis of 4-chlorothieno[2,3-d]pyrimidine (16)

A mixture of **15** (1.52 g, 10 mmol) and POCl_3_ (6 mL) with 2–3 drops of DMF was refluxed for 6 h. After the mixture was cooled to room temperature, POCl_3_ was removed under vacuum, the obtained residue was poured into a saturated solution of NaHCO_3_, and the suspension was neutralised with aq. NaOH (6 M). The mixture was extracted with dichloromethane and the organic phase was washed with water and brine, dried over Na_2_SO_4_ and concentrated in vacuum. The crude product was purified by silica gel column chromatography using hexane–ethyl acetate (8:1 to 3:1, v/v) as eluent to yield the title compound (1.21 g, 71% yield) as a beige solid. M.p. 110–112 °C. IR (KBr) *ν*_max_/cm^–^^1^ 3434, 3113, 1552, 1509, 1477, 1417, 1352, 1276, 1246, 1162, 1131, 877, 844, 715. ^1^H NMR (400 MHz, CDCl_3_) *δ* 8.89 (s, 1H), 7.65 (d, *J* = 6.4 Hz, 1H), 7.44 (d, *J* = 6.4 Hz, 1H).

#### Synthesis of 3-fluoro-4-(thieno[2,3-d]pyrimidin-4-yloxy)aniline (17)

To a solution of 4-amino-2-fluorophenol (0.95 g, 7.5 mmol) in dry DMF (15 mL) was added NaH (216 mg, 9 mmol). The resulting mixture was stirred at 0 °C for 10 min and **16** (1.02 g, 6 mmol) was then added in portions. The reaction mixture was stirred at 0 °C for 1.5 h. Cold water (50 mL) was added to quench the reaction and the mixture was extracted by ethyl acetate (3 × 50 mL). The combined organic layers were washed with brine, dried over Na_2_SO_4_, concentrated under vacuum and purified by silica gel column chromatograph using hexane–ethyl acetate (6:1 to 3:1, v/v) as eluent to give compound **17** (1.33 g, 85% yield) as a brown solid. M.p. 158–160 °C. IR (KBr) *ν*_max_/cm^–^^1^ 3401, 3337, 1638, 1574, 1530, 1508, 1421, 1365, 1337, 1323, 1197, 1166, 968, 953, 845, 822, 695, 612. ^1^H NMR (400 MHz, DMSO-*d*_6_) *δ* 8.61 (s, 1H), 7.94 (d, *J* = 5.6 Hz, 1H), 7.64 (d, *J* = 6.0 Hz, 1H), 7.07–7.03 (m, 1H), 6.51 (dd, *J* = 2.8, 13.2 Hz, 1H), 6.43 (dd, *J* = 2.4, 8.8 Hz, 1H), 5.43 (br s, 2H).

#### General procedure for the preparation of 3-fluoro-4-(thieno[3,2-b]pyridin-7-yloxy)aniline (22), 3-fluoro-4-(thieno[3,2-d]pyrimidin-4-yloxy)aniline (26), and 4-((6,7-dimethoxyquinazolin-4-yl)oxy)-3-fluoroaniline (30)

The intermediates **22**, **26**, and **30** were synthesised according to our previous procedures and some other literature (Supporting Information),^30^^,^^49^^,^^50^ as such only characterisation data for the title compounds are listed here.

##### 3-Fluoro-4-(thieno[3,2-b]pyridin-7-yloxy)aniline (22).^30^

Pale yellow solid; m.p. 137–139 °C. IR (KBr) *ν*_max_/cm^–^^1^ 3451, 3329, 3202, 1651, 1621, 1586, 1552, 1505, 1450, 1381, 1293, 1270, 1211, 1161, 1026, 846, 820, 800, 774, 700. ^1^H NMR (400 MHz, DMSO-*d*_6_) *δ* 8.49 (d, *J* = 5.2 Hz, 1H), 8.13 (d, *J* = 5.6 Hz, 1H), 7.57 (d, *J* = 5.6 Hz, 1H), 7.12–7.07 (m, 1H), 6.57 (d, *J* = 5.2 Hz, 1H), 6.53 (dd, *J* = 2.4, 13.2 Hz, 1H), 6.45 (dd, *J* = 2.0, 8.4 Hz, 1H), 5.55 (br s, 2H).

##### 3-Fluoro-4-(thieno[3,2-d]pyrimidin-4-yloxy)aniline (26).^30^^,^^49^

Brown solid; m.p. 165–167 °C. IR (KBr) *ν*_max_/cm^–^^1^ 3319, 1633, 1575, 1508, 1466, 1441, 1388, 1337, 1326, 1292, 1227, 1211, 1072, 1037, 847, 799, 543. ^1^H NMR (400 MHz, DMSO-*d*_6_) *δ* 8.70 (s, 1H), 8.46 (d, *J* = 5.2 Hz, 1H), 7.66 (d, *J* = 5.6 Hz, 1H), 7.10–7.06 (m, 1H), 6.50 (dd, *J* = 2.0, 12.8 Hz, 1H), 6.42 (dd, *J* = 1.6, 8.4 Hz, 1H), 5.47 (br s, 2H).

##### 4-((6,7-Dimethoxyquinazolin-4-yl)oxy)-3-fluoroaniline (30).^30^^,^^50^

Brown solid; m.p. 182–184 °C. IR (KBr) *ν*_max_/cm^–^^1^ 3353, 3179, 1624, 1573, 1511, 1465, 1448, 1419, 1376, 1256, 1242, 1210, 1163, 1138, 989, 907, 855, 843, 829. ^1^H NMR (400 MHz, DMSO*-d*_6_) *δ* 8.56 (s, 1H), 7.52 (s, 1H), 7.40 (s, 1H), 7.05–7.00 (m, 1H), 6.52 (dd, *J* = 2.4 Hz, *J* = 12.8 Hz, 1H), 6.42 (dd, *J* = 2.0 Hz, *J* = 8.4 Hz, 1H), 5.39 (br s, 2H), 3.96 (s, 3H), 3.94 (s, 3H).

#### Synthesis of 4-((2-chloropyridin-4-yl)oxy)-3-fluoroanilines (32a–32b)

To a solution of 4-amino-2-fluorophenol (1.39 g, 11 mmol) in DMF (15 mL), NaH (0.29 g, 12.1 mmol) was added and the resulting mixture was stirred at 0 °C for 0.5 h. Then, 2,4-dichloropyridine **31a** (1.48 g, 10 mmol) was added and the reaction mixture was stirred at 80 °C overnight. After cooling to room temperature, the mixture was poured into H_2_O (100 mL) and extracted with ethyl acetate (3 × 80 mL). The organic layer was washed with brine (3 × 100 mL), dried over Na_2_SO_4_, evaporated under reduced pressure and purified by flash column chromatography on silica gel using hexane–ethyl acetate (4:1, v/v) as eluent to give compound **32a** (1.47 g, 62% yield) as a yellow solid. M.p. 74–76 °C. IR (KBr) *ν*_max_/cm^–^^1^ 3409, 3224, 1617, 1591, 1559, 1509, 1459, 1388, 1300, 1260, 1237, 1204, 1162, 1117, 1064, 920, 844, 820. ^1^H NMR (400 MHz, DMSO-*d*_6_) *δ* 8.25 (d, *J* = 5.6 Hz, 1H), 7.02–6.98 (m, 1H), 6.94–6.88 (m, 2H), 6.52 (dd, *J* = 2.8, 13.2 Hz, 1H), 6.43 (dd, *J* = 2.4, 8.8 Hz, 1H), 5.52 (br s, 2H). Compound **32b** was prepared following the similar synthetic procedure of **32a**, only the reaction conditions 80 °C overnight were replaced with 100 °C for 3 h. Yellow solid; yield 57%; m.p. 134–136 °C. IR (KBr) *ν*_max_/cm^–^^1^ 3459, 3350, 3220, 1637, 1593, 1573, 1509, 1454, 1381, 1293, 1216, 1205, 1164, 1122, 1054, 956, 937, 841, 825. ^1^H NMR (400 MHz, DMSO-*d*_6_) *δ* 8.18 (d, *J* = 5.6 Hz, 1H), 7.07–7.03 (m, 1H), 6.73 (d, *J* = 5.6 Hz, 1H), 6.54 (dd, *J* = 2.4, 13.2 Hz, 1H), 6.44 (dd, *J* = 2.0, 8.4 Hz, 1H), 5.56 (br s, 2H).

#### Synthesis of 1,3,4-oxathiazol-2-ones (35a–*35b)*

A mixture of acid **33** (5 mmol) in excess SOCl_2_ (2.18 mL, 30 mmol) was stirred at 80 °C for 4 h. After cooling to room temperature, the mixture was concentrated in vacuum to remove excess SOCl_2_ and then suspended in THF (5 mL). The THF solution was added to an ice cooled NH_4_OH (37%, 10 mL). After stirring for 30 min at room temperature, the mixture was extracted with CHCl_3_ (3 × 50 mL). The combined organic extracts were dried and evaporated. The resultant solid was soaked with hexane and filtered to obtain the desired amide **34**. Then, (chlorothio)formyl chloride (4.5 mmol, 0.38 mL) was added to a solution of amide **34** (3 mmol) in toluene (5 mL) under N_2_ atmosphere. The reaction mixture was heated to 100 °C for 3 h. After cooling to room temperature, the mixture was quenched with H_2_O (20 mL) and extracted with ethyl acetate (3 × 20 mL). The combined organic layers were washed with saturated brine, dried over Na_2_SO_4_ and concentrated under reduced pressure. The residue was further purified by flash column chromatography on silica gel using hexane–ethyl acetate (12:1 to 6:1, v/v) as eluent to afford the title compounds.

##### 5-Phenyl-1,3,4-oxathiazol-2-one (35a)

White solid; yield 57% (three steps); m.p. 66–68 °C. IR (KBr) *ν*_max_/cm^–^^1^ 3472, 1741, 1593, 1560, 1493, 1447, 1322, 1291, 1091, 1070, 983, 886, 791, 770, 723, 689, 681, 663, 573, 473. ^1^H NMR (400 MHz, DMSO-*d*_6_) *δ* 7.92 (d, *J* = 7.2 Hz, 2H), 7.67–7.63 (m, 1H), 7.59–7.55 (m, 2H).

##### 5-(4-Fluorophenyl)-1,3,4-oxathiazol-2-one (35b)

White solid; yield 53% (three steps); m.p. 101–103 °C. IR (KBr) *ν*_max_/cm^–^^1^ 3472, 1784, 1755, 1601, 1574, 1505, 1413, 1293, 1240, 1223, 1160, 1093, 984, 891, 844, 781, 722, 676, 592, 508. ^1^H NMR (400 MHz, DMSO-*d*_6_) *δ* 7.99–7.94 (m, 2H), 7.44–7.38 (m, 2H).

#### Synthesis of 1,2,4-thiadiazole-5-carboxylates (36a–36b)

A solution of **35** (2 mmol) and ECF (1.97 mL, 20 mmol) in *n*-dodecane (4 mL) was heated at 160 °C for 16 h. After removal of the excess ECF and *n*-dodecane under reduced pressure, the residue was purified by flash column chromatography on silica gel using hexane–ethyl acetate (9:1 to 4:1, v/v) as eluent to afford the desired compounds.

##### 3-Phenyl-1,2,4-thiadiazole-5-carboxylic acid ethyl ester (36a)

White solid; yield 75%; m.p. 68–70 °C. IR (KBr) *ν*_max_/cm^–^^1^ 3002, 2984, 2907, 1712, 1498, 1470, 1414, 1366, 1325, 1307, 1287, 1258, 1119, 1095, 1008, 843, 764, 711, 685. ^1^H NMR (400 MHz, DMSO-*d*_6_) *δ* 8.24–8.22 (m, 2H), 7.58–7.55 (m, 3H), 4.46 (q, *J* = 7.2 Hz, 2H), 1.37 (t, *J* = 7.2 Hz, 3H).

##### 3-(4-Fluorophenyl)-1,2,4-thiadiazole-5-carboxylic acid ethyl ester (36b)

Pale yellow solid; yield 68%; m.p. 70–72 °C. IR (KBr) *ν*_max_/cm^–^^1^ 3471, 3057, 2987, 1742, 1599, 1518, 1488, 1414, 1294, 1237, 1156, 1121, 1095, 1008, 850, 823, 744, 695. ^1^H NMR (400 MHz, DMSO-*d*_6_) *δ* 8.28–8.23 (m, 2H), 7.40–7.35 (m, 2H), 4.46 (q, *J* = 7.2 Hz, 2H), 1.37 (t, *J* = 7.2 Hz, 3H).

#### Synthesis of 1,2,4-thiadiazole-5-carboxylic acids (37a–37b)

Aqueous solution (1 mL, 2 N LiOH) was added to a solution of carboxylic ester **36** (1 mmol) in methanol (2 mL) at room temperature. The reaction mixture was stirred for 4 h and methanol was then removed by evaporation in vacuum. The resulting residue was adjusted to pH = 2–3 with 1 N HCl. The precipitated solid was collected by filtration and dried to give carboxylic acid **37**, which was used directly in the next step without further purification.

##### 3-Phenyl-1,2,4-thiadiazole-5-carboxylic acid (37a)

White solid; yield 71%; m.p. 90–92 °C. IR (KBr) *ν*_max_/cm^–^^1^ 3650, 3444, 1718, 1693, 1513, 1429, 1411, 1314, 1258, 1123, 1105, 776, 711, 688. ^1^H NMR (400 MHz, DMSO-*d*_6_) *δ* 10.36 (s, 1H), 8.27–8.23 (m, 2H), 7.56–7.52 (m, 3H).

##### 3-(4-Fluorophenyl)-1,2,4-thiadiazole-5-carboxylic acid (37b)

Off-white solid; yield 64%; m.p. 106–108 °C. IR (KBr) *ν*_max_/cm^–^^1^ 3650, 3446, 1737, 1664, 1600, 1518, 1489, 1422, 1409, 1332, 1233, 1161, 837, 746. ^1^H NMR (400 MHz, DMSO-*d*_6_) *δ* 10.35 (s, 1H), 8.30–8.24 (m, 2H), 7.38–7.33 (m, 2H).

#### Synthesis of 5-amino-1,3,4-thiadiazole-2-carboxylic acid ethyl ester (39)

To a stirred solution of thiosemicarbazide **38** (1.64 g, 18 mmol) in POCl_3_ (8.4 mL, 90 mmol), ethyl oxalyl monochloride was added (2.1 mL, 18.9 mmol). The reaction mixture was heated to 70 °C for 6 h. Excess POCl_3_ was completely removed under reduced pressure. The residue was poured into cold water (50 mL), basified to pH = 8 with saturated NaHCO_3_ solution and extracted with ethyl acetate (60 mL). The organic layer was separated, dried over Na_2_SO_4_ and concentrated in vacuum. The residue was purified by flash chromatography on silica gel using hexane–ethyl acetate (4:1, v/v) as eluent to afford **39** (1.31 g, 42% yield) as a yellow solid. M.p. 195–197 °C. IR (KBr) *ν*_max_/cm^–^^1^ 3423, 3321, 3198, 2986, 2899, 1644, 1619, 1539, 1458, 1398, 1283, 1219, 1125, 1098, 1014, 897, 780, 749, 488. ^1^H NMR (400 MHz, DMSO-*d*_6_) *δ* 8.56 (br s, 2H), 4.32 (q, *J* = 7.2 Hz, 2H), 1.30 (t, *J* = 7.2 Hz, 3H).

#### Synthesis of 5-bromo-1,3,4-thiadiazole-2-carboxylic acid ethyl ester (40)

To a solution of **39** (1.21 g, 7 mmol) in CH_3_CN (10 mL), CuBr_2_ was added (3.12 g, 14 mmol) and the mixture was stirred at room temperature for 20 min. *tert-*Butyl nitrite (1.68 mL, 14.1 mmol) was then added for 10 min, and the resulting mixture was heated to 60 °C for 30 min. The reaction mixture was concentrated, diluted with water (80 mL) and extracted with ethyl acetate (2 × 60 mL). The combined organic layer was separated, dried over Na_2_SO_4_ and evaporated to give the title compound **40** (1.16 g, 70% yield) as a dark yellow solid. M.p. 83–85 °C. IR (KBr) *ν*_max_/cm^–^^1^ 3463, 2987, 2699, 1748, 1474, 1451, 1360, 1265, 1140, 1107, 1068, 1036, 1012, 831, 772, 757. ^1^H NMR (400 MHz, DMSO-*d*_6_) *δ* 4.42 (q, *J* = 7.2 Hz, 2H), 1.34 (t, *J* = 7.2 Hz, 3H).

#### *Synthesis of 5-substituted 1,3,4-thiadiazole-2-carboxylic acid ethyl esters (41a*–*41b)*

To a mixture of **40** (543 mg, 2.3 mmol), corresponding boronic acid (2.76 mmol), Pd(OAc)_2_ (11.3 mg, 0.05 mmol) and Xantphos (29 mg, 0.05 mmol) was added *N*-methyl morpholine (0.56 mL, 5.1 mmol), toluene (4 mL), and water (2 mL) at room temperature. The reaction mixture was strongly stirred at room temperature for 7 h and then extracted with ethyl acetate (3 × 10 mL). The organic layer was washed with water (20 mL) and brine (20 mL), dried over MgSO_4_, filtered and concentrated in vacuum. The residue was purified by flash chromatography on silica gel using hexane–ethyl acetate (12:1 to 6:1, v/v) as eluent to give product **41**.

##### 5-Phenyl-1,3,4-thiadiazole-2-carboxylic acid ethyl ester (41a)

Pale yellow solid; yield 83%; m.p. 74–76 °C. IR (KBr) *ν*_max_/cm^–^^1^ 3428, 2988, 1725, 1455, 1441, 1405, 1306, 1276, 1233, 1116, 1090, 1001, 762, 689, 637, 566. ^1^H NMR (400 MHz, DMSO-*d*_6_) *δ* 8.07 (d, *J* = 6.8 Hz, 2H), 7.65–7.55 (m, 3H), 4.44 (q, *J* = 7.2 Hz, 2H), 1.36 (t, *J* = 7.2 Hz, 3H).

##### 5-(4-Fluorophenyl)-1,3,4-thiadiazole-2-carboxylic acid ethyl ester (41b)

Golden yellow solid; yield 76%; m.p. 93–95 °C. IR (KBr) *ν*_max_/cm^–^^1^ 3421, 2986, 1716, 1597, 1509, 1479, 1445, 1422, 1396, 1369, 1309, 1219, 1157, 1111, 1086, 1016, 843, 603, 564. ^1^H NMR (400 MHz, DMSO-*d*_6_) *δ* 8.18–8.14 (m, 2H), 7.46–7.41 (m, 2H), 4.45 (q, *J* = 7.2 Hz, 2H), 1.36 (t, *J* = 7.2 Hz, 3H).

#### Synthesis of thiazole-2-carboxylic acids (46a–46p)

To a solution of ketone **43** (8 mmol) in CH_3_CN (4 mL), NBS (1.5 g, 8.4 mmol) and *p-*toluenesulfonic acid (1.38 g, 8 mmol) were added. The reaction mixture was heated to 50 °C and stirred for 24 h. The solvent was evaporated under reduced pressure and saturated NaHCO_3_ (30 mL) was added to the residue. The mixture was extracted with CH_2_Cl_2_ (3 × 20 mL); the combined organic layers were dried over Na_2_SO_4_ and evaporated in vacuum. The crude product was purified by flash column chromatography on silica gel using hexane–ethyl acetate (15:1 to 6:1, v/v) as eluent to afford α-bromoketone **44**. Afterwards, **44** (5 mmol) was added to a solution of ethyl thiooxamate (692 mg, 5.2 mmol) in ethanol (20 mL), the resulting mixture was heated to reflux for 6 h. The reaction mixture was then concentrated in vacuum, diluted with ethyl acetate (30 mL), and washed with 1 N NaHCO_3_ (3 × 20 mL) and brine (2 × 20 mL). The organic layer was dried over Na_2_SO_4_, filtered and evaporated under vacuum to obtain compound **45**, which underwent a similar procedure as described for **37** to afford the title compounds.

##### 4-Phenylthiazole-2-carboxylic acid (46a)

White solid; yield 38% (three steps); m.p. 76–78 °C. IR (KBr) *ν*_max_/cm^–^^1^ 3423, 3117, 1894, 1720, 1662, 1487, 1439, 1317, 1262, 1205, 1110, 1073, 1025, 764, 689, 672, 634. ^1^H NMR (400 MHz, DMSO-*d*_6_) *δ* 8.46 (s, 1H), 7.99 (d, *J* = 7.6 Hz, 2H), 7.49–7.42 (m, 3H).

##### 4-(4-Fluorophenyl)thiazole-2-carboxylic acid (46b)

White solid; yield 45% (three steps); m.p. 110–112 °C. IR (KBr) *ν*_max_/cm^–^^1^ 3424, 3112, 1870, 1727, 1670, 1602, 1523, 1445, 1325, 1218, 1202, 1164, 1120, 843, 805, 771. ^1^H NMR (400 MHz, DMSO-*d*_6_) *δ* 8.43, 8.03 (dd, *J* = 5.6, 8.8 Hz, 2H), 7.32–7.27 (m, 2H).

##### 4-(p-Tolyl)thiazole-2-carboxylic acid (46c)

Pale yellow solid; yield 31% (three steps); m.p. 117–119 °C. IR (KBr) *ν*_max_/cm^–^^1^ 3467, 3104, 1963, 1697, 1613, 1490, 1447, 1282, 1254, 1126, 1066, 822, 783, 772, 758. ^1^H NMR (400 MHz, DMSO-*d*_6_) *δ* 8.37 (s, 1H), 7.88 (d, *J* = 8.0 Hz, 2H), 7.26 (d, *J* = 8.0 Hz, 2H), 2.32 (s, 3H).

##### 4-(4-Methoxyphenyl)thiazole-2-carboxylic acid (46d)

Off-white solid; yield 42% (three steps); m.p. 116–118 °C. IR (KBr) *ν*_max_/cm^–^^1^ 3467, 3110, 2837, 2558, 1973, 1682, 1609, 1530, 1490, 1450, 1435, 1304, 1280, 1252, 1178, 1125, 1100, 1067, 1026, 770. ^1^H NMR (400 MHz, DMSO-*d*_6_) *δ* 8.28 (s, 1H), 7.92 (d, *J* = 8.4 Hz, 2H), 7.02 (d, *J* = 8.4 Hz, 2H), 3.79 (s, 3H).

##### 4-(3,4-Dimethoxyphenyl)thiazole-2-carboxylic acid (46e)

Pale yellow solid; yield 27% (three steps); m.p. 85–87 °C. IR (KBr) *ν*_max_/cm^–^^1^ 3748, 3503, 2939, 1716, 1527, 1496, 1463, 1436, 1405, 1289, 1267, 1244, 1166, 1147, 1112, 1021, 766. ^1^H NMR (400 MHz, DMSO-*d*_6_) *δ* 8.33 (s, 1H), 7.56–7.54 (m, 2H), 7.03 (d, *J* = 8.4 Hz, 1H), 3.83 (s, 3H), 3.79 (s, 3H).

##### 4-(3-Fluorophenyl)thiazole-2-carboxylic acid (46f)

White solid; yield 48% (three steps); m.p. 84–86 °C. IR (KBr) *ν*_max_/cm^–^^1^ 3448, 1703, 1646, 1616, 1591, 1490, 1455, 1376, 1305, 1264, 1235, 1175, 1131, 1079, 944, 816, 776, 756. ^1^H NMR (400 MHz, DMSO-*d*_6_) *δ* 8.40 (s, 1H), 7.85–7.78 (m, 2H), 7.48 (d, *J* = 7.2 Hz, 1H), 7.18 (s, 1H).

##### 4-(2-Fluorophenyl)thiazole-2-carboxylic acid (46g)

Yellow solid; yield 35% (three steps); m.p. 78–80 °C. IR (KBr) *ν*_max_/cm^–^^1^ 3443, 1704, 1646, 1495, 1456, 1435, 1378, 1299, 1255, 1216, 1127, 1061, 868, 811, 785, 749. ^1^H NMR (400 MHz, DMSO-*d*_6_) *δ* 8.15–8.12 (m, 2H), 7.44–7.39 (m, 1H), 7.35–7.29 (m, 2H).

##### 4-(4-Chlorophenyl)thiazole-2-carboxylic acid (46h)

Pale yellow solid; yield 48% (three steps); m.p. 118–120 °C. IR (KBr) *ν*_max_/cm^–^^1^ 3478, 3114, 1946, 1688, 1597, 1486, 1448, 1294, 1278, 1257, 1123, 1092, 1064, 1013, 832, 782, 772. ^1^H NMR (400 MHz, DMSO-*d*_6_) *δ* 8.50 (s, 1H), 8.00 (d, *J* = 8.0 Hz, 2H), 7.52 (d, *J* = 8.4 Hz, 2H).

##### 4-(3-Chlorophenyl)thiazole-2-carboxylic acid (46i)

Yellow solid; yield 39% (three steps); m.p. 103–105 °C. IR (KBr) *ν*_max_/cm^–^^1^ 3421, 1885, 1722, 1659, 1595, 1573, 1510, 1481, 1442, 1424, 1318, 1207, 1119, 1075, 874, 771. ^1^H NMR (400 MHz, DMSO-*d*_6_) *δ* 8.58 (s, 1H), 8.04 (s, 1H), 7.95 (d, *J* = 7.6 Hz, 1H), 7.51–7.42 (m, 2H).

##### 4-(4-Bromophenyl)thiazole-2-carboxylic acid (46j)

Pale yellow solid; yield 55% (three steps); m.p. 117–119 °C. IR (KBr) *ν*_max_/cm^–^^1^ 3496, 3111, 1684, 1508, 1495, 1450, 1442, 1398, 1280, 1261, 1097, 1069, 1009, 845, 831, 773. ^1^H NMR (400 MHz, DMSO-*d*_6_) *δ* 8.49 (s, 1H), 7.94 (d, *J* = 8.4 Hz, 2H), 7.65 (d, *J* = 8.4 Hz, 2H).

##### 4-(4-(Trifluoromethyl)phenyl)thiazole-2-carboxylic acid (46k)

Yellow solid; yield 21% (three steps); m.p. 116–118 °C. IR (KBr) *ν*_max_/cm^–^^1^ 3449, 1953, 1685, 1618, 1492, 1454, 1322, 1260, 1170, 1125, 1072, 1060, 1018, 851, 781. ^1^H NMR (400 MHz, DMSO-*d*_6_) *δ* 8.65 (s, 1H), 8.20 (d, *J* = 8.0 Hz, 2H), 7.81 (d, *J* = 8.4 Hz, 2H).

##### 4-(3,4-Dichlorophenyl)thiazole-2-carboxylic acid (46l)

Pale yellow solid; yield 52% (three steps); m.p. 126–128 °C. IR (KBr) *ν*_max_/cm^–^^1^ 3435, 3112, 1729, 1638, 1507, 1482, 1434, 1294, 1223, 1139, 1114, 1028, 871, 827, 775. ^1^H NMR (400 MHz, DMSO-*d*_6_) *δ* 8.59 (s, 1H), 8.18 (s, 1H), 7.94 (d, *J* = 8.4 Hz, 1H), 7.68 (d, *J* = 8.0 Hz, 1H).

##### 4-(Naphthalen-2-yl)thiazole-2-carboxylic acid (46m)

Pale red solid; yield 54% (three steps); m.p. 128–130 °C. IR (KBr) *ν*_max_/cm^–^^1^ 3435, 3110, 1708, 1676, 1477, 1434, 1319, 1287, 1256, 1212, 1127, 1100, 892, 863, 836, 779, 761, 751. ^1^H NMR (400 MHz, DMSO-*d*_6_) *δ* 8.58 (s, 1H), 8.56 (s, 1H), 8.13 (d, *J* = 8.8 Hz, 1H), 8.02–7.99 (m, 2H), 7.94–7.92 (m, 1H), 7.55–7.50 (m, 2H).

##### 4-(3-Chloro-4-fluorophenyl)thiazole-2-carboxylic acid (46n)

White solid; yield 40% (three steps); m.p. 119–121 °C. IR (KBr) *ν*_max_/cm^–^^1^ 3435, 1733, 1706, 1677, 1519, 1489, 1443, 1297, 1246, 1110, 1054, 927, 872, 833, 774. ^1^H NMR (400 MHz, DMSO-*d*_6_) *δ* 8.53 (s, 1H), 8.15 (d, *J* = 7.2 Hz, 1H), 7.97 (s, 1H), 7.50–7.46 (m, 1H).

##### 4-(Thiophen-2-yl)thiazole-2-carboxylic acid (46o)

Yellow solid; yield 47% (three steps); m.p. 118–120 °C. IR (KBr) *ν*_max_/cm^–^^1^ 3452, 3083, 2872, 1680, 1547, 1489, 1460, 1412, 1293, 1280, 1219, 1097, 1056, 899, 817, 769, 730, 696. ^1^H NMR (400 MHz, DMSO-*d*_6_) *δ* 8.29 (s, 1H), 7.65 (d, *J* = 3.6 Hz, 1H), 7.58 (d, *J* = 4.8 Hz, 1H), 7.14–7.12 (m, 1H).

##### 4-(3,4-Difluorophenyl)thiazole-2-carboxylic acid (46p)

White solid; yield 29% (three steps); m.p. 125–127 °C. IR (KBr) *ν*_max_/cm^–^^1^ 3436, 3107, 1720, 1707, 1608, 1533, 1502, 1460, 1416, 1379, 1313, 1275, 1208, 1170, 1121, 1061, 885, 819, 775. ^1^H NMR (400 MHz, DMSO-*d*_6_) *δ* 8.50 (s, 1H), 8.02–7.96 (m, 1H), 7.83 (br s, 1H), 7.54–7.48 (m, 1H).

#### Synthesis of 2-oxo-1-ethylamine hydrochlorides (47a–47b)

α-Bromoketone (**44a**–**44b**, 10 mmol) and urotropine (1.4 g, 10 mmol) were dissolved in dry CHCl_3_ (50 mL). The mixture was stirred at 50 °C for 2 h. The white precipitate was isolated by filtration and washed with CHCl_3_ and ethanol to yield the urotropin salt, which was then dissolved in a mixture of absolute ethanol (50 mL) and concentrated HCl (5 mL). The resulting mixture was heated to reflux for 2 h. The residual solid was removed by filtration and the filtrate was evaporated under reduced pressure to yield the title compounds.

##### 2-Amino-1-(phenyl)ethanone hydrochloride (47a)

Brown solid; yield 82%; m.p. 209–211 °C. IR (KBr) *ν*_max_/cm^–^^1^ 3375, 2924, 2609, 1697, 1596, 1548, 1504, 1448, 1431, 1373, 1242, 1120, 1076, 964, 900, 761, 690. ^1^H NMR (400 MHz, DMSO-*d*_6_) *δ* 8.47 (br s, 2H), 8.01 (d, *J* = 7.6 Hz, 2H), 7.71 (d, *J* = 7.2 Hz, 1H), 7.59–7.55 (m, 2H), 4.60 (s, 2H).

##### 2-Amino-1-(4′-fluorophenyl)ethanone hydrochloride (47b)

White solid; yield 67%; m.p. 238–240 °C. IR (KBr) *ν*_max_/cm^–^^1^ 3435, 2982, 2584, 1682, 1600, 1509, 1470, 1430, 1417, 1302, 1255, 1237, 1167, 1124, 1105, 974, 841. ^1^H NMR (400 MHz, DMSO-*d*_6_) *δ* 8.59 (br s, 2H), 8.11 (dd, *J* = 5.6, 8.8 Hz, 2H), 7.44–7.39 (m, 2H), 4.56 (s, 2H).

#### Synthesis of amides (48a–48b)

To a stirred solution of **47** (6 mmol) in CH_2_Cl_2_ (18 mL) under 0 °C, ethyl oxalyl monochloride (0.73 mL, 6.5 mmol) and Et_3_N (1.66 mL, 12 mmol) were added. The reaction mixture was warmed to room temperature and stirred overnight. The mixture was partitioned between CH_2_Cl_2_ and aq. HCl (1 N). After extraction with CH_2_Cl_2_ (2 × 30 mL), the combined organic layers were washed with water and saturated brine, dried over Na_2_SO_4_ and concentrated in vacuum to obtain the crude product **48**, which was used directly in the next step without further purification.

##### Ethyl 2-oxo-2-((2-oxo-2-phenylethyl)amino)acetate (48a)

Off-white solid; yield 78%; m.p. 95–97 °C. IR (KBr) *ν*_max_/cm^–^^1^ 3390, 3066, 2983, 2914, 1733, 1724, 1683, 1589, 1519, 1448, 1432, 1357, 1293, 1238, 1199, 1019, 994, 858, 754, 688, 510. ^1^H NMR (400 MHz, DMSO-*d*_6_) *δ* 9.11 (s, 1H), 8.01 (d, *J* = 6.8 Hz, 2H), 7.70–7.66 (m, 1H), 7.57–7.53 (m, 2H), 4.71 (d, *J* = 6.0 Hz, 2H), 4.28 (q, *J* = 7.2 Hz, 2H), 1.29 (t, *J* = 7.2 Hz, 3H).

##### Ethyl 2-((2-(4-fluorophenyl)-2-oxoethyl)amino)-2-oxoacetate (48b)

Off-white solid; yield 83%; m.p. 121–123 °C. IR (KBr) *ν*_max_/cm^–^^1^ 3391, 3076, 2985, 2919, 1734, 1723, 1682, 1596, 1511, 1415, 1357, 1293, 1237, 1198, 1161, 1103, 1018, 991, 857, 836, 601, 568. ^1^H NMR (400 MHz, DMSO-*d*_6_) *δ* 9.11 (s, 1H), 8.10 (dd, *J* = 5.6, 8.8 Hz, 2H), 7.40–7.35 (m, 2H), 4.69 (d, *J* = 5.6 Hz, 2H), 4.27 (q, *J* = 7.2 Hz, 2H), 1.29 (t, *J* = 7.2 Hz, 3H).

#### Synthesis of 4-phenylthiazole-2-carboxylic acid ethyl esters (49a–49b)

Phosphorus pentasulfide (1.78 g, 8 mmol) was added to a solution of **48** (4 mmol) in dry CHCl_3_ (10 mL) and the mixture was heated to reflux for 5 h. After cooling to room temperature, the mixture was quenched with water and extracted with CHCl_3_. The organic phase was washed with water and brine, dried over Na_2_SO_4_ and concentrated in vacuum. The residue was purified by flash column chromatography on silica gel using hexane–ethyl acetate (7:1 to 3:1, v/v) as eluent to afford the desired compounds.

##### 5-Phenylthiazole-2-carboxylic acid ethyl ester (49a)

Golden-yellow solid; yield 64%, m.p. 70–72 °C. IR (KBr) *ν*_max_/cm^–^^1^ 3434, 2974, 1702, 1518, 1476, 1446, 1419, 1392, 1364, 1296, 1164, 1118, 1086, 1016, 861, 760, 690, 610, 555. ^1^H NMR (400 MHz, DMSO-*d*_6_) *δ* 8.49 (s, 1H), 7.79–7.76 (m, 2H), 7.50–7.41 (m, 3H), 4.38 (q, *J* = 7.2 Hz, 2H), 1.33 (t, *J* = 7.2 Hz, 3H).

##### 5-(4-Fluorophenyl)thiazole-2-carboxylic acid ethyl ester (49b)

Yellow solid; yield 57%, m.p. 110–112 °C. IR (KBr) *ν*_max_/cm^–^^1^ 3434, 3087, 1702, 1599, 1527, 1478, 1426, 1411, 1394, 1323, 1298, 1230, 1182, 1166, 1086, 1019, 838, 757, 595. ^1^H NMR (400 MHz, DMSO-*d*_6_) *δ* 8.45 (s, 1H), 7.82 (dd, *J* = 5.6, 8.8 Hz, 2H), 7.33–7.28 (m, 2H), 4.37 (q, *J* = 7.2 Hz, 2H), 1.33 (t, *J* = 7.2 Hz, 3H).

#### Synthesis of 5-phenylthiazole-2-carboxylic acids (50a–50b)

Compound **50** was prepared from intermediate **49** following the similar procedure as described for **37**.

##### 5-Phenylthiazole-2-carboxylic acid (50a)

Off-white solid; yield 71%; m.p. 113–115 °C. IR (KBr) *ν*_max_/cm^–^^1^ 3427, 2459, 1852, 1736, 1705, 1448, 1426, 1270, 1153, 1118, 870, 790, 770, 759, 685. ^1^H NMR (400 MHz, DMSO-*d*_6_) *δ* 9.08 (s, 1H), 8.31 (s, 1H), 7.69–7.67 (m, 2H), 7.49–7.42 (m, 3H).

##### 5-(4-Fluorophenyl)thiazole-2-carboxylic acid (50b)

Pale yellow solid; yield 76%; m.p. 128–130 °C. IR (KBr) *ν*_max_/cm^–^^1^ 3106, 2535, 1705, 1600, 1534, 1465, 1419, 1404, 1326, 1303, 1244, 1189, 1161, 1111, 881, 826, 785. ^1^H NMR (400 MHz, DMSO-*d*_6_) *δ* 9.06 (s, 1H), 8.26 (s, 1H), 7.74–7.69 (m, 2H), 7.33–7.26 (m, 2H).

#### Synthesis of target compounds 51a–51an

- The prepared acid (**37**, **46**, and **50**, 1 mmol) was suspended in dry CH_2_Cl_2_ (5 mL); oxalyl chloride (0.17 mL, 2 mmol) and DMF (one drop) were added at 0 °C. The reaction mixture was stirred at room temperature for 1.5 h and concentrated in vacuum to afford the corresponding acyl chloride. The acyl chloride without further purification was dissolved in dry CH_2_Cl_2_ (2 mL) and cooled to 0  °C. The corresponding amine (**13**, **17**, **22**, **26**, **30**, and **32**, 1 mmol) was added thereinto, the mixture was warmed to room temperature and stirred for 4–6 h. The solvent was concentrated in vacuum and the residue was purified by flash chromatography on silica gel using hexane–ethyl acetate (8:1 to 2:1, v/v) as eluent to give the target compounds **51a**–**51b** and **51e**–**51an**.
- Aqueous solution (1 mL, 2 N LiOH) was added to a solution of carboxylic ester **41** (1 mmol) in methanol (2 mL) at 0  °C and the resulting mixture was continuously stirred for 1 h. Then, the solvent was removed by evaporation in vacuum to afford the lithium salt **42** which was used directly. Then, HATU (570 mg, 1.5 mmol) and Et_3_N (0.21 mL, 1.5 mmol) were added to a stirred solution of **42** (1 mmol) in DMF (4 mL) at room temperature. The mixture was stirred for 10 min followed by addition of **13a** (296 mg, 1 mmol). The resulting mixture was stirred at room temperature overnight. Water (10 mL) was added to the reaction solution and the mixture was extracted with ethyl acetate (3 × 10 mL). The organic layer was washed with brine, dried over Na_2_SO_4_, concentrated in vacuum and purified by flash chromatography on silica gel using hexane–ethyl acetate (5:1 to 2:1, v/v) as eluent to yield the target compounds **51c**–**51d**.

##### N-(4-((6,7-Dimethoxyquinolin-4-yl)oxy)phenyl)-3-phenyl-1,2,4-thiadiazole-5-carboxamide (51a)

Pale yellow solid; yield 63% (two steps); m.p. 145–147 °C. HPLC purity 95.2%. IR (KBr) *ν*_max_/cm^–^^1^ 3381, 1691, 1626, 1579, 1528, 1506, 1482, 1431, 1414, 1350, 1306, 1253, 1214, 1202, 1174, 1119, 1080, 996, 849, 713. ^1^H NMR (600 MHz, DMSO-*d*_6_) *δ* 11.14 (s, 1H), 8.50 (d, *J* = 4.8 Hz, 1H), 8.38 (dd, *J* = 2.4, 7.2 Hz, 2H), 8.02 (d, *J* = 9.0 Hz, 2H), 7.63–7.59 (m, 3H), 7.51 (s, 1H), 7.40 (s, 1H), 7.35 (d, *J* = 9.0 Hz, 2H), 6.53 (d, *J* = 5.4 Hz, 1H), 3.95 (s, 3H), 3.93 (s, 3H); ^13^C NMR (150 MHz, DMSO-*d*_6_) *δ* 184.9, 173.0, 159.6, 156.2, 152.6, 150.7, 149.3, 148.8, 146.4, 134.6, 131.7, 131.1, 129.0, 128.1, 122.9, 121.3, 115.2, 107.8, 103.4, 99.0, 55.7, 55.6; ESI-MS: *m*/*z* 485.2 [M + H]^+^.

##### N-(4-((6,7-Dimethoxyquinolin-4-yl)oxy)phenyl)-3-(4-fluorophenyl)-1,2,4-thiadiazole-5-carboxamide (51b)

Yellow solid; yield 74% (two steps); m.p. 113–115 °C. HPLC purity 96.5%. IR (KBr) *ν*_max_/cm^–^^1^ 3435, 1667, 1605, 1559, 1546, 1538, 1505, 1479, 1430, 1409, 1349, 1305, 1253, 1214, 1154, 1116, 1080, 995, 849, 746. ^1^H NMR (600 MHz, DMSO-*d*_6_) *δ* 11.13 (s, 1H), 8.50 (d, *J* = 5.4 Hz, 1H), 8.42 (dd, *J* = 5.4, 8.4 Hz, 2H), 8.01 (d, *J* = 9.0 Hz, 2H), 7.51 (s, 1H), 7.47–7.44 (m, 2H), 7.40 (s, 1H), 7.35 (d, *J* = 9.0 Hz, 2H), 6.52 (d, *J* = 5.4 Hz, 1H), 3.95 (s, 3H), 3.93 (s, 3H); ^13^C NMR (150 MHz, DMSO-*d*_6_) *δ* 185.0, 172.0, 163.8 (d, *J* = 247.6 Hz), 159.6, 156.1, 152.6, 150.8, 149.3, 148.8, 146.4, 134.6, 130.5 (d, *J* = 8.7 Hz), 128.4 (d, *J* = 1.9 Hz), 122.9, 121.3, 116.1 (d, *J* = 21.9 Hz), 115.2, 107.8, 103.4, 99.0, 55.7, 55.6; ESI-MS: *m*/*z* 503.1 [M + H]^+^.

##### N-(4-((6,7-Dimethoxyquinolin-4-yl)oxy)phenyl)-5-phenyl-1,3,4-thiadiazole-2-carboxamide (51c)

Yellow solid; yield 72% (two steps); m.p. 141–143 °C. HPLC purity 96.1%. IR (KBr) *ν*_max_/cm^–^^1^ 3423, 1725, 1658, 1630, 1560, 1513, 1476, 1435, 1402, 1364, 1325, 1255, 1210, 1185, 1132, 1074, 985, 832, 694. ^1^H NMR (600 MHz, DMSO-*d*_6_) *δ* 10.99 (s, 1H), 8.54 (s, 1H), 8.49 (d, *J* = 5.4 Hz, 1H), 8.04 (d, *J* = 7.8 Hz, 2H), 7.84 (d, *J* = 7.0 Hz, 2H), 7.53–7.44 (m, 5H), 7.31 (d, *J* = 8.4 Hz, 1H), 6.50 (d, *J* = 5.4 Hz, 1H), 3.95 (s, 3H), 3.94 (s, 3H); ^13^C NMR (100 MHz, DMSO-*d*_6_) *δ* 162.6, 160.3, 158.2, 153.0, 150.6, 149.8, 149.3, 145.0, 140.5, 136.0, 130.6, 129.9, 127.4, 123.6, 122.9, 121.8, 115.6, 108.3, 103.7, 99.6, 56.2, 56.1; ESI-MS: *m*/*z* 485.1 [M + H]^+^.

##### N-(4-((6,7-Dimethoxyquinolin-4-yl)oxy)phenyl)-5-(4-fluorophenyl)-1,3,4-thiadiazole-2-carboxamide (51d)

Pale yellow solid; yield 63% (two steps); m.p. 136–138 °C. HPLC purity 97.0%. IR (KBr) *ν*_max_/cm^–^^1^ 3450, 1699, 1646, 1605, 1504, 1488, 1465, 1432, 1389, 1357, 1316, 1254, 1210, 1172, 1135, 1074, 982, 841, 725. ^1^H NMR (600 MHz, DMSO-*d*_6_) *δ* 11.01 (s, 1H), 8.51 (s, 1H), 8.49 (d, *J* = 4.8 Hz, 1H), 8.04 (d, *J* = 8.7 Hz, 2H), 7.91 (dd, *J* = 5.4, 8.3 Hz, 2H), 7.52 (s, 1H), 7.38–7.35 (m, 2H), 7.31 (d, *J* = 8.6 Hz, 2H), 6.50 (d, *J* = 4.8 Hz, 1H), 3.95 (s, 3H), 3.94 (s, 3H); ^13^C NMR (100 MHz, DMSO-*d*_6_) *δ* 163.0 (d, *J* = 246.1 Hz), 162.6, 160.3, 158.1, 153.0, 150.5, 149.8, 149.3, 146.9, 140.6, 135.9, 129.7 (d, *J* = 7.9 Hz), 127.2 (d, *J* = 2.4 Hz), 122.8, 121.8, 116.9 (d, *J* = 21.7 Hz), 115.6, 108.3, 103.6, 99.5, 56.2, 56.1; ESI-MS: *m*/*z* 503.3 [M + H]^+^.

##### N-(4-((6,7-Dimethoxyquinolin-4-yl)oxy)phenyl)-4-phenylthiazole-2-carboxamide (51e)

Yellow solid; yield 56% (two steps); m.p. 63–65 °C. HPLC purity 97.4%. IR (KBr) *ν*_max_/cm^–^^1^ 3434, 2922, 1672, 1580, 1559, 1531, 1505, 1479, 1431, 1349, 1305, 1252, 1215, 1168, 1080, 994, 853, 755. ^1^H NMR (600 MHz, DMSO-*d*_6_) *δ* 10.79 (s, 1H), 8.52 (s, 1H), 8.49 (d, *J* = 5.4 Hz, 1H), 8.18 (d, *J* = 7.2 Hz, 2H), 8.02 (d, *J* = 8.4 Hz, 2H), 7.53–7.50 (m, 3H), 7.43 (d, *J* = 7.2 Hz, 1H), 7.40 (s, 1H), 7.33 (d, *J* = 9.0 Hz, 2H), 6.52 (d, *J* = 4.8 Hz, 1H), 3.95 (s, 3H), 3.94 (s, 3H); ^13^C NMR (150 MHz, DMSO-*d*_6_) *δ* 163.2, 159.7, 157.7, 155.5, 152.5, 150.2, 149.3, 148.8, 146.5, 135.2, 133.4, 128.8, 128.6, 126.4, 122.6, 121.3, 120.3, 115.2, 107.8, 103.3, 99.0, 55.7, 55.6; ESI-MS: *m*/*z* 484.2 [M + H]^+^.

##### N-(4-((6,7-Dimethoxyquinolin-4-yl)oxy)phenyl)-4-(4-fluorophenyl)thiazole-2-carboxamide (51f)

Yellow solid; yield 65% (two steps); m.p. 84–86 °C. HPLC purity 95.9%. IR (KBr) *ν*_max_/cm^–^^1^ 3434, 2924, 1667, 1604, 1584, 1559, 1531, 1506, 1481, 1431, 1350, 1253, 1216, 1157, 1080, 995, 849. ^1^H NMR (600 MHz, DMSO-*d*_6_) *δ* 10.79 (s, 1H), 8.50–8.49 (m, 2H), 8.23 (dd, *J* = 6.0, 7.8 Hz, 2H), 8.01 (d, *J* = 8.4 Hz, 2H), 7.52 (s, 1H), 7.40 (s, 1H), 7.38–7.35 (m, 2H), 7.33 (d, *J* = 8.4 Hz, 2H), 6.51 (d, *J* = 5.4 Hz, 1H), 3.95 (s, 3H), 3.94 (s, 3H); ^13^C NMR (150 MHz, DMSO-*d*_6_) *δ* 163.3, 162.3 (d, *J* = 244.2 Hz), 159.7, 157.7, 154.4, 152.5, 150.3, 149.3, 148.8, 146.5, 135.2, 130.0 (d, *J* = 2.7 Hz), 128.6 (d, *J* = 8.1 Hz), 122.6, 121.3, 120.1, 115.7 (d, *J* = 21.7 Hz), 115.2, 107.8, 103.3, 99.0, 55.7, 55.6; ESI-MS: *m*/*z* 502.3 [M + H]^+^.

##### N-(4-((6,7-Dimethoxyquinolin-4-yl)oxy)phenyl)-5-phenylthiazole-2-carboxamide (51g)

Pale yellow solid; yield 75% (two steps); m.p. 260–262 °C. HPLC purity 96.2%. IR (KBr) *ν*_max_/cm^–^^1^ 3338, 2923, 1661, 1590, 1527, 1506, 1478, 1449, 1431, 1420, 1343, 1303, 1240, 1219, 1198, 1178, 1158, 1078, 991, 853, 825. ^1^H NMR (600 MHz, DMSO-*d*_6_) *δ* 10.98 (s, 1H), 8.53 (s, 1H), 8.48 (d, *J* = 4.8 Hz, 1H), 8.03 (d, *J* = 8.4 Hz, 2H), 7.83 (d, *J* = 7.2 Hz, 2H), 7.51–7.50 (m, 3H), 7.46–7.44 (m, 1H), 7.40 (s, 1H), 7.30 (d, *J* = 8.4 Hz, 2H), 6.49 (d, *J* = 4.8 Hz, 1H), 3.95 (s, 3H), 3.94 (s, 3H); ^13^C NMR (150 MHz, DMSO-*d*_6_) *δ* 162.1, 159.8, 157.6, 152.5, 150.1, 149.3, 148.8, 146.4, 144.5, 140.0, 135.4, 130.1, 129.4, 126.9, 123.1, 122.4, 121.3, 115.1, 107.8, 103.2, 99.0, 55.7, 55.6; ESI-MS: *m*/*z* 484.3 [M + H]^+^.

##### N-(4-((6,7-Dimethoxyquinolin-4-yl)oxy)phenyl)-5-(4-fluorophenyl)thiazole-2-carboxamide (51h)

Pale yellow solid; yield 59% (two steps); m.p. 252–254 °C. HPLC purity 96.2%. IR (KBr) *ν*_max_/cm^–^^1^ 3336, 2938, 1678, 1622, 1601, 1532, 1508, 1494, 1478, 1428, 1348, 1305, 1273, 1246, 1233, 1214, 1168, 1078, 992, 836, 816. ^1^H NMR (600 MHz, DMSO-*d*_6_) *δ* 10.99 (s, 1H), 8.50 (s, 1H), 8.48 (d, *J* = 4.8 Hz, 1H), 8.02 (d, *J* = 8.4 Hz, 2H), 7.89 (dd, *J* = 5.4, 8.4 Hz, 2H), 7.51 (s, 1H), 7.39 (s, 1H), 7.37–7.34 (m, 2H), 7.30 (d, *J* = 9.0 Hz, 2H), 6.48 (d, *J* = 4.8 Hz, 1H), 3.94 (s, 3H), 3.93 (s, 3H); ^13^C NMR (150 MHz, DMSO-*d*_6_) *δ* 162.6 (d, *J* = 246.3 Hz), 162.2, 159.8, 157.6, 152.5, 150.1, 149.3, 148.8, 146.4, 143.4, 140.1, 135.5, 129.2 (d, *J* = 8.2 Hz), 126.7 (d, *J* = 2.5 Hz), 122.4, 121.3, 116.4 (d, *J* = 21.9 Hz), 115.1, 107.8, 103.2, 99.0, 55.7, 55.6; ESI-MS: *m*/*z* 502.2 [M + H]^+^.

##### N-(4-((6,7-Dimethoxyquinolin-4-yl)oxy)-2-fluorophenyl)-4-phenylthiazole-2-carboxamide (51i)

Pale yellow solid; yield 73% (two steps); m.p. 112–114 °C. HPLC purity 95.5%. IR (KBr) *ν*_max_/cm^–^^1^ 3378, 2926, 1683, 1600, 1531, 1505, 1478, 1430, 1350, 1303, 1253, 1211, 1167, 1144, 1076, 995, 958, 853, 823, 752. ^1^H NMR (400 MHz, DMSO-*d*_6_) *δ* 10.54 (s, 1H), 8.55 (d, *J* = 5.2 Hz, 1H), 8.52 (s, 1H), 8.14 (d, *J* = 7.6 Hz, 2H), 7.85–7.81 (m, 1H), 7.53–7.48 (m, 3H), 7.45–7.40 (m, 3H), 7.19 (dd, *J* = 2.8, 8.8 Hz, 1H), 6.65 (d, *J* = 5.2 Hz, 1H), 3.95 (s, 3H), 3.93 (s, 3H); ^13^C NMR (100 MHz, DMSO-*d*_6_) *δ* 162.4, 159.1, 157.9, 156.1 (d, *J* = 248.4 Hz), 155.5, 152.7, 152.6 (d, *J* = 10.5 Hz), 149.5, 148.9, 146.5, 133.3, 128.9, 128.7, 127.9, 126.4, 121.9 (d, *J* = 12.3 Hz), 120.5, 116.7 (d, *J* = 3.3 Hz), 115.3, 109.2 (d, *J* = 22.7 Hz), 107.8, 104.2, 99.0, 55.8, 55.7; ESI-MS: *m*/*z* 502.1 [M + H]^+^.

##### N-(4-((6,7-Dimethoxyquinolin-4-yl)oxy)-3-fluorophenyl)-4-phenylthiazole-2-carboxamide (51j)

Pale yellow solid; yield 65% (two steps); m.p. 90–92 °C. HPLC purity 97.1%. IR (KBr) *ν*_max_/cm^–^^1^ 3434, 2928, 1668, 1623, 1598, 1559, 1538, 1509, 1480, 1431, 1349, 1305, 1251, 1212, 1165, 1076, 994, 853, 833, 754. ^1^H NMR (400 MHz, DMSO-*d*_6_) *δ* 10.94 (s, 1H), 8.53 (s, 1H), 8.50 (d, *J* = 5.2 Hz, 1H), 8.17 (d, *J* = 7.6 Hz, 2H), 8.09 (d, *J* = 12.8 Hz, 1H), 7.87 (d, *J* = 9.6 Hz, 1H), 7.54–7.50 (m, 4H), 7.44–7.40 (m, 2H), 6.51 (d, *J* = 5.2 Hz, 1H), 3.95 (s, 6H); ^13^C NMR (100 MHz, DMSO-*d*_6_) *δ* 162.8, 162.3, 159.2, 158.0, 155.6, 153.3 (d, *J* = 244.3 Hz), 152.7, 149.5, 148.9, 146.4, 136.8 (d, *J* = 9.6 Hz), 136.6 (d, *J* = 12.0 Hz), 133.3, 128.8, 126.5, 124.0, 120.7, 117.7, 114.6, 109.7 (d, *J* = 23.2 Hz), 107.8, 102.2, 98.9, 55.7; ESI-MS: *m*/*z* 502.3 [M + H]^+^.

##### N-(2-Chloro-4-((6,7-dimethoxyquinolin-4-yl)oxy)phenyl)-4-phenylthiazole-2-carboxamide (51k)

Yellow solid; yield 53% (two steps); m.p. 201–203 °C. HPLC purity 96.8%. IR (KBr) *ν*_max_/cm^–^^1^ 3435, 2924, 1690, 1586, 1531, 1505, 1479, 1430, 1349, 1304, 1248, 1210, 1194, 1165, 1090, 995, 915, 874, 819, 749. ^1^H NMR (600 MHz, DMSO-*d*_6_) *δ* 10.41 (s, 1H), 8.56 (s, 1H), 8.55 (d, *J* = 4.8 Hz, 1H), 8.12 (d, *J* = 7.2 Hz, 2H), 8.02 (d, *J* = 8.4 Hz, 1H), 7.63 (d, *J* = 3.0 Hz, 1H), 7.53–7.51 (m, 2H), 7.49 (s, 1H), 7.44–7.41 (m, 2H), 7.36 (dd, *J* = 3.0, 9.0 Hz, 1H), 6.64 (d, *J* = 5.4 Hz, 1H), 3.95 (s, 3H), 3.93 (s, 3H); ^13^C NMR (150 MHz, DMSO-*d*_6_) *δ* 162.3, 158.9, 157.5, 155.4, 152.6, 152.1, 149.5, 148.9, 146.6, 133.1, 131.2, 128.9, 128.7, 128.5, 127.1, 126.3, 121.9, 120.8, 120.1, 115.3, 107.9, 104.1, 99.0, 55.7; ESI-MS: *m*/*z* 518.1 [M + H]^+^.

##### N-(3-Chloro-4-((6,7-dimethoxyquinolin-4-yl)oxy)phenyl)-4-phenylthiazole-2-carboxamide (51l)

Pale yellow solid; yield 58% (two steps); m.p. 146–148 °C. HPLC purity 97.0%. IR (KBr) *ν*_max_/cm^–^^1^ 3375, 2926, 1668, 1596, 1530, 1505, 1477, 1431, 1385, 1348, 1305, 1251, 1211, 1165, 1084, 1060, 994, 852, 753. ^1^H NMR (600 MHz, DMSO-*d*_6_) *δ* 10.93 (s, 1H), 8.54 (s, 1H), 8.49 (d, *J* = 4.8 Hz, 1H), 8.29 (d, *J* = 2.4 Hz, 1H), 8.18 (d, *J* = 7.2 Hz, 2H), 8.03 (dd, *J* = 3.0, 9.0 Hz, 1H), 7.55 (s, 1H), 7.53–7.51 (m, 3H), 7.43 (d, *J* = 7.2 Hz, 1H), 7.42 (s, 1H), 6.41 (d, *J* = 4.8 Hz, 1H), 3.95 (s, 6H); ^13^C NMR (150 MHz, DMSO-*d*_6_) *δ* 162.8, 158.8, 158.0, 155.5, 152.6, 149.4, 148.8, 146.4, 145.4, 136.7, 133.3, 128.8, 128.7, 126.4, 125.7, 123.8, 122.4, 121.2, 120.6, 114.6, 107.8, 102.5, 98.9, 55.7; ESI-MS: *m*/*z* 518.1 [M + H]^+^.

##### N-(4-((6,7-Dimethoxyquinolin-4-yl)oxy)-2-methylphenyl)-4-phenylthiazole-2-carboxamide (51m)

Yellow solid; yield 46% (two steps); m.p. 209–211 °C. HPLC purity 95.2%. IR (KBr) *ν*_max_/cm^–^^1^ 3435, 2924, 1668, 1574, 1505, 1480, 1428, 1349, 1303, 1253, 1207, 1168, 1075, 996, 947, 886, 851, 818, 762. ^1^H NMR (600 MHz, DMSO-*d*_6_) *δ* 10.35 (s, 1H), 8.52–8.51 (m, 2H), 8.15 (d, *J* = 7.2 Hz, 2H), 7.65 (d, *J* = 8.4 Hz, 1H), 7.52–7.50 (m, 3H), 7.42 (d, *J* = 7.8 Hz, 1H), 7.41 (s, 1H), 7.26 (d, *J* = 1.8 Hz, 1H), 7.17 (dd, *J* = 2.4, 8.4 Hz, 1H), 6.54 (d, *J* = 5.4 Hz, 1H), 3.95 (s, 3H), 3.94 (s, 3H), 2.34 (s, 3H); ^13^C NMR (150 MHz, DMSO-*d*_6_) *δ* 163.0, 159.5, 157.7, 155.4, 152.6, 151.9, 149.3, 148.8, 146.5, 135.7, 133.3, 132.5, 128.8, 128.6, 127.5, 126.3, 122.4, 120.1, 118.4, 115.3, 107.8, 103.6, 99.0, 55.7, 55.6, 17.8; ESI-MS: *m*/*z* 498.2 [M + H]^+^.

##### N-(4-((6,7-Dimethoxyquinolin-4-yl)oxy)-3-methylphenyl)-4-phenylthiazole-2-carboxamide (51n)

Yellow solid; yield 58% (two steps); m.p. 136–138 °C. HPLC purity 98.3%. IR (KBr) *ν*_max_/cm^–^^1^ 3435, 2924, 1667, 1620, 1580, 1559, 1538, 1505, 1477, 1430, 1348, 1303, 1250, 1212, 1165, 1078, 993, 850, 826, 753. ^1^H NMR (600 MHz, DMSO-*d*_6_) *δ* 10.71 (s, 1H), 8.52 (s, 1H), 8.46 (d, *J* = 5.4 Hz, 1H), 8.18 (d, *J* = 7.8 Hz, 2H), 7.94 (s, 1H), 7.88 (dd, *J* = 2.4, 9.0 Hz, 1H), 7.58 (s, 1H), 7.53–7.50 (m, 2H), 7.43 (d, *J* = 7.2 Hz, 1H), 7.41 (s, 1H), 7.25 (d, *J* = 8.4 Hz, 1H), 6.34 (d, *J* = 5.4 Hz, 1H), 3.95 (s, 6H), 2.15 (s, 3H); ^13^C NMR (150 MHz, DMSO-*d*_6_) *δ* 163.2, 159.4, 157.7, 155.4, 152.5, 149.3, 148.9, 148.1, 146.3, 135.5, 133.3, 130.4, 128.8, 128.6, 126.4, 123.9, 121.9, 120.3, 120.2, 114.8, 107.8, 102.2, 99.1, 55.7, 15.7; ESI-MS: *m*/*z* 498.0 [M + H]^+^.

##### N-(4-((6,7-Dimethoxyquinazolin-4-yl)oxy)-3-fluorophenyl)-4-phenylthiazole-2-carboxamide (51o)

Pale yellow solid; yield 74% (two steps); m.p. 115–117 °C. HPLC purity 97.6%. IR (KBr) *ν*_max_/cm^–^^1^ 3373, 2925, 1672, 1619, 1586, 1537, 1509, 1466, 1446, 1418, 1376, 1307, 1237, 1209, 1133, 1077, 994, 850, 753. ^1^H NMR (400 MHz, DMSO-*d*_6_) *δ* 10.90 (s, 1H), 8.58 (s, 1H), 8.53 (s, 1H), 8.17 (d, *J* = 7.2 Hz, 2H), 8.03 (dt, *J* = 2.4, 12.8 Hz, 1H), 7.83 (d, *J* = 8.4 Hz, 1H), 7.57 (s, 1H), 7.55–7.50 (m, 3H), 7.44–7.40 (m, 2H), 3.99 (s, 3H), 3.98 (s, 3H); ^13^C NMR (100 MHz, DMSO-*d*_6_) *δ* 163.9, 162.9, 157.9, 155.9, 155.6, 153.4 (d, *J* = 243.5 Hz), 152.1, 150.3, 149.0, 136.7 (d, *J* = 9.9 Hz), 135.4 (d, *J* = 12.7 Hz), 133.3, 128.8, 128.7, 126.5, 124.4, 120.6, 117.2 (d, *J* = 2.7 Hz), 109.3, 109.1, 106.7, 100.5, 56.2, 56.0; ESI-MS: *m*/*z* 503.2 [M + H]^+^.

##### N-(4-((6,7-Dimethoxyquinazolin-4-yl)oxy)-3-fluorophenyl)-4-(4-fluorophenyl)thiazole-2-carboxamide (51p)

White solid; yield 69% (two steps); m.p. 188–190 °C. HPLC purity 96.2%. IR (KBr) *ν*_max_/cm^–^^1^ 3434, 3091, 1683, 1621, 1579, 1559, 1539, 1506, 1447, 1419, 1381, 1239, 1215, 1079, 995, 913, 855. ^1^H NMR (400 MHz, DMSO-*d*_6_) *δ* 10.90 (s, 1H), 8.58 (s, 1H), 8.51 (s, 1H), 8.21 (dd, *J* = 5.6, 8.8 Hz, 2H), 8.03–7.99 (m, 1H), 7.81 (d, *J* = 8.8 Hz, 1H), 7.57 (s, 1H), 7.54–7.50 (m, 1H), 7.40 (s, 1H), 7.38–7.34 (m, 2H), 3.99 (s, 3H), 3.98 (s, 3H); ^13^C NMR (100 MHz, DMSO-*d*_6_) *δ* 164.0, 163.0, 162.4 (d, *J* = 244.2 Hz), 157.9, 155.9, 154.5, 153.4 (d, *J* = 243.6 Hz), 152.1, 150.3, 149.0, 136.7 (d, *J* = 10.1 Hz), 135.5 (d, *J* = 12.8 Hz), 129.9 (d, *J* = 2.7 Hz), 128.6 (d, *J* = 8.3 Hz), 124.4, 120.4, 117.2 (d, *J* = 3.0 Hz), 115.7 (d, *J* = 21.7 Hz), 109.3, 109.1, 106.8, 100.5, 56.2, 56.0; ESI-MS: *m*/*z* 521.1 [M + H]^+^.

##### N-(3-Fluoro-4-(thieno[3,2-b]pyridin-7-yloxy)phenyl)-4-phenylthiazole-2-carboxamide (51q)

Pale yellow solid; yield 63% (two steps); m.p. 251–253 °C. HPLC purity 96.8%. IR (KBr) *ν*_max_/cm^–^^1^ 3453, 2922, 1668, 1606, 1585, 1547, 1503, 1484, 1452, 1440, 1407, 1381, 1296, 1267, 1202, 1074, 1025, 816, 754. ^1^H NMR (400 MHz, DMSO-*d*_6_) *δ* 10.95 (s, 1H), 8.55 (d, *J* = 5.2 Hz, 1H), 8.54 (s, 1H), 8.19–8.16 (m, 3H), 8.10 (dt, *J* = 2.4, 12.8 Hz, 1H), 7.89–7.85 (m, 1H), 7.62 (d, *J* = 5.2 Hz, 1H), 7.58–7.50 (m, 3H), 7.44–7.42 (m, 1H), 6.70 (d, *J* = 5.2 Hz, 1H); ^13^C NMR (100 MHz, DMSO-*d*_6_) *δ* 162.8, 159.1, 158.9, 158.0, 155.6, 153.3 (d, *J* = 244.1 Hz), 149.6, 137.1 (d, *J* = 10.1 Hz), 135.9 (d, *J* = 12.2 Hz), 133.3, 132.2, 128.8, 128.7, 126.4, 125.0, 123.9, 120.8, 120.7, 117.6 (d, *J* = 3.3 Hz), 109.5 (d, *J* = 23.0 Hz), 103.3; ESI-MS: *m*/*z* 448.0 [M + H]^+^.

##### N-(3-Fluoro-4-(thieno[3,2-b]pyridin-7-yloxy)phenyl)-4-(4-fluorophenyl)thiazole-2-carboxamide (51r)

Pale yellow solid; yield 58% (two steps); m.p. 223–225 °C. HPLC purity 96.4%. IR (KBr) *ν*_max_/cm^–^^1^ 3469, 2923, 1668, 1604, 1548, 1527, 1504, 1484, 1449, 1406, 1382, 1298, 1269, 1233, 1202, 1156, 1130, 1027, 844, 812, 767. ^1^H NMR (400 MHz, DMSO-*d*_6_) *δ* 10.94 (s, 1H), 8.54 (d, *J* = 5.2 Hz, 1H), 8.50 (s, 1H), 8.21 (dd, *J* = 5.6, 8.8 Hz, 2H), 8.17 (d, *J* = 5.6 Hz, 1H), 8.11–8.07 (m, 1H), 7.88–7.84 (m, 1H), 7.61 (d, *J* = 5.2 Hz, 1H), 7.57–7.53 (m, 1H), 7.37–7.32 (m, 2H), 6.69 (d, *J* = 5.2 Hz, 1H); ^13^C NMR (100 MHz, DMSO-*d*_6_) *δ* 162.8, 162.3 (d, *J* = 244.2 Hz), 159.1, 158.9, 157.9, 154.5, 153.3 (d, *J* = 244.7 Hz), 149.6, 137.1 (d, *J* = 10.1 Hz), 135.9 (d, *J* = 12.2 Hz), 132.2, 129.9 (d, *J* = 3.0 Hz), 128.6 (d, *J* = 8.1 Hz), 124.9, 123.9, 120.8, 120.4, 117.5 (d, *J* = 3.0 Hz), 115.7 (d, *J* = 21.6 Hz), 109.5 (d, *J* = 22.6 Hz), 103.4; ESI-MS: *m*/*z* 466.3 [M + H]^+^.

##### N-(3-Fluoro-4-(thieno[3,2-d]pyrimidin-4-yloxy)phenyl)-4-phenylthiazole-2-carboxamide (51s)

Pale yellow solid; yield 67% (two steps); m.p. 95–97 °C. HPLC purity 95.2%. IR (KBr) *ν*_max_/cm^–^^1^ 3434, 2920, 1683, 1608, 1577, 1559, 1529, 1506, 1466, 1439, 1338, 1332, 1294, 1195, 1078, 1033, 870, 799, 752. ^1^H NMR (400 MHz, DMSO-*d*_6_) *δ* 10.92 (s, 1H), 8.75 (s, 1H), 8.53–8.51 (m, 2H), 8.17 (d, *J* = 7.6 Hz, 2H), 8.04 (d, *J* = 12.0 Hz, 1H), 7.83 (d, *J* = 8.8 Hz, 1H), 7.72 (d, *J* = 5.6 Hz, 1H), 7.59–7.50 (m, 3H), 7.44–7.40 (m, 1H); ^13^C NMR (100 MHz, DMSO-*d*_6_) *δ* 163.3, 162.9, 162.8, 158.0, 155.6, 154.1, 153.3 (d, *J* = 243.9 Hz), 137.7, 137.1 (d, *J* = 10.0 Hz), 134.7 (d, *J* = 12.8 Hz), 133.3, 128.8, 128.7, 126.5, 124.3, 120.6, 117.3 (d, *J* = 2.5 Hz), 117.2 (d, *J* = 3.0 Hz), 116.2, 109.2 (d, *J* = 23.2 Hz); ESI-MS: *m*/*z* 449.1 [M + H]^+^.

##### N-(3-Fluoro-4-(thieno[3,2-d]pyrimidin-4-yloxy)phenyl)-4-(4-fluorophenyl)thiazole-2-carboxamide (51t)

Pale yellow solid; yield 75% (two steps); m.p. 146–148 °C. HPLC purity 97.6%. IR (KBr) *ν*_max_/cm^–^^1^ 3373, 3098, 1678, 1607, 1578, 1530, 1509, 1484, 1467, 1441, 1392, 1334, 1294, 1224, 1196, 1079, 837, 795. ^1^H NMR (400 MHz, DMSO-*d*_6_) *δ* 10.91 (s, 1H), 8.74 (s, 1H), 8.52–8.51 (m, 2H), 8.23–8.19 (m, 2H), 8.02 (d, *J* = 12.4 Hz, 1H), 7.81 (d, *J* = 8.8 Hz, 1H), 7.71 (d, *J* = 5.6 Hz, 1H), 7.58–7.54 (m, 1H), 7.38–7.33 (m, 2H); ^13^C NMR (100 MHz, DMSO-*d*_6_) *δ* 163.3, 162.9, 162.8, 162.3 (d, *J* = 244.2 Hz), 157.9, 154.5, 154.1, 153.3 (d, *J* = 243.8 Hz), 137.7, 137.1 (d, *J* = 10.0 Hz), 134.7 (d, *J* = 12.6 Hz), 129.9 (d, *J* = 2.5 Hz), 128.6 (d, *J* = 8.5 Hz), 124.4, 124.3, 120.4, 117.3 (d, *J* = 3.3 Hz), 116.2, 115.7 (d, *J* = 21.4 Hz), 109.2 (d, *J* = 22.9 Hz); ESI-MS: *m*/*z* 467.0 [M + H]^+^.

##### N-(3-Fluoro-4-(thieno[2,3-d]pyrimidin-4-yloxy)phenyl)-4-phenylthiazole-2-carboxamide (51u)

Yellow solid; yield 49% (two steps); m.p. 78–80 °C. HPLC purity 95.8%. IR (KBr) *ν*_max_/cm^–^^1^ 3453, 1719, 1683, 1626, 1559, 1531, 1505, 1477, 1430, 1363, 1335, 1192, 1075, 966, 749. ^1^H NMR (400 MHz, DMSO-*d*_6_) *δ* 10.91 (s, 1H), 8.67 (s, 1H), 8.53 (s, 1H), 8.17 (dd, *J* = 1.6, 8.4 Hz, 2H), 8.05–8.01 (m, 2H), 7.84–7.80 (m, 1H), 7.72 (d, *J* = 6.0 Hz, 1H), 7.56–7.49 (m, 4H); ^13^C NMR (100 MHz, DMSO-*d*_6_) *δ* 169.3, 162.9, 162.3, 157.9, 155.6, 153.3 (d, *J* = 244.0 Hz), 152.9, 136.9 (d, *J* = 10.2 Hz), 135.0 (d, *J* = 12.4 Hz), 133.3, 128.8, 128.7, 128.0, 126.5, 124.3, 120.6, 118.2, 118.0, 117.3 (d, *J* = 3.1 Hz), 109.3 (d, *J* = 23.1 Hz); ESI-MS: *m*/*z* 449.1 [M + H]^+^.

##### N-(3-Fluoro-4-(thieno[2,3-d]pyrimidin-4-yloxy)phenyl)-4-(4-fluorophenyl)thiazole-2-carboxamide (51v)

Yellow solid; yield 62% (two steps); m.p. 154–156 °C. HPLC purity 96.2%. IR (KBr) *ν*_max_/cm^–^^1^ 3452, 3100, 1677, 1607, 1584, 1530, 1506, 1484, 1430, 1364, 1343, 1294, 1223, 1192, 1159, 1070, 968, 839, 763, 707. ^1^H NMR (400 MHz, DMSO-*d*_6_) *δ* 10.90 (s, 1H), 8.66 (s, 1H), 8.50 (s, 1H), 8.21 (dd, *J* = 5.6, 8.4 Hz, 2H), 8.04–8.00 (m, 2H), 7.83–7.79 (m, 1H), 7.71 (d, *J* = 6.0 Hz, 1H), 7.55–7.33 (m, 3H); ^13^C NMR (100 MHz, DMSO-*d*_6_) *δ* 169.2, 162.9, 162.4 (d, *J* = 244.2 Hz), 162.3, 157.9, 154.5, 153.3 (d, *J* = 243.5 Hz), 152.8, 136.9 (d, *J* = 10.1 Hz), 135.0 (d, *J* = 12.5 Hz), 129.9 (d, *J* = 2.9 Hz), 128.6 (d, *J* = 8.3 Hz), 127.9, 124.3, 120.4, 118.2, 118.0, 117.3 (d, *J* = 3.4 Hz), 115.7 (d, *J* = 21.6 Hz), 109.3 (d, *J* = 23.2 Hz); ESI-MS: *m*/*z* 467.4 [M + H]^+^.

##### N-(4-((2-Chloropyridin-4-yl)oxy)-3-fluorophenyl)-4-phenylthiazole-2-carboxamide (51w)

Yellow solid; yield 64% (two steps); m.p. 205–207 °C. HPLC purity 97.0%. IR (KBr) *ν*_max_/cm^–^^1^ 3436, 3105, 2917, 2850, 1672, 1606, 1582, 1560, 1533, 1509, 1484, 1459, 1440, 1393, 1303, 1266, 1235, 1194, 1071, 920, 758. ^1^H NMR (400 MHz, DMSO-*d*_6_) *δ* 10.92 (s, 1H), 8.52 (s, 1H), 8.32 (d, *J* = 5.6 Hz, 1H), 8.16 (d, *J* = 6.8 Hz, 2H), 8.10–8.06 (m, 1H), 7.87–7.83 (m, 1H), 7.53–7.49 (m, 2H), 7.47–7.39 (m, 2H), 7.10 (d, *J* = 2.4 Hz, 1H), 7.00 (dd, *J* = 2.4, 6.0 Hz, 1H); ^13^C NMR (100 MHz, DMSO-*d*_6_) *δ* 166.1, 163.2, 158.4, 156.0, 153.5 (d, *J* = 244.2 Hz), 152.1, 151.9, 137.6 (d, *J* = 9.8 Hz), 136.1 (d, *J* = 12.4 Hz), 133.8, 129.3, 129.2, 126.9, 124.1, 121.1, 118.1 (d, *J* = 3.0 Hz), 111.5, 111.2, 110.1 (d, *J* = 22.9 Hz); ESI-MS: *m*/*z* 426.1 [M + H]^+^.

##### N-(4-((2-Chloropyridin-4-yl)oxy)-3-fluorophenyl)-4-(4-methylphenyl)thiazole-2-carboxamide (51x)

Pale yellow solid; yield 48% (two steps); m.p. 157–159 °C. HPLC purity 98.1%. IR (KBr) *ν*_max_/cm^–^^1^ 3435, 3103, 2923, 2854, 1676, 1604, 1591, 1566, 1531, 1508, 1476, 1442, 1387, 1308, 1267, 1229, 1199, 1065, 922, 822. ^1^H NMR (400 MHz, DMSO-*d*_6_) *δ* 10.91 (s, 1H), 8.45 (s, 1H), 8.33 (d, *J* = 5.6 Hz, 1H), 8.10–8.06 (m, 3H), 7.86 (d, *J* = 9.2 Hz, 1H), 7.50–7.46 (m, 1H), 7.33 (d, *J* = 8.0 Hz, 2H), 7.12–7.11 (m, 1H), 7.02 (d, *J* = 4.4 Hz, 1H), 2.37 (s, 3H); ^13^C NMR (100 MHz, DMSO-*d*_6_) *δ* 165.6, 162.6, 158.0, 155.7, 153.0 (d, *J* = 244.6 Hz), 151.6, 151.4, 138.1, 137.1 (d, *J* = 10.2 Hz), 135.6 (d, *J* = 12.3 Hz), 130.6, 129.3, 126.4, 123.6, 119.8, 117.7 (d, *J* = 3.0 Hz), 111.0, 110.7, 109.6 (d, *J* = 23.0 Hz), 20.8; ESI-MS: *m*/*z* 440.2 [M + H]^+^.

##### N-(4-((2-Chloropyridin-4-yl)oxy)-3-fluorophenyl)-4-(4-methoxyphenyl)thiazole-2-carboxamide (51y)

Pale yellow solid; yield 53% (two steps); m.p. 191–193 °C. HPLC purity 96.4%. IR (KBr) *ν*_max_/cm^–^^1^ 3435, 3105, 2924, 1676, 1612, 1591, 1566, 1531, 1508, 1476, 1438, 1387, 1308, 1267, 1248, 1199, 1177, 1068, 1028, 923, 827. ^1^H NMR (400 MHz, DMSO-*d*_6_) *δ* 10.88 (s, 1H), 8.35 (s, 1H), 8.32 (d, *J* = 5.6 Hz, 1H), 8.09–8.05 (m, 3H), 7.84 (d, *J* = 8.8 Hz, 1H), 7.48–7.44 (m, 1H), 7.10 (d, *J* = 2.4 Hz, 1H), 7.06 (d, *J* = 8.4 Hz, 2H), 7.00 (dd, *J* = 2.4, 6.0 Hz, 1H), 3.82 (s, 3H); ^13^C NMR (100 MHz, DMSO-*d*_6_) *δ* 165.6, 162.5, 159.6, 158.0, 155.6, 153.0 (d, *J* = 244.2 Hz), 151.6, 151.4, 137.1 (d, *J* = 9.8 Hz), 135.6 (d, *J* = 12.2 Hz), 127.9, 126.1, 123.6, 118.7, 117.6 (d, *J* = 3.5 Hz), 114.1, 111.0, 110.7, 109.6 (d, *J* = 23.0 Hz), 55.2; ESI-MS: *m*/*z* 456.1 [M + H]^+^.

##### N-(4-((2-Chloropyridin-4-yl)oxy)-3-fluorophenyl)-4-(3,4-dimethoxyphenyl)thiazole-2-carboxamide (51z)

Yellow solid; yield 65% (two steps); m.p. 120–122 °C. HPLC purity 95.9%. IR (KBr) *ν*_max_/cm^–^^1^ 3377, 2928, 1678, 1589, 1559, 1537, 1506, 1491, 1460, 1431, 1299, 1264, 1235, 1195, 1161, 1146, 1120, 1073, 1027, 922, 758. ^1^H NMR (400 MHz, DMSO-*d*_6_) *δ* 10.89 (s, 1H), 8.40 (s, 1H), 8.32 (d, *J* = 5.6 Hz, 1H), 8.08–8.05 (m, 1H), 7.83 (d, *J* = 8.8 Hz, 1H), 7.73–7.70 (m, 2H), 7.48–7.44 (m, 1H), 7.09–7.05 (m, 2H), 7.00 (d, *J* = 4.8 Hz, 1H), 3.89 (s, 3H), 3.82 (s, 3H); ^13^C NMR (100 MHz, DMSO-*d*_6_) *δ* 165.6, 162.4, 158.0, 155.7, 153.0 (d, *J* = 244.3 Hz), 151.7, 151.4, 149.4, 148.9, 137.1 (d, *J* = 9.9 Hz), 135.6 (d, *J* = 12.1 Hz), 126.2, 123.6, 119.3, 118.9, 117.7 (d, *J* = 3.3 Hz), 111.8, 111.0, 110.7, 110.4, 109.7 (d, *J* = 22.4 Hz), 55.7, 55.5; ESI-MS: *m*/*z* 486.1 [M + H]^+^.

##### N-(4-((2-Chloropyridin-4-yl)oxy)-3-fluorophenyl)-4-(4-fluorophenyl)thiazole-2-carboxamide (51aa)

White solid; yield 70% (two steps); m.p. 150–152 °C. HPLC purity 96.5%. IR (KBr) *ν*_max_/cm^–^^1^ 3434, 3090, 2923, 1674, 1605, 1585, 1560, 1536, 1508, 1485, 1445, 1302, 1266, 1233, 1200, 1162, 1121, 1072, 920, 840. ^1^H NMR (400 MHz, DMSO-*d*_6_) *δ* 10.91 (s, 1H), 8.50 (s, 1H), 8.32 (d, *J* = 6.0 Hz, 1H), 8.20 (dd, *J* = 5.6, 8.8 Hz, 2H), 8.06 (dd, *J* = 2.4, 13.2 Hz, 1H), 7.84–7.81 (m, 1H), 7.49–7.32 (m, 3H), 7.09 (d, *J* = 2.4 Hz, 1H), 7.00 (dd, *J* = 2.4, 5.6 Hz, 1H); ^13^C NMR (100 MHz, DMSO-*d*_6_) *δ* 165.6, 162.8, 162.3 (d, *J* = 244.2 Hz), 157.9, 154.5, 153.0 (d, *J* = 244.6 Hz), 151.6, 151.4, 137.1 (d, *J* = 9.8 Hz), 135.6 (d, *J* = 12.3 Hz), 129.9 (d, *J* = 2.9 Hz), 128.6 (d, *J* = 8.1 Hz), 123.6, 120.4, 117.6 (d, *J* = 3.4 Hz), 115.7 (d, *J* = 21.7 Hz), 111.0, 110.7, 109.6 (d, *J* = 23.0 Hz); ESI-MS: *m*/*z* 444.3 [M + H]^+^.

##### N-(4-((2-Chloropyridin-4-yl)oxy)-3-fluorophenyl)-4-(3-fluorophenyl)thiazole-2-carboxamide (51ab)

Yellow solid; yield 46% (two steps); m.p. 189–191 °C. HPLC purity 97.3%. IR (KBr) *ν*_max_/cm^–^^1^ 3327, 2917, 1668, 1607, 1582, 1561, 1537, 1509, 1480, 1456, 1435, 1407, 1393, 1303, 1268, 1235, 1197, 1176, 1117, 1071, 919, 771. ^1^H NMR (400 MHz, DMSO-*d*_6_) *δ* 10.89 (s, 1H), 8.62 (s, 1H), 8.32 (d, *J* = 5.6 Hz, 1H), 8.08–8.04 (m, 2H), 7.99 (d, *J* = 8.0 Hz, 1H), 7.83 (d, *J* = 8.4 Hz, 1H), 7.57–7.51 (m, 1H), 7.49–7.44 (m, 1H), 7.26–7.21 (m, 1H), 7.09 (d, *J* = 2.0 Hz, 1H), 7.00 (dd, *J* = 2.4, 6.0 Hz, 1H); ^13^C NMR (100 MHz, DMSO-*d*_6_) *δ* 165.6, 162.8, 162.6 (d, *J* = 241.4 Hz), 157.8, 154.1 (d, *J* = 2.9 Hz), 153.0 (d, *J* = 244.5 Hz), 151.6, 151.4, 137.1 (d, *J* = 10.0 Hz), 135.7 (d, *J* = 10.8 Hz), 135.6 (d, *J* = 6.7 Hz), 130.8 (d, *J* = 8.5 Hz), 123.6, 122.4 (d, *J* = 2.1 Hz), 121.8, 117.7 (d, *J* = 3.2 Hz), 115.3 (d, *J* = 21.0 Hz), 113.2 (d, *J* = 23.0 Hz), 111.0, 110.7, 109.6 (d, *J* = 22.6 Hz); ESI-MS: *m*/*z* 444.1 [M + H]^+^.

##### N-(4-((2-Chloropyridin-4-yl)oxy)-3-fluorophenyl)-4-(2-fluorophenyl)thiazole-2-carboxamide (51ac)

Yellow solid; yield 52% (two steps); m.p. 168–170 °C. HPLC purity 98.6%. IR (KBr) *ν*_max_/cm^–^^1^ 3351, 2924, 2854, 1674, 1606, 1582, 1560, 1531, 1511, 1488, 1460, 1429, 1394, 1303, 1268, 1235, 1199, 1120, 1071, 920, 763. ^1^H NMR (400 MHz, DMSO-*d*_6_) *δ* 10.94 (s, 1H), 8.41 (td, *J* = 2.0, 8.0 Hz, 1H), 8.34 (d, *J* = 2.4 Hz, 1H), 8.32 (d, *J* = 6.0 Hz, 1H), 8.06 (dd, *J* = 2.0, 12.8 Hz, 1H), 7.85–7.81 (m, 1H), 7.51–7.44 (m, 2H), 7.40–7.34 (m, 2H), 7.09 (d, *J* = 2.4 Hz, 1H), 7.00 (dd, *J* = 2.0, 5.6 Hz, 1H); ^13^C NMR (100 MHz, DMSO-*d*_6_) *δ* 165.6, 162.3, 159.6 (d, *J* = 247.6 Hz), 157.8, 153.0 (d, *J* = 244.2 Hz), 151.6, 151.4, 149.1 (d, *J* = 1.8 Hz), 137.0 (d, *J* = 9.9 Hz), 135.7 (d, *J* = 12.2 Hz), 130.5 (d, *J* = 8.6 Hz), 130.3 (d, *J* = 2.8 Hz), 124.8 (d, *J* = 3.4 Hz), 124.7 (d, *J* = 13.5 Hz), 123.6, 121.0 (d, *J* = 11.4 Hz), 117.7 (d, *J* = 3.4 Hz), 116.2 (d, *J* = 21.7 Hz), 111.0, 110.7, 109.7 (d, *J* = 22.9 Hz); ESI-MS: *m*/*z* 444.0 [M + H]^+^.

##### 4-(4-Chlorophenyl)-N-(4-((2-chloropyridin-4-yl)oxy)-3-fluorophenyl)thiazole-2-carboxamide (51ad)

Pale yellow solid; yield 71% (two steps); m.p. 167–169 °C. HPLC purity 98.0%. IR (KBr) *ν*_max_/cm^–^^1^ 3435, 3105, 2924, 1674, 1591, 1566, 1538, 1508, 1476, 1441, 1388, 1308, 1268, 1230, 1200, 1096, 1073, 923, 829. ^1^H NMR (400 MHz, DMSO-*d*_6_) *δ* 10.91 (s, 1H), 8.56 (s, 1H), 8.32 (d, *J* = 5.6 Hz, 1H), 8.18 (d, *J* = 8.4 Hz, 2H), 8.06 (dd, *J* = 2.4, 12.8 Hz, 1H), 7.83 (d, *J* = 8.8 Hz, 1H), 7.57 (d, *J* = 8.4 Hz, 2H), 7.48–7.44 (m, 1H), 7.09 (d, *J* = 2.0 Hz, 1H), 7.00 (dd, *J* = 2.0, 5.6 Hz, 1H); ^13^C NMR (100 MHz, DMSO-*d*_6_) *δ* 165.6, 162.9, 157.8, 154.2, 153.0 (d, *J* = 244.9 Hz), 151.6, 151.4, 137.1 (d, *J* = 9.7 Hz), 135.6 (d, *J* = 12.3 Hz), 133.3, 132.1, 128.8, 128.1, 123.6, 121.2, 117.7 (d, *J* = 3.3 Hz), 111.0, 110.7, 109.6 (d, *J* = 22.7 Hz); ESI-MS: *m*/*z* 460.1 [M + H]^+^.

##### 4-(3-Chlorophenyl)-N-(4-((2-chloropyridin-4-yl)oxy)-3-fluorophenyl)thiazole-2-carboxamide (51ae)

Pale yellow solid; yield 65% (two steps); m.p. 171–173 °C. HPLC purity 94.8%. IR (KBr) *ν*_max_/cm^–^^1^ 3431, 2924, 1673, 1593, 1560, 1530, 1508, 1476, 1389, 1309, 1267, 1229, 1200, 1163, 1073, 923, 772. ^1^H NMR (400 MHz, DMSO-*d*_6_) *δ* 10.93 (s, 1H), 8.65 (s, 1H), 8.32 (d, *J* = 6.0 Hz, 1H), 8.29–8.27 (m, 1H), 8.10 (d, *J* = 7.2 Hz, 1H), 8.06 (dd, *J* = 2.4, 12.8 Hz, 1H), 7.85–7.81 (m, 1H), 7.55–7.51 (m, 1H), 7.49–7.45 (m, 2H), 7.10 (d, *J* = 2.4 Hz, 1H), 7.00 (dd, *J* = 2.4, 5.6 Hz, 1H); ^13^C NMR (100 MHz, DMSO-*d*_6_) *δ* 165.6, 163.0, 157.8, 153.8, 153.0 (d, *J* = 244.1 Hz), 151.6, 151.4, 137.0 (d, *J* = 9.7 Hz), 135.7 (d, *J* = 12.4 Hz), 135.2, 133.8, 130.6, 128.4, 126.1, 124.9, 123.6, 121.9, 117.8 (d, *J* = 3.4 Hz), 111.0, 110.8, 109.7 (d, *J* = 22.7 Hz); ESI-MS: *m*/*z* 460.0 [M + H]^+^.

##### 4-(4-Bromophenyl)-N-(4-((2-chloropyridin-4-yl)oxy)-3-fluorophenyl)thiazole-2-carboxamide (51af)

Pale yellow solid; yield: 78% (two steps); m.p. 192–194 °C. HPLC purity 95.9%. IR (KBr) *ν*_max_/cm^–^^1^ 3434, 3102, 2924, 1672, 1592, 1566, 1538, 1508, 1476, 1460, 1441, 1389, 1308, 1268, 1230, 1200, 1166, 1117, 1073, 923, 827. ^1^H NMR (400 MHz, DMSO-*d*_6_) *δ* 10.92 (s, 1H), 8.58 (s, 1H), 8.32 (d, *J* = 6.0 Hz, 1H), 8.12 (d, *J* = 8.4 Hz, 2H), 8.06 (dd, *J* = 2.4, 12.8 Hz, 1H), 7.85–7.81 (m, 1H), 7.71 (d, *J* = 8.8 Hz, 2H), 7.49–7.44 (m, 1H), 7.10 (d, *J* = 2.4 Hz, 1H), 7.00 (dd, *J* = 2.0, 5.6 Hz, 1H); ^13^C NMR (100 MHz, DMSO-*d*_6_) *δ* 165.6, 163.0, 157.8, 154.3, 153.0 (d, *J* = 244.6 Hz), 151.6, 151.4, 137.1 (d, *J* = 10.1 Hz), 135.7 (d, *J* = 12.0 Hz), 132.5, 131.7, 128.4, 123.6, 122.0, 121.3, 117.7 (d, *J* = 3.3 Hz), 111.0, 110.7, 109.6 (d, *J* = 23.0 Hz); ESI-MS: *m*/*z* 504.2 [M + H]^+^.

##### N-(4-((2-Chloropyridin-4-yl)oxy)-3-fluorophenyl)-4-(4(trifluoromethyl)phenyl)thiazole-2-carboxamide (51ag)

Yellow solid; yield 41% (two steps); m.p. 212–214 °C. HPLC purity 97.4%. IR (KBr) *ν*_max_/cm^–^^1^ 3413, 2924, 1672, 1606, 1591, 1560, 1538, 1509, 1476, 1450, 1409, 1388, 1325, 1307, 1270, 1231, 1201, 1166, 1119, 1071, 923, 833. ^1^H NMR (400 MHz, DMSO-*d*_6_) *δ* 10.96 (s, 1H), 8.73 (s, 1H), 8.37 (d, *J* = 8.4 Hz, 2H), 8.32 (d, *J* = 6.0 Hz, 1H), 8.06 (dd, *J* = 2.4, 13.2 Hz, 1H), 7.87 (d, *J* = 8.0 Hz, 2H), 7.84–7.81 (m, 1H), 7.49–7.44 (m, 1H), 7.09 (d, *J* = 2.0 Hz, 1H), 7.00 (dd, *J* = 2.4, 6.0 Hz, 1H); ^13^C NMR (100 MHz, DMSO-*d*_6_) *δ* 165.6, 163.3, 157.8, 153.8, 153.0 (d, *J* = 244.4 Hz), 151.6, 151.4, 137.1, 137.0, 135.8 (d, *J* = 12.3 Hz), 128.7 (q, *J* = 31.6 Hz), 127.0, 125.7 (q, *J* = 3.6 Hz), 124.2 (q, *J* = 270.4 Hz), 123.6, 123.0, 117.7 (d, *J* = 3.4 Hz), 111.0, 110.8, 109.7 (d, *J* = 23.0 Hz); ESI-MS: *m*/*z* 494.1 [M + H]^+^.

##### N-(4-((2-Chloropyridin-4-yl)oxy)-3-fluorophenyl)-4-(3,4-dichlorophenyl)thiazole-2-carboxamide (51ah)

Pale yellow solid; yield 68% (two steps); m.p. 195–197 °C. HPLC purity 96.5%. IR (KBr) *ν*_max_/cm^–^^1^ 3452, 3100, 2924, 2854, 1667, 1593, 1566, 1532, 1508, 1474, 1435, 1391, 1310, 1271, 1230, 1201, 1167, 1071, 923, 825. ^1^H NMR (400 MHz, DMSO-*d*_6_) *δ* 10.90 (s, 1H), 8.67 (s, 1H), 8.44 (d, *J* = 2.0 Hz, 1H), 8.32 (d, *J* = 6.0 Hz, 1H), 8.11 (dd, *J* = 2.0, 8.4 Hz, 1H), 8.04 (dd, *J* = 2.4, 13.2 Hz, 1H), 7.83–7.79 (m, 1H), 7.75 (d, *J* = 8.4 Hz, 1H), 7.48–7.44 (m, 1H), 7.09 (d, *J* = 2.4 Hz, 1H), 7.00 (dd, *J* = 2.0, 5.6 Hz, 1H); ^13^C NMR (100 MHz, DMSO-*d*_6_) *δ* 165.6, 163.1, 157.7, 153.0 (d, *J* = 244.2 Hz), 152.8, 151.6, 151.4, 137.0 (d, *J* = 10.0 Hz), 135.7 (d, *J* = 12.3 Hz), 133.8, 131.8, 131.1, 131.0, 128.1, 126.3, 123.6, 122.4, 117.7 (d, *J* = 3.4 Hz), 111.0, 110.7, 109.7 (d, *J* = 23.0 Hz); ESI-MS: *m*/*z* 493.9 [M + H]^+^.

##### N-(4-((2-Chloropyridin-4-yl)oxy)-3-fluorophenyl)-4-(naphthalen-2-yl)thiazole-2-carboxamide (51ai)

Pale yellow solid; yield 86% (two steps); m.p. 182–184 °C. HPLC purity 95.9%. IR (KBr) *ν*_max_/cm^–^^1^ 3435, 2923, 1673, 1589, 1559, 1549, 1531, 1506, 1476, 1440, 1387, 1308, 1265, 1230, 1200, 1167, 1071, 924, 817. ^1^H NMR (400 MHz, DMSO-*d*_6_) *δ* 11.00 (s, 1H), 8.73 (s, 1H), 8.66 (s, 1H), 8.33 (d, *J* = 5.6 Hz, 1H), 8.29 (d, *J* = 8.4 Hz, 1H), 8.13–7.96 (m, 4H), 7.88 (d, *J* = 8.4 Hz, 1H), 7.60–7.47 (m, 3H), 7.12 (d, *J* = 1.6 Hz, 1H), 7.02 (dd, *J* = 2.4, 5.6 Hz, 1H); ^13^C NMR (100 MHz, DMSO-*d*_6_) *δ* 165.6, 162.9, 158.0, 155.5, 153.0 (d, *J* = 244.3 Hz), 151.6, 151.4, 137.1 (d, *J* = 10.2 Hz), 135.7 (d, *J* = 12.3 Hz), 133.0, 132.9, 130.7, 128.4, 128.1, 127.7, 126.7, 126.5, 125.3, 124.4, 123.6, 121.1, 117.7 (d, *J* = 2.6 Hz), 111.0, 110.8, 109.7 (d, *J* = 22.9 Hz); ESI-MS: *m*/*z* 476.2 [M + H]^+^.

##### N-(4-((2-chloropyridin-4-yl)oxy)-3-fluorophenyl)-4-(thiophen-2-yl)thiazole-2-carboxamide (51aj)

Yellow solid; yield 56% (two steps); m.p. 184–186 °C. HPLC purity 95.7%. IR (KBr) *ν*_max_/cm^–^^1^ 3435, 3102, 2924, 2853, 1672, 1606, 1584, 1559, 1538, 1509, 1458, 1406, 1394, 1303, 1264, 1234, 1196, 1120, 1070, 920, 712. ^1^H NMR (400 MHz, DMSO-*d*_6_) *δ* 10.90 (s, 1H), 8.34 (s, 1H), 8.32 (d, *J* = 6.0 Hz, 1H), 8.05 (dd, *J* = 2.4, 12.8 Hz, 1H), 7.84–7.80 (m, 1H), 7.74 (dd, *J* = 1.2, 3.6 Hz, 1H), 7.63 (dd, *J* = 1.2, 5.2 Hz, 1H), 7.49–7.44 (m, 1H), 7.18 (dd, *J* = 3.6, 5.2 Hz, 1H), 7.10 (d, *J* = 2.0 Hz, 1H), 7.00 (dd, *J* = 2.4, 6.0 Hz, 1H); ^13^C NMR (100 MHz, DMSO-*d*_6_) *δ* 165.6, 163.0, 157.8, 153.0 (d, *J* = 244.1 Hz), 151.6, 151.4, 150.3, 137.0 (d, *J* = 10.0 Hz), 136.6, 135.7 (d, *J* = 12.2 Hz), 128.0, 126.9, 125.8, 123.6, 119.3, 117.8 (d, *J* = 3.1 Hz), 111.0, 110.8, 109.8 (d, *J* = 22.8 Hz); ESI-MS: *m*/*z* 431.9 [M + H]^+^.

##### 4-(3-Chloro-4-fluorophenyl)-N-(4-((2-chloropyridin-4-yl)oxy)-3-fluorophenyl)thiazole-2-carboxamide (51ak)

White solid; yield 76% (two steps); m.p. 168–170 °C. HPLC purity 96.6%. IR (KBr) *ν*_max_/cm^–^^1^ 3435, 3096, 2924, 1683, 1585, 1559, 1538, 1506, 1482, 1442, 1328, 1299, 1266, 1232, 1200, 1166, 1123, 1073, 919, 820. ^1^H NMR (400 MHz, DMSO-*d*_6_) *δ* 10.90 (s, 1H), 8.62 (s, 1H), 8.41 (dd, *J* = 2.4, 7.2 Hz, 1H), 8.32 (d, *J* = 5.6 Hz, 1H), 8.17–8.13 (m, 1H), 8.05 (dd, *J* = 2.4, 12.8 Hz, 1H), 7.81 (d, *J* = 8.8 Hz, 1H), 7.58–7.45 (m, 2H), 7.09 (d, *J* = 2.0 Hz, 1H), 7.00 (dd, *J* = 2.0, 5.6 Hz, 1H); ^13^C NMR (100 MHz, DMSO-*d*_6_) *δ* 165.6, 163.0, 157.7, 157.3 (d, *J* = 246.9 Hz), 153.1 (d, *J* = 244.8 Hz), 153.0, 151.6, 151.4, 137.0 (d, *J* = 10.1 Hz), 135.7 (d, *J* = 12.0 Hz), 131.1 (d, *J* = 3.6 Hz), 128.4, 127.0 (d, *J* = 7.5 Hz), 123.6, 121.6, 120.2 (d, *J* = 18.0 Hz), 117.7 (d, *J* = 3.4 Hz), 117.3 (d, *J* = 21.0 Hz), 111.0, 110.8, 109.7 (d, *J* = 22.9 Hz); ESI-MS: *m*/*z* 478.1 [M + H]^+^.

##### N-(4-((2-Chloropyridin-4-yl)oxy)-3-fluorophenyl)-4-(3,4-difluorophenyl)thiazole-2-carboxamide (51al)

Pale yellow solid; yield 67% (two steps); m.p. 170–172 °C. HPLC purity 96.2%. IR (KBr) *ν*_max_/cm^–^^1^ 3434, 3086, 2923, 1667, 1607, 1584, 1559, 1537, 1509, 1459, 1302, 1268, 1234, 1199, 1120, 1073, 920, 774. ^1^H NMR (400 MHz, DMSO-*d*_6_) *δ* 10.88 (s, 1H), 8.59 (s, 1H), 8.32 (d, *J* = 5.6 Hz, 1H), 8.29–8.23 (m, 1H), 8.06–7.99 (m, 2H), 7.81 (d, *J* = 8.4 Hz, 1H), 7.62–7.55 (m, 1H), 7.50–7.45 (m, 1H), 7.10 (d, *J* = 2.4 Hz, 1H), 7.00 (dd, *J* = 2.4, 5.6 Hz, 1H); ^13^C NMR (100 MHz, DMSO-*d*_6_) *δ* 165.6, 162.9, 157.7, 153.2, 153.0 (d, *J* = 244.2 Hz), 151.6, 151.4, 150.8 (dd, *J* = 10.4, 243.7 Hz), 148.3 (dd, *J* = 10.7, 243.9 Hz), 137.0 (d, *J* = 10.1 Hz), 135.7 (d, *J* = 12.2 Hz), 130.9 (dd, *J* = 3.5, 6.5 Hz), 123.6, 123.2 (dd, *J* = 3.5, 6.5 Hz), 121.5, 118.0 (d, *J* = 17.3 Hz), 117.7 (d, *J* = 3.3 Hz), 115.6 (d, *J* = 18.4 Hz), 111.0, 110.8, 109.6 (d, *J* = 22.6 Hz); ESI-MS: *m*/*z* 462.2 [M + H]^+^.

##### 4-(3-Chloro-4-fluorophenyl)-N-(4-((2,3-dichloropyridin-4-yl)oxy)-3-fluorophenyl)thiazole-2-carboxamide (51am)

Yellow solid; yield 78% (two steps); m.p. 123–125 °C. HPLC purity 97.3%. IR (KBr) *ν*_max_/cm^–^^1^ 3434, 2928, 2850, 1684, 1610, 1568, 1559, 1549, 1538, 1506, 1485, 1460, 1310, 1263, 1199, 1130, 1075, 935, 820, 728. ^1^H NMR (400 MHz, DMSO-*d*_6_) *δ* 10.93 (s, 1H), 8.62 (s, 1H), 8.41 (dd, *J* = 2.4, 7.6 Hz, 1H), 8.24 (d, *J* = 5.6 Hz, 1H), 8.17–8.13 (m, 1H), 8.07 (dd, *J* = 2.4, 13.2 Hz, 1H), 7.83 (d, *J* = 8.8 Hz, 1H), 7.58–7.49 (m, 2H), 6.89 (d, *J* = 5.6 Hz, 1H); ^13^C NMR (100 MHz, DMSO-*d*_6_) *δ* 162.9, 161.2, 157.8, 157.3 (d, *J* = 246.9 Hz), 153.0, 152.7 (d, *J* = 244.7 Hz), 149.4, 148.7, 137.2 (d, *J* = 10.0 Hz), 135.8 (d, *J* = 12.2 Hz), 131.1 (d, *J* = 3.8 Hz), 128.4, 127.0 (d, *J* = 7.6 Hz), 123.4, 121.6, 120.2 (d, *J* = 18.0 Hz), 117.8, 117.7 (d, *J* = 3.3 Hz), 117.3 (d, *J* = 21.2 Hz), 110.4, 109.7 (d, *J* = 22.5 Hz); ESI-MS: *m*/*z* 512.1 [M + H]^+^.

##### N-(4-((2,3-Dichloropyridin-4-yl)oxy)-3-fluorophenyl)-4-(3,4-difluorophenyl)thiazole-2-carboxamide (51an)

Yellow solid; yield 69% (two steps); m.p. 156–158 °C. HPLC purity 96.2%. IR (KBr) *ν*_max_/cm^–^^1^ 3364, 2924, 2855, 1678, 1608, 1559, 1538, 1506, 1482, 1453, 1379, 1328, 1300, 1199, 1116, 1077, 936, 821, 773. ^1^H NMR (400 MHz, DMSO-*d*_6_) *δ* 10.92 (s, 1H), 8.61 (s, 1H), 8.30–8.27 (m, 1H), 8.24 (d, *J* = 5.2 Hz, 1H), 8.07 (dd, *J* = 2.4, 12.8 Hz, 1H), 8.04–8.00 (m, 1H), 7.85–7.81 (m, 1H), 7.63–7.51 (m, 2H), 6.90 (d, *J* = 5.6 Hz, 1H); ^13^C NMR (100 MHz, DMSO-*d*_6_) *δ* 162.8, 161.2, 157.8, 153.2, 152.7 (d, J = 244.7 Hz), 150.8 (dd, *J* = 9.7, 243.8 Hz), 149.4, 148.7, 148.4 (dd, *J* = 10.2, 244.3 Hz), 137.2 (d, *J* = 10.2 Hz), 135.9 (d, *J* = 12.3 Hz), 130.9 (dd, *J* = 3.6, 6.4 Hz), 123.4, 123.3 (dd, *J* = 2.9, 6.5 Hz), 121.6, 118.0 (d, *J* = 17.4 Hz), 117.8, 117.7 (d, *J* = 2.8 Hz), 115.6 (d, *J* = 18.6 Hz), 110.4, 109.6 (d, *J* = 22.8 Hz); ESI-MS: *m*/*z* 496.2 [M + H]^+^.

### Biology

#### Cell proliferation assay

MTT assay was used to determine the cytotoxic activity of target compounds against different cell lines. The cells (∼4 × 10^3^ cells/well) were seeded in 96-well plates filled with minimum essential medium (MEM) supplemented with 10% foetal bovine serum (FBS) and incubated in 5% CO_2_ at 37 °C for 24 h. Then, the cells were treated with five doses of test compounds (pre-dissolved in DMSO), and 0.1% DMSO was used as a negative control. After incubation at 37 °C for 48 h, MTT was added to each well and cells were cultured for an additional 4 h. Next, the formazan crystal in the well was dissolved with 100 µL of DMSO for optical density reading at 492 nm (for the absorbance of formazan) and 630 nm (for the reference wavelength). The results expressed as IC_50_ were the average of three determinations calculated by using the Bacus Laboratories Incorporated Slide Scanner (Bliss) software.

#### Tyrosine kinases assay

The tyrosine kinases inhibitory activities of target compounds were evaluated in 384-well microtiter plates using purified kinases purchased from Invitrogen (Waltham, MA) by homogeneous time-resolved fluorescence (HTRF) assay. The HTRF KinEASE TK kit was performed according to the instructions. After the kinases and test compounds were incubated at 25–30 °C for 5 min, the kinase reactions were initiated by addition of 39 μL of kinase reaction buffer solution and incubated at 25–30 °C for 30 min. The reactions were quenched by addition 10 µL mixed detection solution. Then, the plates were read using an Envision plate reader at 615 nm and 620 nm, respectively. The inhibition rate (%) was calculated using the following equation: inhibition rate = [(activity of enzyme with tested compound – min)/(max – min)] × 100 (max: the enzyme activity in the presence of enzyme, substrates and cofactors; min: the enzyme activity in the presence of substrates, cofactors and in the absence of enzyme). IC_50_ value was calculated from the inhibition curve using GraphPad Prism 5.0 software (La Jolla, CA).

#### Analysis of cellular apoptosis

Apoptosis was detected by an Annexin V/propidium iodide (PI) double staining kit (Beyotime Biotechnology, Shanghai, China). Briefly, MKN-45 cells were treated with different doses of test compounds for 24 h. Cells were harvested by trypsinisation and washed with ice-cold PBS solution. Cells were labelled with annexin V and PI and incubated at room temperature for 30 min in the dark. The labelled cells were analysed by a FACScan flow cytometer (Becton Dickinson, Franklin Lakes, NJ).

#### Analysis of cell cycle distribution

After MKN-45 cells were incubated in the presence of test compounds at the indicated concentrations for 24 h, cells were fixed with ice-cold 70% EtOH at 4 °C overnight, incubated with RNase at 37 °C for 30 min and stained with PI working solution for 30 min in the dark. The cells were analysed by flow cytometry (FACScan, Becton Dickinson, Franklin Lakes, NJ) and the distribution and percentages of cells in the G1, S, and G2/M phases of the cell cycle were analysed using the ModFit LT software.

#### Western blot analysis

MKN-45 cells were incubated with the test compound at various concentrations for 24 h. After incubation, cells were harvested in PBS solution containing proteinase inhibitors and phosphatase inhibitors, and then sonicated. Whole cell lysates were separated by sodium dodecyl sulphate-polyacrylamide gel electrophoresis (SDS-PAGE) and transferred to nitrocellulose membrane (Beyotime Biotechnology, Shanghai, China). The membrane was incubated with primary antibody followed by labelling with horseradish peroxidase (HRP)-conjugated secondary antibody (Beyotime Biotechnology, Shanghai, China). The blots were developed with enhanced chemiluminescence and visualised by an LAS4000 imager (Fuji Photo Film, Minato City, Japan).

#### Pharmacokinetic study

The experimental procedures and the animal use and care protocols were approved by the Committee on Ethical Use of Animals of Shenzhen Second People’s Hospital. Each male BALB/c mice received a single compound **51am** as a solution in PEG 400/water (70:30) by either oral route (10 mg/kg) or i.v. route (1.5 mg/kg). Blood samples were collected into the microcentrifuge tube containing heparin at time points 0 (prior to dosing) 5, 10, 20, 30, 60, 180, 360, 480, 600, 720, 1080, and 1440 min after dosing. The blood samples were centrifuged; the obtained plasma was separated and stored at –20 °C. All samples for **51am** were measured by liquid chromatography–tandem mass spectrometry (LC/MS/MS).

### Docking study

The three-dimensional structures of c-Met and VEGFR-2 were retrieved from the RCSB Protein Data Bank (http://www.pdb.org). Under physiological pH, the binding water and ligand were deleted and the polar hydrogen was added, the protonated state of the important residues was also adjusted by using Sybyl 6.9.1. Molecular docking analysis was carried out by Autodock 4.2 package to explore the binding mode for the active site of c-Met/VEGFR-2 with ligands.

## Supplementary Material

Supplemental Material
