# Supplementary material for: Design, synthesis, and biological evaluation of thiazole/thiadiazole carboxamide scaffold-based derivatives as potential c-Met kinase inhibitors for cancer treatment
Source: J Enzyme Inhib Med Chem. 2023 Aug 29;38(1):2247183. doi: 10.1080/14756366.2023.2247183 (PMC10467532; doi:10.1080/14756366.2023.2247183)

Supporting Information

Design, synthesis and biological evaluation of thiazole/thiadiazole carboxamide scaffolds based derivatives as potential c-Met kinase inhibitors for cancer treatment

Xiang Nan[a,b], Qiu-Xu Wang[a,*], Shao-Jun Xing[b] and Zhi-Gang Liang[a,*]

[a]*Department of Stomatology, Shenzhen Second People's Hospital, Shenzhen, China*

[b]*Guangdong Key Laboratory for Biomedical Measurements and Ultrasound Imaging, National-Regional Key Technology Engineering Laboratory for Medical Ultrasound, School of Biomedical Engineering, Shenzhen University Medical school, Shenzhen, China*

Corresponding authors

*Qiu-Xu Wang: wangqx8811@163.com; *Zhi-Gang Liang: liangzhigang@yeah.net

1. General synthetic procedures for the preparation of intermediates **9–13**, **18–26** and **28–30**………....2

2. NMR Spectra of target compounds **51a–51an**…………………………………………………......6

1. General synthetic procedures for the preparation of intermediates **9–13**, **18–26** and **28–30**

*Synthesis of 4,5-dimethoxy-2-nitroacephenone (**9**)*

3,4-Dimethoxyacetophenon **8** (3.6 g, 20 mmol) was added to HNO_3 (40%, 25 ml) under 0 °C and the reaction mixture was stirred at 0 °C overnight. After the reaction was completed, the mixture was poured into cold water (60 ml), stirred for a while and extracted with ethyl acetate (4 × 50 ml). The combined organic layers were washed with brine, dried over Na_2SO_4, concentrated in vacuum and purified by silica gel chromatography using hexane-ethyl acetate (12:1~5:1, v/v) as eluent to give **9** (2.52 g, 56% yield) as a yellow solid. M.p. 134–136 °C. IR (KBr) ν_{max}/cm^{-1} 3422, 1702, 1576, 1514, 1463, 1440, 1326, 1283, 1224, 1183, 1114, 1046, 882, 790, 600, 565. ^1H NMR (400 MHz, $CDCl_3$) δ 7.59 (s, 1 H), 6.74 (s, 1 H), 3.96 (s, 6 H), 2.48 (s, 3 H).

*Synthesis of 1-(4,5-dimethoxy-2-nitrophenyl)-3-(dimethylamino)-prop-2-en-1-one (**10**)*

To a stirred solution of compound **9** (2.25 g, 10 mmol) in toluene (20 ml) was added DMF-DMA (6.7 ml, 50 mmol). The reaction mixture was heated to reflux for 10 h. After cooling to -10 °C, the resultant solid was collected by filtration, washed with toluene (8 ml) and dried under vacuum to yield the desired compound **10** (2.1 g, 75% yield) as a yellow powder. M.p. 150–152 °C. IR (KBr) ν_{max}/cm^{-1} 1638, 1603, 1580, 1543, 1526, 1489, 1462, 1433, 1418, 1357, 1275, 1216, 1175, 1092, 897, 774. ^1H NMR (400 MHz, $CDCl_3$) δ 7.55 (s, 1 H), 6.81 (s, 1 H), 5.17 (d, J = 10.8 Hz, 1 H), 3.93 (s, 6 H), 3.05 (s, 3 H), 2.82 (s, 3 H).

*Synthesis of 6,7-dimethoxy-4-quinolin-ol (**11**)*

Fe powder (1.95 g, 35 mmol) was added to a solution of **10** (1.96 g, 7 mmol) in acetic acid (18 ml), the mixture was heated to 80 °C with vigorous agitation for 2 h. The hot solution was filtered immediately through celite and the filter cake was washed to be colorless with water and dried at room temperature to obtain **11** (0.93 g, 65% yield) as a white solid. M.p. 228–230 °C. IR (KBr) ν_{max}/cm^{-1} 3096, 2970, 1604, 1546, 1498, 1440, 1402, 1301, 1272, 1239, 1220, 1088, 1074, 993, 871, 836, 821, 729, 561, 519. ^1H NMR (400 MHz, DMSO-d_6) δ 11.62 (br s, 1 H), 7.78 (d, J = 7.2 Hz, 1 H), 7.43 (s, 1 H), 6.96 (s, 1 H), 5.96 (d, J = 7.2 Hz, 1 H), 3.85 (s, 3 H), 3.82 (s, 3 H).

*Synthesis of 4-chloro-6,7-dimethoxyquinoline (**12**)*

A solution of **11** (0.82 g, 4 mmol) in $POCl_3$ (8 ml) with a few drops DMF (~10) were heated to reflux for 1 h. After cooling to room temperature, the mixture was concentrated and azetroped with toluene. The residue was poured into cold water, neutralized with saturated $NaHCO_3$ solution and extracted with ethyl acetate (3 × 40 ml). The combined organic layers were washed with brine, dried over Na_2SO_4, concentrated in vacuum and purified by silica gel chromatography using hexane-ethyl acetate (7:1~3:1, v/v) as eluent to afford **12** (0.74 g, 83% yield) as a white solid. M.p. 130–132 °C. IR (KBr) ν_{max}/cm^{-1} 3444, 3004, 2939, 2830, 1618, 1580, 1568, 1503, 1473, 1425, 1331, 1296, 1248, 1223, 1143, 1007, 855, 780, 684. ^1H NMR (400 MHz, $CDCl_3$) δ 8.58 (d, J = 4.8 Hz, 1 H), 7.51 (d, J = 4.8 Hz, 1 H), 7.40 (s, 1 H), 7.29 (s, 1 H), 3.94 (s, 6 H).

*Synthesis of anilines (**13a–13g**)*

A mixture of **12** (0.67 g, 3 mmol) and corresponding 4-nitrophenol (3.6 mmol) in chlorobenzene (10 ml) was heated to 140 °C for 20 h. After cooling to room temperature, the reaction mixture was concentrated in vacuum. The residue was dissolved in CH_2Cl_2 (20 ml), the resulting solution was washed with saturated aqueous solution of K_2CO_3 (2 × 20 ml) and water (20 ml), dried over Na_2SO_4 and concentrated in vacuum to afford a yellow solid, which was used directly without further purification. Next, tin (II) chloride dihydrate (1.13 g, 5 mmol) was added to a solution of the prepared yellow solid (2 mmol) in ethanol (10 ml). The resulting yellow slurry was heated at 70 °C for 6 h. The mixture was cooled to room temperature, diluted with aq. NaOH (1 N, 20 ml) and extracted with ethyl acetate (3 × 20 ml). The combined organic layers were washed with aq. NaOH (1 N, 50 ml), H_2O and brine. After drying over Na_2SO_4, filtering, and concentrating of the organic phase, the yellow residue was purified by silica gel column chromatography using methanol-dichloromethane (1:25~1:10, v/v) as eluent to yield the title compounds with 39%–54% overall yield in two-step.

*Synthesis of 3-aminothiophene (**18**)*

Compound **14** (6.28 g, 40 mmol) was added to aq. NaOH (2 M, 40 ml), the resulting mixture was refluxed for 30 min. The mixture was cooled to room temperature and acidified to pH 3 with concentrated HCl, the thick precipitate was filtered off. The precipitate was pressed as dry as possible and then dissolved in acetone (40 ml). The solution was dried over $MgSO_4$, filtered and concentrated to near dryness at room temperature. The residue was treated with 1-propanol (12 ml) and anhydrous oxalic acid (4.05 g, 45 mmol) at 38 °C for 45 min. The mixture was cooled and diluted with ether, and the resulting precipitate was filtered off, washed with ether and dried. The residue was suspended in water (20 ml), basified with concentrated ammonia and extracted with CH_2Cl_2 (3 × 20 ml). The combined organic layers were dried over $MgSO_4$, filtered and concentrated in vacuum to give compound **18** (2.53 g, 64% overall yield) as a brown oil. IR (film) ν_{max}/cm^{-1} 3105, 2873, 2585, 1952, 1584, 1509, 1428, 1401, 1239, 1149, 1065, 940, 866, 830, 779, 698, 590, 544. ^{1}H NMR (400 MHz, $CDCl_3$) δ 7.15 (d, J = 5.2 Hz, 1 H), 6.65 (d, J = 5.2 Hz, 1 H), 6.22 (d, J = 3.2 Hz, 1 H), 3.62 (br s, 2 H).

*Synthesis of 4H-thieno[3,2-b]pyridin-7-one (**20**)*

Trimethyl orthoformate (13.1 ml, 120 mmol) was added to dimethyl-1,3-dioxane-4,6-dione (4.32 g, 30 mmol) and the resulting mixture was stirred at room temperature for 1 h. Then, **18** (2.38 g, 24 mmol) was added in small portions to the mixture. After stirring at 85 °C overnight, the reaction mixture was cooled to room temperature, diluted with isopropyl ether (30 ml) and stirred for 1 h. The resulting precipitate was filtered, washed with isopropyl ether and dried in vacuum. The residue was dissolved in CH_2Cl_2 (40 ml) and stirred for 30 min, the solid was removed by filtration and the solution was concentrated in vacuum to give compound **19**. Next, **19** (5.56 g, 22 mmol) was added to Ph_2O (20 ml) and heated at 240 °C for 30 min. After cooling to 0 °C, the mixture was added with ether and stirred for 1 h. The resulting precipitate was filtered, washed with ether and dried in vacuum to afford **20** (2.36 g, 65% overall yield) as a light orange powder. M.p. 238–240 °C. IR (KBr) ν_{max}/cm^{-1} 3452, 3046, 1614, 1527, 1504, 1439, 1396, 1224, 1105, 811, 796, 685, 633, 504. ^{1}H NMR (400 MHz, DMSO-d_6) δ 11.41 (br s, 1 H), 7.06 (d, J = 5.2 Hz, 1 H), 6.94 (d, J = 7.2 Hz, 1 H), 6.35 (d, J = 5.2 Hz, 1 H), 5.14 (d, J = 6.8 Hz, 1 H).

*Synthesis of 7-chlorothieno[3,2-b]pyridine (**21**)*

To a stirred solution of $POCl_3$ (6.7 ml, 72 mmol) with a few drops DMF (~10) was added **20** (2.27 g, 15 mmol) in portions under 0 °C. After stirring at reflux for 2 h, the mixture was concentrated and the residue was subjected to azeotrope with toluene (3 × 25 ml). The residue was poured into ice-water and neutralized with saturated $NaHCO_3$ solution. Then CH_2Cl_2 (30 ml) was added to the mixture, the insoluble material was filtered off and the filtrate was extracted with CH_2Cl_2 (3 × 30 ml). The combined organic layers were washed with saturated brine, dried over $MgSO_4$ and concentrated in vacuum. The crude product was purified by silica gel column chromatography using hexane-ethyl acetate (8:1~4:1, v/v) as eluent to provide **21** (2.10 g, 83% yield) as a pale yellow solid. M.p. 34–36 °C. IR (KBr) ν_{max}/cm^{-1} 3345, 1567, 1538, 1486, 1450, 1363, 1332, 1200, 1113, 937, 824, 816, 776, 703, 676, 611, 582, 525. ^{1}H NMR (400 MHz, DMSO-d_6) δ 8.64 (d, J = 4.8 Hz, 1 H), 8.25 (d, J = 5.6 Hz, 1 H), 7.66 (d, J = 5.6 Hz, 1 H), 7.56 (d, J = 5.2 Hz, 1 H).

*Synthesis of 3-fluoro-4-(thieno[3,2-b]pyridin-7-yloxy)aniline (**22**)*

A mixture of **21** (2.03 g, 12 mmol), K_2CO_3 (6.62 g, 48 mmol) and 2-fluoro-4-nitrophenol (2.83 g, 18 mmol) was heated to 160 °C in Ph_2O (40 mL) for 6 h. The mixture was cooled to room temperature, diluted with ethyl acetate (80 ml) and washed with water (100 ml). The organic phase was collected, dried over Na_2SO_4 and concentrated under reduced pressure and purified by silica gel column chromatography using petroleum ether-ethyl acetate (9:1~3:1, v/v) as eluent to provide the nitro intermediate as a yellow solid (1.67 g, 48% yield). Subsequently, tin (II) chloride dihydrate (2.82 g, 12.5 mmol) was added to a solution of the nitro compound (1.45 g, 5 mmol) in ethanol (40 ml). The resulting yellow slurry was heated to 70 °C for 6 h. The mixture was then cooled to room temperature, diluted with aq. NaOH (1 N, 40 ml) and extracted with ethyl acetate (3 × 40 ml). The combined organic layers were washed with aq. NaOH (1 N, 50 ml), H_2O and brine. After drying over Na_2SO_4, filtering, and concentrating of the organic phase, the yellow residue was purified by silica gel column chromatography using methanol-dichloromethane (1:20~1:10, v/v) as eluent to yield compound **22** (1.09 g, 84% yield) as a pale yellow solid.

*Synthesis of methyl 3-formamidothiophene-2-carboxylate (**23**)*

To a stirred mixture of formic acid (8 ml) and acetic anhydride (12 ml) under 0 °C was added compound **14** (2.04 g, 13 mmol) in small portions. The cooling bath was removed and the reaction mixture was stirred at room temperature for 12 h. Water (20 ml) was added to the reaction mixture and the resulting solid was collected by vacuum filtration. The crude product was further purified by column chromatography on silica gel using hexane-ethyl acetate (4:1~1:1, v/v) as eluent to afford compound **23** (1.97 g, 82% yield). M.p. 110–112 °C. IR (KBr) ν_{max}/cm^{-1} 3340, 2936, 1680, 1575, 1430, 1380, 1276, 1220, 1084, 946, 876, 782, 652, 550, 503. ^{1}H NMR (400 MHz, DMSO-d_6) δ 10.36 (s, 1 H), 8.44 (s, 1 H), 8.02 (d, J = 5.2 Hz, 1 H), 7.84 (d, J = 5.2 Hz, 1 H), 3.84 (s, 3 H).

*Synthesis of 3H-thieno[3,2-d]pyrimidine-4-one (**24**)*

To a stirred solution of ammonium formate (3.4 g, 54 mmol) in formamide (10 ml) was added **23** (1.85 g, 10 mmol). The resulting mixture was heated to 150 °C for 8 h. After cooling to room temperature, water (20 ml) was added to the mixture. The resulting precipitate was filtered, washed with water and dried. The residue was purified by silica gel column chromatography using hexane-ethyl acetate (6:1~3:1, v/v) to afford **24** (1.09 g, 72% yield) as a white solid. M.p. 269–271 °C. IR (KBr) ν_{max}/cm^{-1} 2864, 1662, 1597, 1509, 1477, 1435, 1361, 1298, 1180, 1037, 900, 867, 815, 791, 572. ^{1}H NMR (400 MHz, DMSO-d_6) δ 12.49 (br s, 1 H), 8.16 (d, J = 5.2 Hz, 1 H), 8.15 (s, 1 H), 7.39 (d, J = 5.2 Hz, 1 H).

*Synthesis of 4-chlorothieno[3,2-d]pyrimidine (**25**)*

To a stirred mixture of DMF (0.2 ml) and dry CH_2Cl_2 (12 ml) at 0 °C under N_2 was added oxalyl chloride (1.02 ml, 12 mmol) slowly over 10 min and compound **24** (0.91 g, 6 mmol) was then added. The cooling bath was removed and the resulting mixture was heated to reflux for 3 h. After cooling to room temperature, the mixture was poured into saturated $NaHCO_3$ and extracted with CH_2Cl_2 (3 × 20 ml). The combined organic layers were dried over Na_2SO_4, filtered and concentrated under reduced pressure to obtain the title compound (0.82 g, 80% yield) as a dark brown solid. M.p. 126–128 °C. IR (KBr) ν_{max}/cm^{-1} 3435, 3081, 1549, 1517, 1455, 1435, 1378, 1301, 1235, 1182, 1102, 1053, 958, 824, 801, 784, 742, 673, 619, 531. ^{1}H NMR (400 MHz, DMSO-d_6) δ 9.04 (s, 1 H), 8.60 (d, J = 5.6 Hz, 1 H), 7.76 (d, J = 5.2 Hz, 1 H).

*Synthesis of 3-fluoro-4-(thieno[3,2-d]pyrimidin-4-yloxy)aniline (**26**)*

To a stirred solution of 4-amino-2-fluorophenol (635 mg, 5 mmol) in dry DMF (15 ml) under 0 °C was added NaH (144 mg, 6 mmol). The resulting mixture was stirred at 0 °C for 10 min and compound **25** (680 mg, 4 mmol) was then added in portions. The reaction mixture was heated to 80 °C for 2 h. After cooling to room temperature, cold water (50 ml) was added to quench the reaction and the mixture was extracted by ethyl acetate (3 × 50 ml). The combined organic layers were washed with brine, dried over Na_2SO_4, concentrated in vacuum and purified by silica gel column chromatograph using petroleum ether-ethyl acetate (8:1~3:1, v/v) as eluent to give compound **26** (0.79 g, 76% yield) as a brown solid.

*Synthesis of 6,7-dimethoxyquinazolin-4(3H)-one (**28**)*

A mixture of 2-amino-4,5-dimethoxybenzoic acid **27** (0.87 g, 4.5 mmol) and formamide (3.6 ml, 90 mmol) was heated to 150 °C for 8 h. After cooling to room temperature, water (20 ml) was added to the reaction mixture and stirred for 45 min. The resulting precipitate was filtered and washed with water. The crude product was recrystallized from ethanol to yield compound **28** (0.76 g, 82% yield) as a white solid. M.p. >300 °C. IR (KBr) ν_{max}/cm^{-1} 3017, 2842, 1651, 1623, 1610, 1505, 1488, 1439, 1357, 1288, 1271, 1249, 1219, 1077, 985, 912, 877, 825. ^{1}H NMR (400 MHz, DMSO-d_6) δ 12.05 (br s, 1 H), 7.98 (s, 1 H), 7.44 (s, 1 H), 7.13 (s, 1 H), 3.91 (s, 3 H), 3.88 (s, 3 H).

*Synthesis of 4-chloro-6,7-dimethoxyquinazoline (**29**)*

Compound **28** (0.62 g, 3 mmol) was added slowly to $POCl_3$ (5.6 ml, 60 mmol) under 0 °C. The cooling bath was then removed and the reaction mixture was heated at reflux for 6 h. After cooling to room temperature, the excess $POCl_3$ was evaporated under vacuum. The residue was poured into ice-water (20 ml), neutralized with ammonium hydroxide and extracted with CH_2Cl_2 (3 ×

20 ml). The combined organic layers were washed with water, brine, dried over $MgSO_4$ and concentrated under reduced pressure. The crude residue was recrystallized from DMF to obtain compound **29** (0.47 g, 70% yield) as a yellow solid. M.p. 181–183 °C. IR (KBr) ν_{max}/cm^{-1} 3452, 1619, 1560, 1507, 1475, 1445, 1434, 1412, 1348, 1256, 1235, 1203, 1162, 1125, 968, 874, 851, 832, 794, 699. ^{1}H NMR (400 MHz, DMSO-d_6) δ 8.84 (s, 1 H), 7.43 (s, 1 H), 7.39 (s, 1 H), 4.02 (s, 6 H).

*Synthesis of 4-((6,7-dimethoxyquinazolin-4-yl)oxy)-3-fluoroaniline (**30**)*

To a stirred solution of 4-amino-2-fluorophenol (305 mg, 2.4 mmol) in dry DMSO (6 ml) under 0 °C was added NaH (72 mg, 3 mmol) and the resulting mixture was continuously stirred at 0 °C for 30 min. Compound **29** (448 mg, 2 mmol) was then added thereto. Next, the cooling bath was removed and the reaction mixture was heated to 60 °C for 2 h. After cooling to room temperature, cold water (50 ml) was added to quench the reaction and the mixture was extracted by ethyl acetate (3 × 50 ml). The combined organic layers were washed with brine, dried over Na_2SO_4, concentrated under vacuum and purified by silica gel column chromatograph using petroleum ether-ethyl acetate (6:1~2:1, v/v) as eluent to give compound **30** (391 mg, 62% yield) as a brown solid.

2. NMR Spectra of target compounds **51a–51an**

^{1}H NMR Spectrum of **51a** (600 MHz, DMSO-d_6)

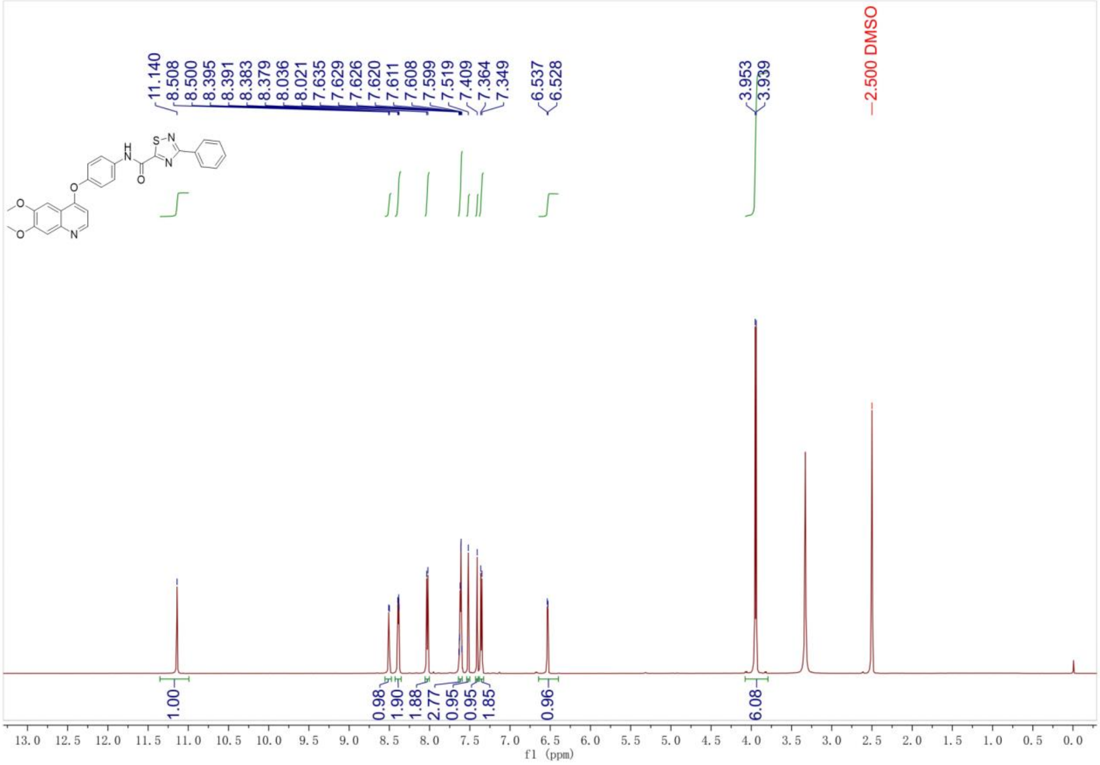

^{13}C NMR Spectrum of **51a** (150 MHz, DMSO-d_6)

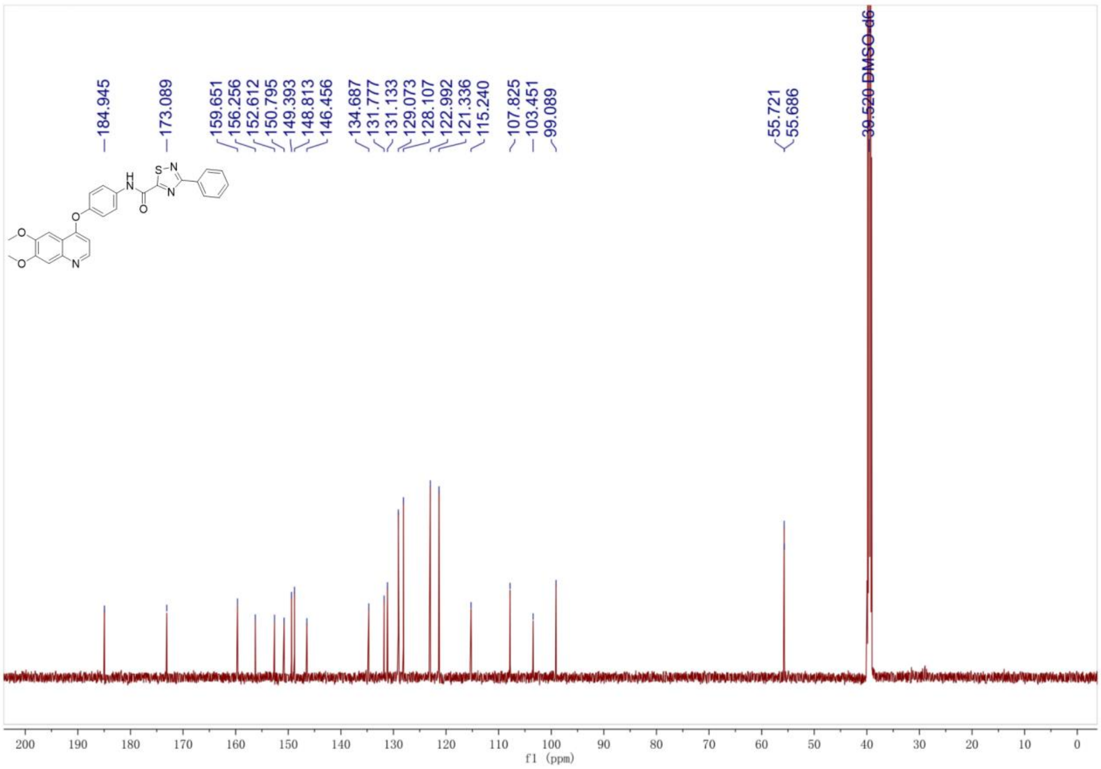

^{1}H NMR Spectrum of **51b** (600 MHz, DMSO-d_6)

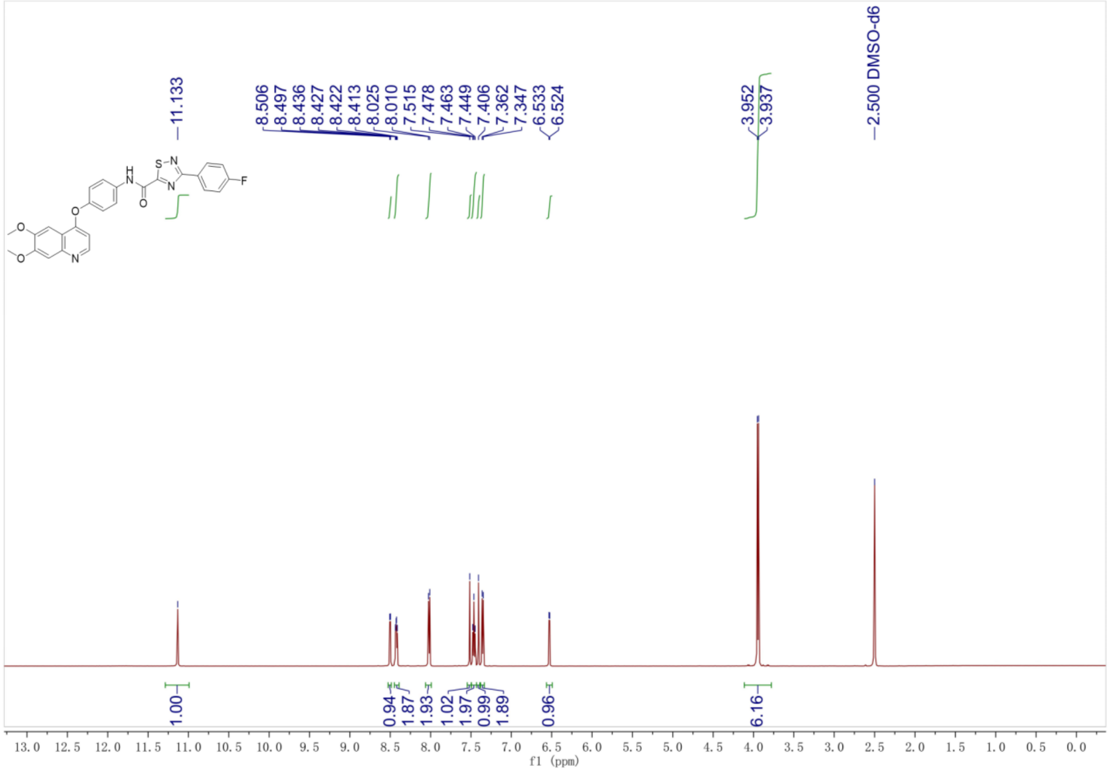

^{13}C NMR Spectrum of **51b** (150 MHz, DMSO-d_6)

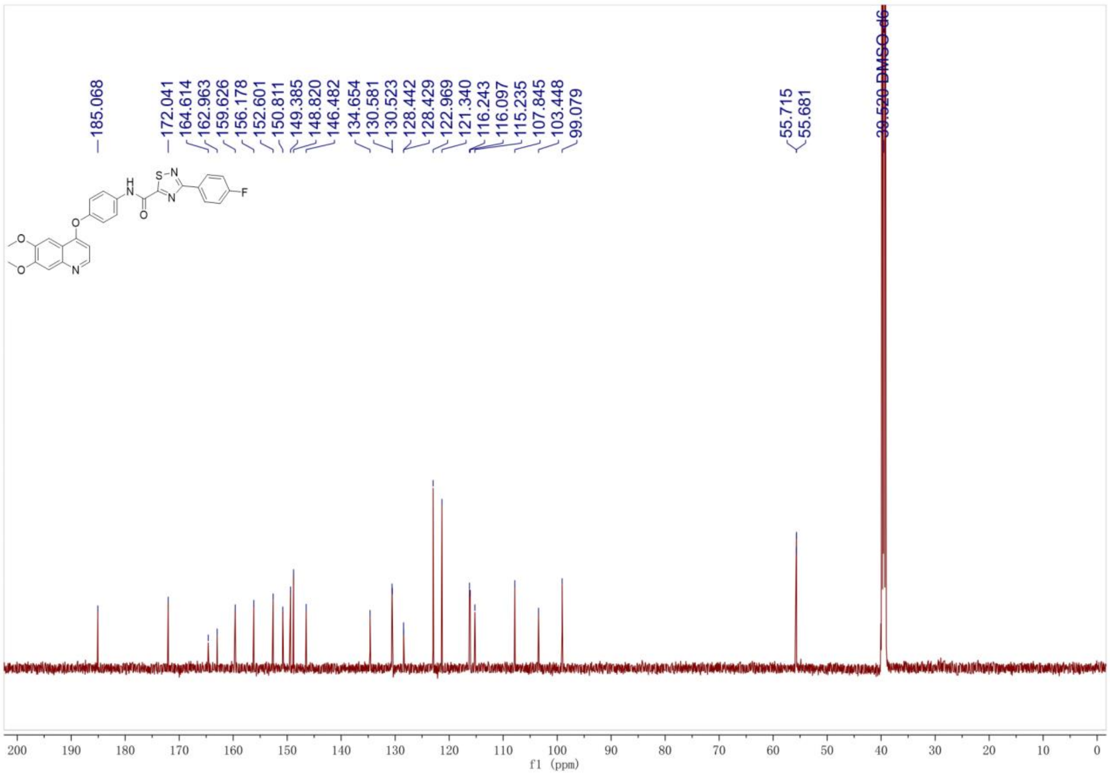

^{1}H NMR Spectrum of **51c** (600 MHz, DMSO-d_6)

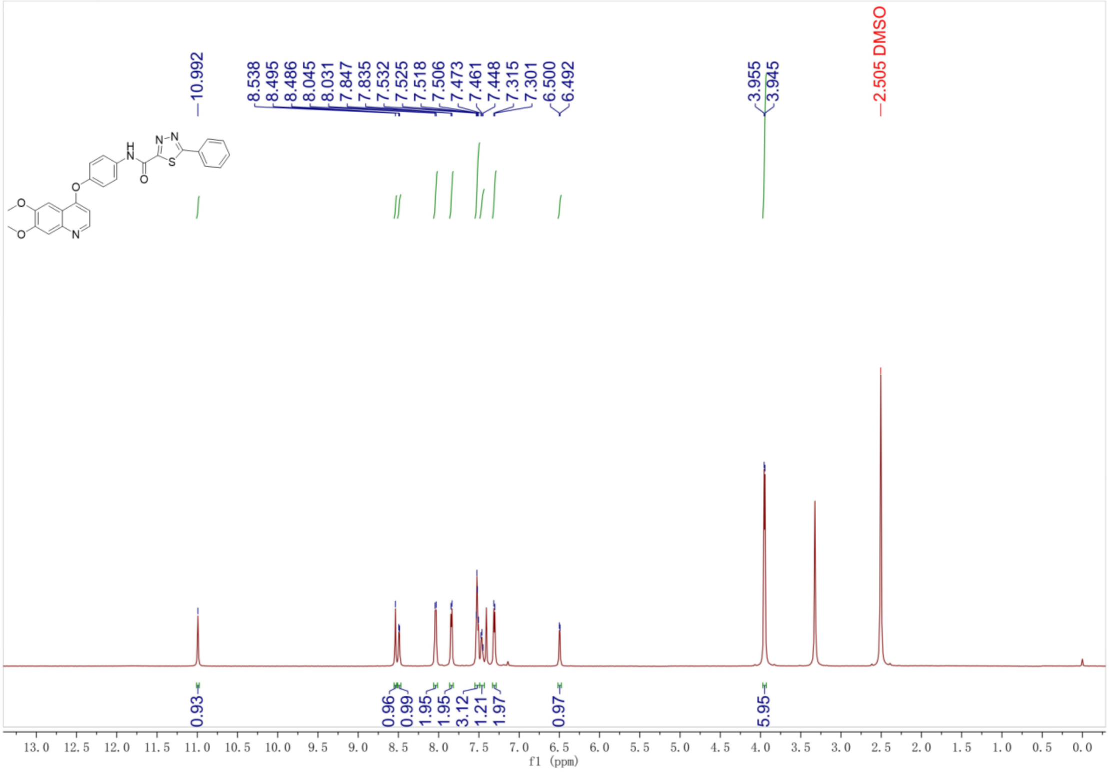

^{13}C NMR Spectrum of **51c** (150 MHz, DMSO-d_6)

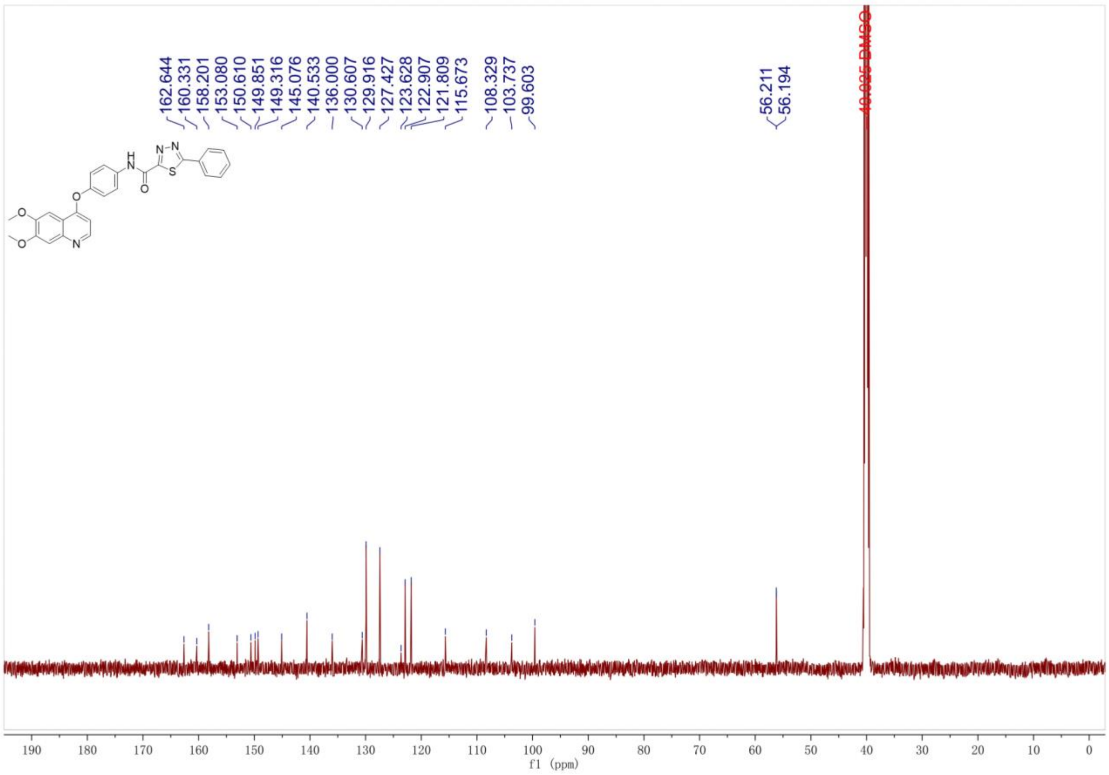

^{1}H NMR Spectrum of **51d** (600 MHz, DMSO-d_6)

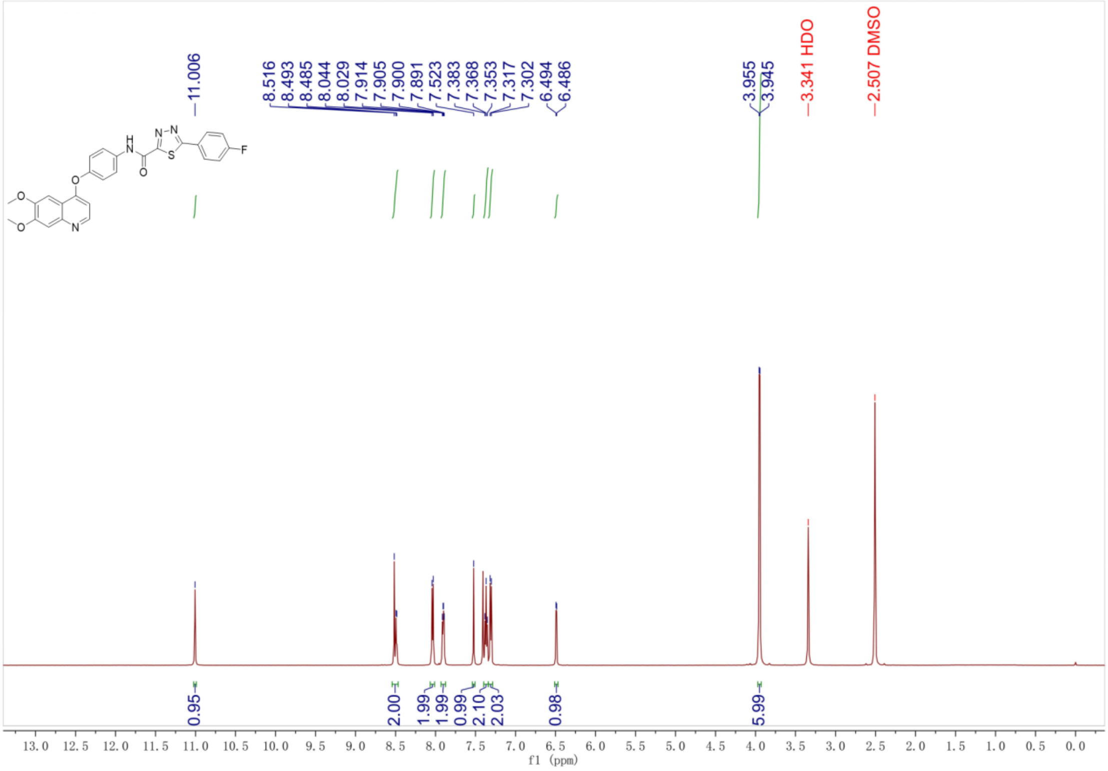

^{13}C NMR Spectrum of **51d** (150 MHz, DMSO-d_6)

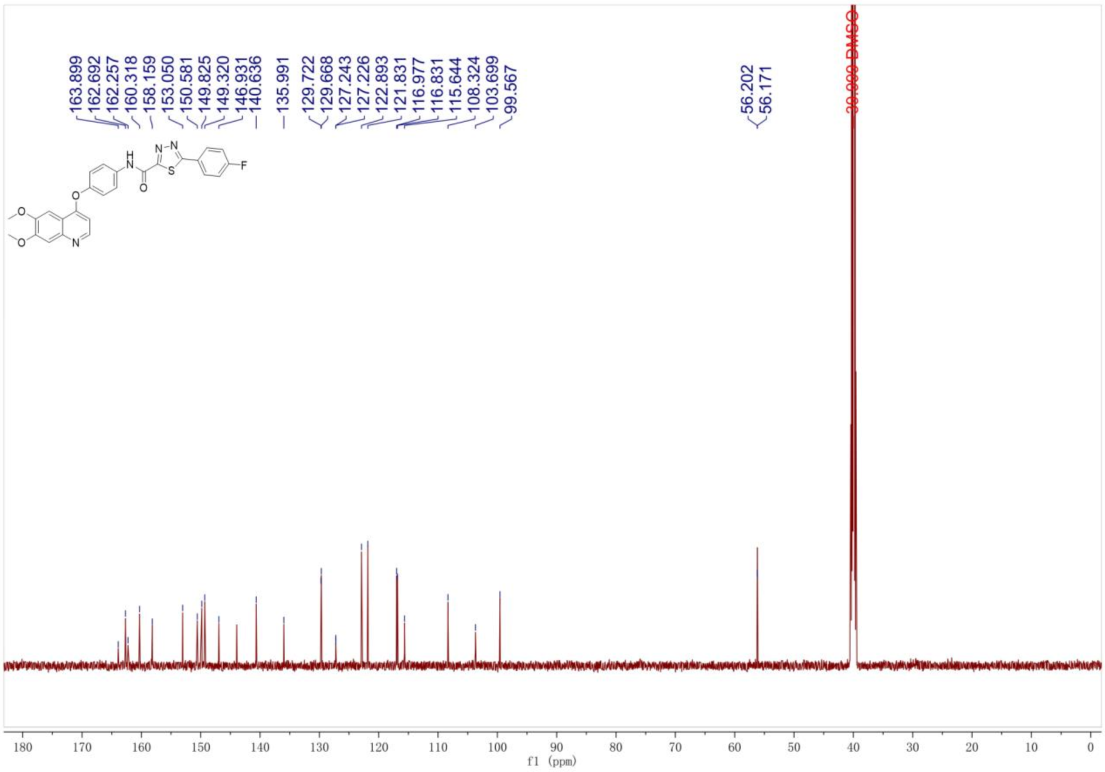

^{1}H NMR Spectrum of **51e** (600 MHz, DMSO-d_6)

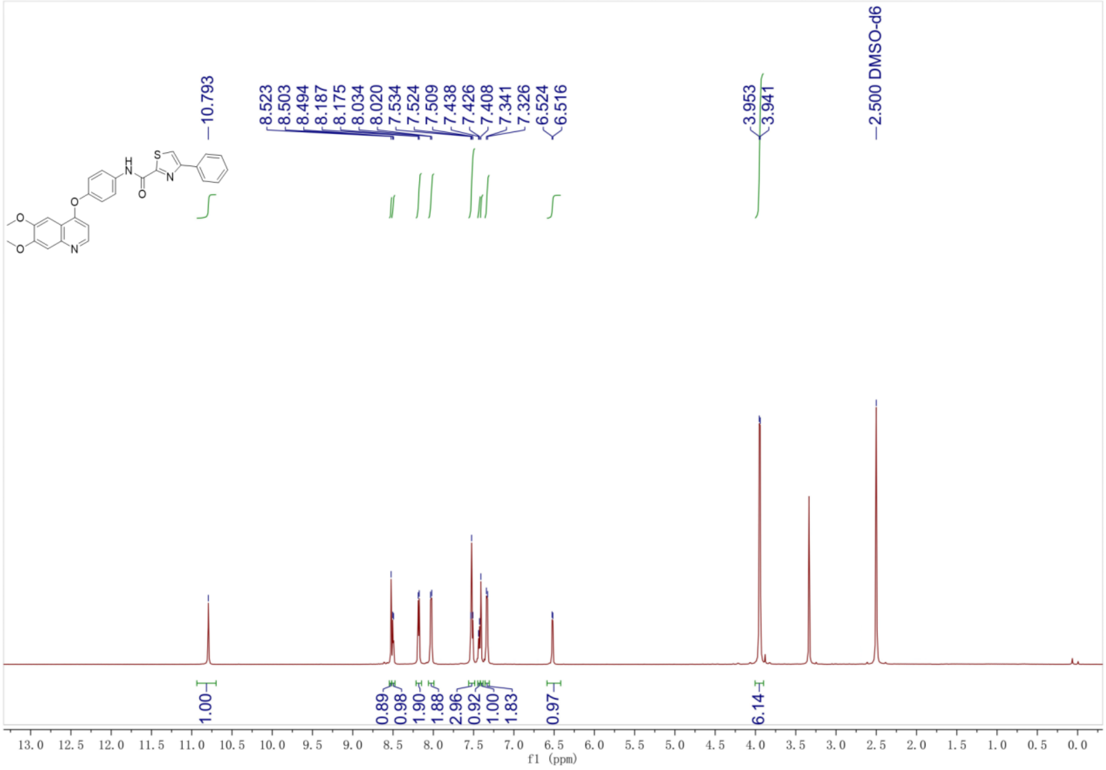

^{13}C NMR Spectrum of **51e** (150 MHz, DMSO-d_6)

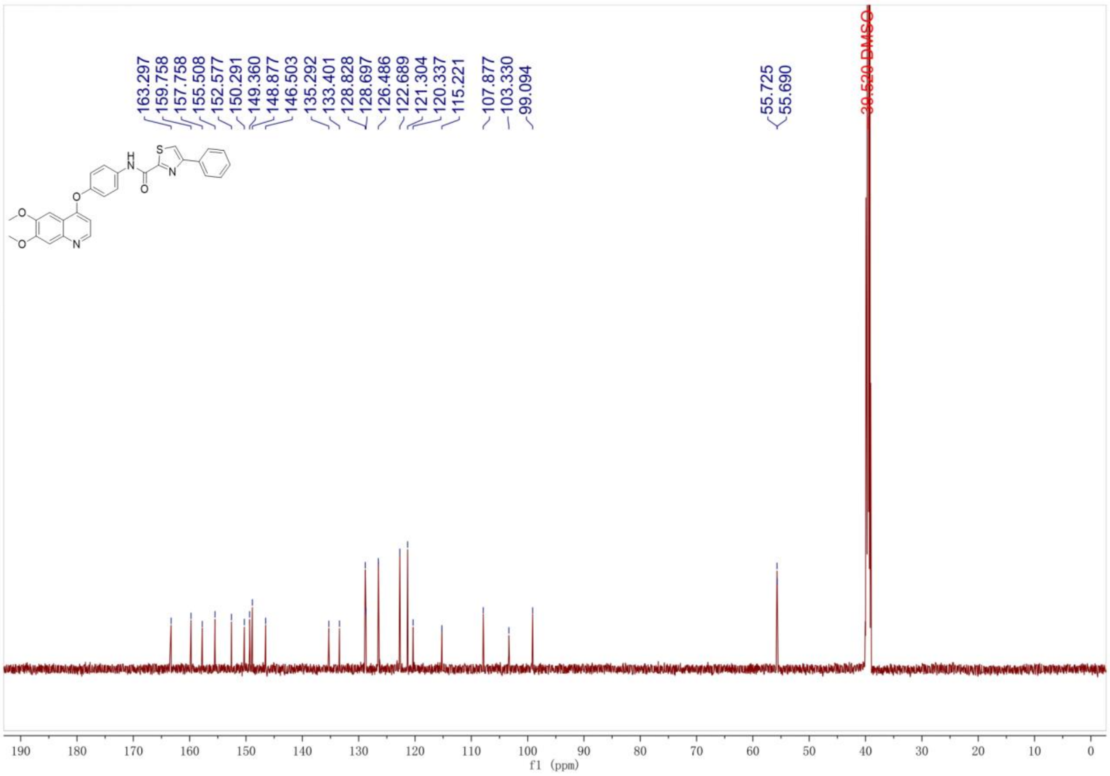

^{1}H NMR Spectrum of **51f** (600 MHz, DMSO-d_6)

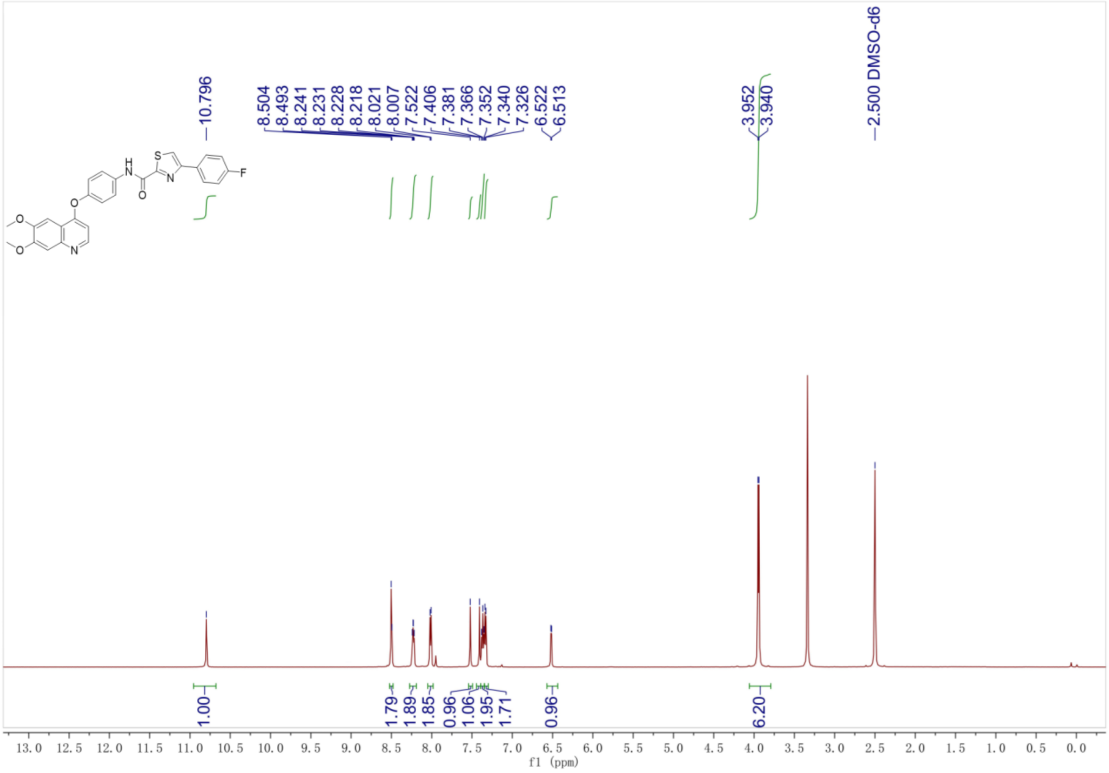

^{13}C NMR Spectrum of **51f** (150 MHz, DMSO-d_6)

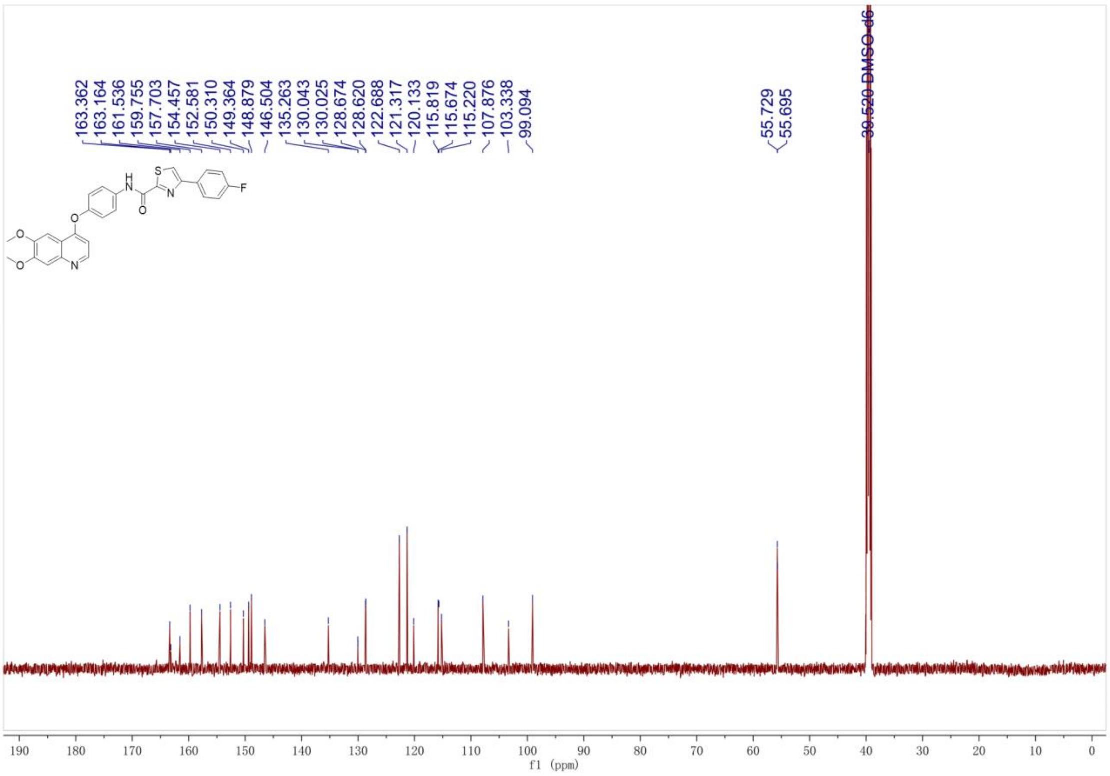

^{1}H NMR Spectrum of **51g** (600 MHz, DMSO-d_6)

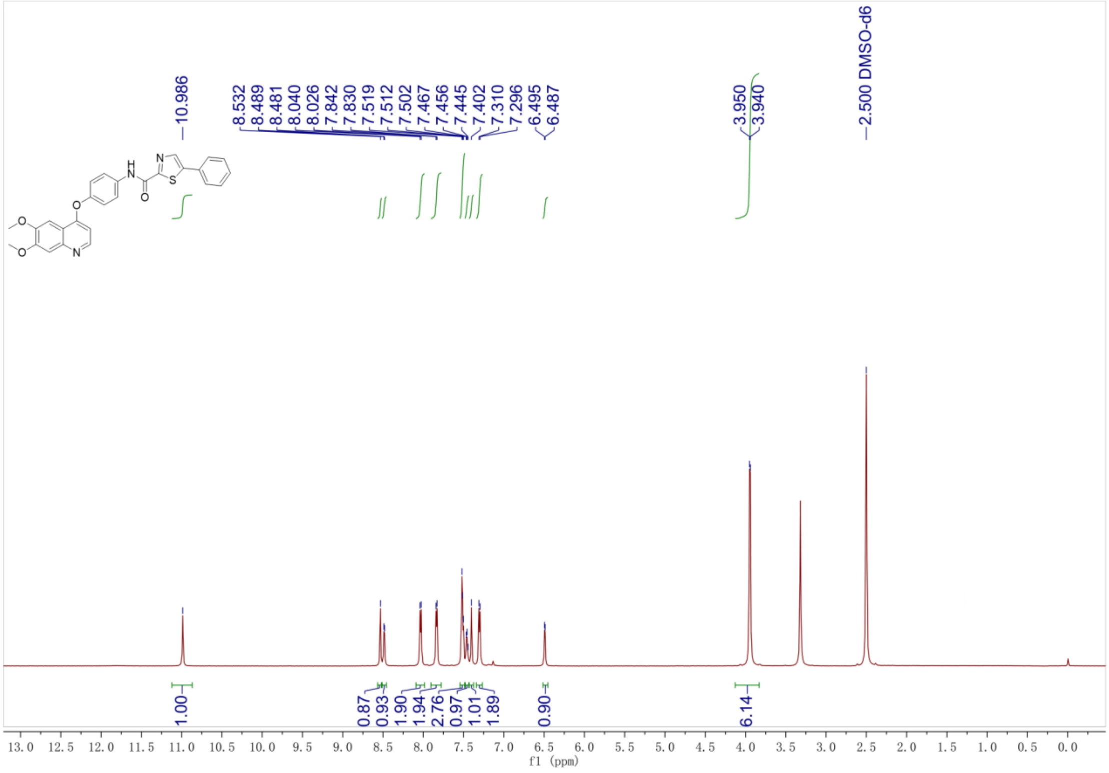

^{13}C NMR Spectrum of **51g** (150 MHz, DMSO-d_6)

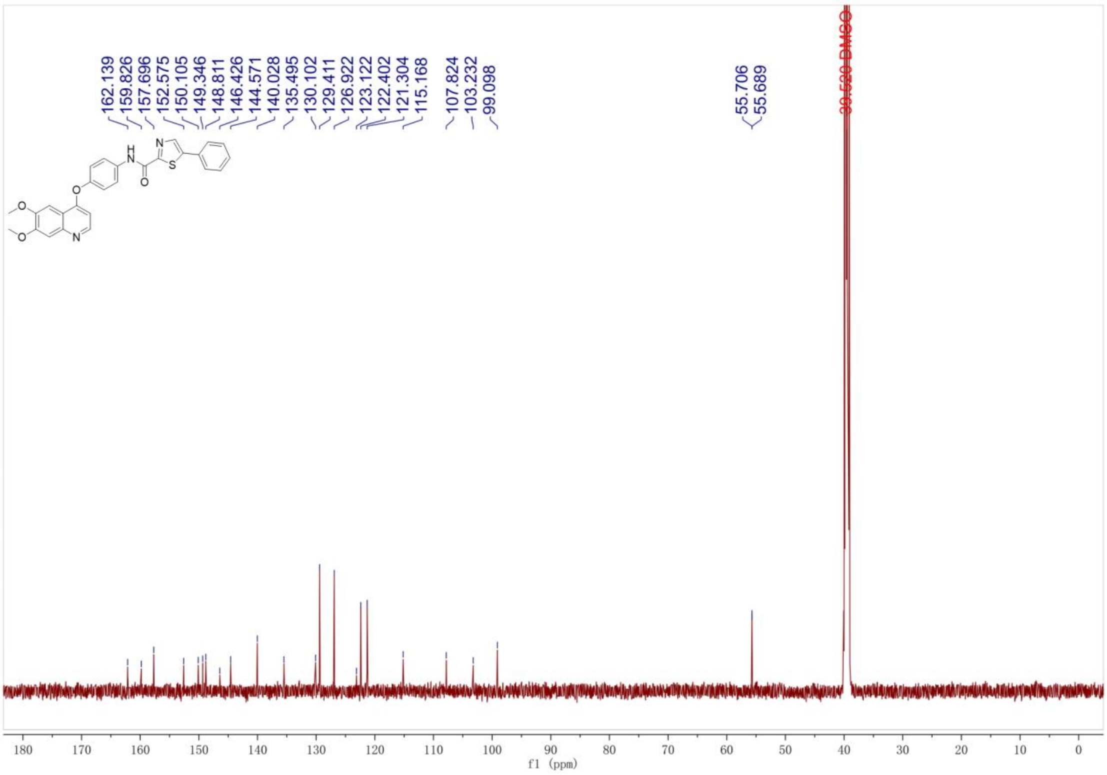

^{1}H NMR Spectrum of **51h** (600 MHz, DMSO-d_6)

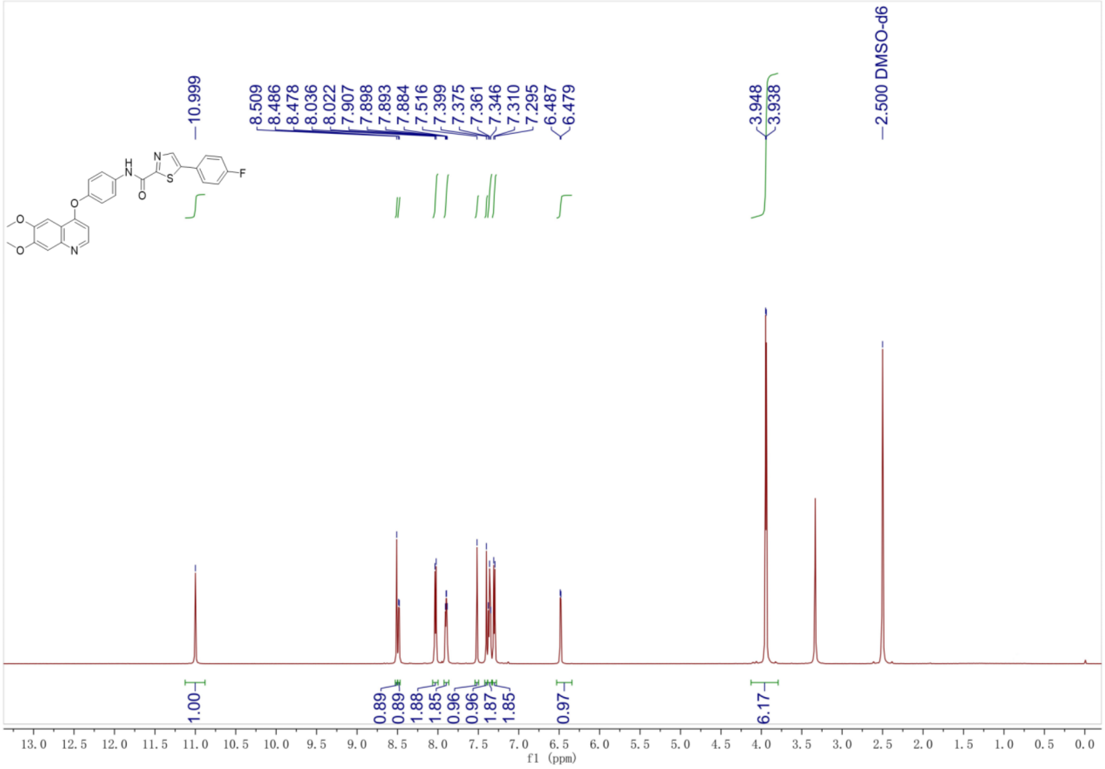

^{13}C NMR Spectrum of **51h** (150 MHz, DMSO-d_6)

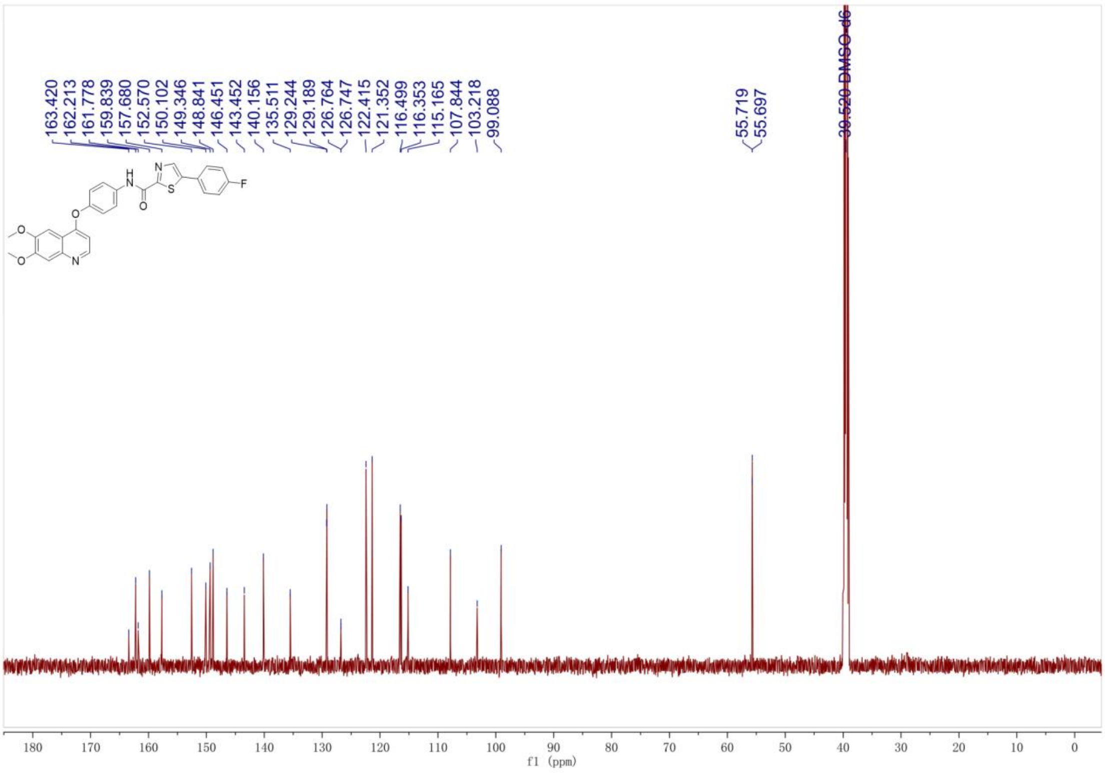

^{1}H NMR Spectrum of **51i** (400 MHz, DMSO-d_6)

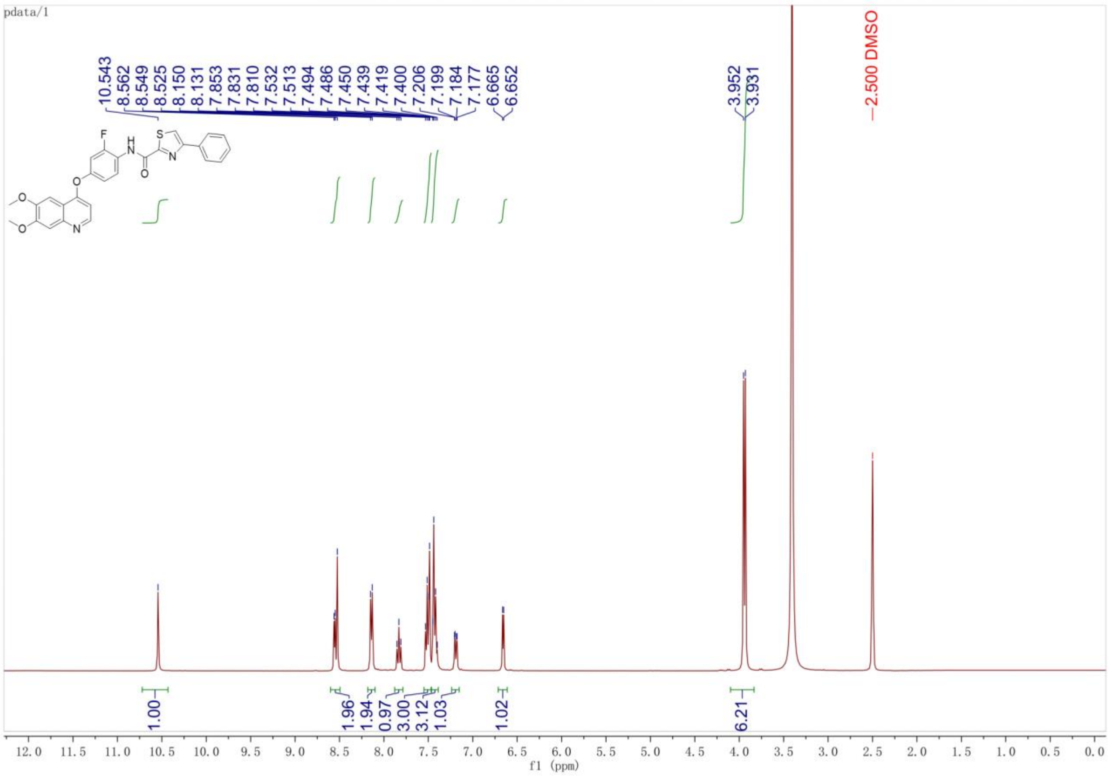

^{13}C NMR Spectrum of **51i** (100 MHz, DMSO-d_6)

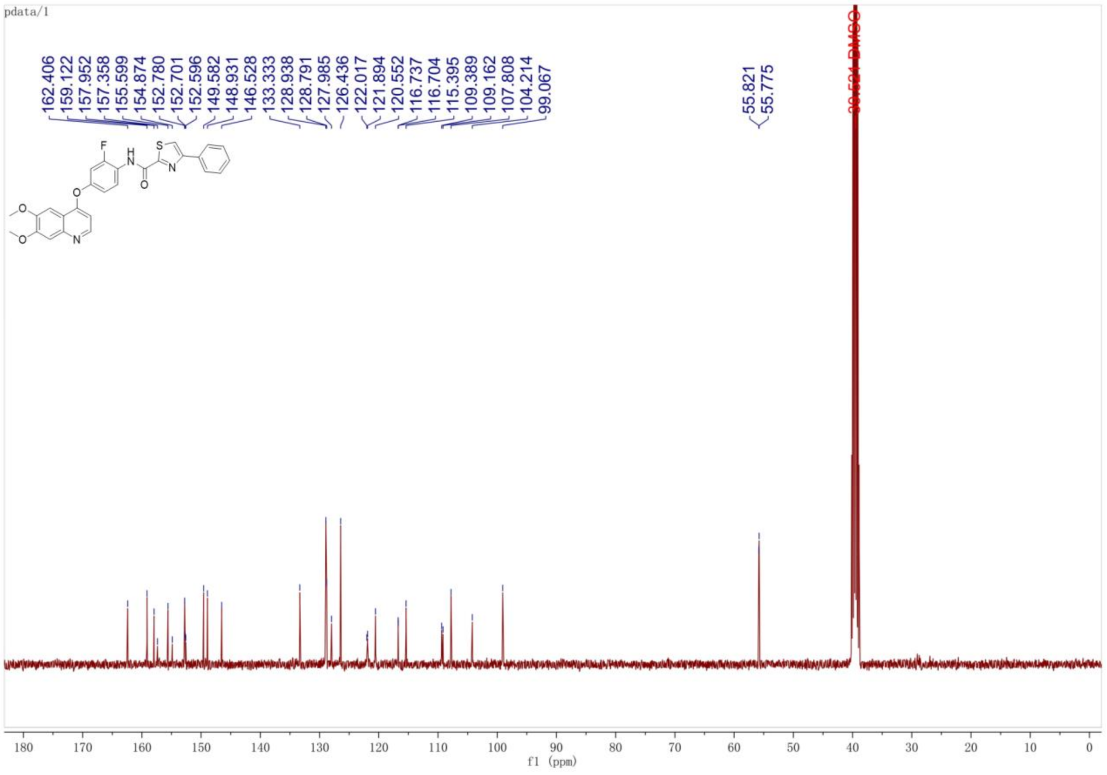

^{1}H NMR Spectrum of **51j** (400 MHz, DMSO-d_6)

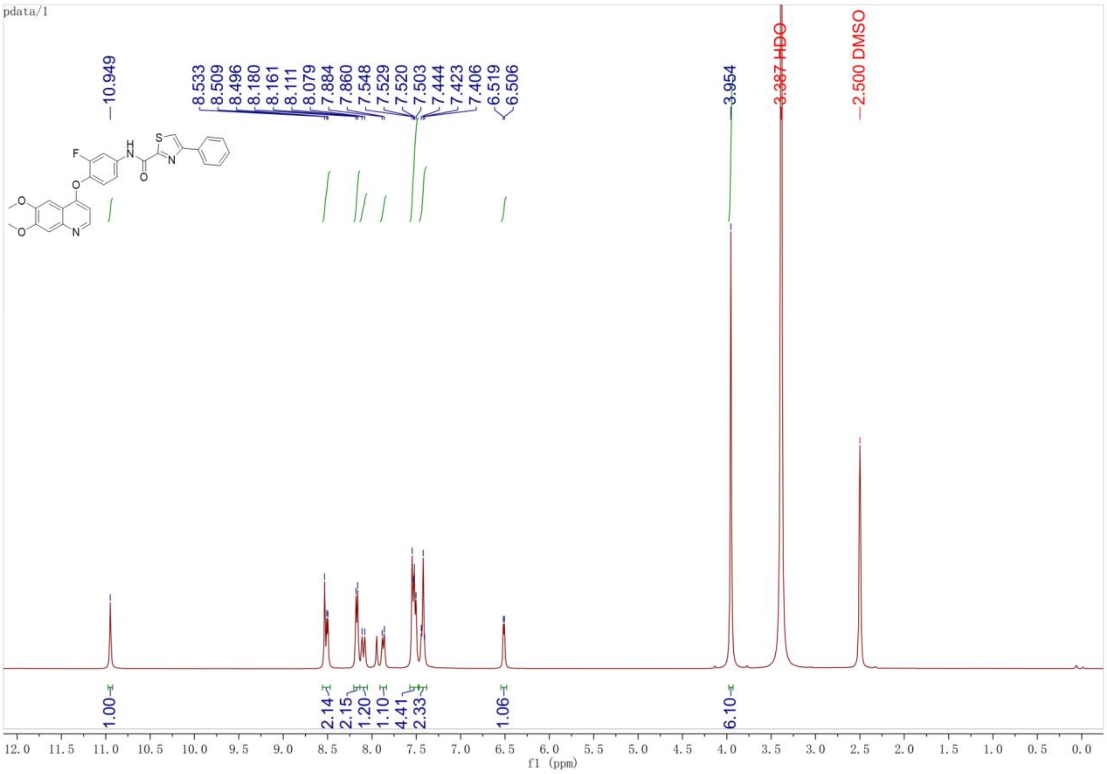

^{13}C NMR Spectrum of **51j** (100 MHz, DMSO-d_6)

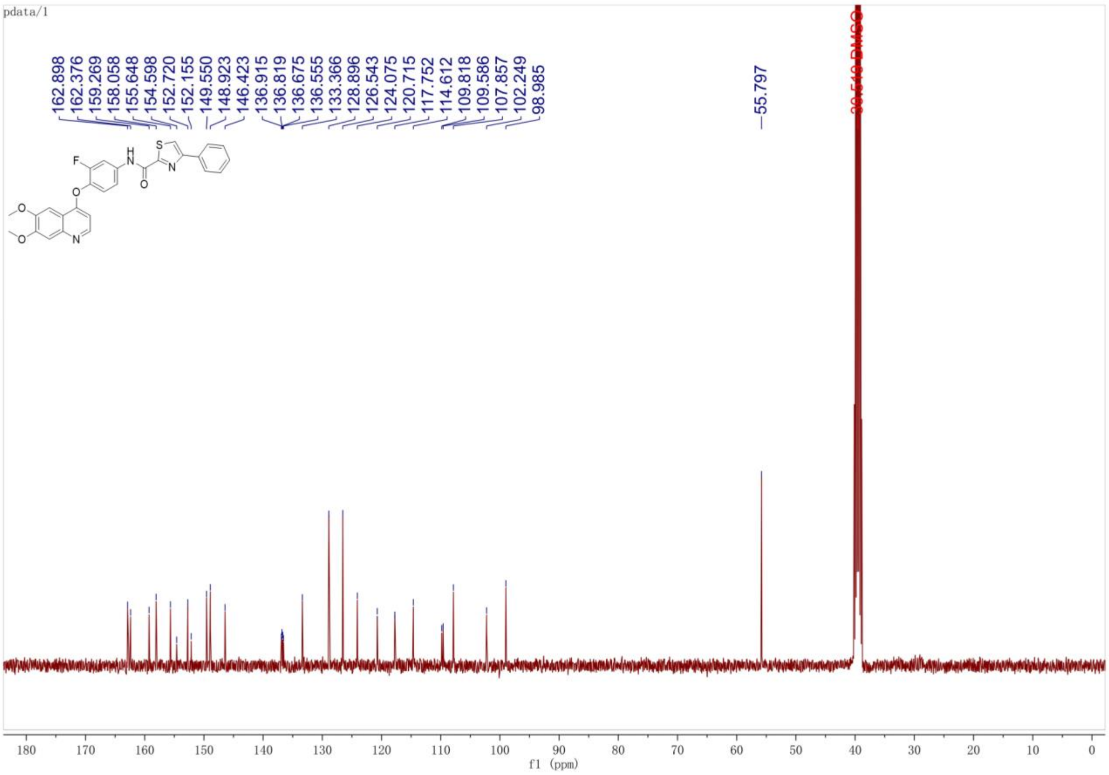

^{1}H NMR Spectrum of **51k** (600 MHz, DMSO-d_6)

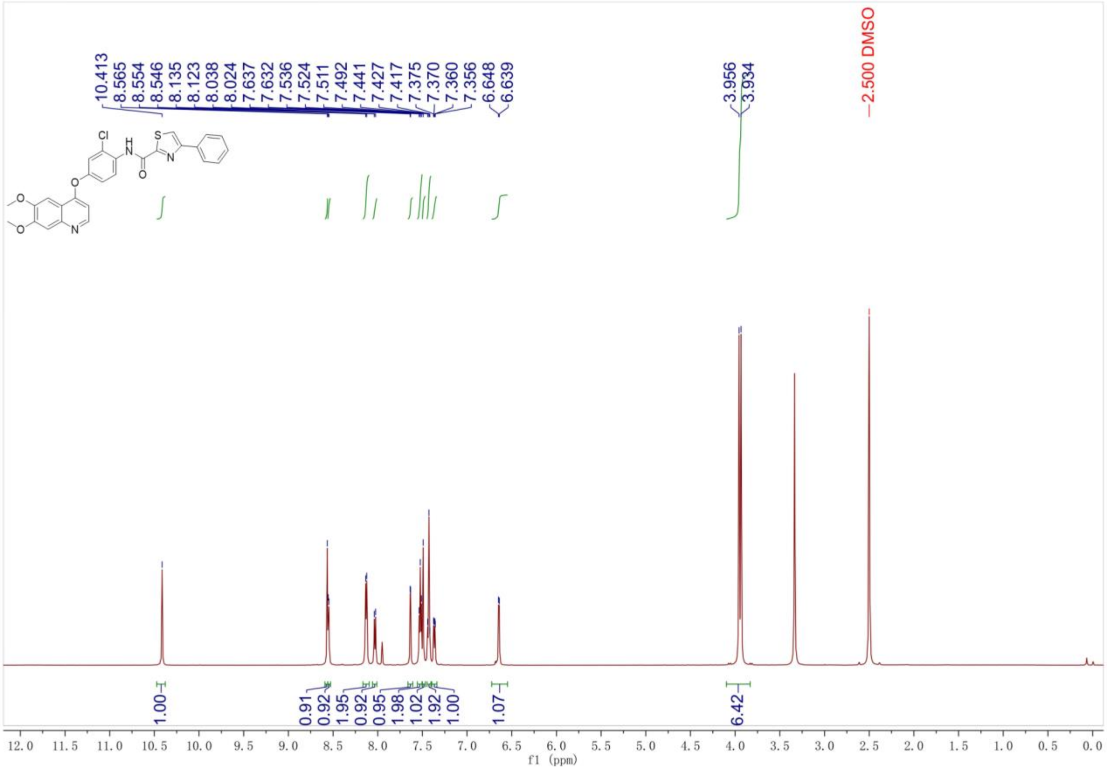

^{13}C NMR Spectrum of **51k** (150 MHz, DMSO-d_6)

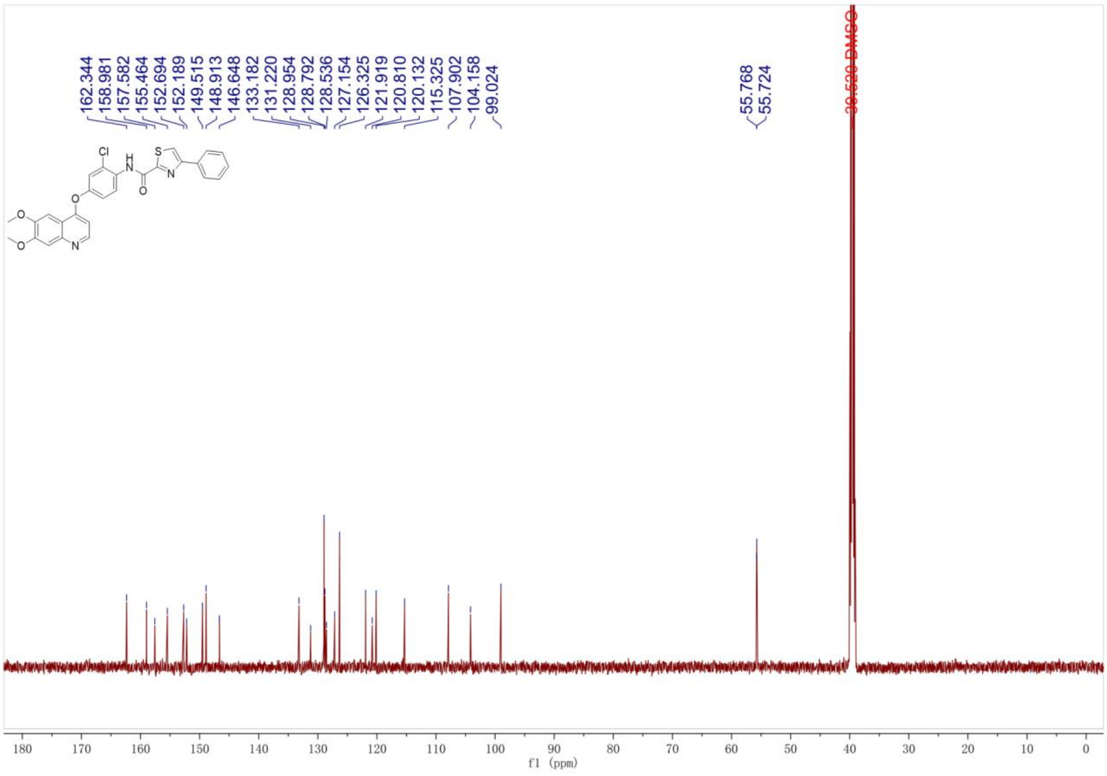

^{1}H NMR Spectrum of **51l** (600 MHz, DMSO-d_6)

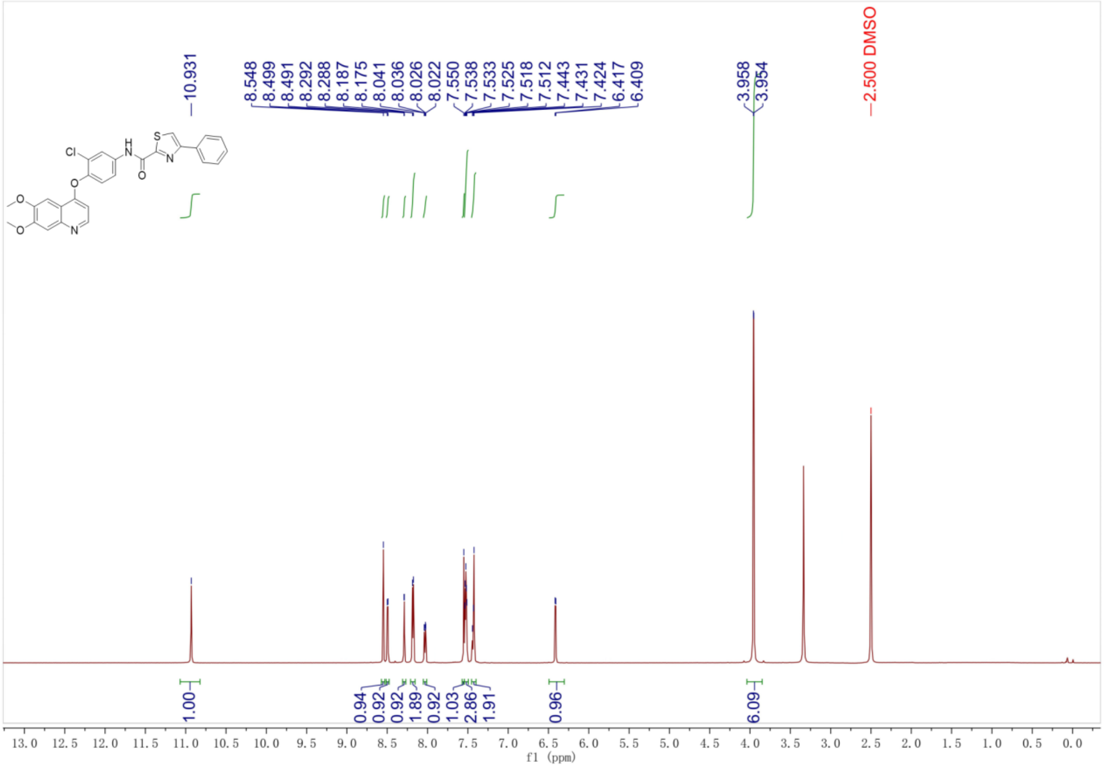

^{13}C NMR Spectrum of **51l** (150 MHz, DMSO-d_6)

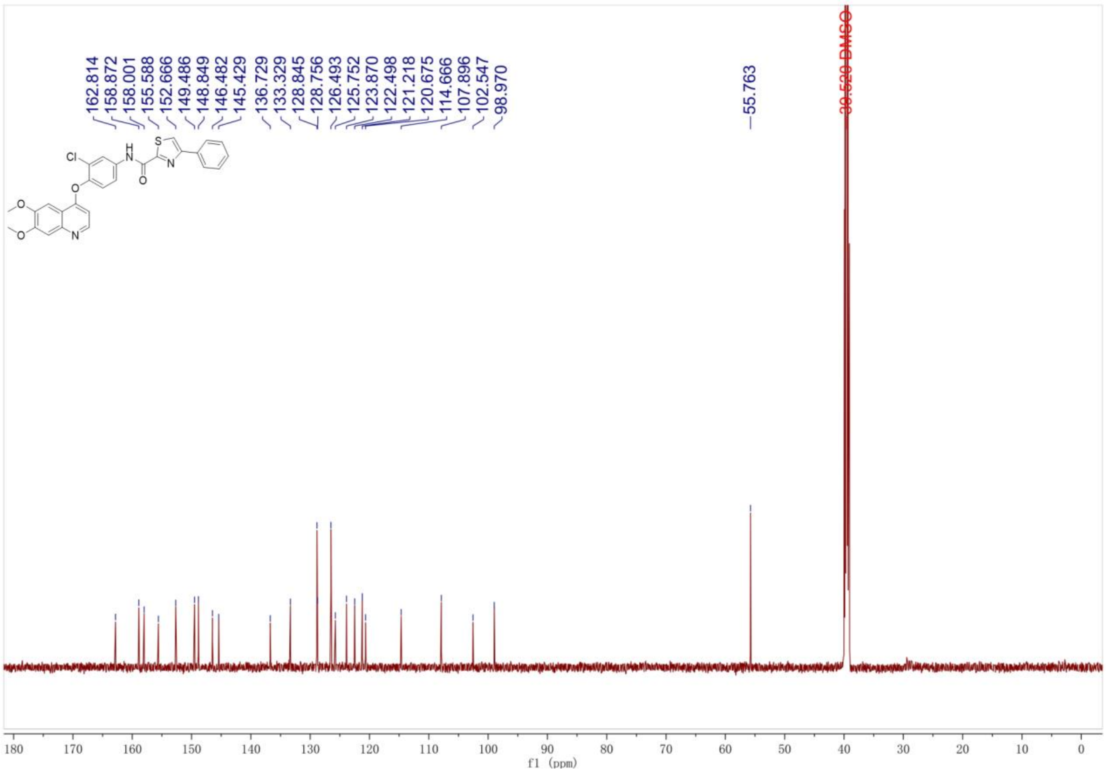

^{1}H NMR Spectrum of **51m** (600 MHz, DMSO-d_6)

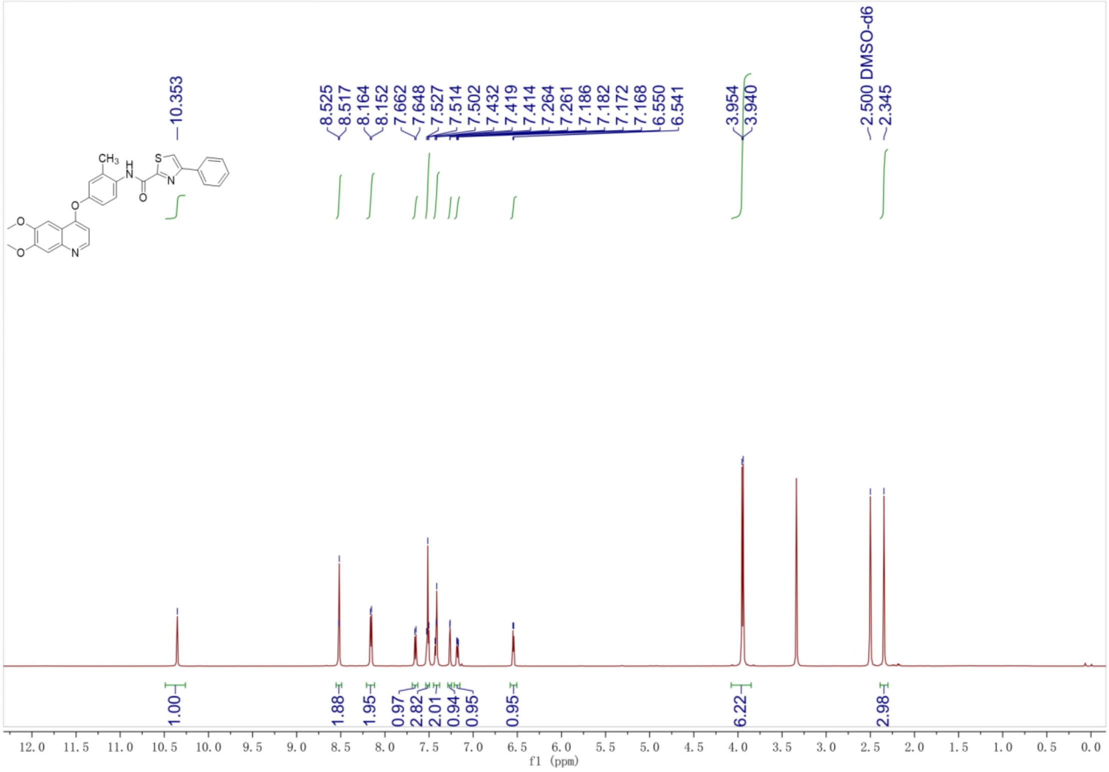

^{13}C NMR Spectrum of **51m** (150 MHz, DMSO-d_6)

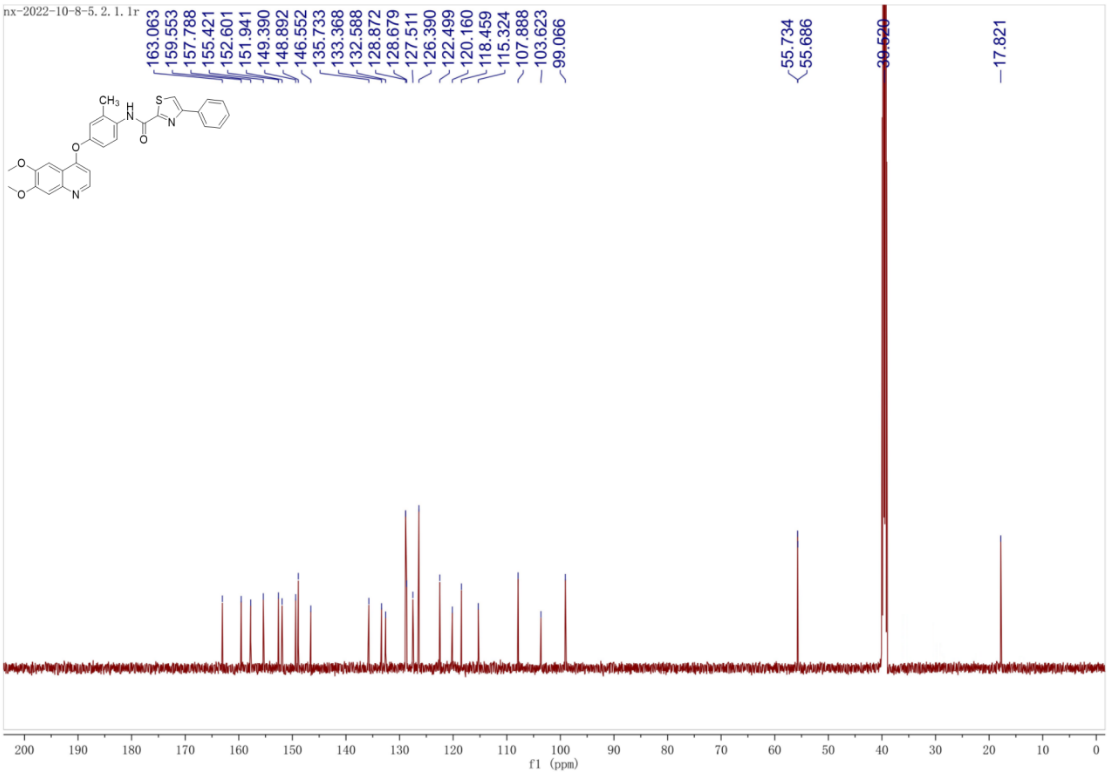

^{1}H NMR Spectrum of **51n** (600 MHz, DMSO-d_6)

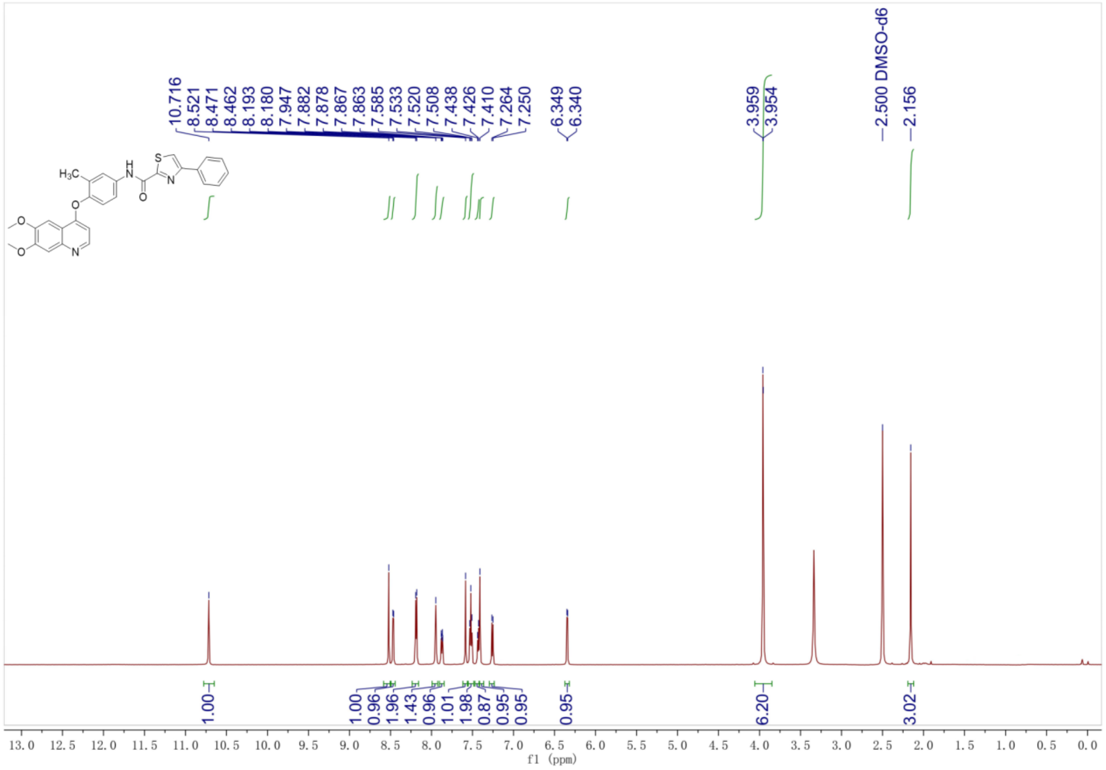

^{13}C NMR Spectrum of **51n** (150 MHz, DMSO-d_6)

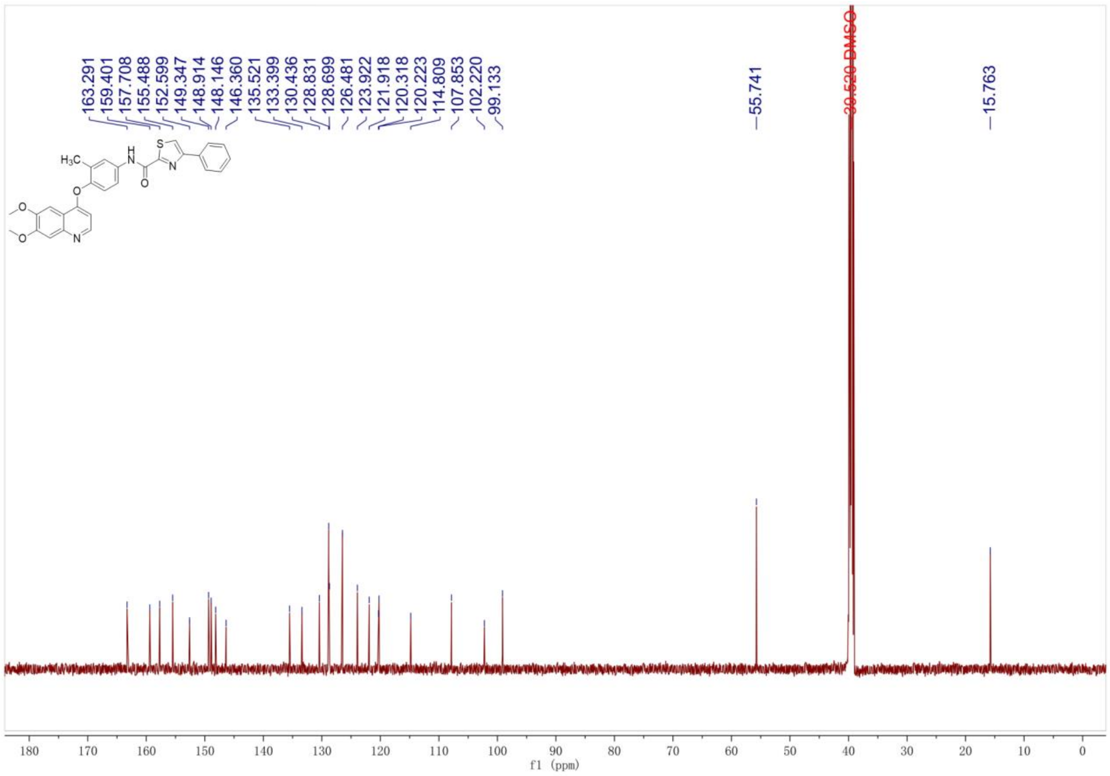

^{1}H NMR Spectrum of **51o** (400 MHz, DMSO-d_6)

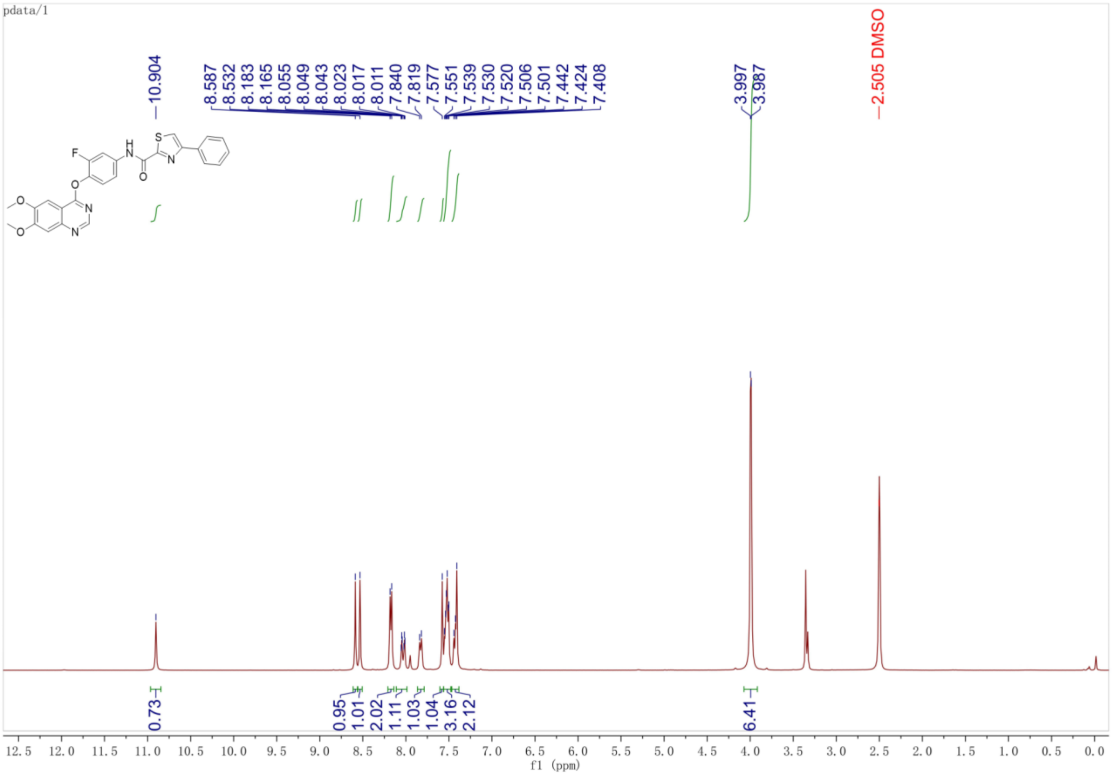

^{13}C NMR Spectrum of **51o** (100 MHz, DMSO-d_6)

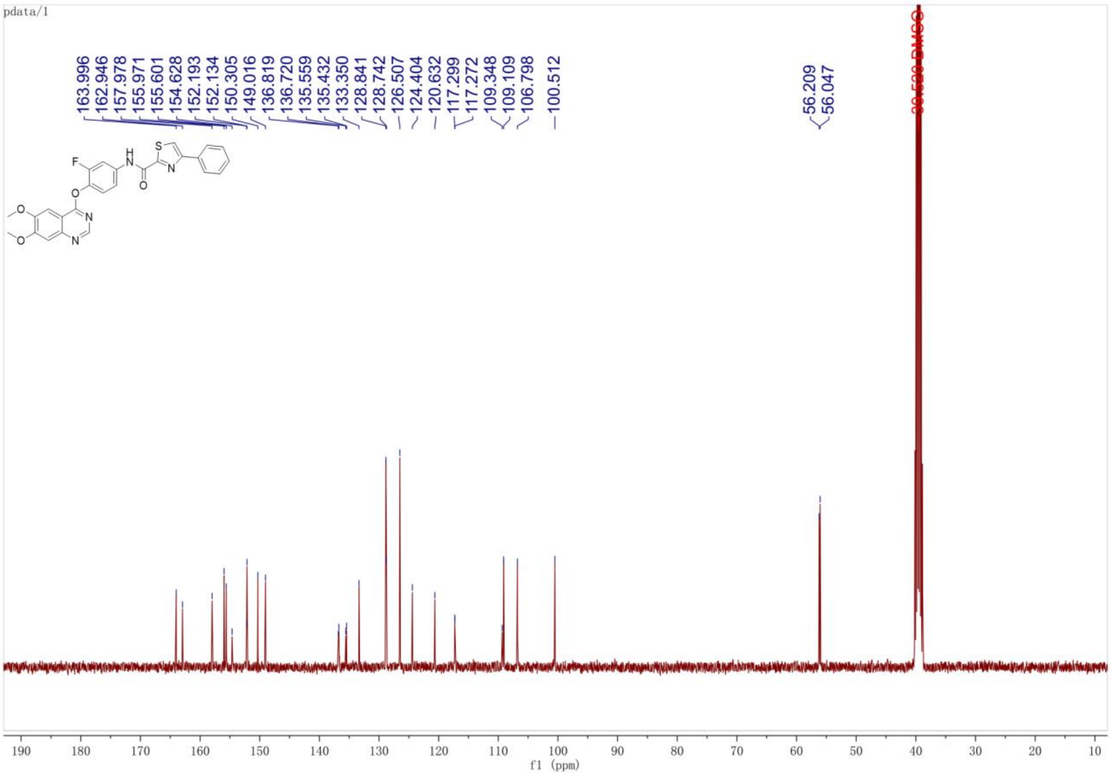

^{1}H NMR Spectrum of **51p** (400 MHz, DMSO-d_6)

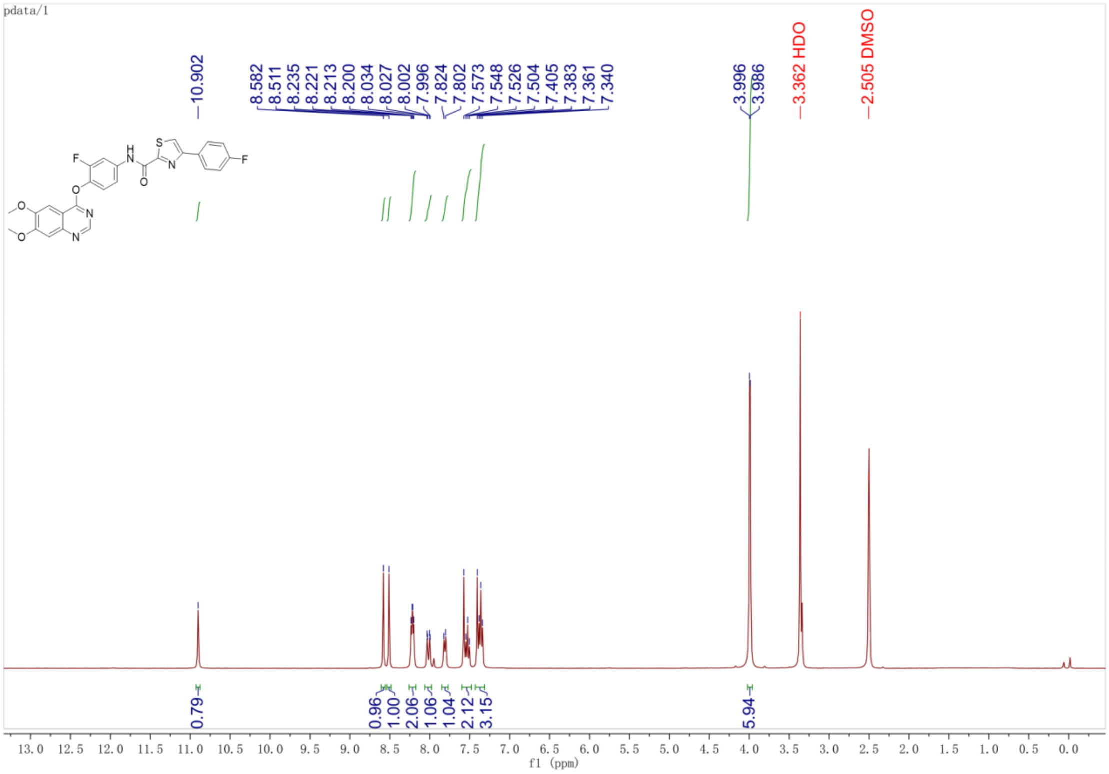

^{13}C NMR Spectrum of **51p** (100 MHz, DMSO-d_6)

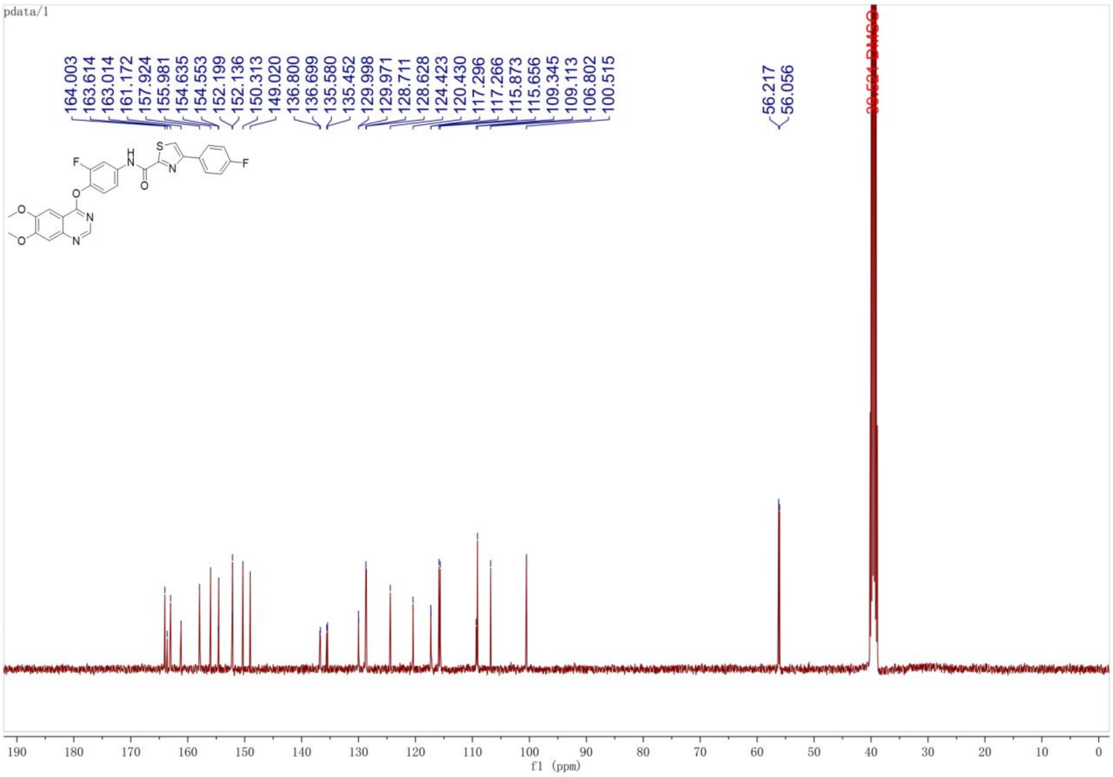

^{1}H NMR Spectrum of **51q** (400 MHz, DMSO-d_6)

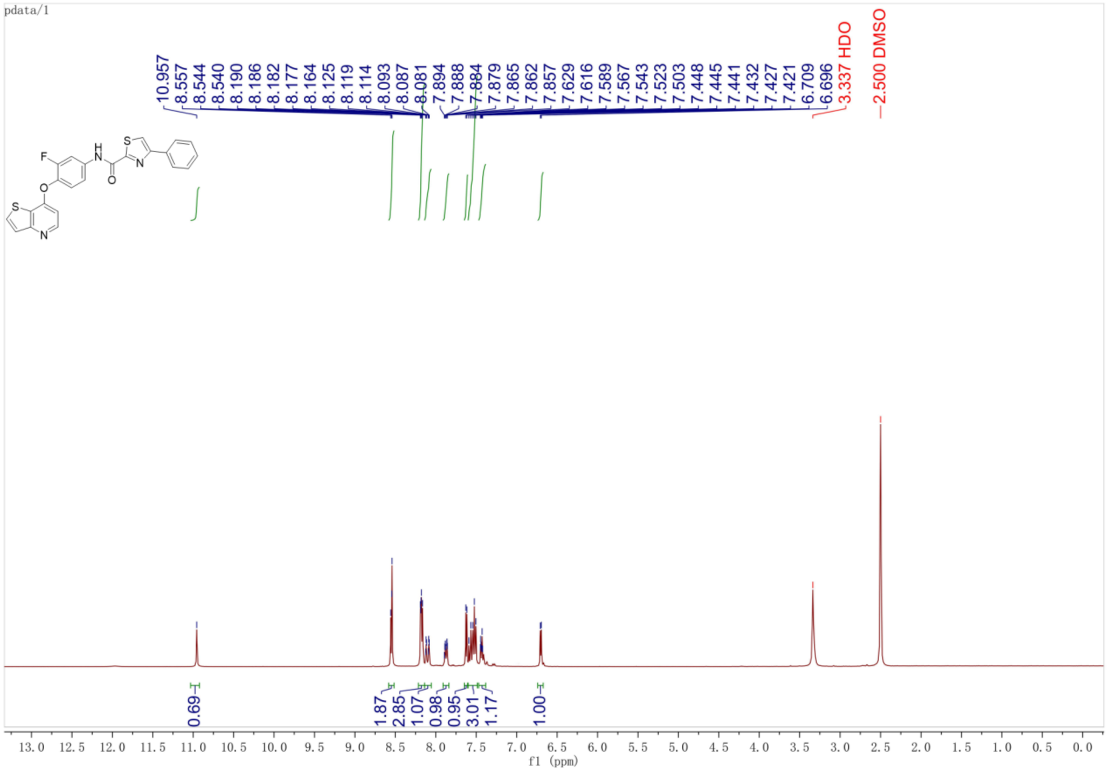

^{13}C NMR Spectrum of **51q** (100 MHz, DMSO-d_6)

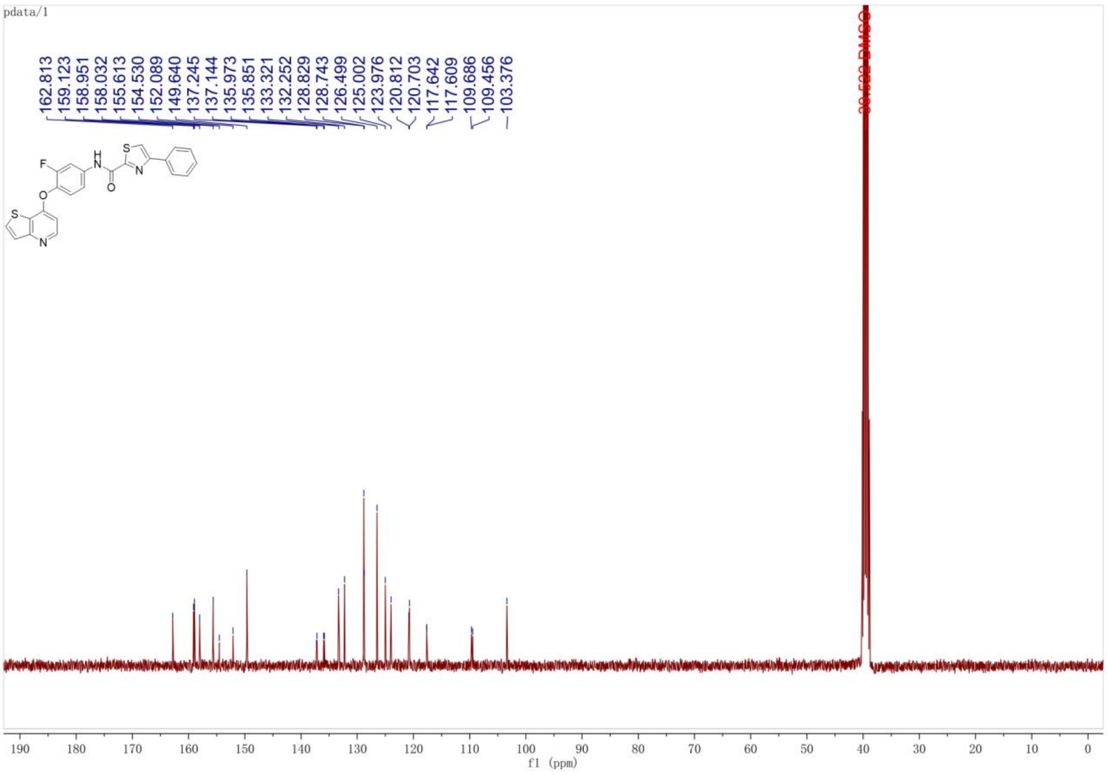

^{1}H NMR Spectrum of **51r** (400 MHz, DMSO-d_6)

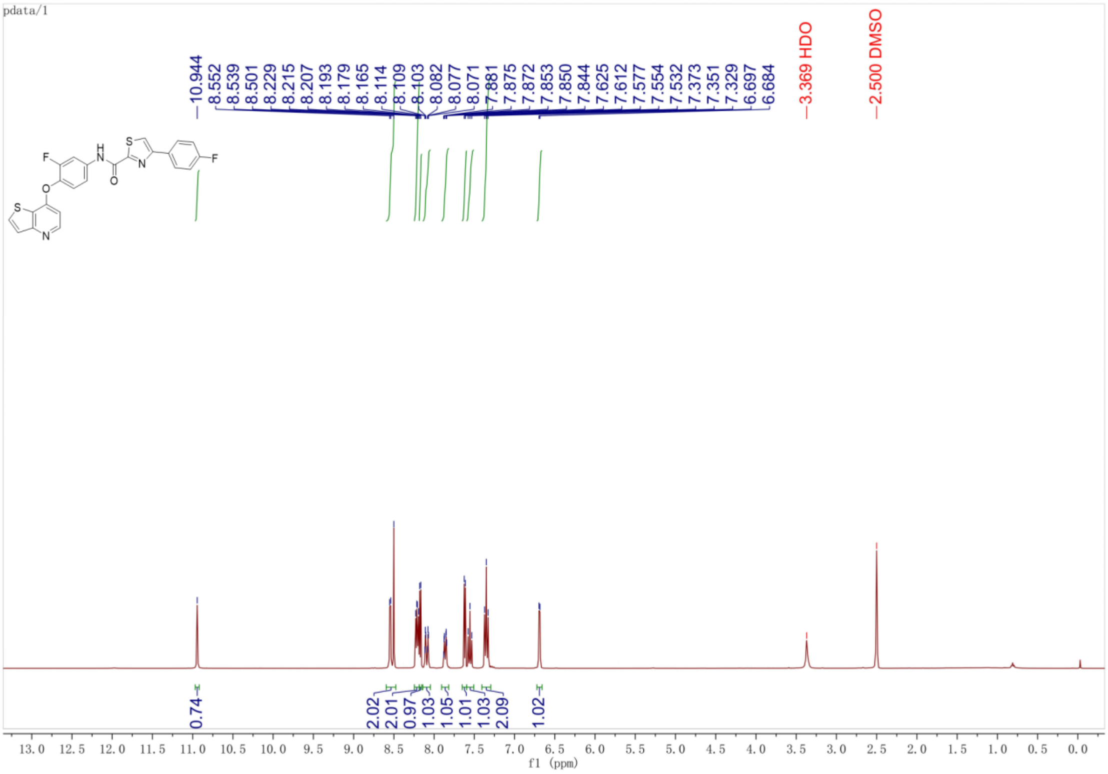

^{13}C NMR Spectrum of **51r** (100 MHz, DMSO-d_6)

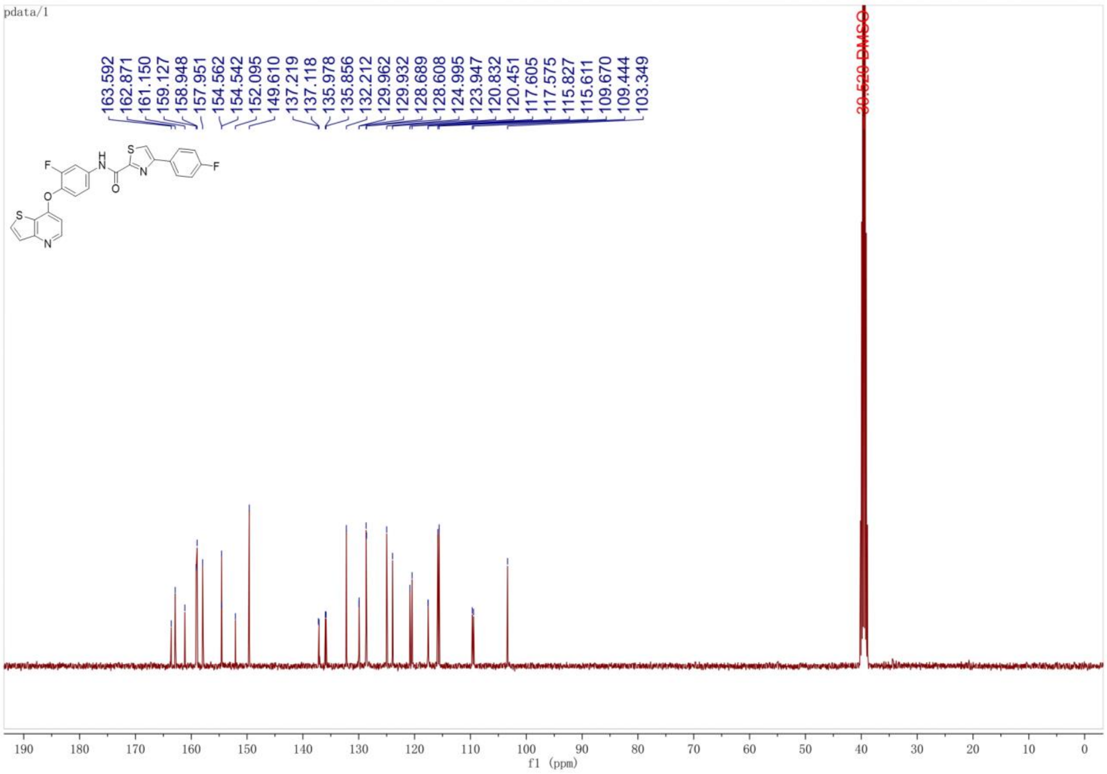

^{1}H NMR Spectrum of **51s** (400 MHz, DMSO-d_6)

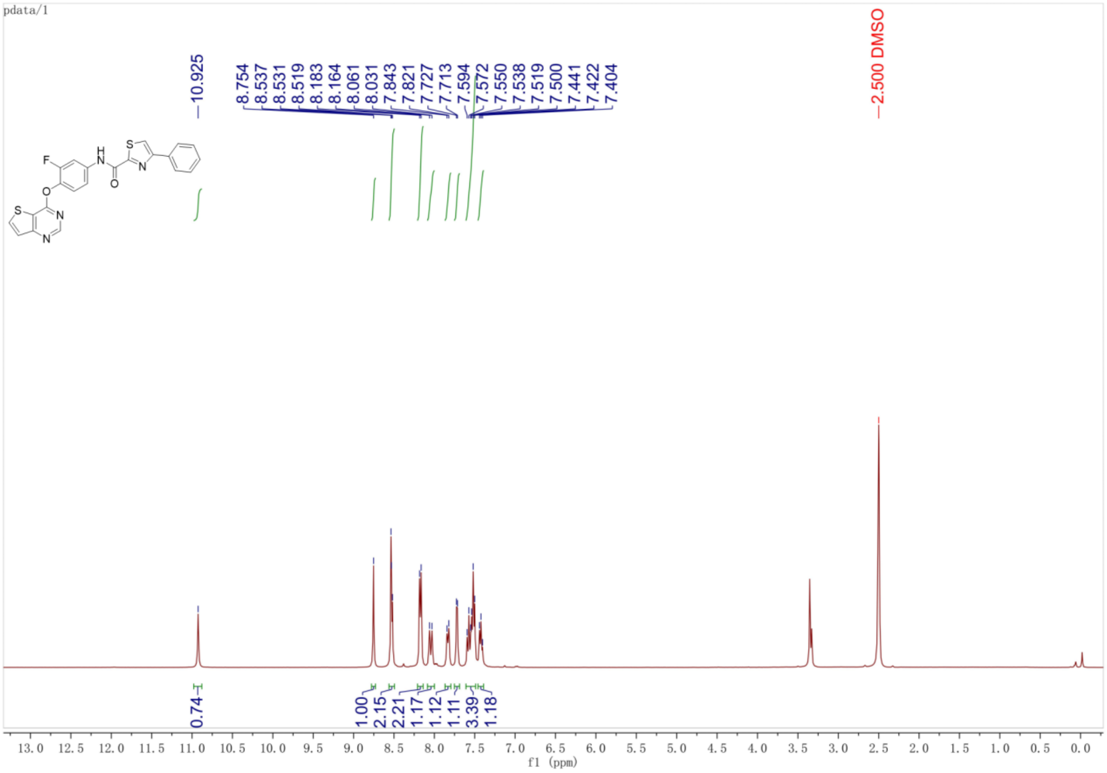

^{13}C NMR Spectrum of **51s** (100 MHz, DMSO-d_6)

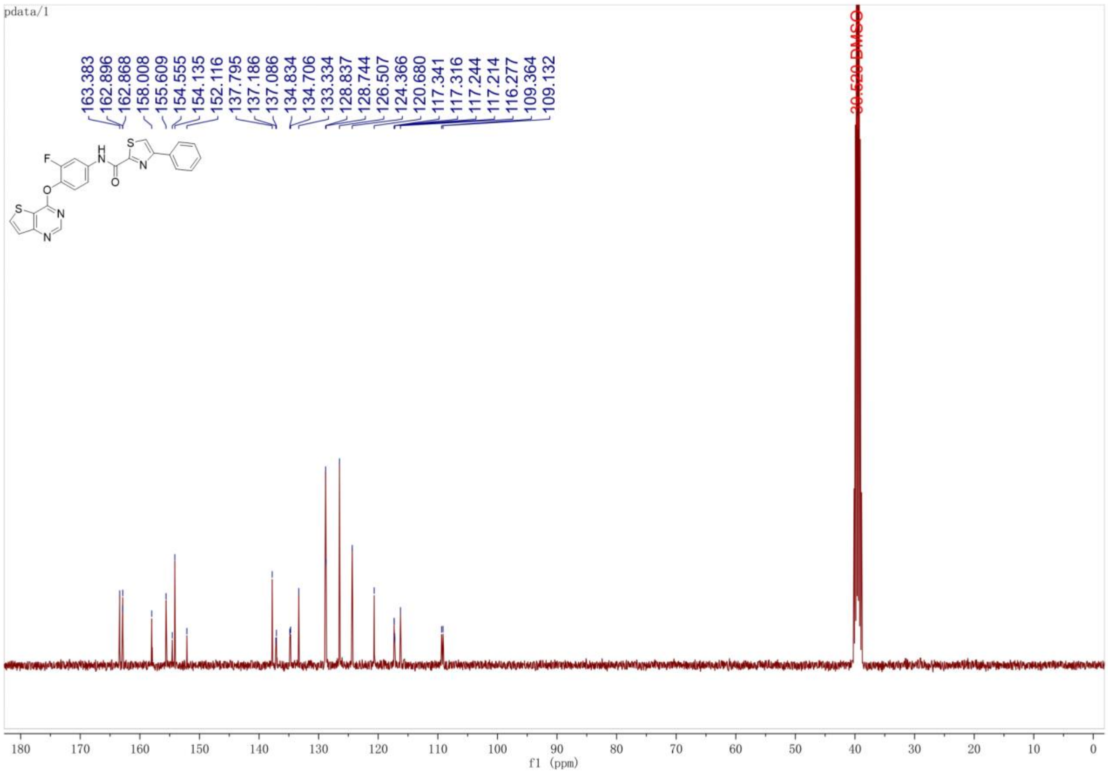

^{1}H NMR Spectrum of **51t** (400 MHz, DMSO-d_6)

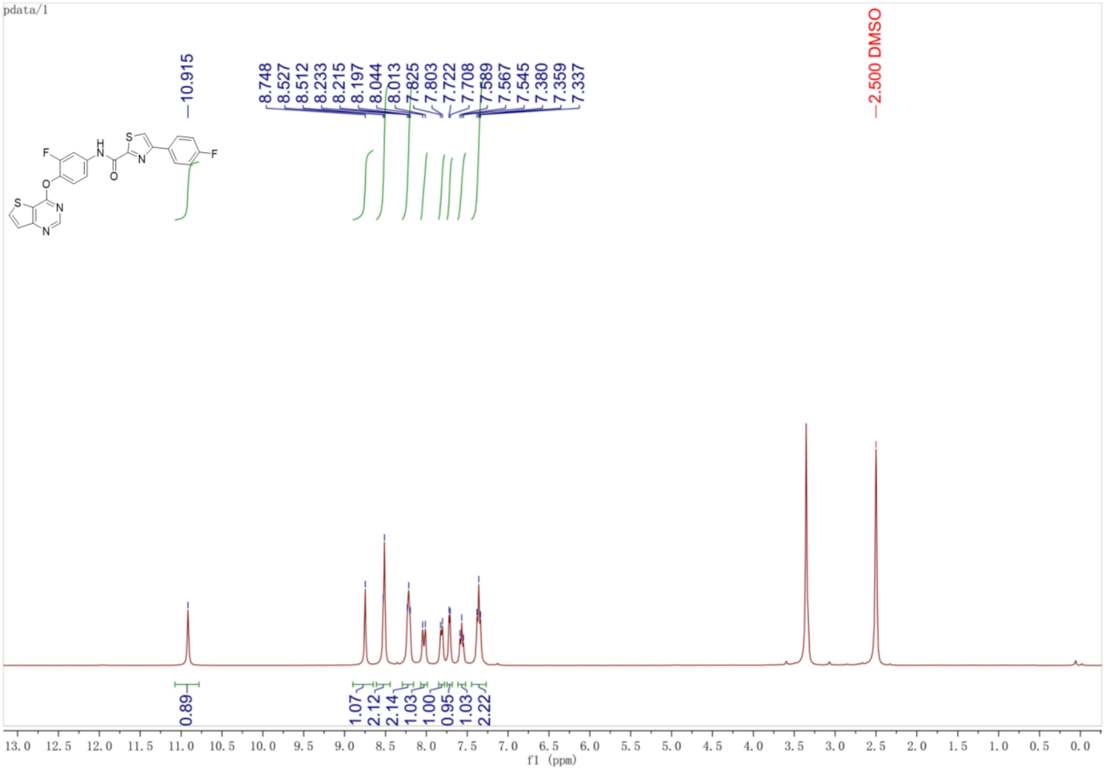

^{13}C NMR Spectrum of **51t** (100 MHz, DMSO-d_6)

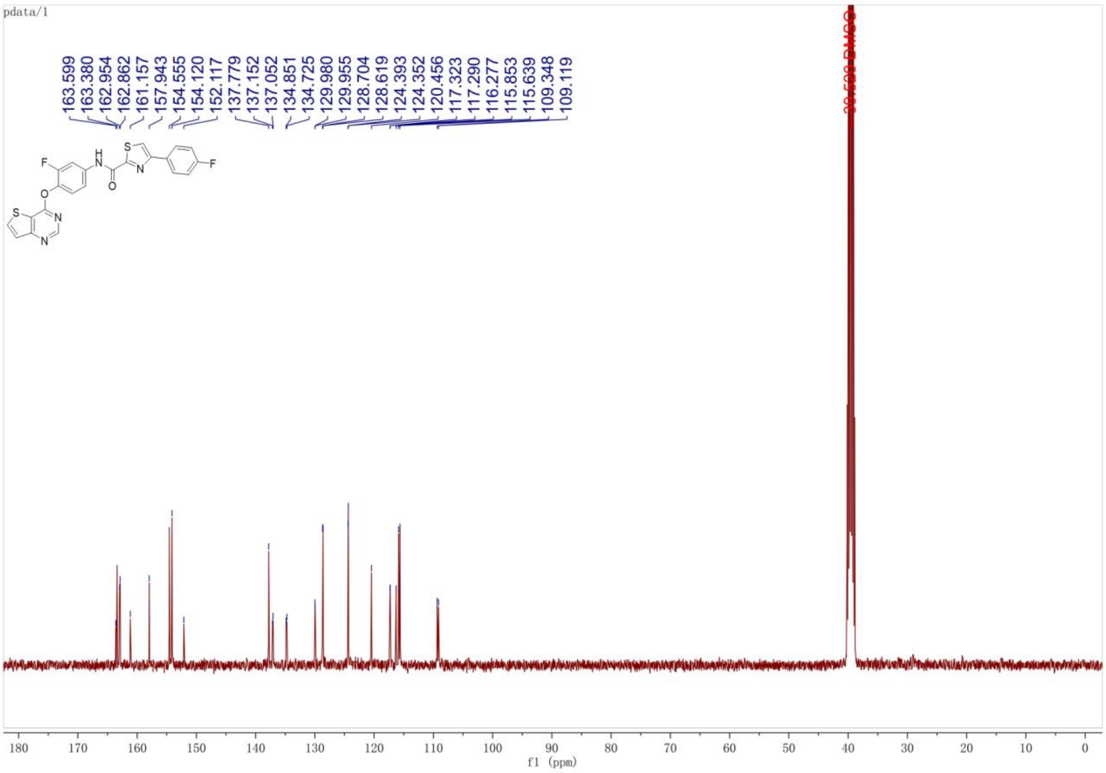

^{1}H NMR Spectrum of **51u** (400 MHz, DMSO-d_6)

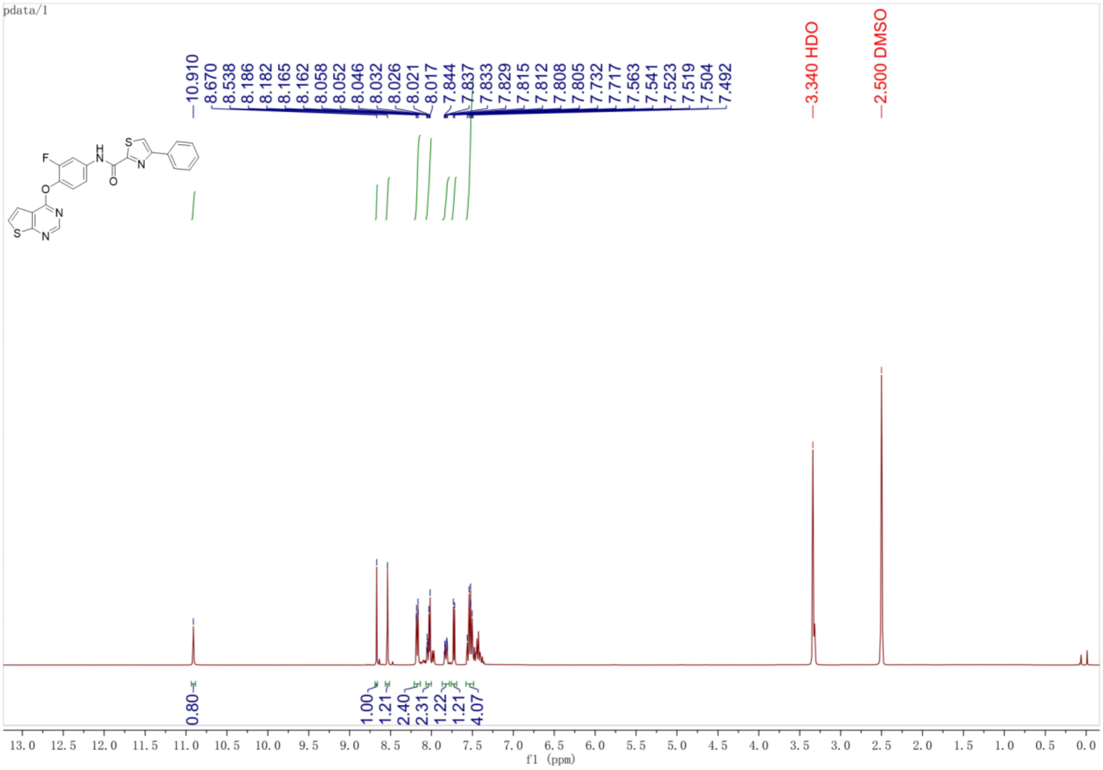

^{13}C NMR Spectrum of **51u** (100 MHz, DMSO-d_6)

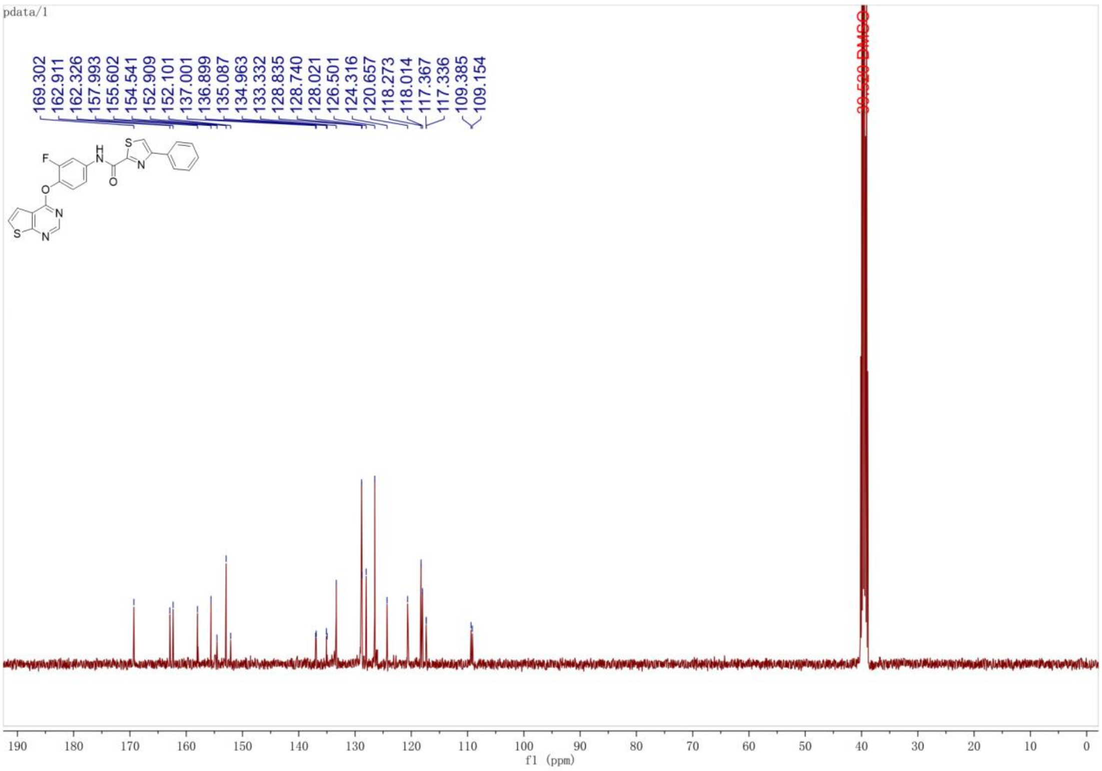

^{1}H NMR Spectrum of **51v** (400 MHz, DMSO-d_6)

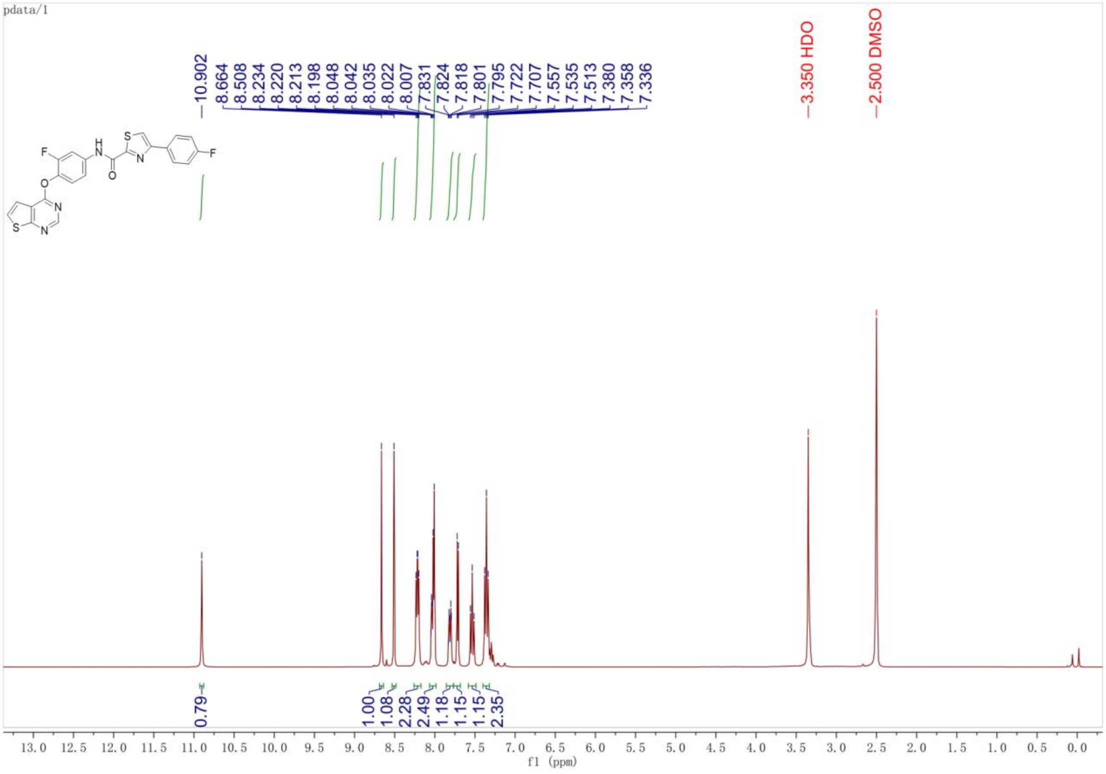

^{13}C NMR Spectrum of **51v** (100 MHz, DMSO-d_6)

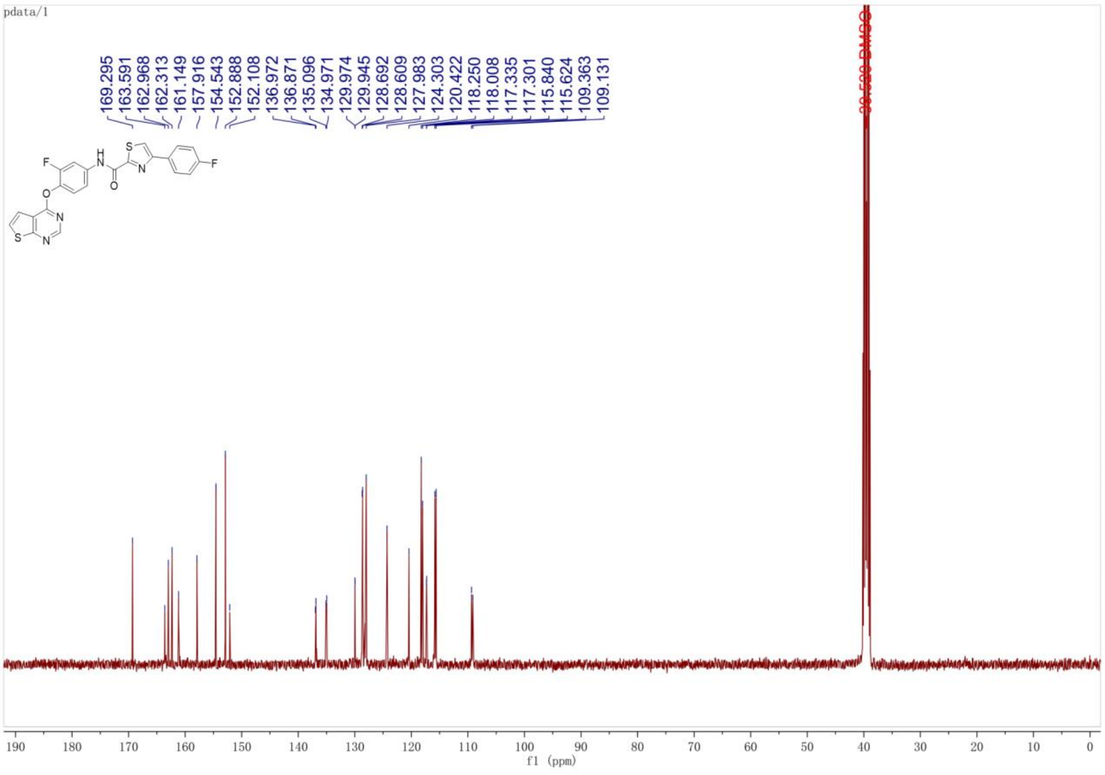

^{1}H NMR Spectrum of **51w** (400 MHz, DMSO-d_6)

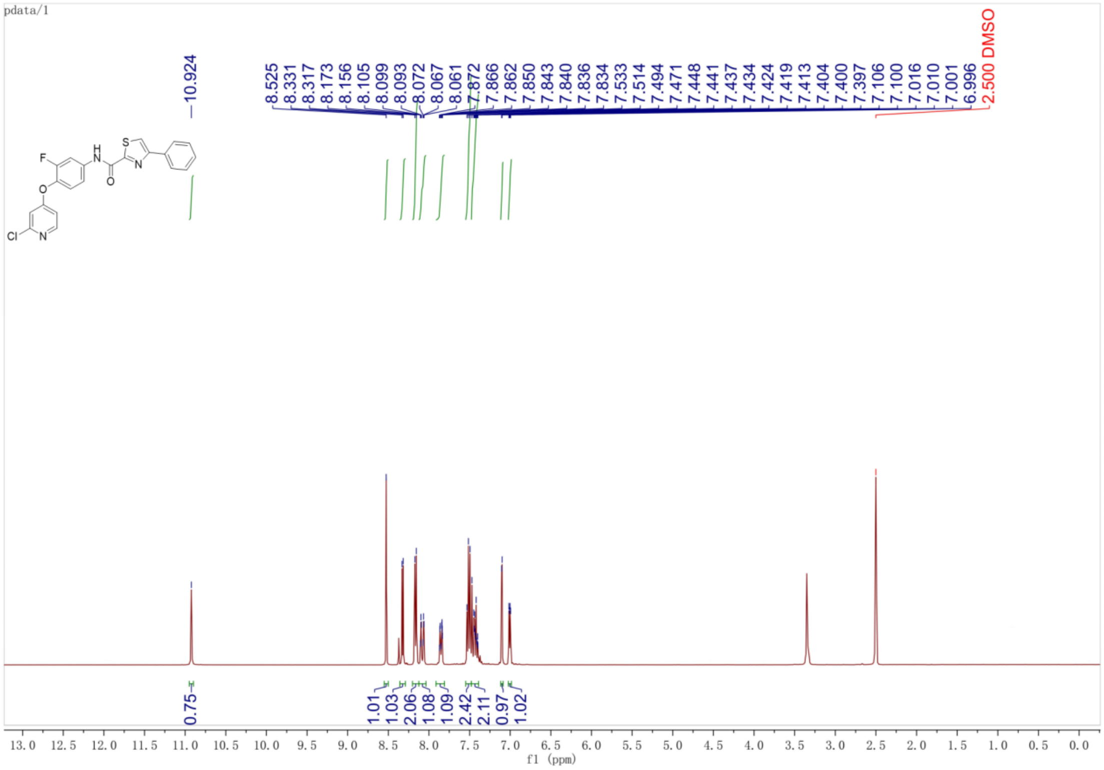

^{13}C NMR Spectrum of **51w** (100 MHz, DMSO-d_6)

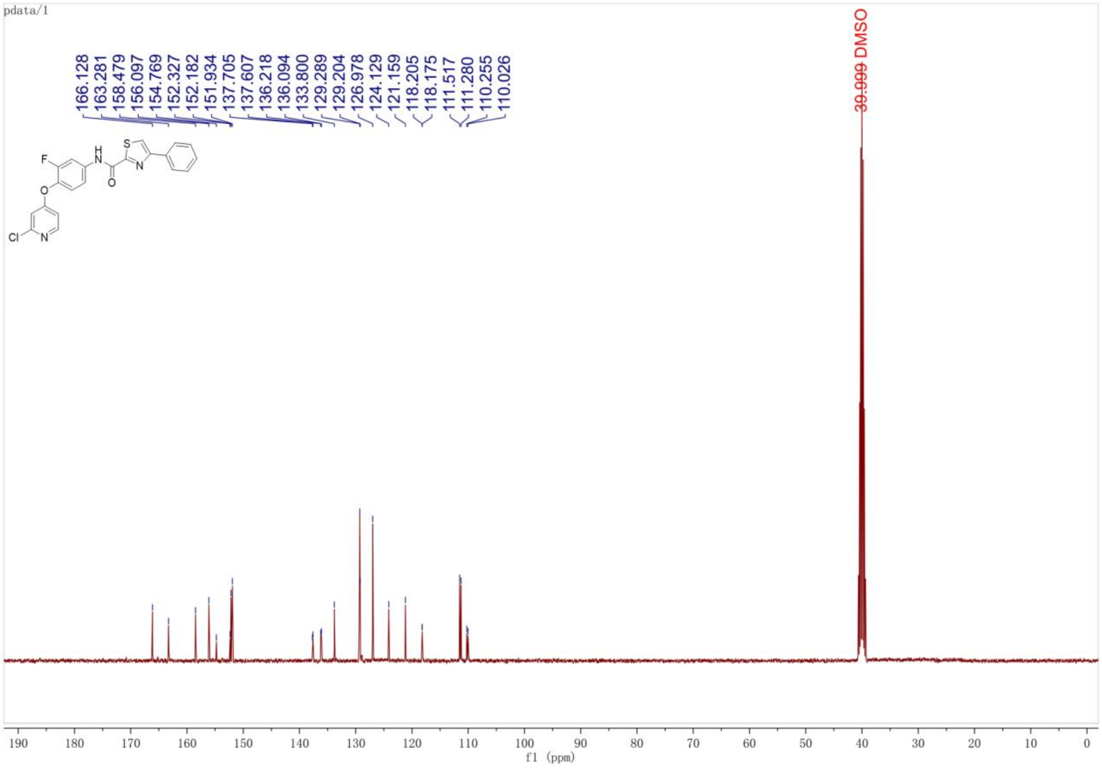

^{1}H NMR Spectrum of **51x** (400 MHz, DMSO-d_6)

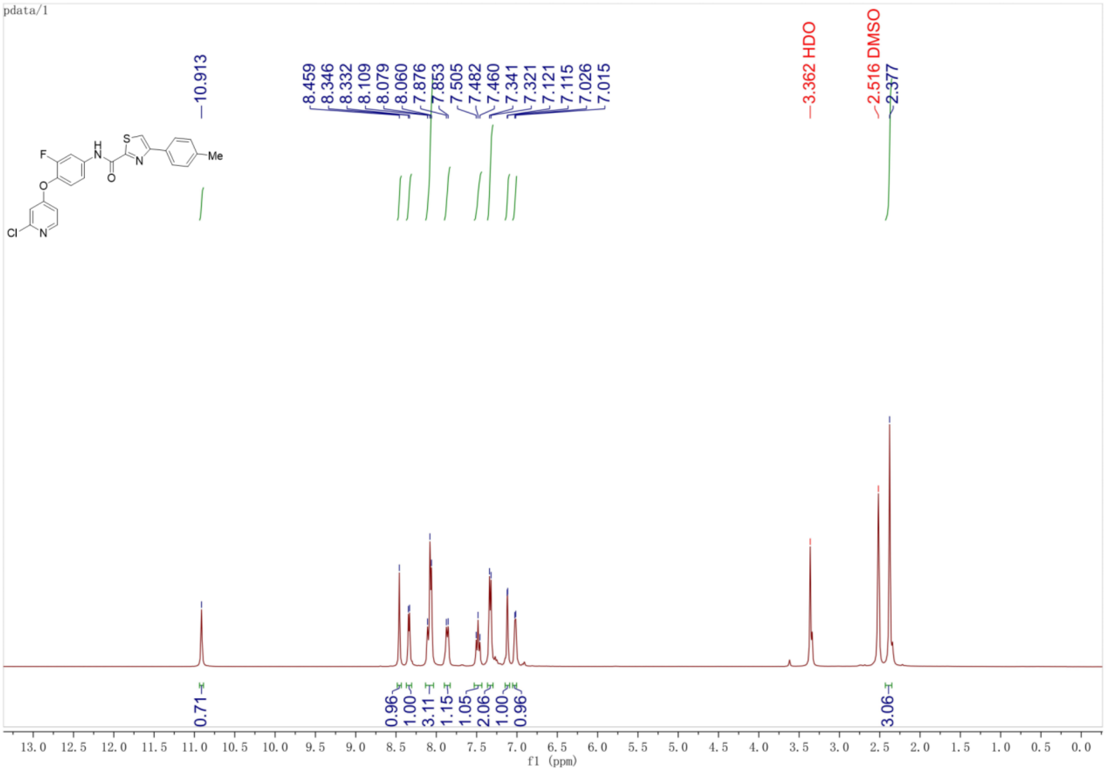

^{13}C NMR Spectrum of **51x** (100 MHz, DMSO-d_6)

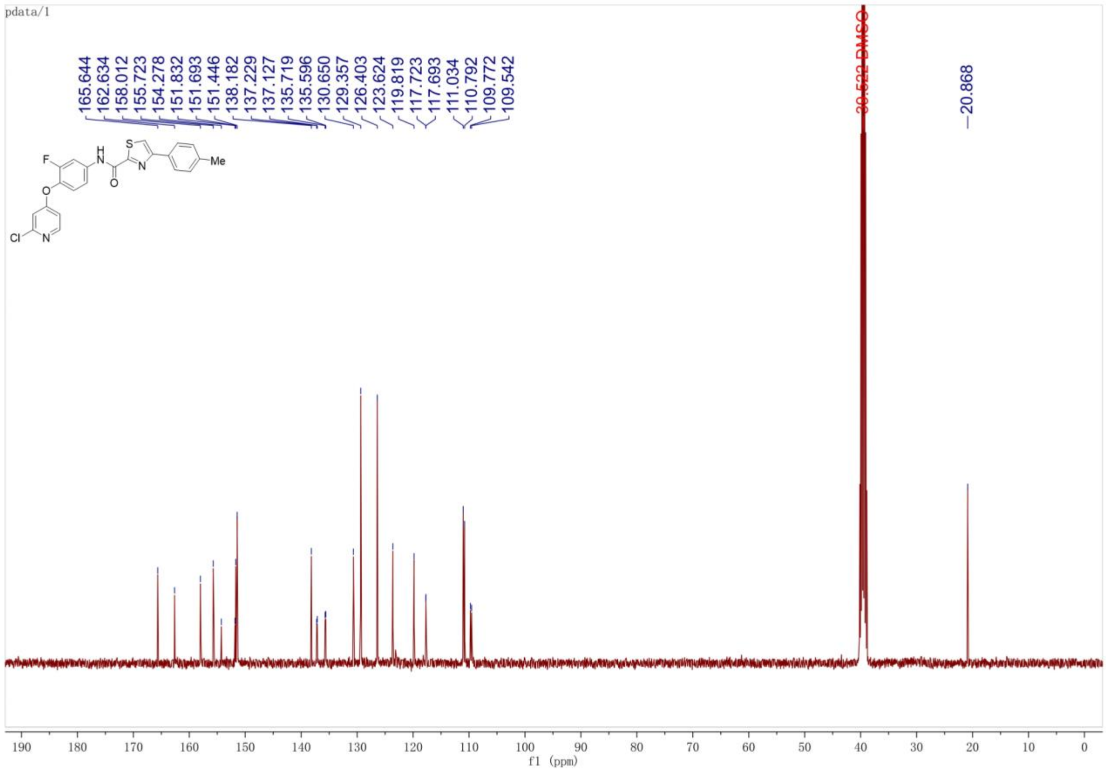

^{1}H NMR Spectrum of **51y** (400 MHz, DMSO-d_6)

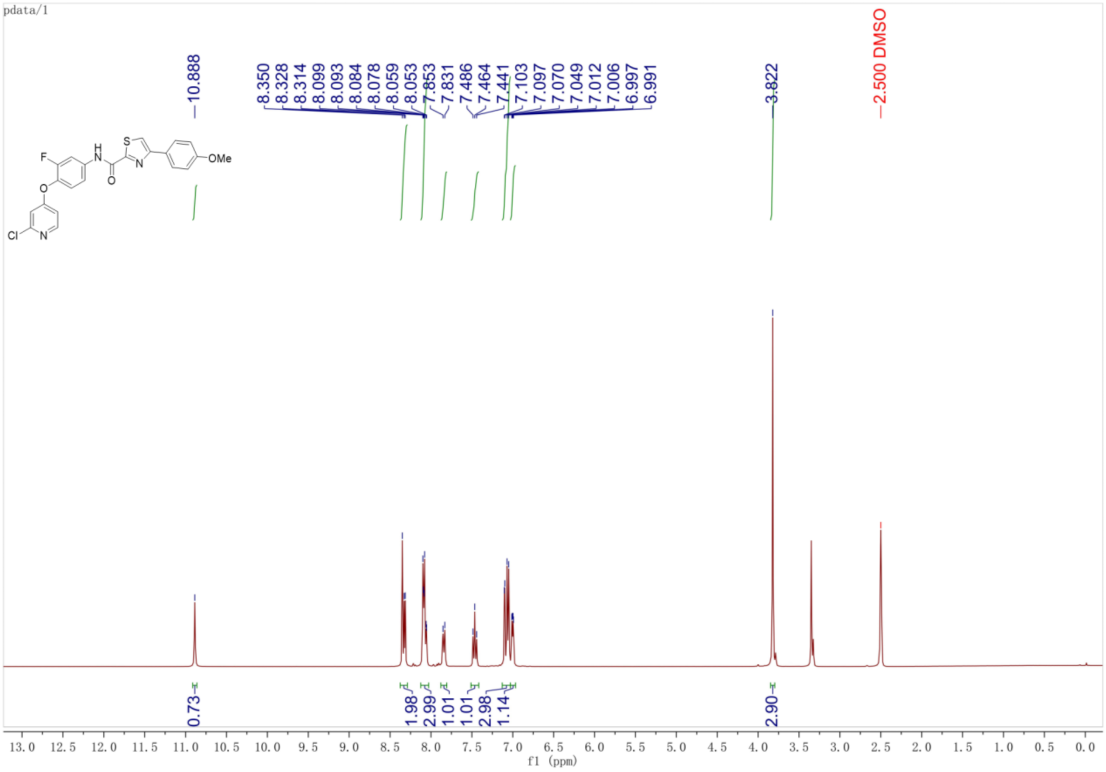

^{13}C NMR Spectrum of **51y** (100 MHz, DMSO-d_6)

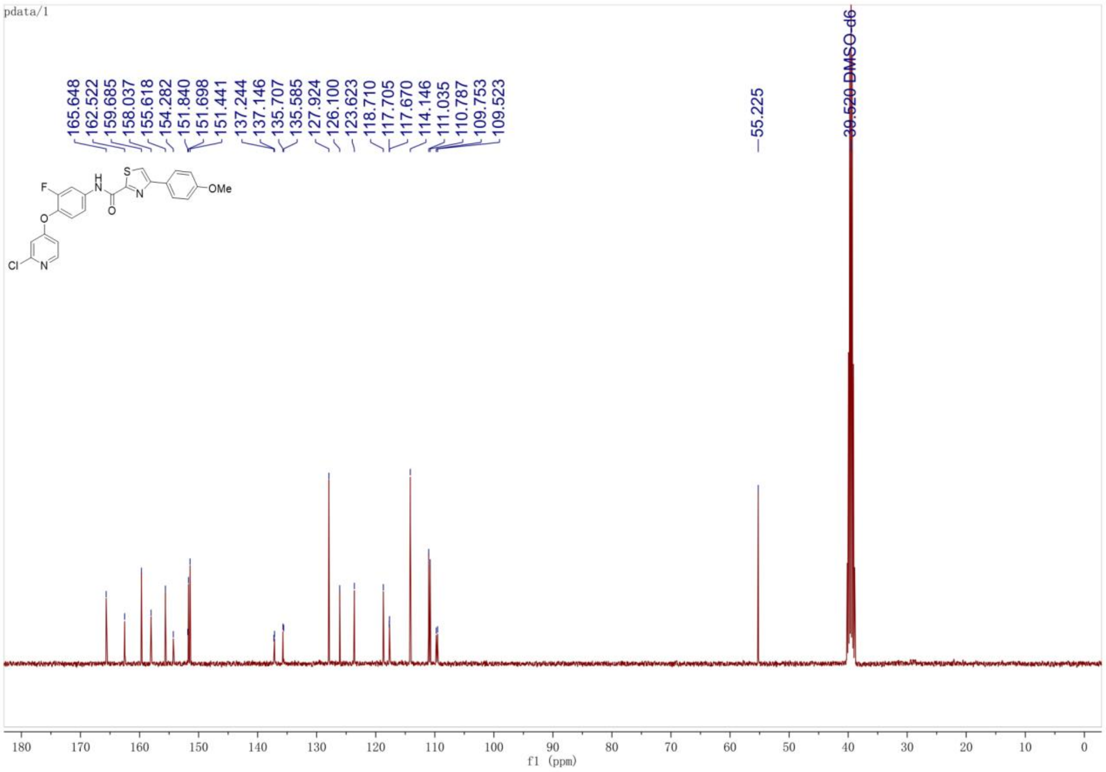

^{1}H NMR Spectrum of **51z** (400 MHz, DMSO-d_6)

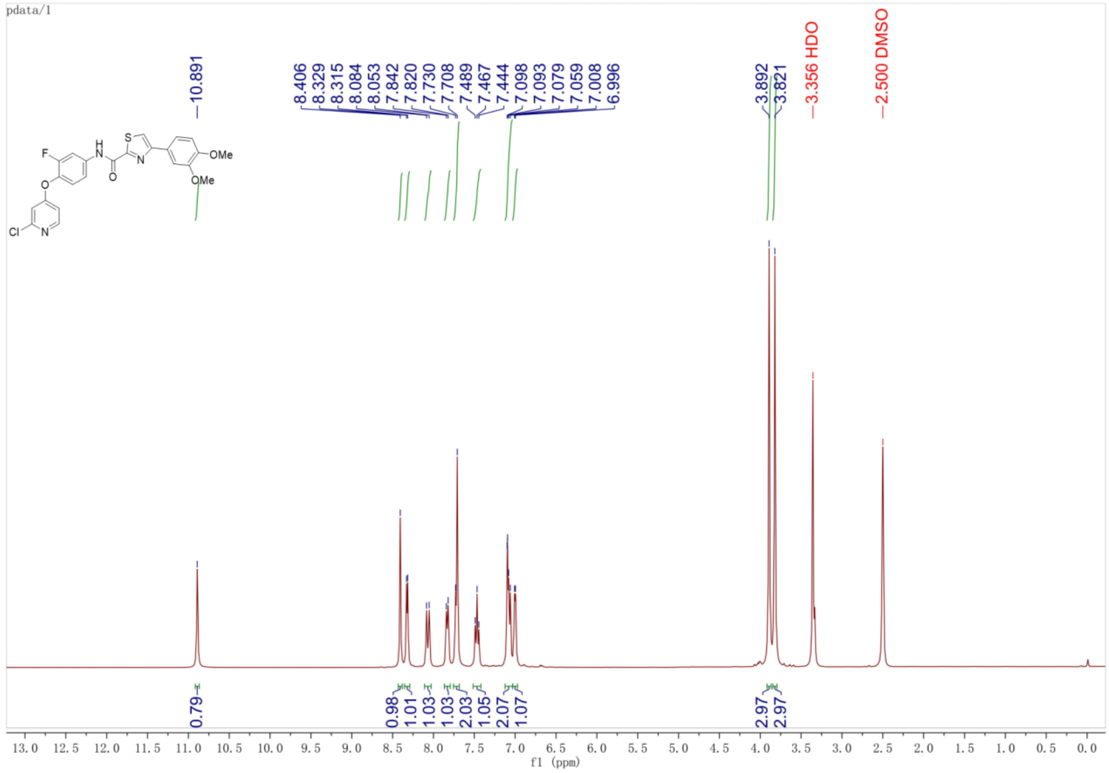

^{13}C NMR Spectrum of **51z** (100 MHz, DMSO-d_6)

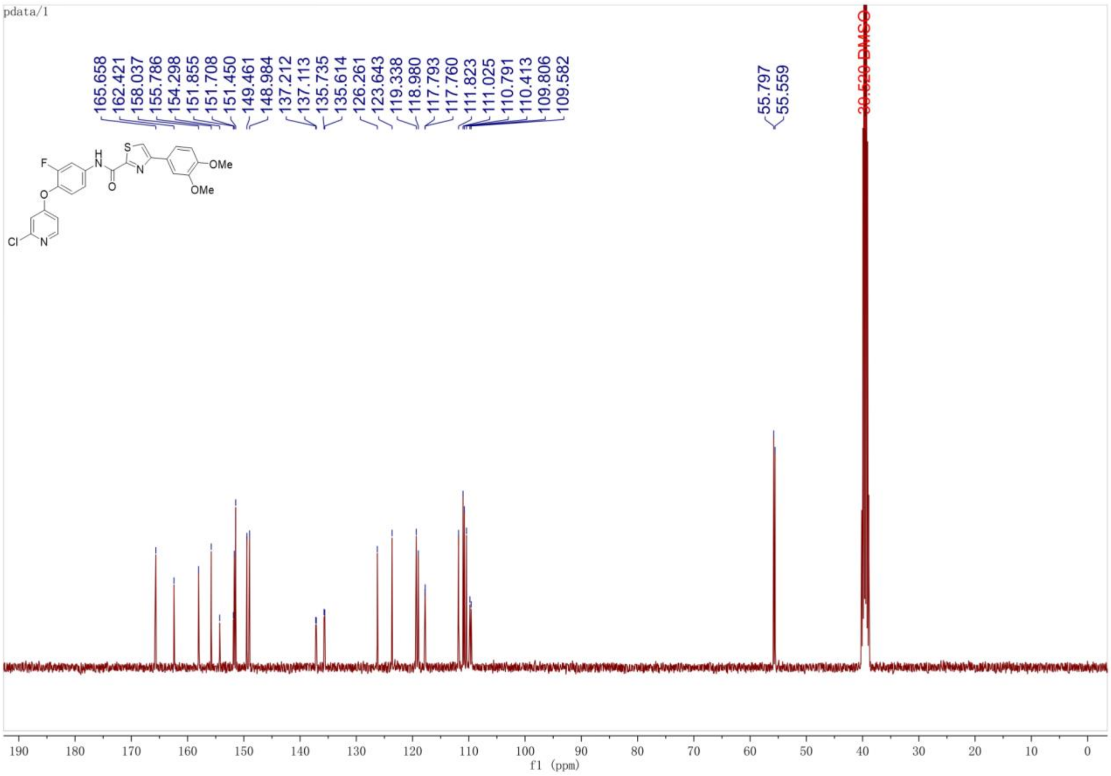

^{1}H NMR Spectrum of **51aa** (400 MHz, DMSO-d_6)

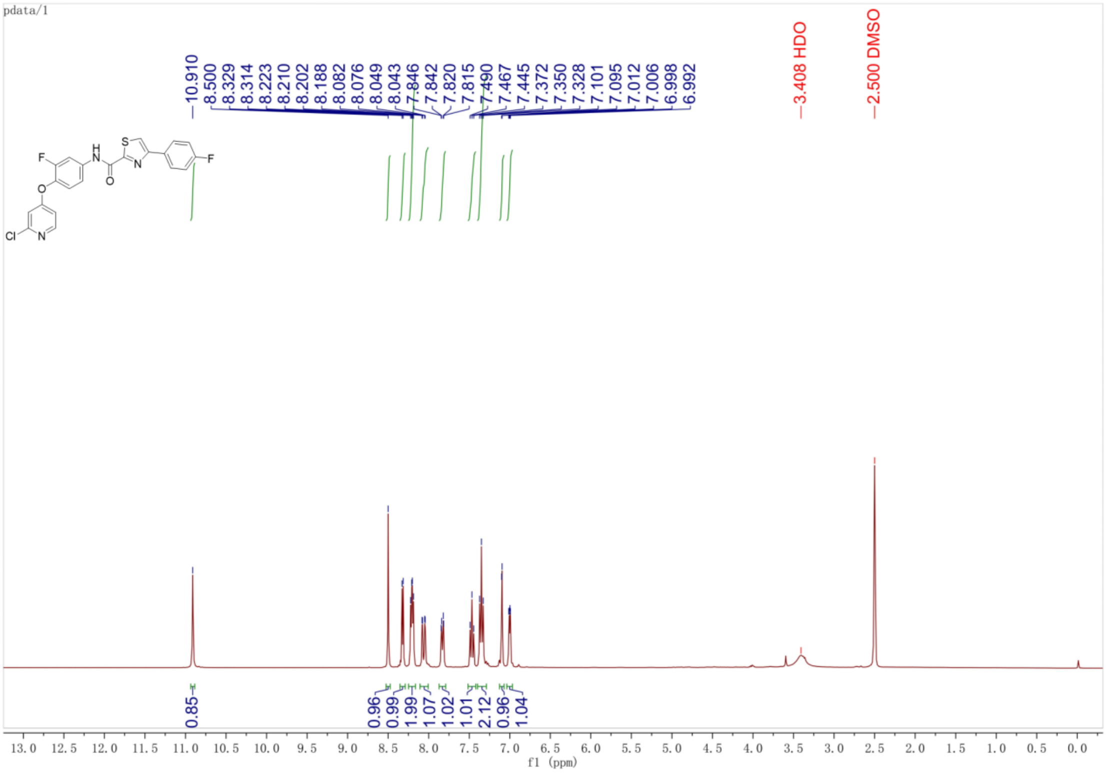

^{13}C NMR Spectrum of **51aa** (100 MHz, DMSO-d_6)

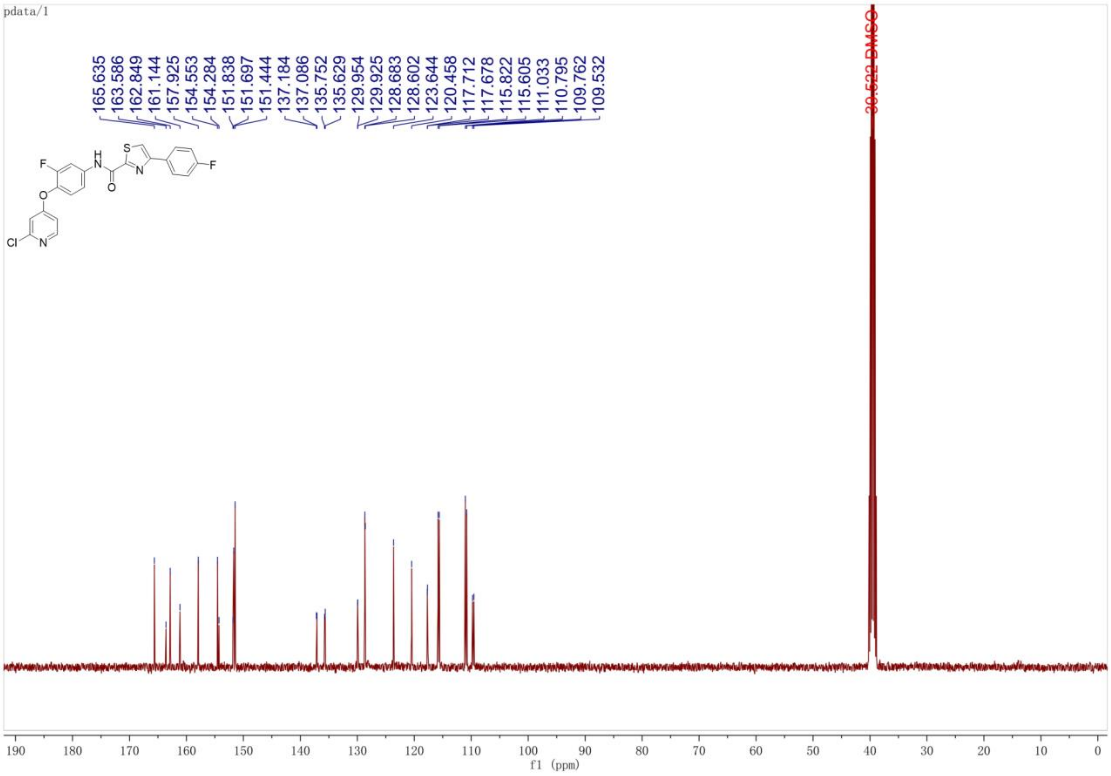

^{1}H NMR Spectrum of **51ab** (400 MHz, DMSO-d_6)

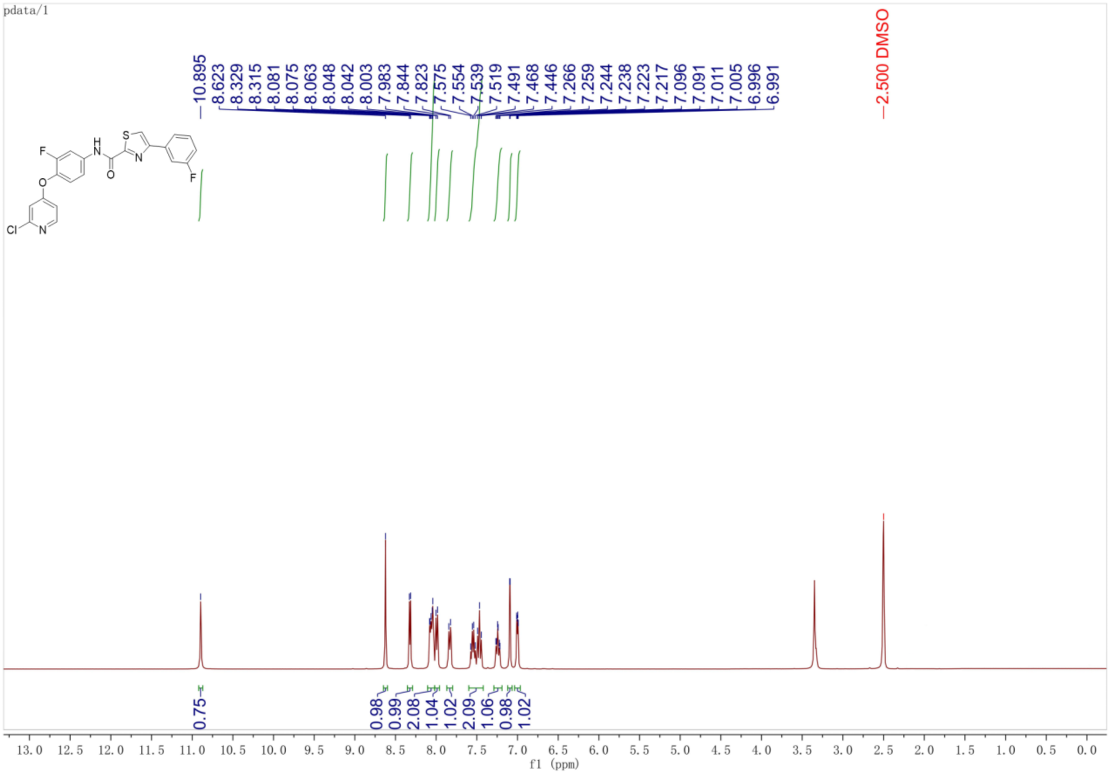

^{13}C NMR Spectrum of **51ab** (100 MHz, DMSO-d_6)

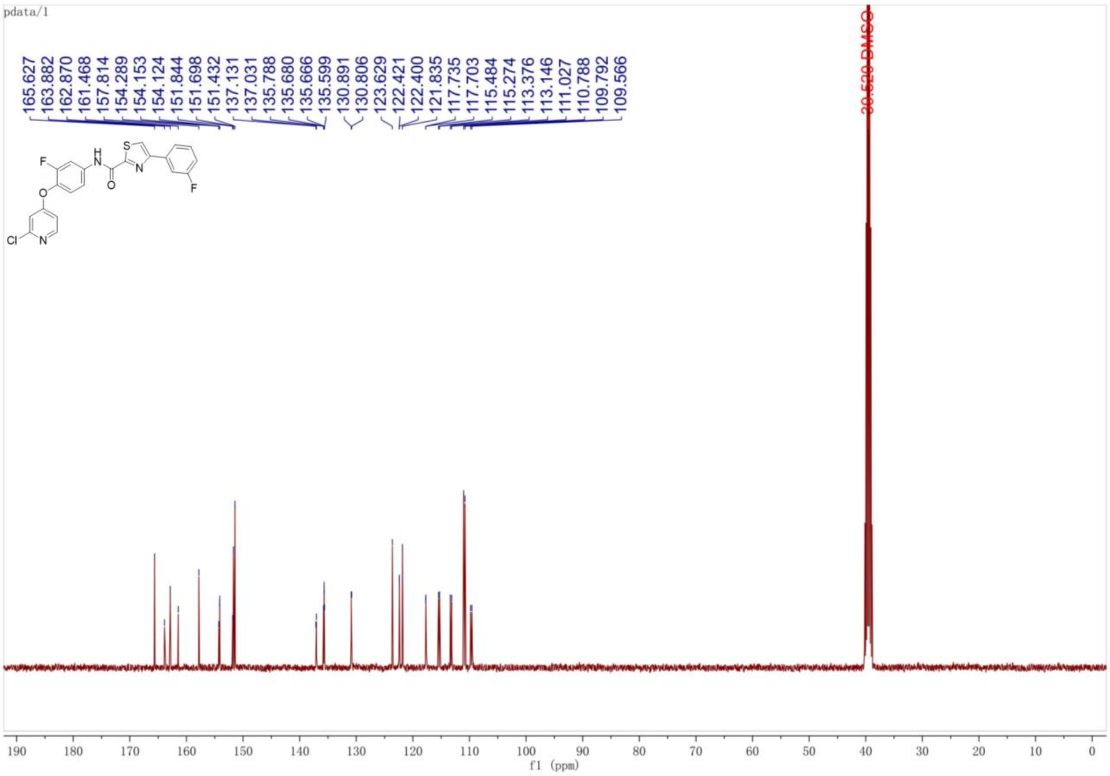

^{1}H NMR Spectrum of **51ac** (400 MHz, DMSO-d_6)

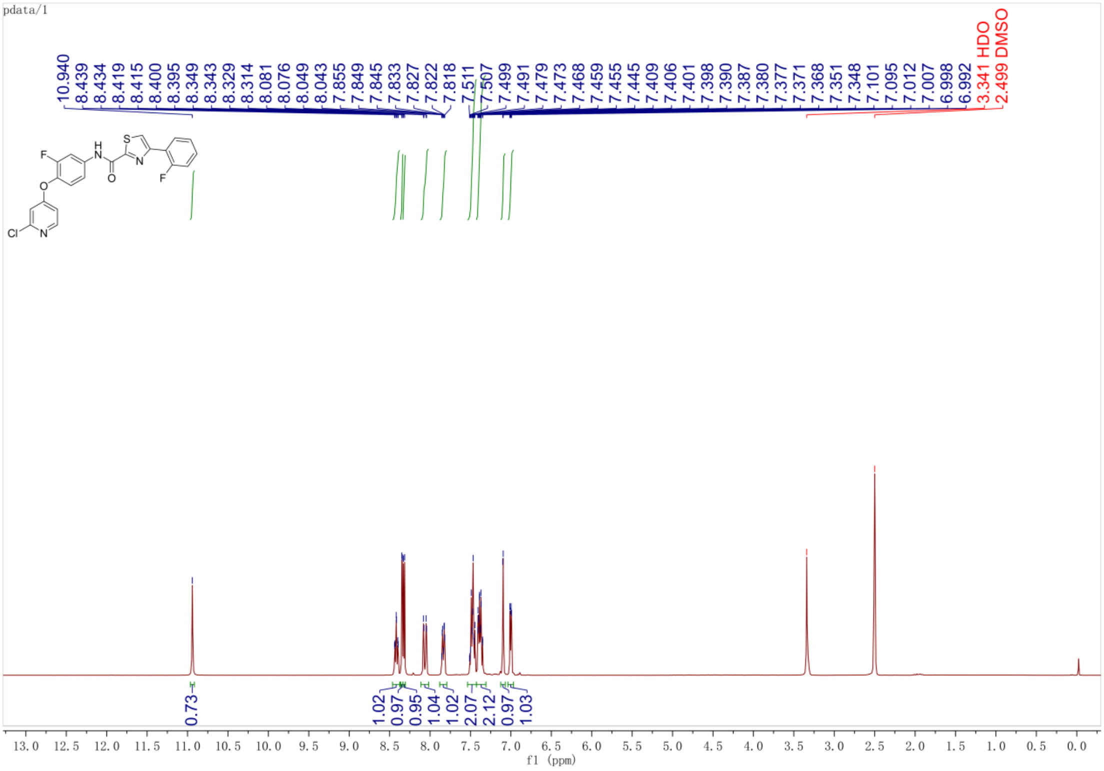

^{13}C NMR Spectrum of **51ac** (100 MHz, DMSO-d_6)

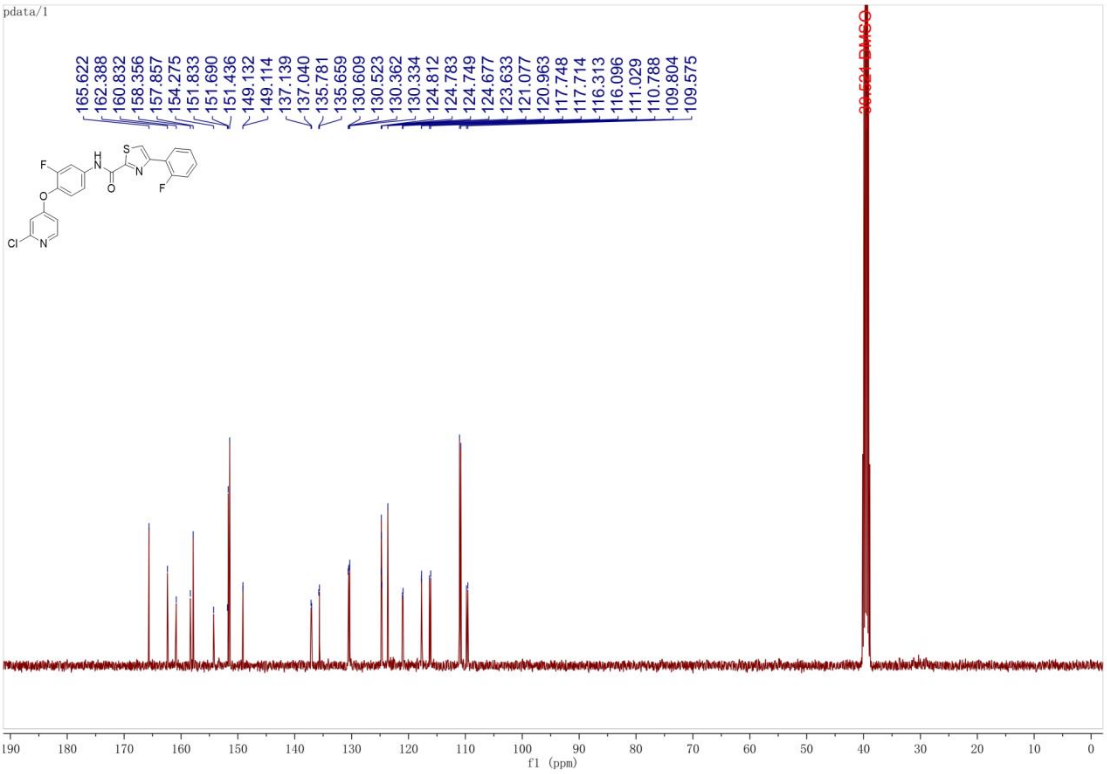

^{1}H NMR Spectrum of **51ad** (400 MHz, DMSO-d_6)

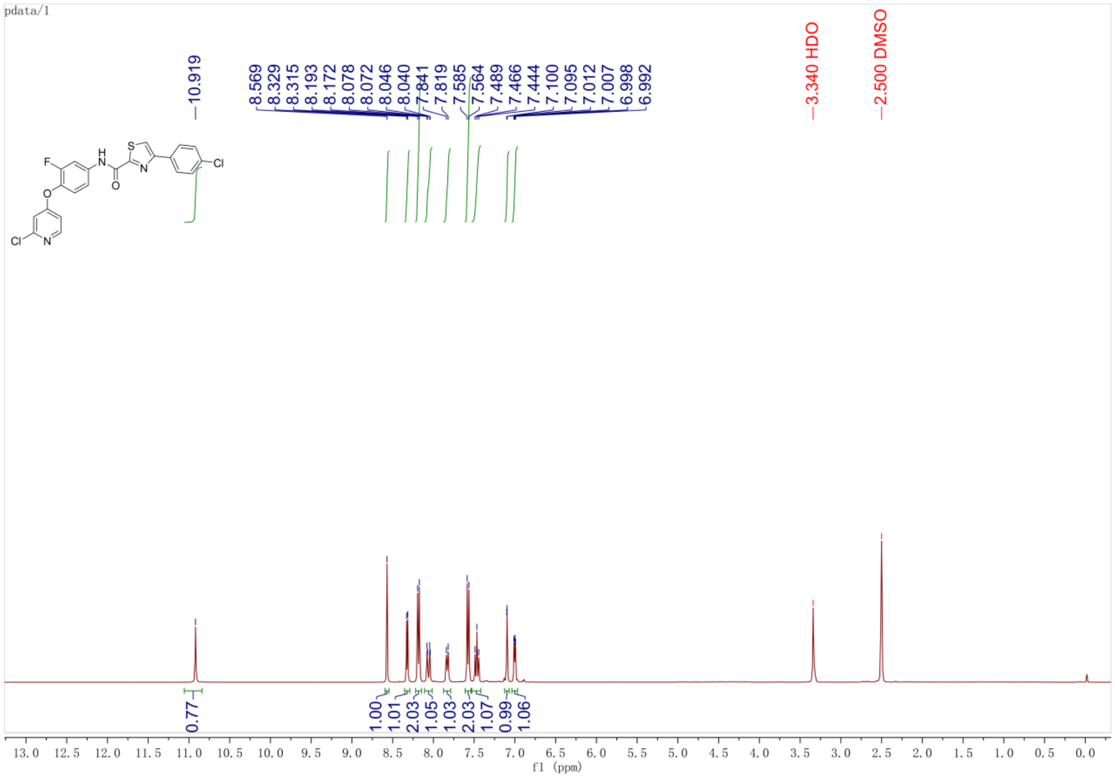

^{13}C NMR Spectrum of **51ad** (100 MHz, DMSO-d_6)

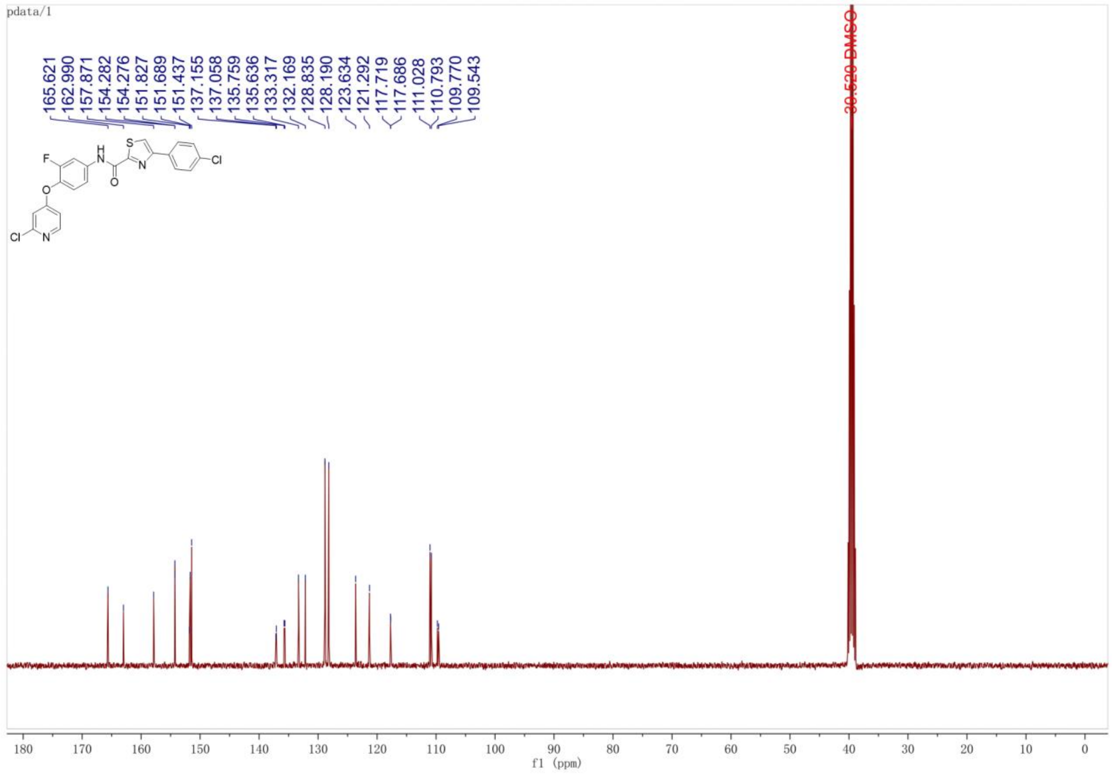

^{1}H NMR Spectrum of **51ae** (400 MHz, DMSO-d_6)

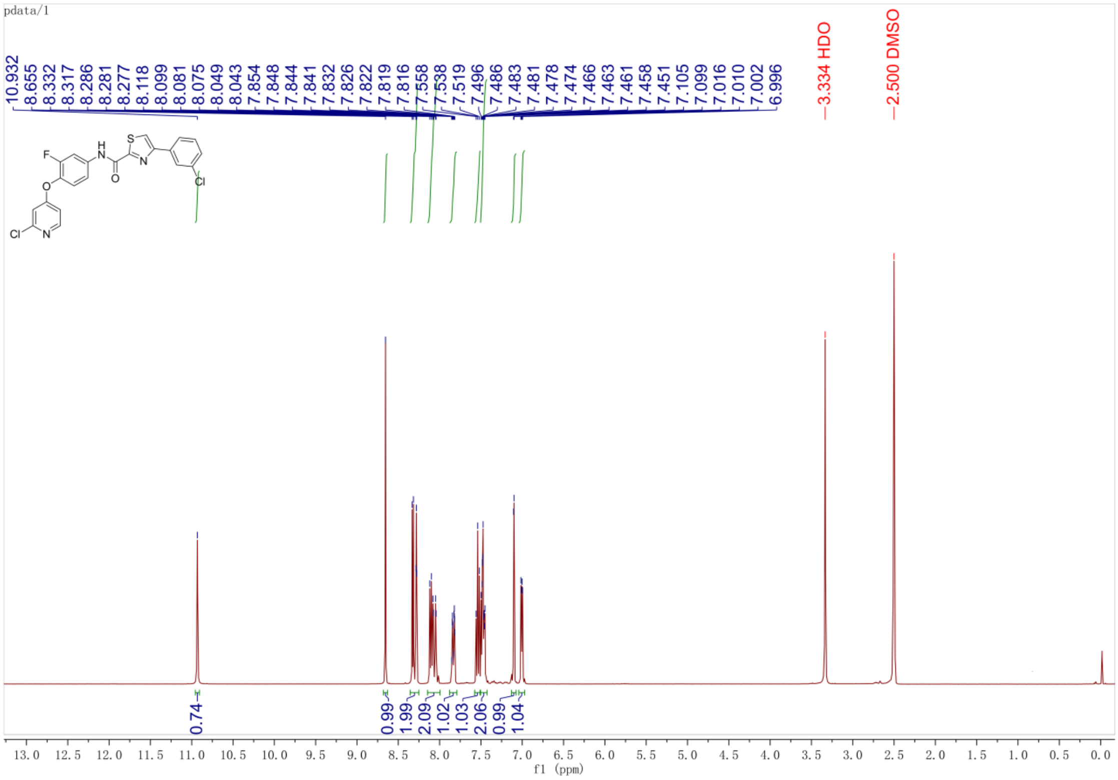

^{13}C NMR Spectrum of **51ae** (100 MHz, DMSO-d_6)

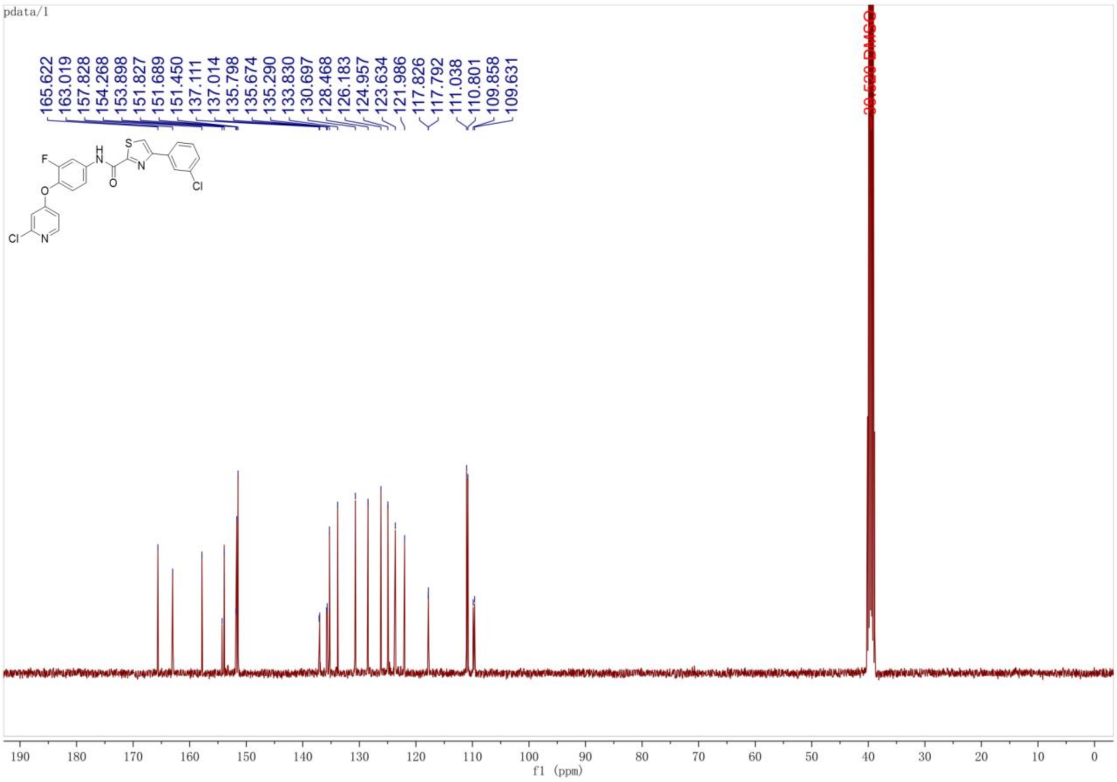

^{1}H NMR Spectrum of **51af** (400 MHz, DMSO-d_6)

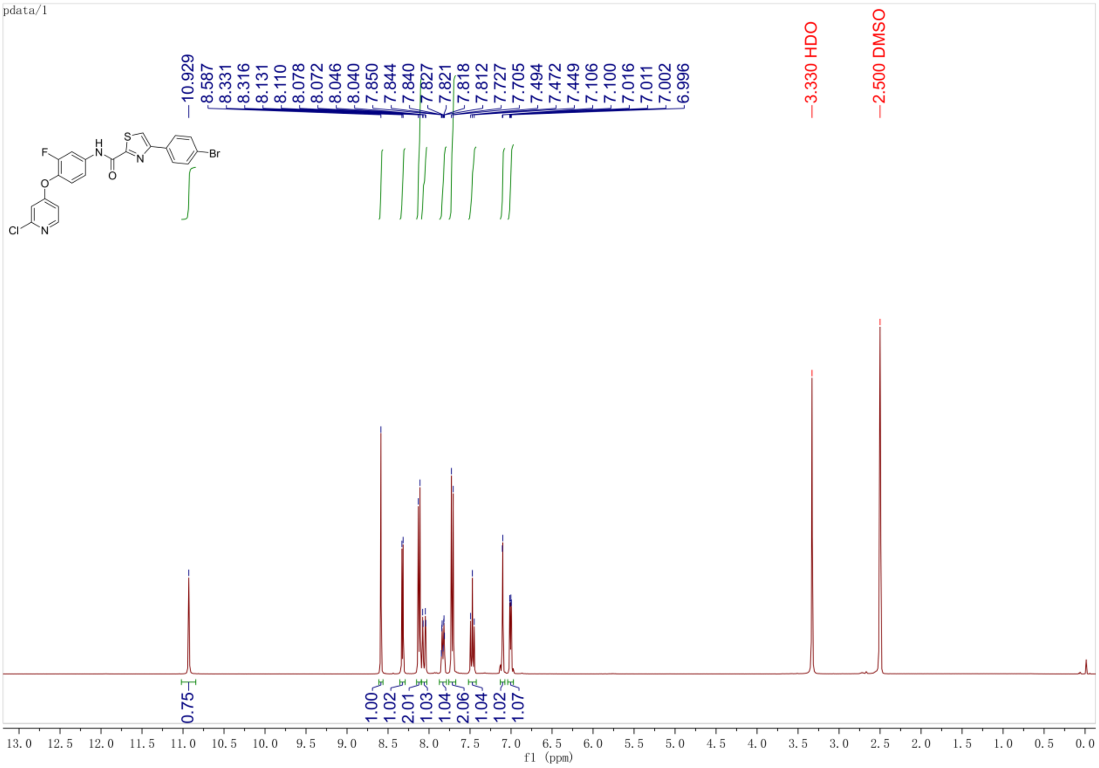

^{13}C NMR Spectrum of **51af** (100 MHz, DMSO-d_6)

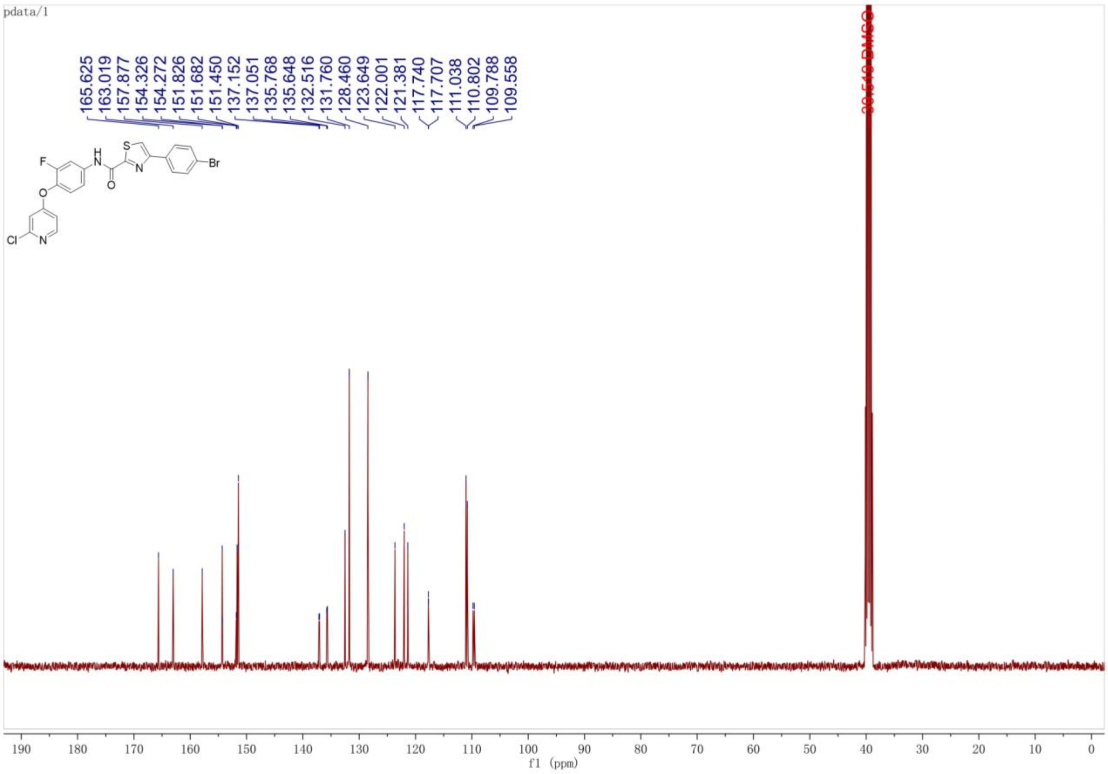

^{1}H NMR Spectrum of **51ag** (400 MHz, DMSO-d_6)

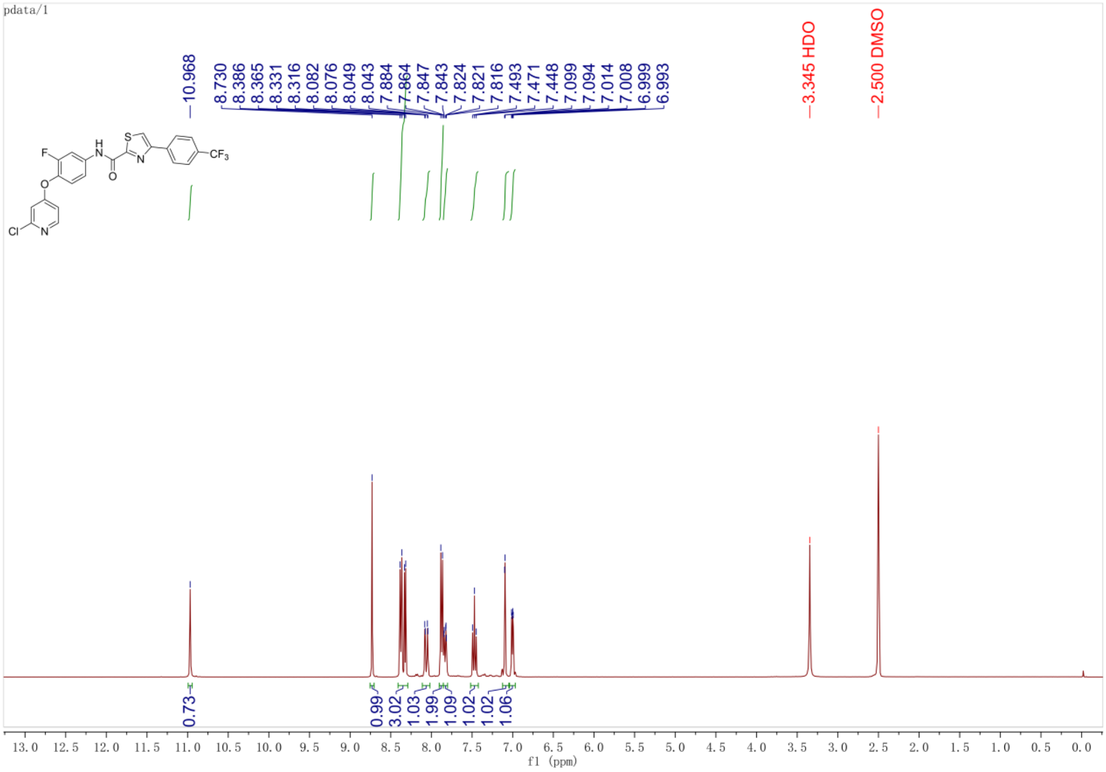

^{13}C NMR Spectrum of **51ag** (100 MHz, DMSO-d_6)

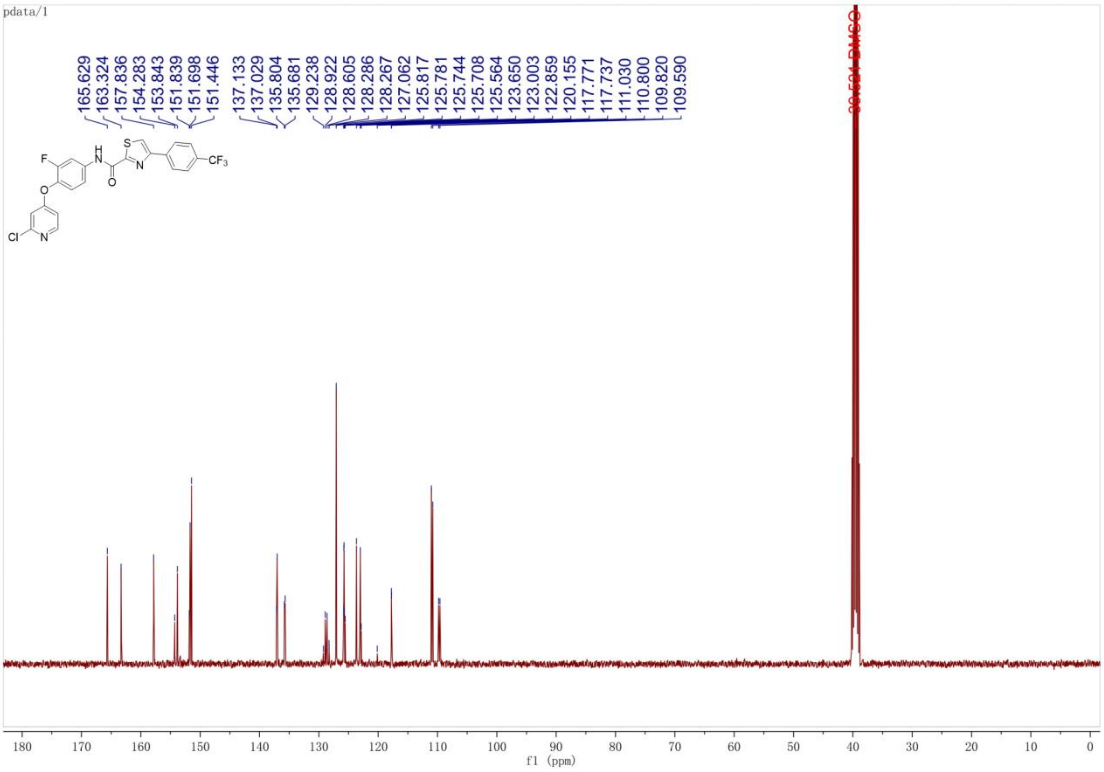

^{1}H NMR Spectrum of **51ah** (400 MHz, DMSO-d_6)

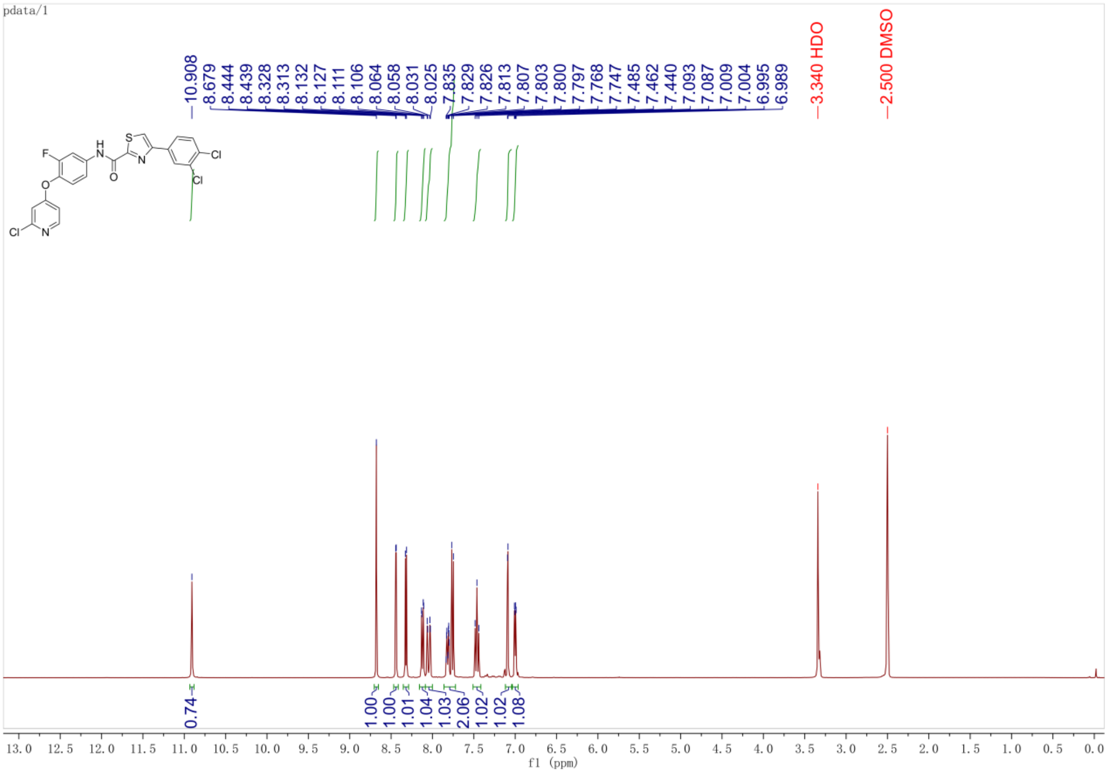

^{13}C NMR Spectrum of **51ah** (100 MHz, DMSO-d_6)

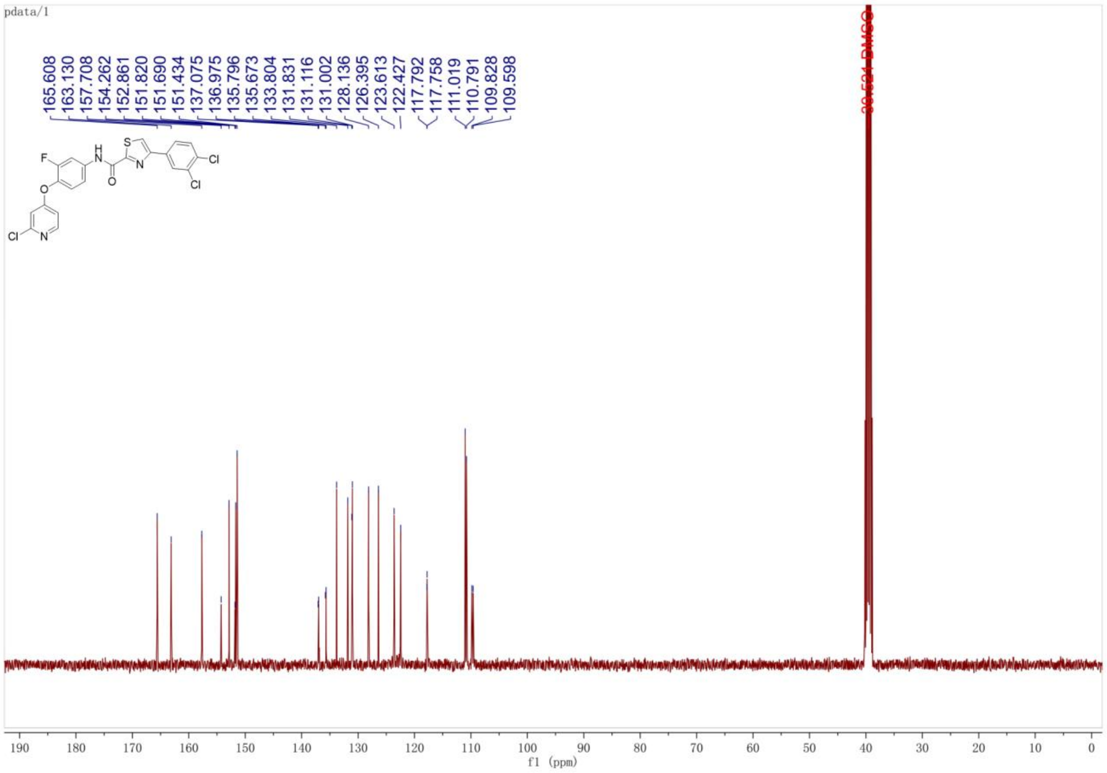

^{1}H NMR Spectrum of **51ai** (400 MHz, DMSO-d_6)

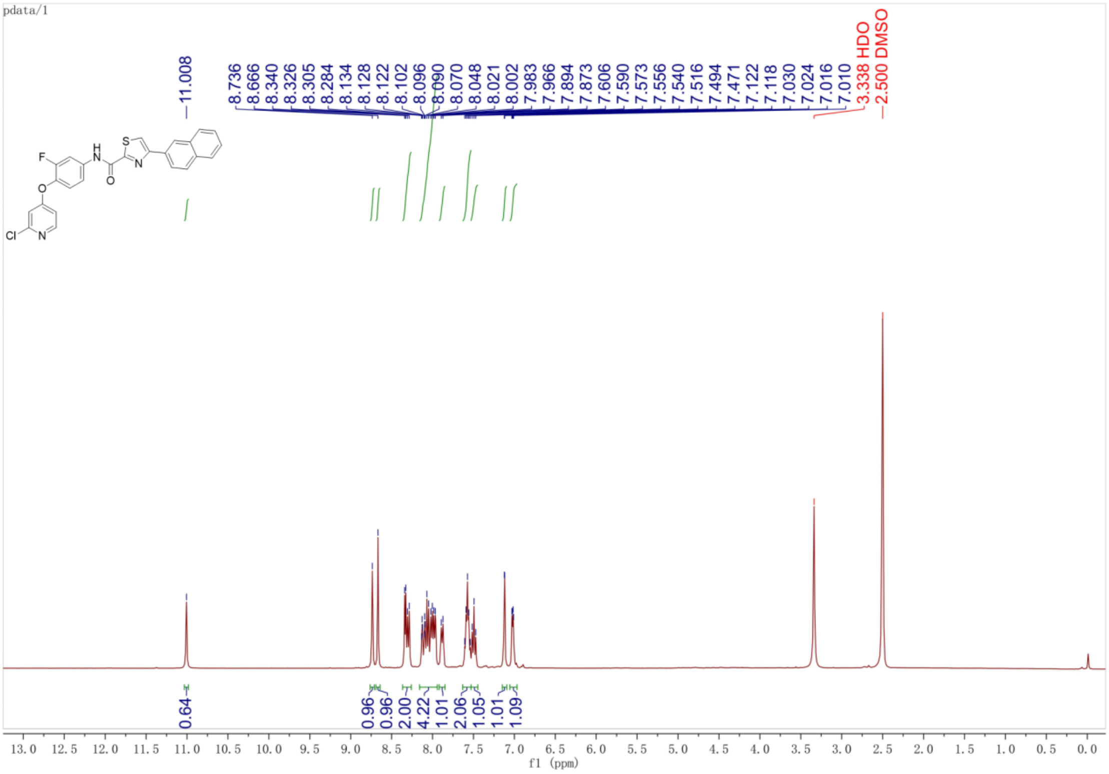

^{13}C NMR Spectrum of **51ai** (100 MHz, DMSO-d_6)

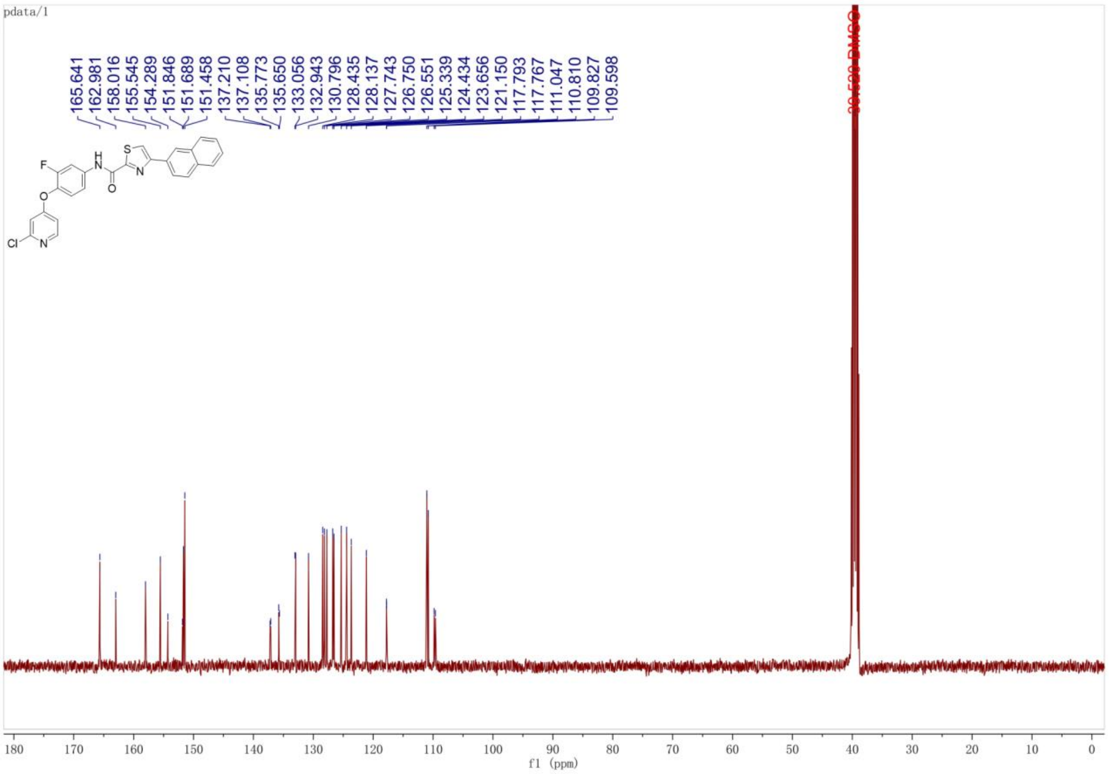

^{1}H NMR Spectrum of **51aj** (400 MHz, DMSO-d_6)

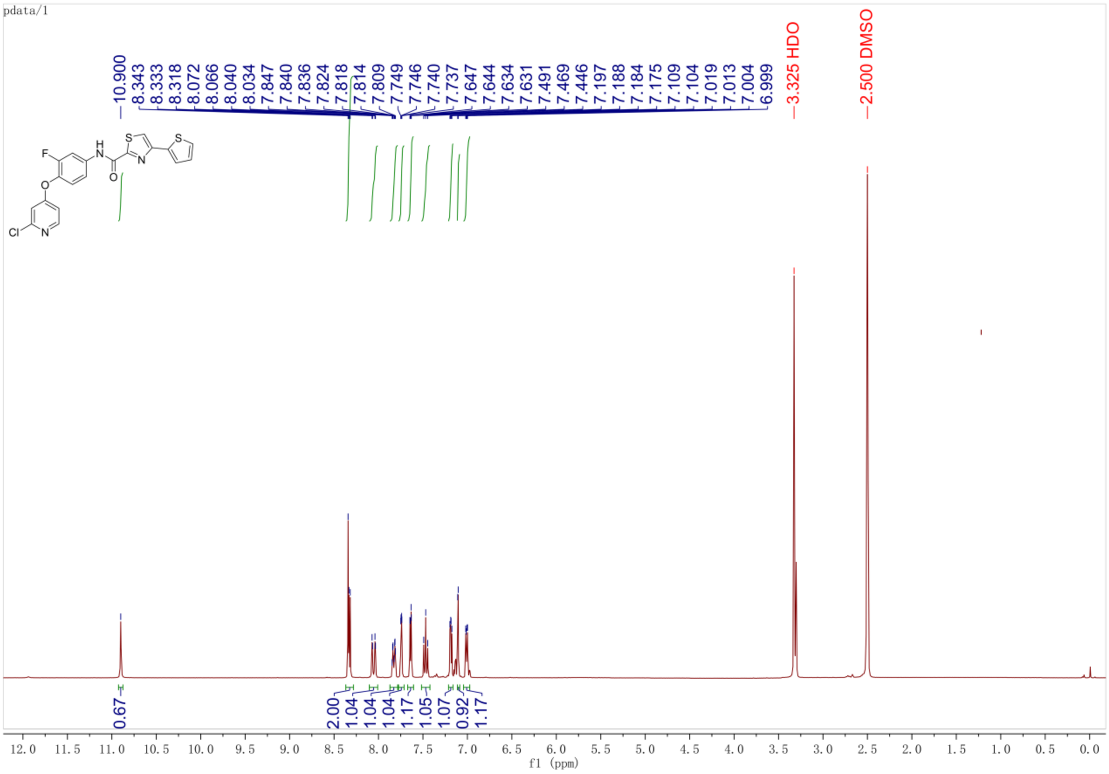

^{13}C NMR Spectrum of **51aj** (100 MHz, DMSO-d_6)

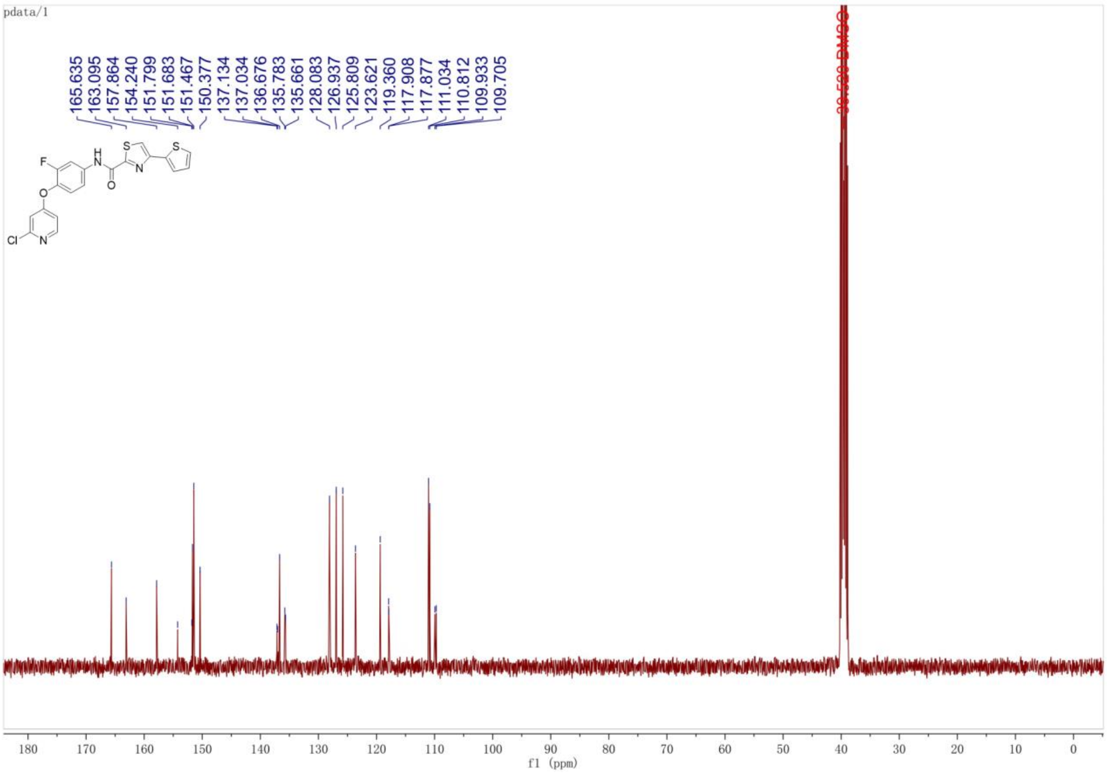

^{1}H NMR Spectrum of **51ak** (400 MHz, DMSO-d_6)

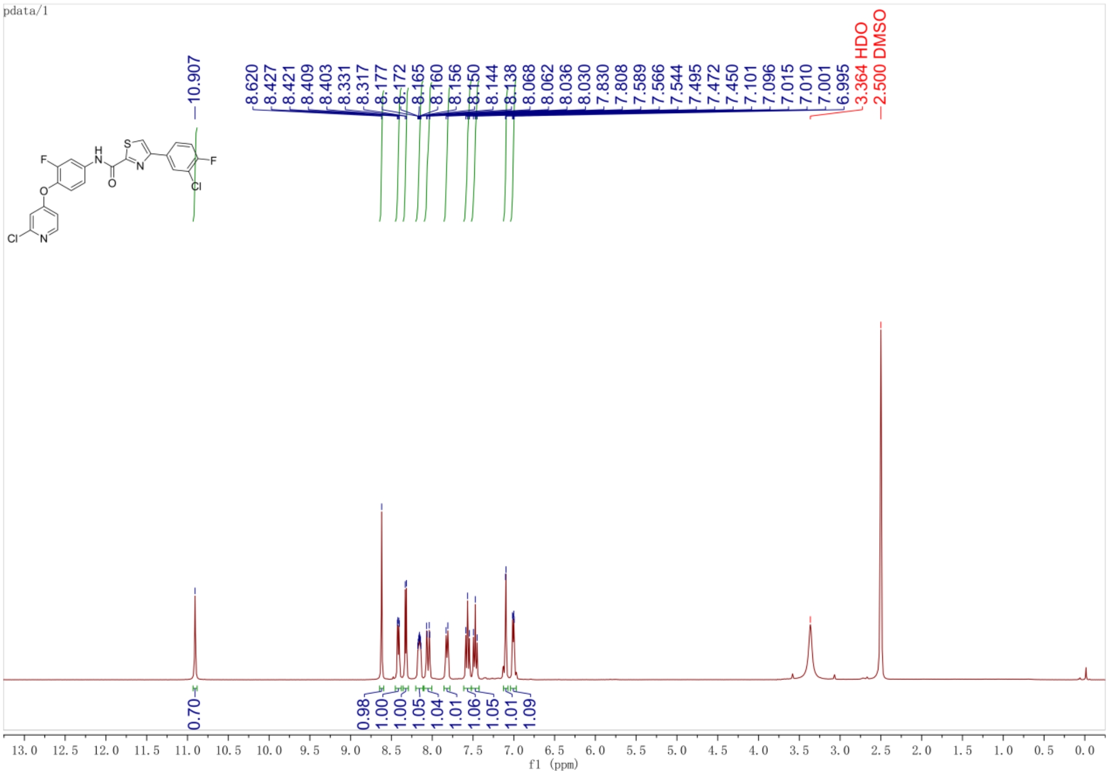

^{13}C NMR Spectrum of **51ak** (100 MHz, DMSO-d_6)

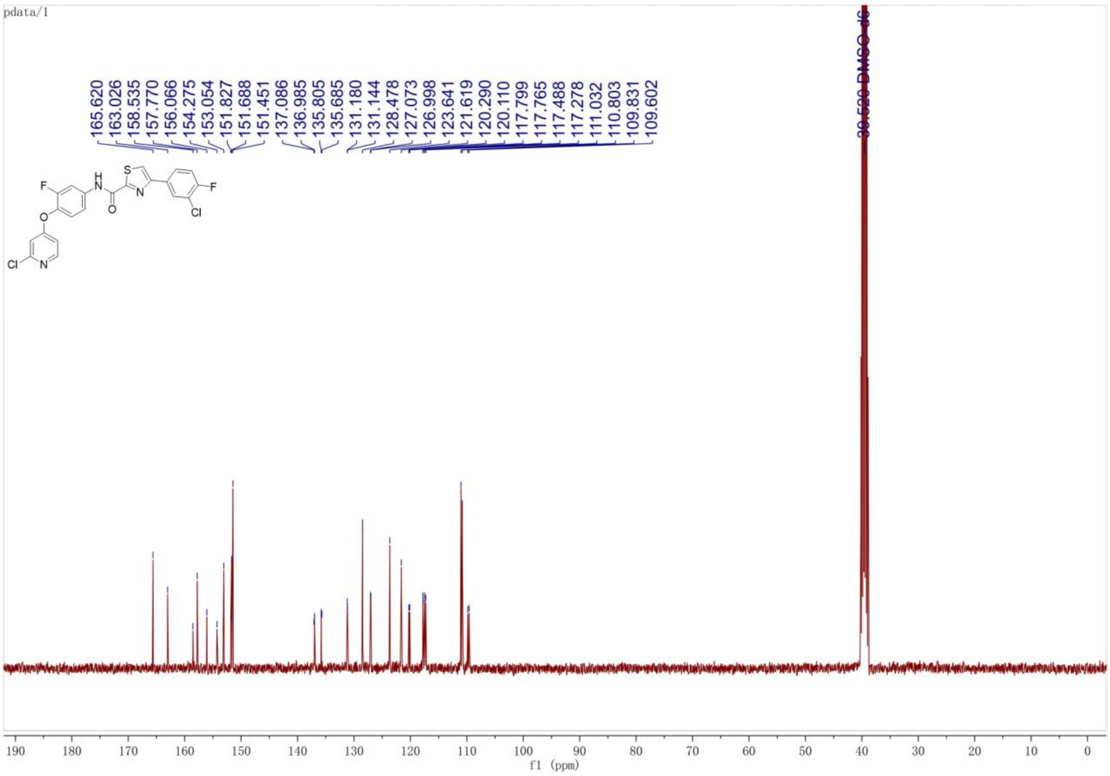

^{1}H NMR Spectrum of **51al** (400 MHz, DMSO-d_6)

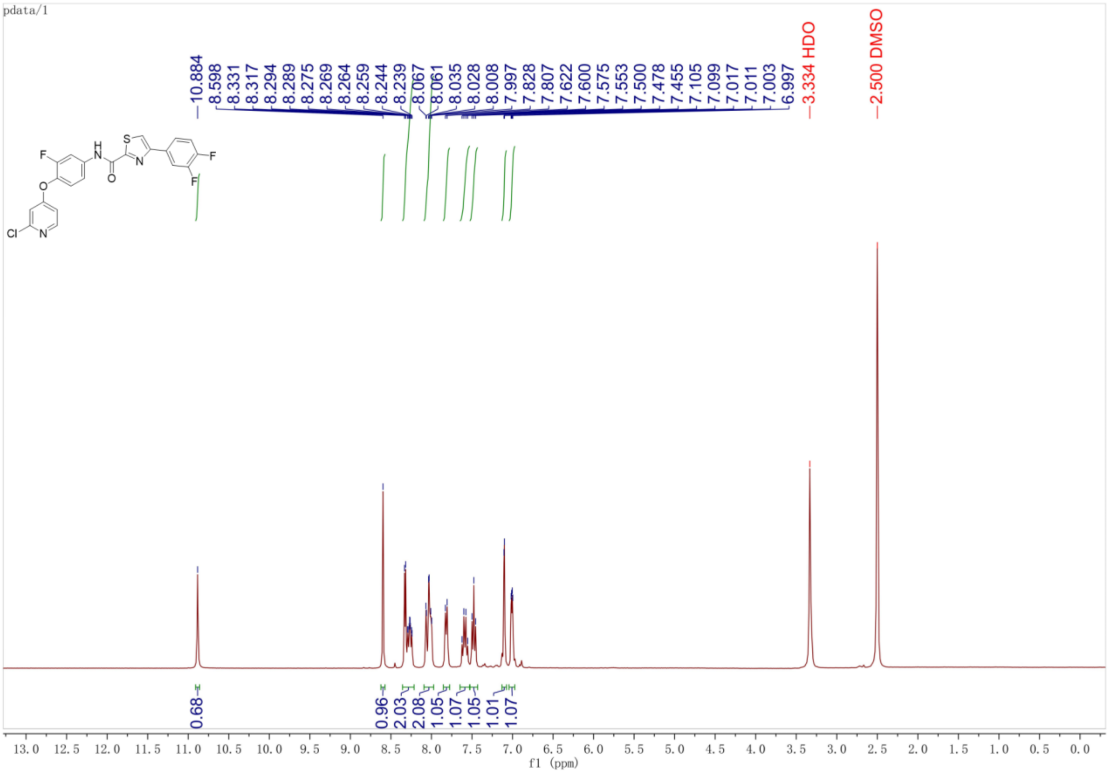

^{13}C NMR Spectrum of **51al** (100 MHz, DMSO-d_6)

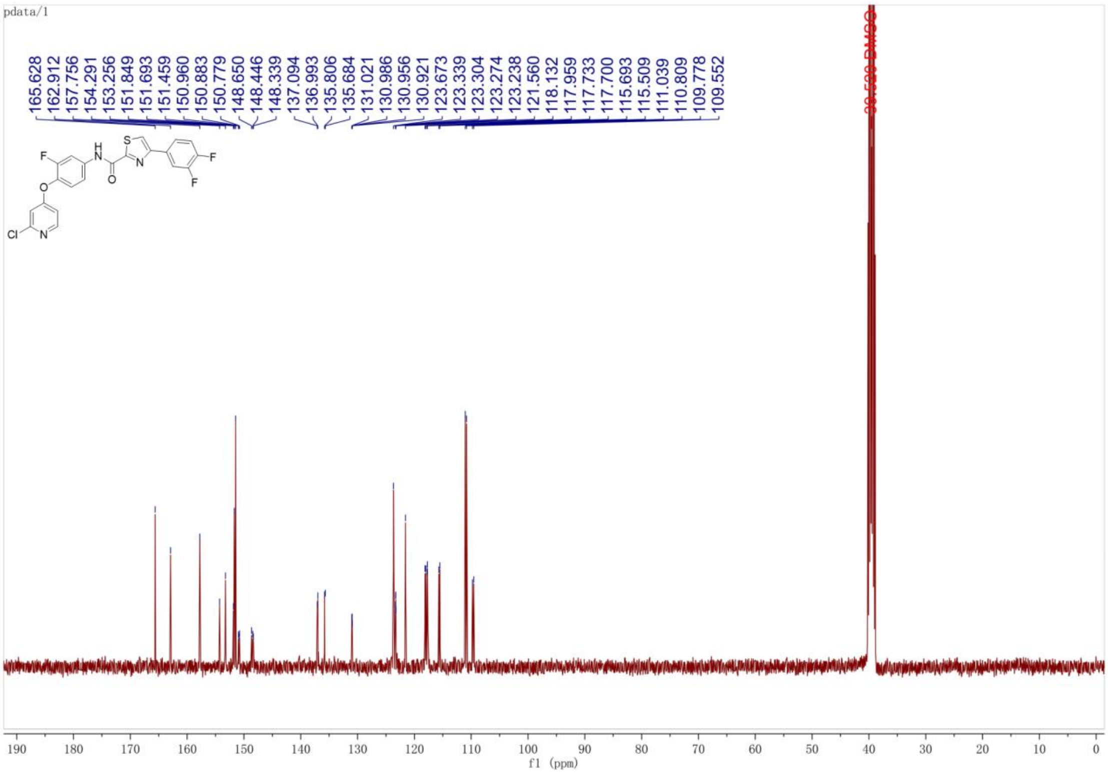

^{1}H NMR Spectrum of **51am** (400 MHz, DMSO-d_6)

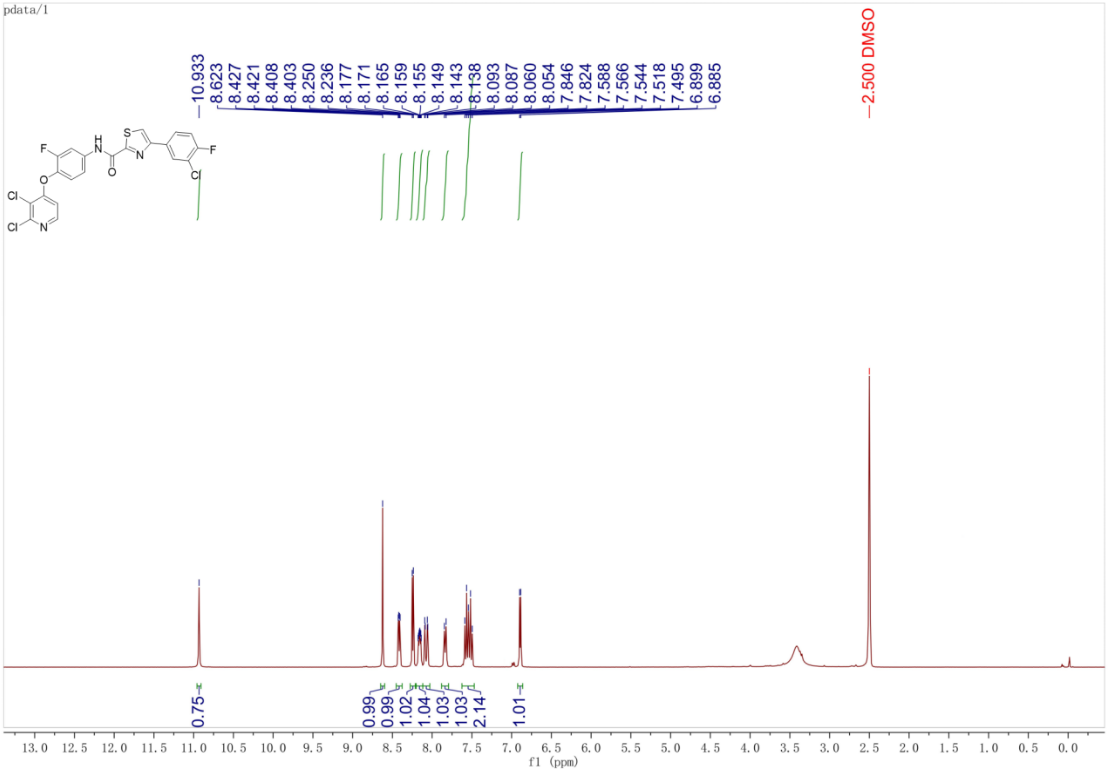

^{13}C NMR Spectrum of **51am** (100 MHz, DMSO-d_6)

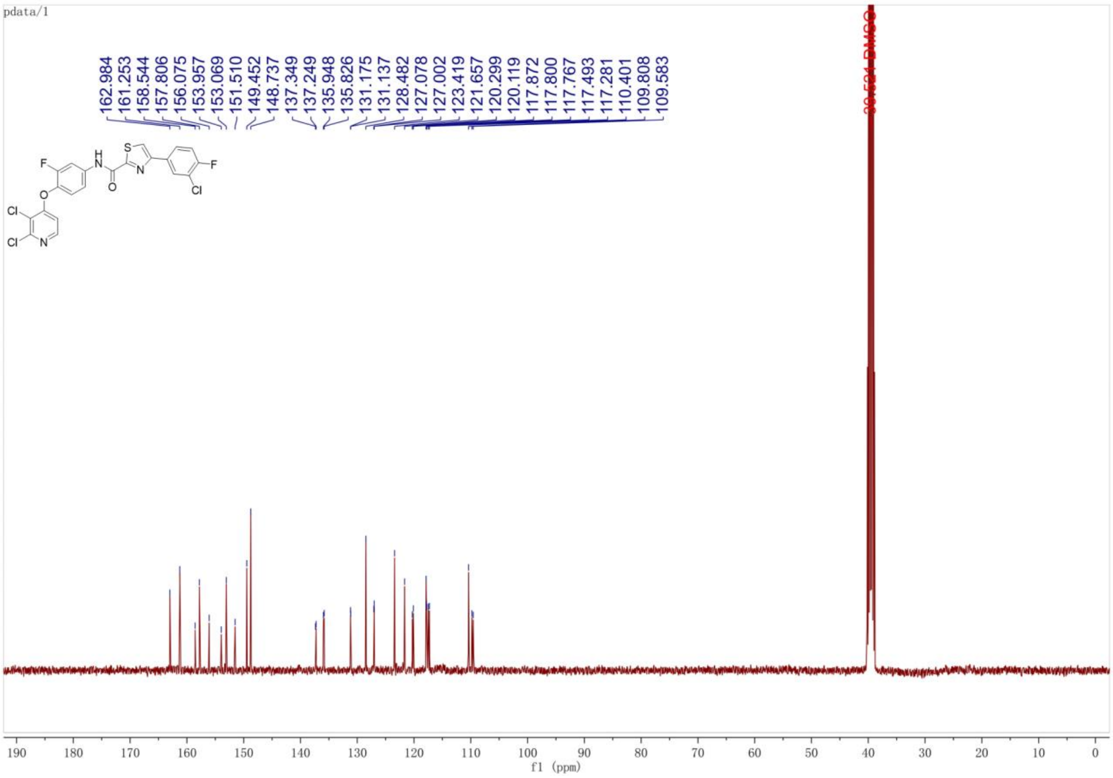

^{1}H NMR Spectrum of **51an** (400 MHz, DMSO-d_6)

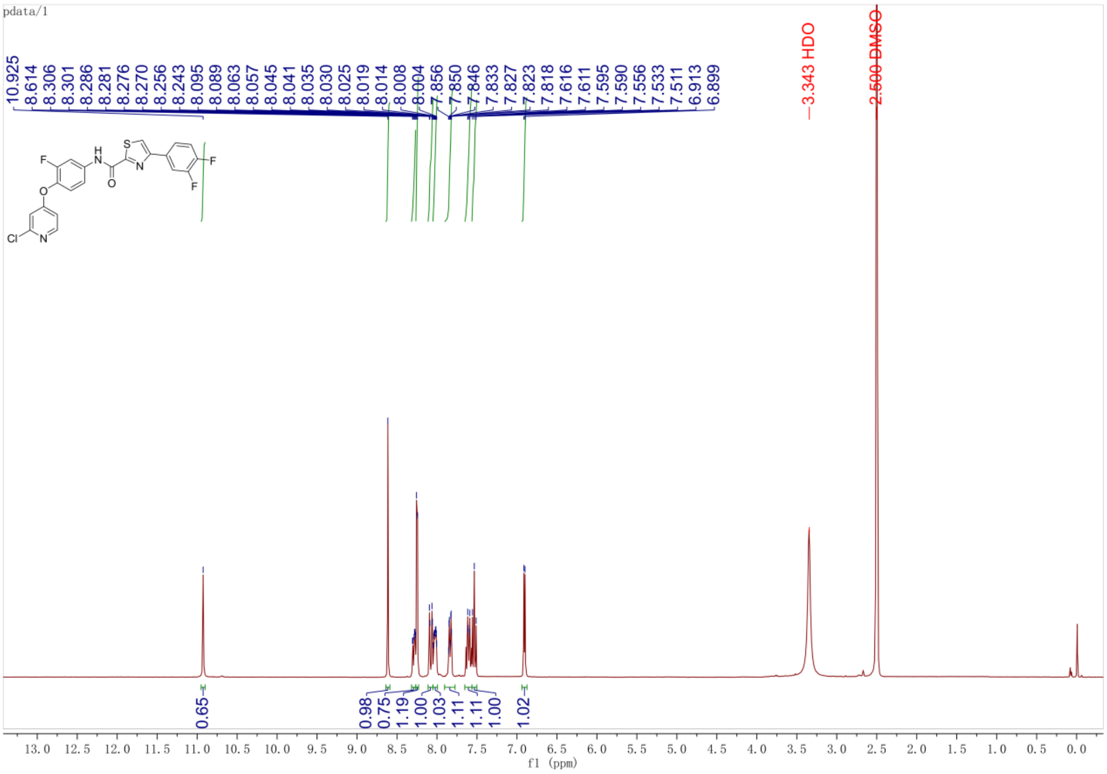

^{13}C NMR Spectrum of **51an** (100 MHz, DMSO-d_6)

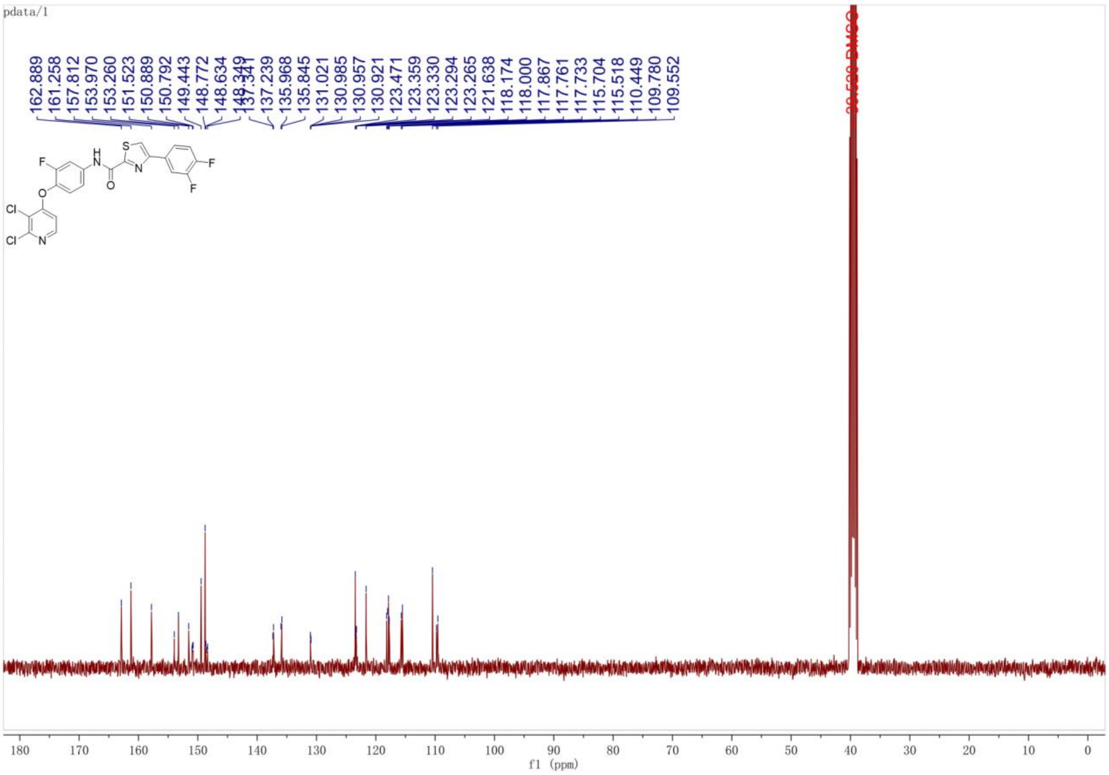

Supplement: Supplemental Material [file IENZ_A_2247183_SM7089.pdf]
